# Supplementary material for: Mission impossible accomplished? A European cross-national comparative study on the integration of the harm-benefit analysis into law and policy documents
Source: PLoS One. 2024 Feb 20;19(2):e0297375. doi: 10.1371/journal.pone.0297375 (PMC10878508; doi:10.1371/journal.pone.0297375)
Supplement: S1 File — (PDF) [file pone.0297375.s001.pdf]

European Commission

Caring for animals
aiming for better science

DIRECTIVE 2010/63/EU
ON PROTECTION OF ANIMALS USED
FOR SCIENTIFIC PURPOSES

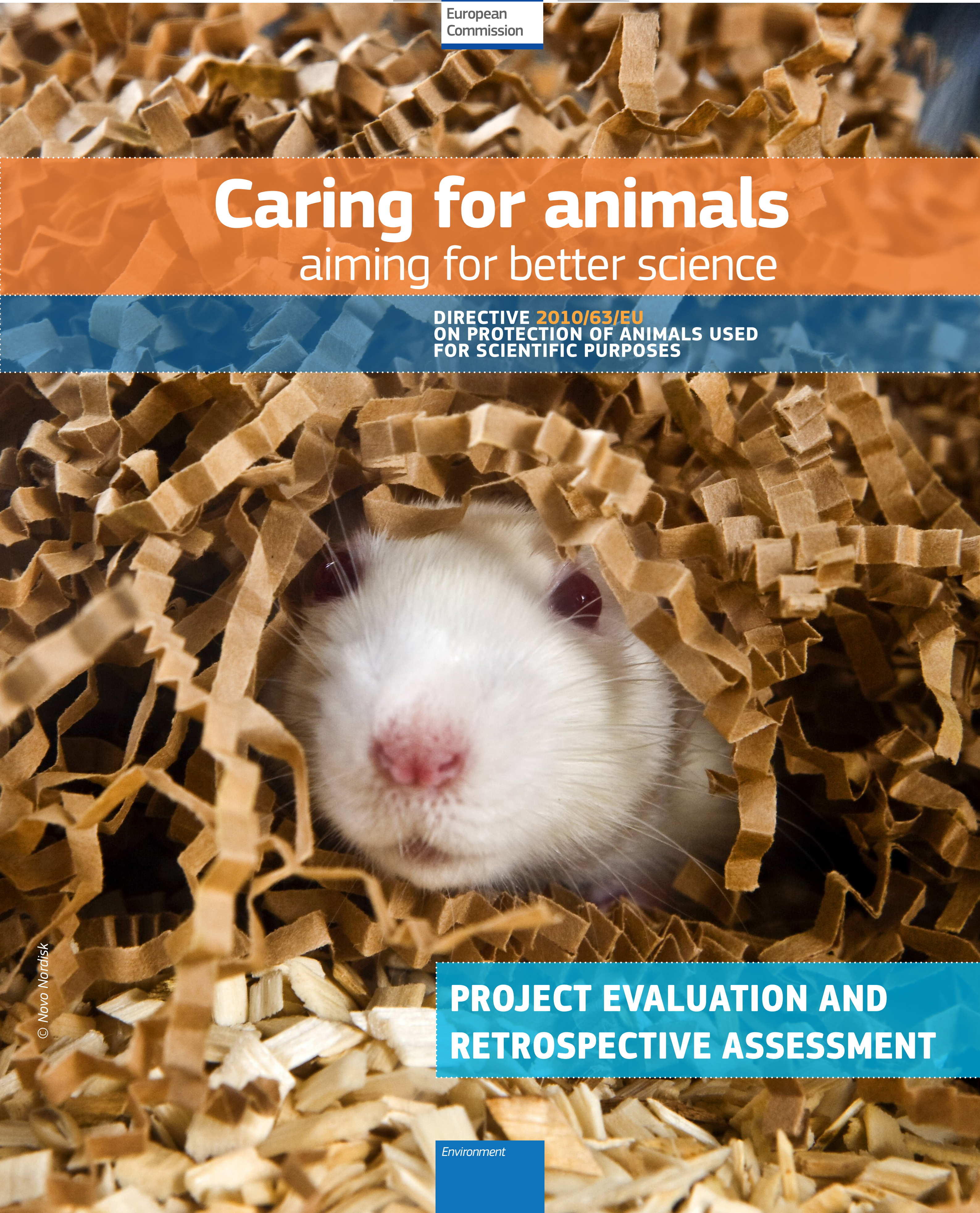

© Novo Nordisk

PROJECT EVALUATION AND RETROSPECTIVE ASSESSMENT

Environment

National Competent Authorities for the implementation of Directive 2010/63/EU on the protection of animals used for scientific purposes

Working document on Project Evaluation and Retrospective Assessment

Brussels, 18-19 September 2013

The Commission established an Expert Working Group (EWG) for Project Evaluation (PE) and Retrospective Assessment (RA) of projects to facilitate the implementation of Directive 2010/63/EU on the protection of animals used for scientific purposes. All Members States and main stakeholder organisations were invited to nominate experts to participate in the work.

The main objectives of the EWG were to develop guidance and principles for PE and RA in line with Articles 38 and 39 of the Directive to assist all those involved in the preparation, evaluation and assessment of projects. The EWG for PE & RA was convened 19-20 March 2013.

This document is the result of the work of the EWG meeting, discussions with the Member States as well as legal input from the Commission on the understanding of the requirements for these two processes, its components, participants and working tools and methods. It was endorsed by the National Competent Authorities for the implementation of Directive 2010/63/EU at their meeting of 18-19 September 2013.

Disclaimer:

The following is intended as guidance to assist the Member States and others affected by Directive 2010/63/EU on the protection of animals used for scientific purposes to arrive at a common understanding of the provisions contained in the Directive and to facilitate its implementation. All comments should be considered within the context of this Directive 2010/63/EU. It provides some suggestions on how the requirements of the Directive may be met. The content of the document does not impose additional obligations beyond those laid out in the Directive.

Only the Court of Justice of the European Union is entitled to interpret EU law with legally binding authority.

The table of contents

Introduction 4
The related articles of Directive 2010/63/EU 5
Information requirements 6
Examples of problems encountered with project applications 7
Project application 8
Use of templates for project application 8
The level of detail in project applications 9
Use of declarations 10
Formulating specific questions 10
Project evaluation process (who/how) 12
Principles for an effective project evaluation process 12
Models used in project evaluation process 12
How can these principles be addressed in practice? 13
Consideration of different methodologies 16
Additional comments on project evaluation process 18
Evaluation of the scientific justification for exemptions and application of the Three Rs 18
Evaluation of the scientific justification for exemptions 18
Evaluation of the application of the Three Rs 19
Performing a harm-benefit analysis 20
Factors to take into consideration in assessing benefits 21
Factors to take into consideration in assessing harms 23
Factors to take into consideration in assessing likelihood of success 24
Evaluation of the project application 24
How to weigh harms and benefits 25
How to perform a harm-benefit analysis 26
Retrospective assessment 28
The benefits of carrying out retrospective assessment 28
Factors to determine whether or not a retrospective assessment should be carried out 28
The most appropriate time to carry out a retrospective assessment 29
Securing the necessary information for a retrospective assessment 29

Guidance on information requirements 30
Outcomes to derive from a retrospective assessment 31
General recommendations 32
Bibliography 33
Appendix I: Pre-formulated questions for building a project application template 34
Requirements of Annex VI 34
Use of live animals for the purposes of education and training 38
Request for an exemption 39
Appendix II: Modified Bateson Cube 40
Appendix III: Further guidance on issues to consider in retrospective assessment 41

Introduction

Directive 2010/63/EU on the protection of animals used for scientific purposes requires that "*no project is carried out unless a favourable project evaluation by the competent authority has been received*".

As per recitals 39 and 40 of the Directive, "*it is essential, both on moral and scientific grounds, to ensure that **each use of an animal is carefully evaluated** as to the scientific or educational validity, usefulness and relevance of the expected result of that use. The likely harm to the animal should be balanced against the expected benefits of the project.*

*Therefore, an **impartial** project evaluation **independent of those involved in the study** should be carried out as part of the authorisation process of projects involving the use of live animals. Effective implementation of a project evaluation should also allow for an appropriate assessment of the use of any new scientific experimental techniques as they emerge."*

Furthermore, it provides that "*due to the **nature of the project**, the **type of species** used and the **likelihood of achieving the desired objectives** of the project, it might be necessary to carry out a retrospective assessment. Since projects may vary significantly in terms of complexity, length, and the time period for obtaining the results, it is necessary that the decision on retrospective assessment should be made taking those aspects fully into account*."

The key findings of this report highlight the importance of correct, complete, current and relevant provision of information which can be facilitated by well-designed templates accompanied by appropriate guidance. Training of all involved in these processes is crucial. The different approaches to project evaluation/retrospective assessment each have advantages and disadvantages. It is therefore essential that due consideration is given to the requirements of the Directive as to how these are best achieved in a given structure.

An effective harm-benefit analysis requires a good understanding of the nature and impact of the potential benefits, of all of the expected harms to the animals, taking into account all refinement measures, and the likelihood of achieving the projected benefits. The end result is based on an analysis of this three-dimensional weighing through informed discussion among well-trained evaluators with relevant expertise. Consistency in the process can be achieved over time with appropriate feed-back mechanisms in place and co-operation and exchange of information among all those involved at local, national and international level.

The related articles of Directive 2010/63/EU

Article 37 – Application for project authorisation

"1. Member States shall ensure that an application for project authorisation is submitted by the user or the person responsible for the project. The application shall include at least the following:

(a) the project proposal;

(b) a non-technical project summary; and

(c) information on the elements set out in Annex VI.

2. Member States may waive the requirement in paragraph 1(b) for projects referred to in Article 42(1)."

Article 38 – Project evaluation

"1. The project evaluation shall be performed with a degree of detail appropriate for the type of project and shall verify that the project meets the following criteria:

(a) the project is justified from a scientific or educational point of view or required by law;

(b) the purposes of the project justify the use of animals; and

(c) the project is designed so as to enable procedures to be carried out in the most humane and environmentally sensitive manner possible.

2. The project evaluation shall consist in particular of the following:

(a) an evaluation of the objectives of the project, the predicted scientific benefits or educational value;

(b) an assessment of the compliance of the project with the requirement of replacement, reduction and refinement;

(c) an assessment and assignment of the classification of the severity of procedures;

(d) a harm-benefit analysis of the project, to assess whether the harm to the animals in terms of suffering, pain and distress is justified by the expected outcome taking into account ethical considerations, and may ultimately benefit human beings, animals or the environment;

(e) an assessment of any justification referred to in Articles 6 to 12, 14, 16 and 33; and

(f) a determination as to whether and when the project should be assessed retrospectively.

3. The competent authority carrying out the project evaluation shall consider expertise in particular in the following areas:

(a) the areas of scientific use for which animals will be used including replacement, reduction and refinement in the respective areas;

(b) experimental design, including statistics where appropriate;

(c) veterinary practice in laboratory animal science or wildlife veterinary practice where appropriate;

(d) animal husbandry and care, in relation to the species that are intended to be used.

4. The project evaluation process shall be transparent.

Subject to safeguarding intellectual property and confidential information, the project evaluation shall be performed in an impartial manner and may integrate the opinion of independent parties."

Article 39 – Retrospective assessment

"1. Member States shall ensure that when determined in accordance with Article *38(2)(f), the retrospective assessment shall be carried out by the competent authority which shall, on the basis of the necessary documentation submitted by the user, evaluate the following:*

(a) whether the objectives of the project were achieved;

(b) the harm inflicted on animals, including the numbers and species of animals used, and the severity of the procedures; and

(c) any elements that may contribute to the further implementation of the requirement of replacement, reduction and refinement.

2. All projects using non-human primates and projects involving procedures classified as 'severe', including those referred to in Article 15(2), shall undergo a retrospective assessment.

3. Without prejudice to paragraph 2 and by way of derogation from Article 38(2)(f), Member States may exempt projects involving only procedures classified as 'mild' or 'non- recovery' from the requirement for a retrospective assessment."

Information requirements

The quality of information made available for the evaluators and assessors plays a fundamental role for both PE (and RA). It is therefore of utmost importance that the submitted information is *complete*, *correct*, *current* and *relevant*.

There needs to be a mechanism for ensuring provision of sufficient good quality information, with evidence that the applicant has considered and understood all the relevant issues, to facilitate a well-informed harm-benefit analysis.

Directive 2010/63/EU includes specific information requirements for applications for projects[1], to enable a project evaluation to be carried out.

An application for a project authorisation shall include a project proposal, non-technical project summary[2] and specific information as set out in Annex VI of the Directive. This includes *inter alia* relevance and justification for the use of animals, the application of the Three Rs, severity classification, housing and care conditions, methods of killing, where applicable, and the competence of persons involved in the project.

The Directive also requires specific justification for the use of certain types or sources of animal, methods of killing other than those listed in Annex IV, and for work that is carried outside a user establishment.

It is in the applicant's and evaluator's best interest to ensure that all relevant information is included and presented in sufficient detail to facilitate an effective evaluation of the harms and benefits of the project.

Although incomplete information in an application is likely to delay the evaluation of a project, overly detailed and poorly focused submissions are also likely to result in delays.

Over time, Member States have developed tools to assist applicants in the preparation of applications to use live animals in scientific procedures. These commonly include:

- *An application form* with questions which will deliver information and prompt proper consideration of the issues (rather than encouraging a simple tick box approach).

- *Guidance* on the nature of information expected for each requirement.

Examples of problems encountered with project applications

- Failing to adequately explain benefits
  - Lacking the wider context of the research programme (and potential benefits of the specific project to the overall research programme);
  - Benefits not sufficiently described or explained - especially in the area of basic research;

[1] **Project:** a project means a coherent programme of work having a defined scientific objective(s) and involving one or more procedures. Projects can vary in size and complexity, for example, from the work of a single scientist consisting of a single blood harvest procedure in a single species, to an entire department's drug discovery programme, which involves many scientists, multiple complex procedures and a wide range of species. http://ec.europa.eu/environment/chemicals/lab_animals/pdf/Consensus_document.pdf

[2] http://ec.europa.eu/environment/chemicals/lab_animals/pdf/Recommendations%20for%20NTS.pdf

- Unsubstantiated/unrealistic claims of potential benefits;
- Benefits not linked to the objectives set out in the application;
- Not indicating the timescale when benefits may be expected (when feasible).

- Failing to sufficiently address likelihood of success i.e. likelihood of attaining the objectives set for the project
  - No information on group's (or establishment's) track record (for example, previous experience; relevant publications; resource availability, including animal facilities and funding) to help assess likelihood of success;
  - Justification for the work not well structured, lack of key indicators of success, insufficient focus and relevance;
  - Insufficient details to allow evaluation of the likelihood of achieving success;
  - Insufficient details on animal models (and where applicable, the use of Genetically Altered Animals [GAs]) and why they were chosen;
  - Insufficient information on how the procedures contribute to the objectives of the project.

- Failing to sufficiently address the application of the Three Rs
  - Insufficient information necessary to consider whether or not all 3Rs have been addressed – for example missing information on how harms are reduced to a minimum consistent with scientific objectives and no justification given for circumstances where recognised good practices are not employed e.g. use of analgesia ; social housing.

- Failing to adequately estimate harms
  - Procedures on animals not sufficiently detailed to estimate harms to individual animals;
  - No information on nature and level of harms or information on welfare assessment or humane end-points.

Project application

Use of templates for project application

The use of templates for project applications is considered to be helpful provided the right questions are asked.

A template serves two important purposes:

- To facilitate the provision of correct, complete, accurate, relevant, and timely information on the project;
- To provoke the applicant to consider all elements of the scientific work and how and where the Three Rs should be applied.

<u>General considerations on the use of templates include</u>

- The use of Yes/No questions and tick-boxes should be limited to circumstances when no further clarification is necessary.
- Questions should provoke thinking – using terms such as ‘explain how’, 'explain why' ‘describe’, 'demonstrate', 'show' encourage a correct and complete application.
- A recommended maximum number of words could encourage applicants to focus on the essential and relevant information – acknowledging that these may be exceeded in certain complex applications.
- An understanding and verification of scientific hypotheses and rationale are needed to assess the potential benefits of a project and the likelihood of achieving these.
- Scientific evaluation (for funding/grant application purposes) is seldom sufficient alone to satisfy the requirements for PE. This does not necessarily include important elements such as ethical considerations, assessment of societal benefits or application of the Three Rs.
- It is important to use a simple and easily understandable language.

The level of detail in project applications

There are elements that impact the level of detail in a project application. These are not easy to demonstrate in a simple template, without some additional explanatory guidance. It may be useful to establish different templates for different types of projects.

The following considerations on the level of information should be taken into account when designing (a) template(s) for project applications.

- The level of detail ***may vary*** based on
  - Type of project (e.g. regulatory work, basic research, education);
  - Species and numbers of animals and their level of sentience;
  - Scale of project, complexity, novelty (e.g. pilot studies);
  - Duration of project;
  - Expected severity of the procedures in the project.

- Elements that ***should <u>not</u> vary*** in level of detail include
  - Application of the Three Rs (e.g. refinement, use of humane end-points and pain relief, housing and care practices);
  - Individual procedures and how the related severity classification translates to the numbers of animals involved, which is essential for the assessment of the overall harms expected in the project. The severity classification for each of the procedures relates to the worst case scenario for a single animal *within a procedure*. For the *harms of the project* to be described and assessed, the application needs to include ‘expected’ severities for *all the animals involved in the project*.

Even with a well-structured application form and helpful guidance, further information is sometimes required by the evaluator – the argument given is that with the breadth of project content it can be difficult to obtain the necessary information using a standard form and it is necessary on occasion to give specific case-by-case prospective guidance. Such discussion should, where possible, take place before the project application is submitted, to facilitate the PE process by ensuring provision of any additional information or clarification at the point of application.

Use of declarations

There may be some information requirements for which the *use of declarations* is appropriate, for example compliance with Annex III housing conditions; competence of staff involved in the project.

It is important to note that different approaches may be taken by Member States to ensure the competence of those involved in projects. For example, one approach is to require that all persons involved in the project be named in the application. Another approach is the use of a declaration by the person responsible for competence within the establishment. The former approach carries a greater administrative burden, especially if changes to authorisation have to be approved every time named individuals change over the life of the project (up to 5 years).

Regardless of which method is used, the project application should include a specific question requesting confirmation of the competence of all people involved in the project and how this will be assured.

Formulating specific questions

Purpose of the project should

- Set out the key scientific questions to be addressed;
- Include the purpose of the project as detailed in Article 5;
- Use SMART objectives – Specific; Measurable; Achievable; Realistic; Timely.

Objectives of the project

- Regulatory studies
    - For testing or screening projects: indicate the relevant statutory requirements or regulatory guidelines;
    - For service or production projects (such as production of blood products/antibodies; new GA lines): state the likely demands for the service or product in the lifetime of the authorisation.

- Basic research
    - The current state of knowledge on which project intends to build;
    - The way in which the project will help to advance knowledge.

- Goals already achieved by previous project(s) (where applicable) should be included: the progress summarised and an indication of which specific objectives should be achieved <u>through this project.</u>

- Scientific case, arguments/hypotheses should be presented concisely and <u>supported with references/literature review.</u>

    - List of up to 10 key references and/or regulatory guidelines supporting the need for the work and/or benefits set out above and relevant references for any specific models that are proposed;
    - Inclusion of key search sources and search dates.

- Applications for projects will normally require some information not required by grant applications; for example in project applications the proposed use of animals needs to be clearly explained and put into the context of the scientific programme.

<u>Predicted scientific benefits, educational value or law requiring the use of animals</u>

- **What** benefits, **who** is going to benefit, **how** and **when (**where possible) benefits may be realised.

- Acknowledgment that in some areas of basic research expanding knowledge can be a suitable objective in its own right – but <u>**should, where possible, be linked to dissemination**</u> of results (having regard to IP), and potential longer term benefits.

- When animals are used for the purposes of education and training, consideration should also be given to the type of trainees and the knowledge and skills likely to be required in their future careers.

<u>The programme of work should include at least the following information</u>

- Why it is not possible to achieve the objectives without the use of animals;

- How *ex-vivo/in vitro* work contributes with the *in vivo* work;

- The choice of model; why it is considered the most relevant and translatable;

- The links between the overall objectives and animals/models to be used - which and how each scientific study will contribute to the objective(s);

- Indication of the relationship between each component of the project and the sequence of the work.

Appendix I provides a collection of pre-formulated questions on the different elements, focusing specifically on Annex VI of the Directive and requests for exemptions, which should be addressed in the project application. These illustrative questions may be helpful for Competent Authorities when drafting (a) template(s) for use by project applicants.

Project evaluation process (who/how)

Principles for an effective project evaluation process

1. Availability of suitable scientific and technical expertise – including access to experts in less common areas of science and ensuring that all requirements in Article 38 are properly covered
2. Impartiality – lack of conflict of interest
3. Proportionality
4. Consistency
5. Efficiency
6. Transparency of the process
7. Access to an independent appeals process
8. Detailed understanding of the context of and criteria for PE, in particular harm – benefit analysis.
9. Sufficient resources
10. Knowledge of local culture and practices in establishment(s) where work is carried out

Models used in project evaluation process

National board - deals with all applications – includes a number of individuals with expertise in the use of animals for experimental purposes: scientific research (also alternative methods), scientific procedures, husbandry and care of animals, veterinary medicine, animal protection and ethics.

Pool of members allows anyone with conflicts of interest to stand down.

National assessment/Inspectorate – deals with all applications – flexibility to co-opt additional expertise as necessary. Individuals employed by Member States - no conflict of interest – and work in a department which neither commissions, nor carries out, research on animals.

Regional boards – deal with project applications from establishments within their region – similar composition to national boards.

Local review panels – similar to regional boards, but evaluate applications from establishments within a smaller geographical area.

Establishment review panel – have a role in contributing and improving the project applications by providing local input in the PE process.

How can these principles be addressed in practice?

A number of different approaches can be taken to meet the requirements of Article 38 as well as other elements considered essential for an effective project evaluation.

1. **Availability of suitable expertise**

Project evaluation requires a range of expertise, including an understanding of the areas of science under investigation and developments in the Three Rs related to these, experimental design, and animal health, care and welfare. Some areas under investigation are very specialist, with only a few experts having sufficient knowledge to assess the quality of the scientific argument for the use of animals and the likelihood of success. Access to a wide range of expertise is therefore necessary and often can more easily be attained within a national or regional structure than at a local level.

Contributions to the process should be balanced, considering the interests of both science and welfare and incorporating an independent view wherever possible.

2. **Impartiality – lack of conflict of interest**

Subject to safeguarding intellectual property and confidential information, PE shall be performed in an impartial manner and the Competent Authority carrying out the task should have no conflict of interest.

Removal of identification of the applicant may be helpful in preventing potential conflicts.

Within any PE system, all contributors should be encouraged to make conflict of interest declarations (and confidentiality), and the terms of references should explain how impartiality is assured.

The use of lay members[3] may be helpful in securing impartiality and lack of conflict of interest.

[3] lay member = a person who does not have specialised or professional knowledge of a subject

The choice of decision making method can also play a role in guaranteeing impartiality: simple majority vote - with recording of a minority opinion where appropriate - versus consensus decision.

The background and remuneration of those involved should be considered as it may influence impartiality.

3. Proportionality

The broader the scope and the greater number of applications evaluated will facilitate exercising some degree of proportionality. Conversely, processes assessing small number of applications are likely to find it difficult to develop a common proportionate approach.

Factors which may be considered when determining the degree of detail required include

- Type of project (regulatory / generic production / basic/ educational);
- Species (and numbers) of animals – level of sentience;
- Scale of project, complexity, novelty (e.g. new/pilot studies);
- Duration of project;
- Severity of procedures in the project;
- Previous experience (track record) of the applicant or establishment.

A risk based approach may be adopted. This is elaborated further in section "How to perform a harm-benefit analysis?"

4. Consistency

Consistency of decision making process is important to give confidence to applicants that their applications are being dealt with equitably. This is of particular importance for a level playing field within the EU scientific community where the authorisation for projects should follow a similar approach and give very similar outcomes.

Consistency is likely to improve as experience and the numbers of applications dealt with increase.

At a local level, it is very difficult to compare harm/benefit analysis for different types of application or areas of science, as often only a narrow field is covered by the applications dealt with. A national or regional oversight makes comparison easier and allows gaining experience and understanding and weighing of different types of harms and benefits.

Consistency can be improved by good common training programmes and exchange of information, personnel and practices among those involved in PE. The National Committee (Article 49) may also consider a role in promoting consistency in PE.

At a local level, there may be a "consistent approach and outcomes", but it is important that there are mechanisms in place to exchange and share information to promote common national standards, otherwise applicants will be left in a "geographical lottery".

Despite a common approach, it is unlikely that there will ever be a uniform outcome, due to different local, regional and national approaches to certain types of procedures and projects. However, the decisions and reasons should be justified and appropriately documented to clarify how outcomes have been reached.

5. Efficiency

The evaluation process should be designed and managed to ensure that the timescale for authorisation decisions set out in Article 41 can be achieved.

Ensuring a "complete and correct application" is very important to applicants and evaluators and can be facilitated by a well-structured application form and input from local advisors such as the designated veterinarian and the person responsible for overseeing welfare and care. Some systems actively encourage contact with the project evaluator (s) during preparation of the application, to ensure as far as possible that any necessary improvements and clarifications can be prepared prior to submission. Ideally, an efficient PE should be based on a two-way communication starting from when the project is first conceived.

6. Transparency of the process

The project evaluation process shall be transparent. All interested in the use of animals for scientific procedures should understand what is required of the process (how to make an application; what information is required), which authorities are involved in the PE process (structure of PE) and how the evaluation is carried out. This, in particular, helps project applicants to better understand the information requirements and how decisions are made. In the case of refusals following PE, it is helpful to provide the applicant with the reasoning for the decision.

To ensure the process of PE is properly understood, Member States may consider publishing national guidelines on the process, assessment criteria, including the application and assessment forms.

7. Access to appeals process

Following a PE, where an application is unsuccessful, there should be an appeal process available which allows the decision to be reconsidered using a process, independent of those involved in the initial PE.

8. Detailed understanding of the context of and criteria for PE, in particular harm –benefit analysis

Those involved in PE should understand the context, the principles and the criteria of project evaluation, be able to contribute to a consistent approach to project evaluation and to formulate and deliver well-informed, impartial and justified opinions.

Evaluators should have access to training in the process, in particular on how the objectives of the project, the application of the Three Rs and the assessment of severity classification should be evaluated, and on how the harm-benefit analysis should be undertaken.[4]

9. Sufficient Resources

Resources which would be helpful to evaluators, include – access to scientific search engines, in particular on the Three Rs, databases and -where deemed necessary- administrative support and access to additional expert pools.

10. Knowledge of local culture and practices

Local knowledge is important for the PE process. Such information may be included in the application. Assurance from the establishment should be provided indicating that suitable expertise and facilities are available, and providing information on local standards e.g. dosing / sampling regime; welfare assessment.

Consideration of different methodologies

No single methodology or structure is recommended, but there are strengths and weaknesses of each approach which need due consideration to ensure that the evaluation process is fair, robust, complies with the requirements of the Directive and is consistent within the EU.

It is important that the potential limitations of the different systems are acknowledged and measures taken to address these.

1. National systems / inspectorate

Strengths

- Impartiality/lack of conflict of interest;
- Consistency and proportionality due to high volumes of projects for evaluation;
- Available expertise - wide range of expertise available – care; veterinary; experimental design; scientific disciplines;
- Inspectorate – likely to have knowledge of local culture and practices.

Potential weaknesses

- National panels - need information on local issues e.g. experience/expertise on accommodation and care at the establishment where project will be carried out. The Animal Welfare Body (Article 26) could be helpful in providing local input in the process;
- Need for access to an independent appeal process.

[4] http://ec.europa.eu/environment/chemicals/lab_animals/pdf/guidance/education_training/en.pdf

2. Project Evaluation at regional / local level

Strengths

- Impartiality/lack of conflict of interest;
- Consistency and proportionality dependent on volumes;
- Local knowledge for local panels;
- Appeal process could be at national level.

Potential weaknesses

- Local knowledge more difficult to acquire the more distance from the individual establishment;
- Consistency and proportionality - less exposure to breadth of applications than found at national level and weakens towards local process;
- More limited access to wide range of expertise, compared with National level.

3. Project evaluation at establishment / institute

Strengths

- Local knowledge;
- Appeal process could be at regional or national level.

Potential weaknesses

- Impartiality/lack of conflict of interest; very difficult to meet these requirements, e.g. the importance of research programme to institute's reputation, future finances etc. could influence all those employed by the institute, including the evaluators;
- Lack of availability of necessary breadth of expertise;
- Consistency/proportionality – as projects are likely to be in relatively narrow areas of work it is very difficult to achieve consistency and common approach with a risk of high variation between institutes.

Assessment must be done on a case by case basis. As required by Article 59, in each case the Member State must have proof that the designated body (including which carries out the project evaluation) is free of any conflict of interests as regards the performance of that task. Without external, independent involvement, an institutional review process when done solely by the personnel employed by the applicant's establishment is highly unlikely to meet the requirements of Articles 38(4) and 59 of the Directive.

Additional comments on project evaluation process

A decision by consensus is considered as ideal. However, if no consensus can be reached, the panel may need to resort to simple majority voting. In such circumstances, the differing views should be recorded.

An external lay member is considered to be helpful, if confidentiality can be assured. The role of a lay person should be to ensure ethical and societal concerns are taken into consideration, but not necessarily for the implementation of Three Rs as the individual may lack the necessary technical knowledge.

Those involved in PE should be committed to promoting effective implementation of Three Rs and animal use in scientific procedures. This could be encouraged through formal application processes to identify suitable project evaluators.

Discussion amongst those involved in PE is essential – PE should generally not be carried out in isolation or solely by e-mail exchange.

Evaluation of the scientific justification for exemptions and application of the Three Rs

Evaluation of the scientific justification for exemptions

A number of articles in the Directive require that specific scientific justification is required to permit the use of, for example, certain types of animals or procedures.

In some circumstances, the justification is self-evident, for example, conducting scientific studies on an endangered species aimed at preservation of that species: the information is integrally linked to the scientific purpose and therefore considered in the overall harm-benefit assessment i.e. scientific objectives cannot be achieved without the use of a particular species.

Often, however, a clear separate scientific case is required to explain why specific animals/procedures are required.

For the purposes of PE, the applicant should have made clear why the animals/procedures are needed to meet the objectives of the project. This information should cite all relevant supporting evidence, which can be verified, as appropriate, during PE. Those involved in the process may have the necessary expertise to determine whether or not such exemptions are necessary and justified within the context of the project. If not, there will be a need to seek external advice on specific issues, beyond the expertise of the PEs.

There may be some national variations in the acceptance of certain exemptions, for example, the use of stray animals or animals in prolonged pain which cannot be alleviated. However, the PEs should be knowledgeable on the national legislation and can identify these issues should these arise in an application.

Specific exemption requests may prompt further enquiries – for example the use of an unusual exotic species should prompt enquiries on housing and care practices.

There should be an on-going challenge of "traditional" models or species i.e. has consideration been given to non-animal methods or approaches, the development of a different more refined model or the use of animals with a lower capacity to experience pain, suffering or distress.

Evaluation of the application of the Three Rs

The application must demonstrate consideration has been given to the Three Rs and that these have been applied wherever possible, consistent with the scientific objectives.

Evaluation of the correct application of the Three Rs can be facilitated by

- Checks on the material submitted in the application – review referenced literature; conduct search in relevant area;
- Ensuring necessary competence within PE process
  - Inclusion of expertise in the Three Rs relevant to the area of research;
  - Relevant up-to-date expertise on the Three Rs searches for project evaluators (web-sites, search engines[5]);
- Challenging applications where replacement methods are available but may not be being utilised;
- Clarity in demonstration that the selected model is the most appropriate and that adverse effects/severity have been minimised consistent with scientific objectives;
- Consideration for / use of systematic review by the applicant to determine the most appropriate approach;
- The timeliness of references – confirmation that most up to date references have been considered;
- Confidence in the competence and knowledge of local support (e.g. by Animal Welfare Body) in facilitating correct application of the Three Rs within establishment.

Evaluation of the Three Rs in projects for educational and training purposes

When PE is carried out for a project for educational or training purposes, a tiered approach using non–animal alternatives, cadaver work and finally live animals should be part of a systematic assessment to ensure implementation of the Three Rs:

[5] http://ec.europa.eu/environment/chemicals/lab_animals/3r/alternatives_information_en.htm

1. No animal use
    - Theory;
    - Demonstration of procedures/techniques (or physiological responses) by the use of e.g. pictures, videos, interactive audio-visual tools;
    - Observation of a competent person performing the procedure live as part of an existing study;
    - Practice of technical/practical skills on "simulators".
2. Use of cadavers
3. Use of live animals
    a. Non-recovery (anaesthetised) animals
        - Use of the animal for more than one technique is recommended since the harms for the animal are the same.

    b. Use of conscious animals
        - If the procedure will not influence experimental outcome, or significantly affect severity, training could be done on animals within an existing study;
        - Training should always begin with teaching of the appropriate handling techniques to the species in question.

Consideration should also be given to the provenance of the animals to be used e.g. surplus stock animals; animals from completed studies, which have not yet been euthanased.

Performing a harm-benefit analysis

Article 38 provides the basis for the elements which need to be considered in the project evaluation seeking to establish whether the use of animals is justified and that the procedures are carried out in the most humane and environmentally sensitive manner. The PE shall include

- *an evaluation of the* ***objectives*** *of the project, the predicted scientific* ***benefits*** *or* ***educational value;***
- *a* ***harm-benefit analysis*** *of the project, to assess whether the harm to the animals in terms of suffering, pain and distress is justified by the expected outcome taking into account* ***ethical considerations****, and may ultimately benefit human beings, animals or the environment.*

To facilitate the harm-benefit analysis, sufficient information must be included in the application, to enable the evaluators to make a reasoned judgement on the harms to animals and the benefits likely to accrue from the project and the likelihood of these being achieved.

The application form/template should invite these questions to be addressed.

Factors to take into consideration in assessing benefits

There should be an expectation and confirmation in the application that the project will contribute new knowledge, and that there is no unjustified duplication of animal use.

Identification of direct and indirect benefits

What will be the benefits of the work?

Who will benefit from the work?

How will they benefit – impact?

When (where possible) will the benefits be achieved?

Significance and impact of potential benefits

Assessment of projects with early applied benefits, such as a new vaccine to deliver improved health for humans, where the benefits can be easily recognised and may even be quantifiable, in terms of patients affected, lend themselves much more readily to a harm/benefit assessment than a project where advancement of knowledge in a particular scientific discipline is the primary benefit expected.

Although not always possible, some quantitative/qualitative estimate of the impact of the research would be helpful to evaluators – for example the number of persons/animals affected and the level of improvement which can be expected if project is successful.

The benefits should be linked to the purposes of the project set out in Article 5. Benefits may include:

- Basic research
    - Better understanding of the issue (increased knowledge – acknowledging the importance of filling a knowledge/information gap);
    - Wherever possible, "increased knowledge" as the primary benefit should be linked to a more tangible strategic goal, even though any wider benefits may be much further in the future and less predictable; benefits should go beyond "it would be nice to know";
    - Scale of improvement (man, animal, environment) (numbers; size and quality – need informed judgement – orphan drugs may be used in a few people but high impact on individuals) and burden to the society of the problem (both on basic/applied research);

    - Acknowledgement of basic research driven by hypotheses – evaluation needs to confirm hypothesis is scientifically sound and realistic;
    - Dissemination of information, whether positive or negative, is particularly important for basic research to ensure the benefits are realised.
- Safety assessment
    - Product safety (including the whole product cycle);
    - Food/feed safety.
- Improved human/animal health – provide if possible measures of improvement.

Other factors to consider in relation to predicted benefits

- Timeliness and relevance of work - may consider review of recent citations linked to area of work.

An acknowledgement is given that there may be differing priorities among Member States resulting in differing weights being allocated to benefits. Regional differences within a Member State may also influence PE considerations (e.g. type of environment, patterns of disease). A consistency review by National Committees may identify/explain these differences.

Can benefits be "weighed"?

Weighing of non-comparable, sometimes abstract benefits arising from different types of research programmes is very difficult to perform objectively.

There is an acknowledgment that without basic/fundamental research, many of the subsequent applied benefits would not have occurred.

It could be argued that for example the use of live animals in education may be of less importance than testing safety of medicines under a regulatory regime, or that human health should come before animal health. However, since there is no common agreement, it is not possible to place the benefits from the use of animals in research projects objectively in a simple hierarchical order to assist in the harm-benefit assessment of individual projects.

The "importance" of work is a subjective judgement changing with time and place depending on a number of variables such as culture, environment, economic situation, acquired knowledge, emerging unsolved scientific problems and ethical values.

This in turn further emphasises **the need for a unique, case-by-case evaluation** of the importance and magnitude of benefits **for each proposed project**.

Key considerations in the assessment of benefits

1. Consider the immediate / short term benefits (product; data; outcome).
2. Consider longer term benefits (product; specific long term).
3. Consider the wider impact (increase of knowledge; translational potential).

Factors to take into consideration in assessing harms

The EU EWG Reports on Severity Classification and Retrospective Reporting (available on EC website http://ec.europa.eu/environment/chemicals/lab_animals/pubs_guidance_en.htm) include guidance on the assessment and classification of severity and a number of illustrative examples are also available.

The assessment of harms during PE has to consider the impact on all animals planned for use in the project – prospective severity classification of procedures is based on the highest severity anticipated for a single animal – however, this effect may only be expected in 1/100 animals or could be expected 90/100 animals, significantly affecting the overall welfare "costs". It is important therefore to know what the predicted severity is for *all animals* used on the procedure, taking into account the methods used to minimise adverse effects.

The key issues which need to be included in consideration of harms include:

- Procedures being applied to animals;
  - Frequency/duration of procedures;
  - Likelihood of adverse effects;
  - Severity level and methodology to minimise severity;
  - Monitoring regime; welfare assessment protocols;
  - Humane end-points and triggers for interventions;
- Species/strain/age of animals being used;
- Number of animals;
- Fate of animals;
  - Death – intrinsic value of animal ; “quality” of death impacts on animal’s experience and on severity;
  - Criteria for re-use or rehoming.
- Contingent harms – husbandry and care practices; transportation.

When using animals for the purposes of education and training, the severity of procedures should be restricted to “non–recovery” or “mild”. However, it is recognised that rare, well justified exceptions could be accepted to this general principle.

Examples of such exceptions include

- a surgeon training to undertake a particular procedure where it could be important to evaluate the success during the recovery phase;

- preparation of educational material to replace future live animal use.

Consideration should also be given to the cumulative effects of techniques considered to be 'lower than or 'below threshold' which when used in combination or on more than one occasion (multiple) may lead to a 'higher' or above 'minimum threshold' severity when repeated.

The importance and availability of competent supervision cannot be over-emphasised. These aspects should be specifically addressed and evaluated in proposals for projects using live animals for educational and training purposes.

Factors to take into consideration in assessing likelihood of success

Many factors can influence the potential of a project to achieve the predicted benefits and these need to be given due consideration in the analysis of the Project. These include:

- realistic objectives (SMART - Specific; Measurable; Achievable; Realistic; Timely);
- scientific soundness;
- deliverable in the time frame outlined;
- adequately resourced (financial, appropriate facilities, personnel – scientific and care staff);
- experience/track record in field and in specific area of planned work;
- publications; citations;
- clearly defined plan of work – choice of methods/design/species/animal model;
- publication plan;
- feedback from retrospective assessment of previous projects from the applicant/research group should increasingly facilitate determination of likelihood of success.

Evaluation of the project application

To be able to evaluate a project effectively, the evaluators need to be confident that the information provided is *complete, correct, current and relevant*.

Prior to carrying out a harm-benefit analysis, the project evaluators need to

1. be assured that all Three R opportunities have been given due consideration and implemented to the maximum extent possible;
2. confirm that the proposed exemptions are scientifically justified;
3. have an understanding of the potential benefits, their nature and timing;

4. confirm the severities and quantify the harms;
5. have a view as to the likelihood of achieving the benefits claimed.

Harm – Benefit analysis

The weighing of harms against benefits is not a simple decision-making process and requires carefully consideration. For well-informed judgements to be made all relevant information must be available to those undertaking the PE.

The process should be well-balanced, robust and challenging:

- should not automatically assume that claims of potential scientific benefit are always correct;
- should understand all the potential harms to the animals;
- should be prepared to challenge the status quo and to reject poorly designed and ill thought through projects and
- be prepared to challenge cultural/social/political issues e.g. outdated methodologies or views that animals do not need pain relief.

How to weigh harms and benefits

There is more guidance (and experience) in assessing and "weighing" harms to animals than there is available for attributing significance to benefits. In particular, it can be very difficult to assign a value to projects aimed at generating fundamental knowledge.

As experience evolves under the new Directive, a review in a few years' time of how PEs assign harms and benefits may be worthwhile.

Project evaluation requires consideration of three separate but integrally linked components – the predicted benefits, the harms to the animals and the likelihood of the applicant achieving these benefits.

General comments: benefits

- Higher "benefits" are accorded to human health application and to seriousness of the condition and numbers affected;
- Presence of existing drugs for the disease in question may militate against a high welfare cost application;
- Magnitude of improvement in human population/environment (quality/quantity);
- The "values" of benefits are dynamic and influenced by politics; social factors; health and economic issues. These will move with time and there will be variations among Member States;
- The benefits of regulatory testing can be difficult to determine beyond that of safety and efficacy, but there are legal requirements that these be conducted – however in all circumstances, the expectation is that the Three Rs are fully implemented.

General comments: harms

- Changes in public perceptions impact on the acceptability of certain types of procedures;
- Need examples of "No go"-areas; e.g. the use of animals in severe procedures to investigate trivia issues of little significance;
- Need to consider, in addition to scientific justification, any potential additional related harms due to the use of endangered species, strays, NHPs, re-use, continued use, methods of killing etc. and how these are addressed.

General comments: likelihood of achievement

- The appropriateness of animal models and, for example, the extrapolation of results to the human condition is realistic;
- Clarity, reliability and robust arguments by the applicant;
- Trust and confidence in culture at establishment where work will be conducted;
- Economic factors need due consideration – for example moving from a rodent model to zebra fish may be considered a refinement – rodent research which remains valid should not need to stop, while new infrastructure/finances are put in place – still requires a confirmation that the research is well justified.

How to perform a harm-benefit analysis

All relevant information needs to be available to project evaluators, and a step-wise approach should be taken to ensure that all the necessary justifications and explanations have been included.

This analysis also provides an opportunity to confirm that the benefits have been clearly explained and optimised and the harms reduced to the minimum consistent with the objectives.

A number of "models" have been used to inform the process, for example Voipio et al 2005, but there is no formula which can be applied to replace a fully considered analysis of the different elements by experienced evaluators.

The Bateson cube (1986) is used by some as a simple matrix to assist the process. This algorithm suggests that the level of suffering, should be linked to the importance of research (potential benefits) and the likelihood of the benefit being achieved. Where high welfare cost is linked to low importance research with low likelihood of any benefit, the use of animals for such work should not be permitted. However, for wider application, consideration of basic research (e.g. advancement of knowledge as a primary benefit) would need to be incorporated in the terminology.

Following discussion at the meeting, a revised “Cube” was developed (see Appendix II) using colours to indicate which applications would require most scrutiny with regard to a harm/benefit analysis. The colours reflect a relationship between the level of harms, degree of benefits and likelihood of success. Where the intersection of the variables is shaded green it is likely that a favourable harm-benefit analysis will result from the project evaluation. In contrast, it is likely that much more detailed considerations will be needed for the amber and red shaded cubes.

The Bateson Cube concept fits with many of the frameworks/formulae developed to assist in the evaluation of research proposals.

$$\text{Justification} = \frac{\text{Importance of objectives} \times \text{Probability of achievement}}{\text{Harms to animals}}$$

A thorough understanding of these three areas is essential to enable an informed decision to be reached.

In considering the harms, there is the expectation that the Three Rs have already been fully implemented, and the harms already minimised consistent with the scientific objectives. An informed judgement is required of the benefits and likelihood of success, giving due consideration to ethical and societal need.

The evaluation process is multi-factorial and no simple numerical allocation formula can provide a simple yes/no-answer. Knowledge of the different published models of harm/benefit analysis is needed. These systems can be useful tools for discussion to ensure all issues are given structure and systematic consideration but these should not be used in isolation to replace intelligent interpretation of the information provided.

A check list would be helpful to ensure all issues are addressed and could form a framework for gathering common data for evaluation.

Subjectivity has to be recognised as an unavoidable component of the analysis – necessitating a balanced composition of competent assessors and a structured approach to the evaluation to ensure consistency in the analysis.

An informed discussion among well-trained evaluators with all relevant expertise available is most likely to give robust, reliable and consistent outcomes.

Retrospective assessment

Article 39 of the Directive describes the requirements for Retrospective Assessment (RA). RA is not mandatory for all projects and it is left to Member States to determine the need for RAs beyond those required by the Directive (all projects using non-human primates and those containing procedures classified as severe).

Retrospective assessment is considered an extremely powerful tool to facilitate critical review of the use of animals in scientific procedures, to identify future Three R improvements and, if published, to inform future studies and to enhance transparency to public.

The benefits of carrying out retrospective assessment

- Opportunity to review the outcome of the project against the objectives set, and, where applicable, the reasons where these were not achieved;
- Comparison of the actual versus predicted harms;
- Comparison of actual numbers of animals used versus estimates and consideration of reasons for variations;
- Opportunities to identify future refinement possibilities;
- Opportunity, should something go wrong during the study, to analyse reasons thereof and learn from these;
- Allows for Competent Authority to review the effectiveness of PE / harm-benefit analysis providing a tool for quality assurance, and improving consistency;
- Increased transparency and accountability especially when the results are published;
- Dissemination of outcomes regardless of the results[6] as it *inter alia*
    - Facilitates improved design for similar studies;
    - Raises awareness of appropriate use and best practice;
    - Raises awareness of inappropriate animal use, thus contributing to Reduction;
    - Prevents others from repeating problems/mistakes;
    - Assists Competent Authorities to review effectiveness of PE / harm-benefit analysis;

Factors to determine whether or not a retrospective assessment should be carried out

RAs are required for all projects involving non-human primates and all those containing procedures classified as severe. The Competent Authority can require RA to be carried out on

[6] Publication of RA results of so called "negative results" (studies for which the original hypotheses was not proven) is equally important as it increases the knowledge base, reduces risks of unjustified duplication of similar studies and guides future research.

other projects. It is the task of the PE to decide if a RA should be carried out and when. The factors which should be considered in such a determination should include the following:

- Introduction of new models or new research areas;
- Significant uncertainties in outcomes or effects on animals e.g. creation and breeding of certain GA lines;
- In the regulatory context, use of new classes of compounds, with little background data, knowledge or experience;
- Projects for the purposes of education and training ;
- In projects where severities are higher than predicted – this would generally necessitate an amendment to the project authorisation to allow the work to continue. Such projects may be marked for RA (if not already identified in initial authorisation process).

The most appropriate time to carry out a retrospective assessment

- Project evaluators will determine the most appropriate time for RA on a case-by- case basis. Ideally, to assess if the objectives have been met, the harms and elements to contribute to the Three Rs identified, it would seem reasonable to have RA carried out as soon as practicable following completion of the project. It is worth noting that benefits in some cases may not be realised until sometime after the project has been completed e.g. basic research to increase knowledge.

- If new models are being introduced, or there are significant unknowns with regard to severity or effects on the animal, pilot studies are often authorised. There should be a RA undertaken on completion of such studies to ensure adequate consideration is given to the results, and further changes/ measures introduced before more extensive studies are progressed.

Securing the necessary information for a retrospective assessment

In some cases, the most appropriate time to carry out a RA may be a considerable time after completion of the project, it is therefore essential that due consideration is placed on ensuring mechanisms are in place for securing the necessary information for the RA.

- Although the primary responsibility remains with the user (Art. 39(1) and 40(2)(a)), there should be some responsibility on the person responsible for the overall implementation of the project (Art. 40(2)(b)) to ensure delivery of the required information.

- There should be the option of having some flexibility within the process, for example there may be merit in encouraging on-going/periodic feedback (for example (publications, refinement opportunities) especially in cases of longer-running projects (which could be up to five years).

- Proportionality - using similar criteria as those identified for PE.

- There are benefits from providing local input, where all those involved are available, there is access to all relevant information, and timely introductions of improved practices/refinements can be progressed.

- To be effective there needs to be input from all the relevant people - those involved in the project and those with animal care and welfare responsibilities.

- A template to invite the correct information is considered helpful – some questions have been identified (see below). Such a template would assist in the preparation of the material for RA and also for those persons reviewing the material. However, there may be a need on occasion for additional specific questions tailored to particular projects.

- There should be feedback to the researcher in order that improvements/changes can be introduced to future studies.

Guidance on information requirements

Information is required on whether the objectives of the project were achieved; the harms inflicted on animals, including the numbers and species of animals used, and the severity of the procedures; and any elements that may contribute to the further implementation of the requirement of replacement, reduction and refinement.

Section 1 - Achievements

- Have the objectives of the project been achieved?
  - *Explain, briefly, whether, and to what extent, the objectives set out in your application have been achieved.*
  - *Have there been any other significant findings?*
  - *Provide reasons if objectives have not been attained.*
  - *What benefits have been accrued from the work to date, and are further benefits expected?*

Section 2 - Animal use and severity

- State animal numbers and species used together with actual severity experienced.
- How do these compare with those estimated in the application? Where these differ, please provide an explanation.

Section 3 - Implementation of the 3Rs

i. <u>Replacement</u>

- Have there been any developments in your scientific field (including the development/validation of new *in vitro* or *in silico* techniques) which would replace some or all of the use of animals?

ii. Reduction

- Has the project/experimental design been revisited to enable any further reduction in predicted animal use?
- Were the models used still the most appropriate?
- Were the numbers of animals used appropriate for statistical analysis (too many/too few)? Could different approaches reduce further animal use?

iii. Refinement

- List any refinements you introduced during the project to reduce harm to the animals.
- Could harms be further reduced?
- Could the procedures (for example administration/sampling routes; surgery) be further refined?
- Could animal monitoring regimes be improved?
- Were score sheets/welfare assessment protocols working well?
- Could humane end-points be refined?
- Could euthanasia methods be refined?

Appendix III contains further guidance on issues to be taken into account in RA.

Outcomes to derive from a retrospective assessment

1. Feedback to research group

Reviewer(s) should provide feedback to the researcher on issues raised by the assessment process. This may include suggestions for future improvements and recommendations to disseminate key information.

2. Dissemination of information on the use of animals and the Three Rs (both positives and negatives)

- Within establishment;
- Promotion of publications, presentations by researcher/user;
- Role of the National Committee under Article 49 in sharing of best practice in dissemination;
- Identification, collation and publication of key issues arising from RAs.

3. Updated information on non-technical project summaries

This will provide greater transparency on the actual harms and benefits related to the use of animals in scientific procedures. Impact may be improved by having the non-technical project summary updated once RA has been carried out.

4. Information can be used in review of effectiveness of project evaluation and in training of project evaluators and persons conducting RAs.

General recommendations

- A check-list for PE and RA should be developed to ensure all issues are addressed.
- Examples of PE and RA processes would be helpful both to applicants and evaluators.
- Promoting consistency- to promote and review consistency in PE and RA there should be
    - Regular review of evaluations and assessments;
    - Sharing evaluations among those responsible for PE/RA at national and EU level,
- Specific training where needed would be beneficial for persons involved in PE and RA, including for lay contributors[7].
- National Committees for the protection of animals used for scientific purposes under Article 49 of the Directive should promote consistency of approach to PE and to provide reassurance to the scientific community (level playing field).
- National Committees, together with the Member State Competent Authorities, should share best practice to promote dissemination of outcomes from RA processes.
- Listing of reference documents and tools should be developed for harm-benefit analysis.

By providing the necessary information in a suitable format, ensuring proper expertise, with as necessary, appropriate training for evaluators, and a suitable breadth of knowledge and experience into the processes of PE and RA, consistency can be promoted and common outcomes achieved.

The essential outcomes of these processes are to ensure that scientific procedures on animals are only performed when properly justified, when no alternatives are available, the minimum numbers of animals are used, and the procedures cause the least pain, suffering, distress or lasting harm consistent with the scientific needs, taking into account ethical considerations, and that there is a system of review to ensure a continued focus on the Three R improvements.

[7] http://ec.europa.eu/environment/chemicals/lab_animals/pdf/guidance/education_training/en.pdf

Bibliography

Animal Procedures Committee, Home Office, UK – Review of the Cost-Benefit Assessment in the Use of Animals in Research 2003.

Bateson, P. When to experiment on animals. New Scientist, 109 (1986), 30–32

Cuthill, I.C. Ethical regulation and animal science: why animal behaviour is not so special. Animal Behaviour. Volume 74, Issue 1, July 2007, 15–22

LASA (2004) Guidance Notes on Retrospective Review. A discussion document prepared by the LASA Ethics and Training Group (M Jennings and B Howard eds). Available for download from www.lasa.co.uk/position_papers/publications.asp

Lindl,T., Gross,U, Ruhdel, I., von Aulock, S., Volkel, M. Guidance on Determining Indispensability and Balancing Potential Benefits of Animal Experiments with Costs to the Animals with Specific Consideration of EU Directive 2010/63/EU, ALTEX 292/12 219-228

Smith, J.A. (Convenor, UK), van den Broek, F.A.R. (The Netherlands), Martorell, J., C., (Spain), Hackbarth, H. (Germany), Ruksenas, O., (Lithuania) and Zeller, W. (Switzerland). Principles and practice in ethical review of animal experiments across Europe: summary of the report of a FELASA working group on ethical evaluation of animal experiments. FELASA Working Group on Ethical Evaluation of Animal Experiments: FELASA, 25 Shaftesbury Avenue, London W1D 7EG, United Kingdom : Laboratory Animals (2007) 41 143-160

Voipio, H-M., Hirsjarvi, P., Ritskes-Hoitinga,M., Nevalainen, T. Nordic Forum for Ethical Evaluation of Animal Proceedings p60-62 In Proceedings of 9th FELASA Symposium, Nantes, France 2004

Appendix I

Pre-formulated questions for building a project application template

The example questions below were developed to give guidance to Member States in the development of project application templates. The list is not exhaustive and it remains the responsibility of the competent authority to determine how the necessary information is obtained.

Requirements of Annex VI

1. Relevance and justification of the following:

(a) *Use of animals including their origin, estimated numbers, species and life stages*

- Include origin e.g. authorised breeder within EU;
- Include strain, especially types of GA, where applicable;
- Explain the estimated scale of numbers of animals if exact numbers are not possible (e.g. development of a new GA line);
- Explain the scientific relevance of the proposed models.

(b) *Procedures*

- What is being done to the animals? (in sufficient detail to enable harms to be assessed)

2. Application of the Three Rs

Replacement

- Why is it not possible to achieve the objectives of your project without using animals?
- What alternatives have you considered and why are they not suitable?
- What alternatives will be used in achieving your objectives?
- Does an alternative method (without use of animals) with the same reliability exist for this procedure?
    - If yes, justify why this alternative method is not used:
    - If no, which sources did you consult to track possible alternatives? State the date of consultation (examples below).

Regulatory tests:

- Recent list of alternatives, adopted by OECD/OCDE (http://www.oecd.org);
- Recent list of alternatives, validated by EURL ECVAM (http://ecvam-dbalm.jrc.ec.europa.eu/);

- Recent list of alternatives adopted by European Pharmacopoeia;
- Others.

Research:

- Databases and/or publications of ECVAM or FRAME on in vitro methods;
- EURL ECVAM databases (http://ihcp.jrc.ec.europa.eu/our_labs/eurl-ecvam/databases);
- Others: Go3Rs (searches pub med) (http://www.go3r.org/).

Education:

- Norina (Database of Alternatives to Laboratory Animals): (http://oslovet.veths.no/norina/);
- Eurca: (http://www.eurca.org);
- NCA (Netherlands Centre Alternatives to Animal Use): (http://www.nkca.nl/algemeen/menu/english/) (under "links");
- Interniche (From guinea pig to computer mouse, Alternative methods for a progressive, humane education, N. Jukes et M. Chiuia): (http://www.interniche.org/en/resources/book);
- Others.

Reduction

- What measures have been or will be taken to ensure that the minimum number of animals will be used in this project?

- Explain the principles of experimental design you will use and any sources of advice you will consult e.g. on statistics.

- Has a collaboration with another laboratory (internal or external) been considered to reduce the number of animals used (joint use of animals) (e.g. different organs of the same animal are used in more than one laboratory)?

- Justify the number of animals to be used by appropriate statistical analysis?
  - If yes, give reference to this analysis.
  - If no, justify why this analysis has not been made.

Refinement

- Explain your choice of species, model(s) and method(s). Explain why they are the most refined for the intended purpose.
- How will you minimise animal suffering in order to achieve your objectives?
- Provide specific justification for any substantial severity procedures.

3. The planned use of anaesthesia, analgesia and other pain relieving methods.

- If anaesthesia is not being used, explain why.

- If analgesia is not being used, explain why.

- Explain how you will ensure that the most appropriate regimes are used.

  N.B. Differing approaches used: declaration regarding advice/input/oversight from designated veterinarian versus detailed description of regimes, including agents, routes volumes. Designated veterinarian (and/or AWB) should confirm appropriate anaesthetic and analgesic advice has been provided and applicant / compliance person should confirm this advice will be taken.

- If no precise information is stated, how will you ensure the most up-to-date anaesthesia/analgesia/dosing/route is being used appropriate to individual animals in the project? Who is being consulted?

  N.B. It was considered that if all agents, routes and dosages were to be provided – this would be inflexible and likely to increase numbers of project amendments.

- Have you considered other methods of housing and care as means to reduce pain, suffering or distress e.g. soft bedding and food provision on cage floor in arthritis studies?

4. Reduction, avoidance and alleviation of any form of animal suffering, from birth to death where appropriate.

It is important to minimise suffering, consistent with specific scientific objectives, not just use "standard" end-points – tailor to meet specific requirements.

- List the likely adverse effects of each of the regulated procedures being applied. Indicate how you will manage these effects to minimise severity. There is no need to detail uncommon or unlikely adverse effects or effects from procedures that cause no more than transient discomfort and no lasting harm, for example intravenous injection.

  For each adverse effect indicate:

    - the likely incidence;
    - how the adverse effect will be recognised;
    - the measures you will take to prevent or control occurrence and severity;
    - practicable and realistic humane end-points.

5. Use of humane end-points.

- Define clearly the envisaged humane end-points.
- Will pilot studies be used to define end-points in main studies?
- If death is to be an endpoint, explain why it is essential and what measures are in place to minimise the impact on the animals.

  N.B standardised establishment guidelines can be helpful for similar types of studies.

6. Experimental or observational strategy and statistical design to minimise animal numbers, pain, suffering, distress and environmental impact where appropriate.

- Provide an outline of the stages of the programme of work and indicate clearly, by using the protocol (procedure) numbers, how each protocol will be used to achieve your objectives.
- Where it would aid clarity, illustrate the steps of the programme using an annotated flow diagram or process map. This should include use of pilot studies and decision points.
- Describe previous experience with proposed models.
- Are pilot studies being used? Why are they necessary? For example, to identify/refine humane end-points.
- How will the animals be monitored? Describe the welfare assessment scheme that you will use.

7. Re-use of animals and its cumulative effect on the animal.

- Will any animal be re-used?
  - If yes – what is the justification; explain limitations, how the decisions will be made and the proposed severities in the new procedures.

8. The proposed severity classification of procedures.

- Explain how the proposed severities have been derived.[8]

9. Avoidance of unjustified duplication of procedures where appropriate.

- Has this animal experiment already been carried out before?

[8] See consensus document on Severity Assessment Framework at http://ec.europa.eu/environment/chemicals/lab_animals/pdf/guidance/severity/en.pdf

- If yes, justify why the work is to be repeated.
- If no, which information databases/search tools did you consult to check whether the animal experiment has not already been carried out before (list a minimum of X and provide the search date).

N.B. Competent Authorities may have information available which is not available to applicants – it should be considered how this information can be used by the Competent Authority to avoid duplication or even disseminated if confidentiality/IP issues can be avoided, e.g. one contract research organisation conducting studies on similar compound. Better exchange / dissemination of information could reduce animal numbers.

10. Housing, husbandry and care conditions for the animals.

- How do you ensure your animals have suitable quality of life from birth to death?
- How have you considered e.g. transport: international, national as well as local (within establishment); the suitability and reliability of source (breeder/supplier/ user/other).
- Describe the social and environmental enrichment programme.
- Describe and justify any lowering of the minimum standards in Annex III e.g. single housing. Explain the expected effects on animals and how these will be mitigated.

11. Fate of the animals.

- Could animals be kept alive after the study and re-used, released or rehomed?
- If not, state the method of killing. If not one of the methods listed in Annex IV explain and justify why another method is needed. Identify any additional welfare costs associated with these methods and measures taken to minimise these. Include sources consulted for ensuring the most refined method.

12. Competence of persons involved in the project.

- Confirm the competence of all people involved in the project and how this will be assured.

Use of live animals for the purposes of education and training

- Describe the learning objective(s) of each of the procedures and how the proposed procedure(s) will meet these.
- Describe the particular trainee (group) which requires this training.

- What is the purpose of and need for the procedure(s)?
- Will the procedure(s) be used solely as a demonstration, for the making of a video-recording or for the provision of tissues etc., or will there be participation by students?
- Why it is essential to use (an) *in vivo* model(s) for the procedure(s)?
    - Confirmation should be provided that a thorough search for suitable alternative methods has been made.
    - The range of alternative teaching methods available should be explored (particularly experiments on human volunteers, video- and computer-based learning methods, and in vitro and ex vivo studies).
    - If alternative methods are not used, provide justification as to why these are unsuitable.
    - If alternative methods are unavailable or unsuitable, has consideration been given to the production of suitable material (e.g. video recordings) for future use in teaching?
- How and which alternative approaches are used prior to *in vivo* work?
- Explain why the learning objective cannot be fulfilled by observation of on-going research?
- Provide a specific justification for procedure(s) with a severity greater than "mild" and reasoning why the severity classification is the lowest which can achieve the learning outcomes.
- What feedback will be sought from the students on whether the educational objectives have been attained?

Request for an exemption

- Provide *scientific and/or other justification* for the use of exemptions for
    - methods of killing not included in Annex IV;
    - endangered species (Article 7);
    - non-human primates (Article 8);
    - animals taken from the wild (Article 9);
    - non-purpose bred animals (Article 10 and Annex I);
    - stray or feral animals (Article 11);
    - work outside a user establishment (Article 12);
    - re-use taking into account the cumulative severity (Annex VIII) and lifetime experience (Article 16);
    - care and accommodation practices failing to meet standards in Article 33.

- For all questions: provide literature support and references if relevant.

Appendix II

Modified Bateson Cube

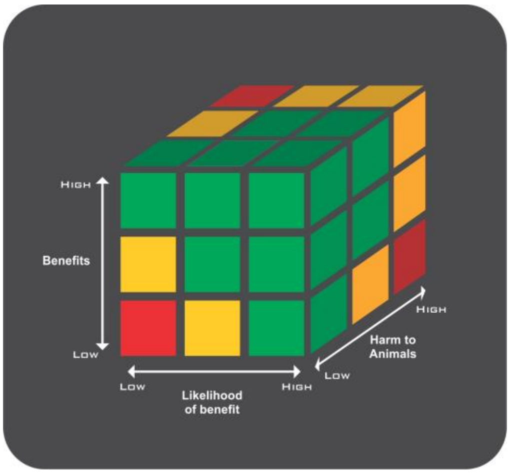

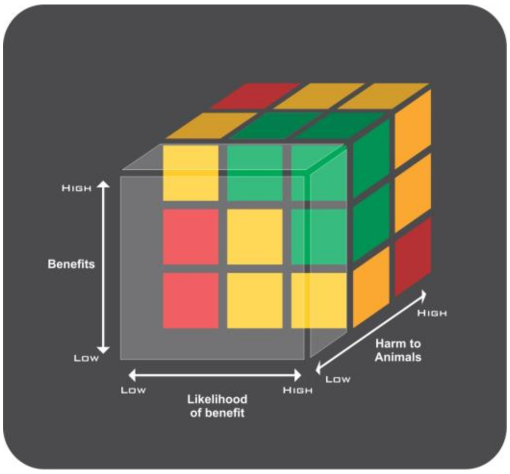

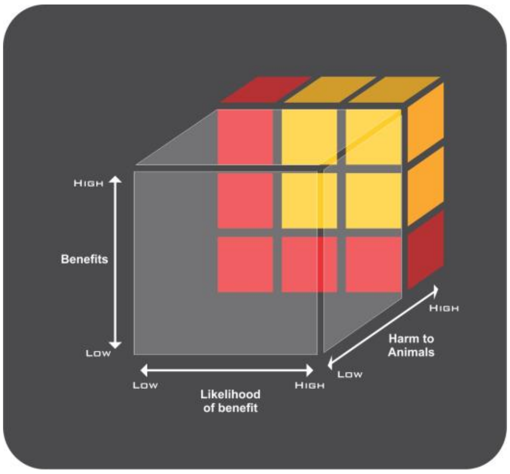

Appendix III

Further guidance on issues to consider in retrospective assessment

http://www.rspca.org.uk/ImageLocator/LocateAsset?asset=document&assetId=1232712119425&mode=prd

(see following page)

The Value of Looking Back:
Improving Science and Welfare through Retrospective Review

Maggy Jennings [1], Bryan Howard [2] & Manuel Berdoy [3]

[1] Royal Society for the Prevention of Cruelty to Animals, UK, [2] Laboratory Animal Science Association, UK, [3] Oxford University, UK.

SUMMARY

Several European countries now require retrospective review of research projects. This provides a clear time point at which to review the scientific progress in relation to the actual versus predicted harm/benefit assessment, to consider further implementation of the Three Rs and to facilitate project management. Retrospective Review can improve welfare, science, ethics and project management *when it is done well* but its value is heavily dependant upon how it is carried out.

The following presents the conclusions and recommendations of two workshops organised by the Ethics, Training and Education Section of the UK Laboratory Animal Science Association (LASA).

A) It identifies the key benefits and objectives of retrospective review and provides a set of '25 points to consider' within the review.

B) It provides some guidance on how the efficiency and effectiveness of the process can be optimised.

The general principles are relevant to any arrangement for reviewing animal work, including reviews carried out by funding or grant-awarding bodies.

A THE KEY OBJECTIVES OF THE RETROSPECTIVE REVIEW & 25 POINTS TO HELP ENSURE THAT THEY ARE MET

Key Objective 1:

To determine whether the *actual* harms & benefits are in line with those anticipated.

i.e. the <u>current state</u> of the harm/benefit ratio:

1. Are the adverse effects and severity in line with what was predicted?
2. Is the science on track? Are the results as expected? Are there successes to be recognised (including unexpected ones)?
3. Is the animal model still the most appropriate for this type of study?
4. Are there any recent developments in science or technology which should influence the direction or conduct of the study?

Key Objective 2:

To identify, build on and encourage implementation and improvements in the 3Rs during the course of a project.

i.e. the <u>technical aspects</u> of easing the harm/benefit ratio:

5. Are there alternative methods/models (including new *in vitro* techniques) available that would involve less suffering?
6. Can the experimental design be improved to answer the hypothesis more effectively?
7. Are the numbers of animals used statistically appropriate (not enough/too many) in the light of the results to date?
8. Could procedures (*e.g.* surgery, administration, sampling) be further refined?
9. Could monitoring regimes be improved? Are score sheets working well? Can humane endpoints be refined?
10. Can any negative effects to animals associated with supply and transport, or housing and care, be reduced and welfare improved?
11. How are animals on long term studies coping? Are there any physical or behavioural problems?
12. Have special housing and care needs arisen?
13. Can euthanasia be refined?
14. Is there any animal wastage and can this be avoided?
15. Is there opportunity for re-homing and is this in the best interests of the animals?

Key Objective 3:

To facilitate project management.

i.e. the <u>managerial aspects</u> of easing the cost/benefit ratio:

16. Are any amendments likely to be needed in the near future, perhaps due to unexpected costs or unexpected discoveries as highlighted in key objective 1?
17. Is the programme of work appropriately flexible?
18. Are the facilities (still) appropriate? Is there anything that the researcher should be made aware of (*e.g.* refurbishment, equipment supplies)?
19. Are there an human resource issues (e.g. staff shortages)?
20. Is communication within and/or between research team(s) appropriate?
21. Has a training need been identified?
22. Do the Animal Care staff or the Veterinary Surgeons have any general concerns?
23. Are their roles well supported by the establishment?
24. Has/can the information on 3Rs be disseminated within and/or between institutions ?
25. Are commendations possible within the establishment?

B The BEST PROCESSES lead to the BEST OUTPUTS

There is no clear harmonised guidance on how retrospective review should be done. A 2005 FELASA survey shows a variety of approaches, including ongoing or annual reviews, or at completion of the project. The focus should be on achieving a successful output rather than on developing overly bureaucratic processes. The following factors are key to its success.

Key factor 1:
Make it a positive & constructive experience

- Staff need to see how it benefits them, their science and animal welfare
- The process and objectives should be clear
- Include information in local training courses
- Be inclusive of all relevant staff
- Focus on discussion and outputs not filling in forms
- Provide - and explain – feedback. If there are concerns (about the project or the process), do something about it !

Key factor 2:
Create a workable process - there is no one rule for all!

- Be flexible with timing - think about this at the outset of a project
- Prioritise projects for review *e.g.* those using large numbers of animals, severe procedures, new models, certain species
- Keep documentation to a minimum - be clear about input, and how this should be provided
- Be clear who is involved - it may not need a whole committee
- Focus on the outputs and how to take things forward

Key factor 3:
Ensure that it is properly resourced

- Make it an integral part of project management & team meetings
- Combine with other activities (*e.g.* review by grant-awarding body, preparing papers or presentations for publication, submission of amendments)
- Involve senior management so they see the value

Further Information

LASA (2004) has defined a list of key objectives and ideas for effective operation, which are already in use in the UK. LASA is now developing further resources. The current & updated report will be on the LASA website www.lasa.co.uk/position_papers/publications.asp

An electronic copy of this page can be obtained by writing to:
training@vet.ox.ac.uk

References

FELASA (2005) Principles and practice in ethical review of animal experiments across Europe. A report prepared by the Working Group on Ethical Evaluation of Animal Experiments. www.felasa.eu/recommendations.htm

LASA (2004). Guidance Notes on Retrospective Review. A Discussion Document Prepared by the LASA Ethics and Training Group (M. Jennings and B. Howard eds). Available for download from www.lasa.co.uk/publications.html
