# Supplementary material for: Mission impossible accomplished? A European cross-national comparative study on the integration of the harm-benefit analysis into law and policy documents
Source: PLoS One. 2024 Feb 20;19(2):e0297375. doi: 10.1371/journal.pone.0297375 (PMC10878508; doi:10.1371/journal.pone.0297375)
Supplement: S2 File — (PDF) [file pone.0297375.s002.pdf]

Kurztitel

Tierversuchs-Kriterienkatalog-Verordnung

Kundmachungsorgan

BGBl. II Nr. 460/2015 zuletzt geändert durch BGBl. II Nr. 542/2020

Typ

V

§/Artikel/Anlage

Anl. 1

Inkrafttretensdatum

05.12.2020

Abkürzung

TVKKV

Index

86/01 Veterinärrecht allgemein

Text

Anlage

Kriterienkatalog

1. Allgemeine Angaben zum Projekt

Lfd. Nr.	Feldtitel bzw. -beschreibung
1.1	**Projekttitel**:
1.2	**Zweck(e)** des Projekts (Mehrfachauswahl möglich):
☐	Grundlagenforschung
☐	Translationale und angewandte Forschung zur Verhütung, Vorbeugung, Diagnose oder Behandlung von Krankheiten oder anderen Anomalien oder deren Folgen bei Menschen, Tieren oder Pflanzen
☐	Translationale und angewandte Forschung zur Beurteilung, Erkennung, Regulierung oder Veränderung physiologischer Zustände bei Menschen, Tieren oder Pflanzen
☐	Translationale und angewandte Forschung zur Verbesserung des Wohlergehens der Tiere und Verbesserung der Produktionsbedingungen für die zu landwirtschaftlichen Zwecken aufgezogenen Tiere
☐	Entwicklung, Herstellung sowie Qualitäts-, Wirksamkeits- und Unbedenklichkeitsprüfung von Arzneimitteln, Lebensmitteln, Futtermitteln und anderen Stoffen oder Produkten, die zur Erreichung der unter „Translationale und angewandte Forschung“ genannten Ziele

	erforderlich sind
☐	Schutz der natürlichen Umwelt im Interesse der Gesundheit oder des Wohlergehens von Mensch oder Tier
☐	Forschung im Hinblick auf die Erhaltung der Arten
☐	Ausbildung an Hochschulen oder Ausbildung zwecks Erwerb, Erhaltung oder Verbesserung von beruflichen Fähigkeiten
☐	forensische Untersuchungen
1.3	Wird das Projekt zur Einhaltung **regulatorischer Anforderungen**, d.h. zur Einhaltung eines nationalen Gesetzes, einer nationalen Verordnung oder eines unmittelbar anwendbaren Rechtsaktes der Europäischen Gemeinschaften oder Europäischen Union, wie etwa einer Verordnung, durchgeführt?
○	JA, alle Tierversuche im Rahmen des Projekts sollen zur Einhaltung regulatorischer Anforderungen durchgeführt werden.
○	NEIN, zumindest ein Tierversuch des Projekts wird nicht zur Einhaltung regulatorischer Anforderungen durchgeführt.
1.3.1	Benennung der anzuwendenden Prüfvorschrift(en) oder Verweis auf den Projektvorschlag (gegebenenfalls Gutachten) Seite ___ bzw. Punkt ___:

2. Angaben zum Nutzen

**Lfd. Nr.**	**Feldtitel bzw. -beschreibung**
2.1	Wie groß ist der **erwartete wissenschaftliche Nutzen oder pädagogische Wert des Projekts**?
○	gering
○	mittel
○	groß
2.2	Wem können die **Ergebnisse** des Projekts **letztlich zugutekommen**? (Mehrfachauswahl möglich)
☐	Menschen
☐	Tieren
☐	Umwelt
2.3	**Begründung** der Angabe zu den Kriterien 2.1 und 2.2 durch Beschreibung der getätigten Angaben sowie den dahinterstehenden, ethischen Erwägungen oder durch Verweis auf den Projektvorschlag (gegebenenfalls Gutachten) Seite ___ bzw. Punkt ___:
2.4	Wie groß ist der erwartete **Nutzen** aus dem Projekt **für andere wissenschaftliche oder pädagogische Zwecke?**
○	Kriterium nicht anwendbar.
○	gering
○	mittel
○	groß
2.4.1	**Begründung** der Angabe zu Kriterium 2.4 mit Bezug zu den jeweiligen Zwecken durch Beschreibung der getätigten Angabe oder durch Verweis auf den Projektvorschlag (gegebenenfalls Gutachten) Seite ___ bzw. Punkt ___:
2.5	Wie hoch ist der Stellenwert des Projekts innerhalb der einschlägigen **internationalen Forschungslandschaft**?
○	Kriterium nicht anwendbar.
○	gering
○	mittel
○	groß
2.5.1	**Begründung** der Angabe zu Kriterium 2.5 durch Beschreibung der getätigten Angabe oder durch Verweis auf den Projektvorschlag (gegebenenfalls Gutachten) Seite ___ bzw.

	Punkt ___:
2.6	Wie sieht der Beitrag des Projekts zur Vermeidung („**Replacement**“) zukünftiger Tierversuche aus?
○	Kriterium nicht anwendbar.
○	Ein solcher Beitrag ist GERING.
○	Ein solcher Beitrag ist MITTEL.
○	Ein solcher Beitrag ist GROSS.
2.6.1	**Begründung** der Angabe zu Kriterium 2.6 durch Beschreibung der getätigten Angabe oder durch Verweis auf den Projektvorschlag (gegebenenfalls Gutachten) Seite ___ bzw. Punkt ___:
2.7	Wie sieht der Beitrag des Projekts zur Verminderung („**Reduction**“) der Tierzahl in zukünftigen Tierversuchen aus?
○	Kriterium nicht anwendbar.
○	Ein solcher Beitrag ist GERING.
○	Ein solcher Beitrag ist MITTEL.
○	Ein solcher Beitrag ist GROSS.
2.7.1	**Begründung** der Angabe zu Kriterium 2.7 durch Beschreibung der getätigten Angabe oder durch Verweis auf den Projektvorschlag (gegebenenfalls Gutachten) Seite ___ bzw. Punkt ___:
2.8	Wie sieht der Beitrag des Projekts zur Verbesserung („**Refinement**“) der Bedingungen für die Zucht, Unterbringung, Pflege und Verwendung von Tieren in zukünftigen Tierversuchen aus?
○	Kriterium nicht anwendbar.
○	Ein solcher Beitrag ist GERING.
○	Ein solcher Beitrag ist MITTEL.
○	Ein solcher Beitrag ist GROSS.
2.8.1	**Begründung** der Angabe zu Kriterium 2.8 durch Beschreibung der getätigten Angabe oder durch Verweis auf den Projektvorschlag (gegebenenfalls Gutachten) Seite ___ bzw. Punkt ___:
2.9	Können die Ergebnisse des Projekts auch **auf andere Tierarten bzw. den Menschen übertragbar** sein?
○	Kriterium nicht anwendbar.
○	NEIN, eine Übertragbarkeit der Ergebnisse ist nicht zu erwarten.
○	JA, eine Übertragbarkeit der Ergebnisse auf andere Tierarten bzw. den Menschen ist zu erwarten.
2.9.1	**Begründung** der Angabe zu Kriterium 2.9 durch Beschreibung der getätigten Angabe oder durch Verweis auf den Projektvorschlag (gegebenenfalls Gutachten) Seite ___ bzw. Punkt ___:
2.10	Werden die Ergebnisse des Projekts zu einem wissenschaftlichen, praktischen oder pädagogischen Nutzen führen?
○	Nein
○	Ja
2.10.1	**Begründung** der Angabe zu Kriterium 2.10 durch Beschreibung der getätigten Angabe oder durch Verweis auf den Projektvorschlag (gegebenenfalls Gutachten) Seite ___ bzw. Punkt ___:
2.11	Ist es faktisch möglich und rechtlich zulässig, die **Verbreitung der Ergebnisse** des Projekts zu ermöglichen (*z. B. um ihre Verwendung im Wissenschaftsfeld zu ermöglichen und unnötige Wiederholungsversuche zu vermeiden*)?
○	NEIN, eine Verbreitung der Ergebnisse ist nicht möglich (Kriterium nicht anwendbar).
○	JA, eine Verbreitung der Ergebnisse wäre möglich, wird jedoch nicht angestrebt.
○	JA, eine Publikationsstrategie zur Verbreitung der Ergebnisse ist vorgesehen.
2.11.1	**Begründung** der Angabe zu Kriterium 2.11 durch Beschreibung der getätigten Angabe oder durch Verweis auf den Projektvorschlag (gegebenenfalls Gutachten) Seite ___ bzw. Punkt ___:

2.12	Wie hoch ist die **Wahrscheinlichkeit, den erwarteten Nutzen** des Projektes zu **generieren**?
◯	gering
◯	mittel
◯	groß
2.12.1	**Begründung** der Angabe zu Kriterium 2.12 durch Beschreibung der getätigten Angabe oder durch Verweis auf den Projektvorschlag (gegebenenfalls Gutachten) Seite ___ bzw. Punkt ___:

3. Angaben zu den Schäden

Lfd. Nr. **Feldtitel bzw. -beschreibung**

3.1 TIERE

3.1.1 Wie viele Tiere sollen verwendet werden? Angabe der absoluten **Zahl der Tiere**, die in Tierversuchen verwendet werden sollen:
_______ Tiere.

3.1.1.1 **Nähere Angaben** zu Kriterium 3.1.1 oder Verweis auf den Projektvorschlag (gegebenenfalls Gutachten) Seite ___ bzw. Punkt ___:

3.1.2 Sollen andere als in § 4 Z 5 lit. a TVG 2012 angeführte, nichtmenschliche Primaten verwendet werden?

3.1.2.1 Begründung der Angabe zu Kriterium 3.1.2 durch Beschreibung der getätigten Angaben sowie der dahinterstehenden, ethischen Erwägungen oder durch Verweis auf den Projektvorschlag (gegebenenfalls Gutachten) Seite ___ bzw. Punkt ___:

3.1.3 Ist die Verwendung von nichtmenschlichen Primaten (siehe Kriterium 3.1.2) absolut notwendig, um das Leben von Menschen zu schützen?

3.1.3.2 Begründung der Angabe zu Kriterium 3.1.3 durch Beschreibung der getätigten Angaben sowie der dahinterstehenden, ethischen Erwägungen oder durch Verweis auf den Projektvorschlag (gegebenenfalls Gutachten) Seite ___ bzw. Punkt ___:

3.2 UMFANG DER ERWARTETEN SCHWEREGRADE

3.2.1 Wie groß sind die zu erwartenden Schäden für die Tiere in Form von Leiden, Schmerzen und Ängsten? Angabe der absoluten Zahl der Tiere, die in Tierversuchen mit folgenden Schweregraden verwendet werden:
___ Tiere mit Schweregrad „gering“.
___ Tiere mit Schweregrad „mittel“.
___ Tiere mit Schweregrad „schwer“.

3.2.1.1 **Begründung** der Angabe zu Kriterium 3.2.1 durch Beschreibung der getätigten Angaben sowie der dahinterstehenden, ethischen Erwägungen oder durch Verweis auf den Projektvorschlag (gegebenenfalls Gutachten) Seite ___ bzw. Punkt ___:

3.2.2 Wie viele Tiere sind dem Schweregrad „keine Wiederherstellung der Lebensfunktion“ zuzurechnen? Angabe der absoluten Zahl der Tiere:
___ Tiere mit Schweregrad „keine Wiederherstellung der Lebensfunktion“.

3.2.2.1 **Begründung** der Angabe zu Kriterium 3.2.2 durch Beschreibung der getätigten Angabe sowie der dahinterstehenden, ethischen Erwägungen oder durch Verweis auf den Projektvorschlag (gegebenenfalls Gutachten) Seite ___ bzw. Punkt ___:

3.3 BESONDERE BELASTUNGEN

3.3.1 Werden durch das Projekt voraussichtlich *„starke Schmerzen, schwere Leiden oder schwere Ängste verursacht, die voraussichtlich lang anhalten und nicht gelindert werden können“*?

◯ Ja
◯ Nein

3.3.2 Gibt es **wissenschaftlich berechtigte Gründe** dafür, dass ein Tierversuch erforderlich ist, der *„starke Schmerzen, schwere Leiden oder schwere Ängste verursacht [werden], die voraussichtlich lang anhalten und nicht gelindert werden können“*?
(*Dieses Kriterium ist nur zu beantworten, wenn bei Kriterium 3.3.1 „Ja“ angegeben wurde; wenn zu gegenständlichem Kriterium „Nein“ angegeben wird, ist der Tierversuch unzulässig.*)

- ○ Ja. Nähere Angaben zu den wissenschaftlichen Gründen oder Verweis auf den Projektvorschlag (gegebenenfalls Gutachten) Seite ___ bzw. Punkt ___:
- ○ Nein

Schlagworte

Feldbeschreibung, Qualitätsprüfung, Wirksamkeitsprüfung

Zuletzt aktualisiert am

07.12.2020

Gesetzesnummer

20009431

Dokumentnummer

NOR40228240
