# Supplementary material for: Mission impossible accomplished? A European cross-national comparative study on the integration of the harm-benefit analysis into law and policy documents
Source: PLoS One. 2024 Feb 20;19(2):e0297375. doi: 10.1371/journal.pone.0297375 (PMC10878508; doi:10.1371/journal.pone.0297375)
Supplement: S3 File — (PDF) [file pone.0297375.s003.pdf]

GUIDANCE FOR HARM-BENEFIT ANALYSIS

1. LEGISLATION

The need to perform a harm-benefit analysis (HBA) has been explicitly mentioned in the European Directive 2010/36/EU **(SCIENTIFIC, 2019)** for the protection of animals used for scientific purposes in article 38(2). More specifically, the EU Directive requires that "no project is carried out unless a favorable project evaluation by the competent authority has been received."

As per recital 39 of the Directive, "It is essential, both on moral and scientific grounds, to ensure that each use of an animal is carefully evaluated as to the scientific or educational validity, usefulness and relevance of the expected result of that use. The likely harm to the animal should be balanced against the expected benefits of the project. Therefore, an impartial project evaluation independent of those involved in the study should be carried out as part of the authorization process of projects involving the use of live animals."

Article 38 clarifies the elements that should be included in the project evaluation to determine if the use of animals is justified and that the procedures are carried out in the most humane way. "The project evaluation shall consist in particular of the following:

(a) an evaluation of the objectives of the project, the predicted scientific benefits or educational value;

(d) **a harm-benefit analysis** of the project, to assess whether the harm to the animals in terms of suffering, pain and distress is justified by the expected outcome taking into account ethical considerations, and may ultimately benefit human beings, animals or the environment."

This directive is also adopted by the national laws of the individual membership countries, inclusive the Belgian law (Royal Decree of 29 May 2013 on the protection of laboratory animals , Art. 20) **(STAATSBLAD, 2021)** .

However, **the European Directive does not state, in any specific way, how to conduct an HBA** and how to make sure that benefits will truly outweigh the harm. Therefore the practical implementation of HBA is not clear for many project applicants and members of ethics committees. For this reason, Brussels Environment, in cooperation with the Brussels Commission for Animal Experimentation, has developed an HBA which has been integrated into the current project evaluation template.

2. PRACTICAL IMPLEMENTATION OF THE HARM-BENEFIT ANALYSIS

The Working Document on Project Evaluation and Retrospective Assessment of the European Commission **(COMMISSION, 2013)** states it is necessary to include sufficient information in the application to facilitate the HBA. In this way it is possible for the evaluators to make a justified judgment on the harms and benefits of the project. They state that an effective HBA requires a good understanding of the potential benefits and their impact and of all the expected harms to the animals and takes into account all the refinement measures and the likelihood of achieving predicted benefits. Further, the European Commission Working Document and the Recommendations for addressing an HBA by the AALAS-FELASA Working Group **(AURORA BRØNSTAD, 2016 JUN; 50(1 SUPPL))** describes that the harms imposed on the animals and the benefits of the animal experiments must be explained in plain language. The information in the HBA should be presented in a way such that it is clear what harm and benefit factors have been evaluated and how they have been evaluated.

3. EVALUATION OF THE HARMS AND BENEFITS IN THE BRUSSELS CAPITAL REGION

An HBA assesses whether the harm that would be caused to the animals, in terms of suffering, pain, distress and lasting harm, can be justified by the expected outcome, taking into account ethical consideration and the expected benefit to human beings, animals, or the environment.

The weighing of harms against benefits is not a simple decision-making process and requires careful consideration. A **unique, case-by-case evaluation** for each proposed project in which the importance and magnitude of the benefits is assessed will have to be performed by an ethical commission. The HBA that an ethical committee will conduct in the Brussels Capital Region is based on several factors that should be taken into account when assessing benefits and harms of animal-based experiments. All these relevant factors were listed in 4 tables (primary benefits, the likelihood of achieving the benefits, the main harms and the modulating factors for harm) and were transposed to specific questions in the project evaluation template (see also Annex I : Correlation Table). These questions should be answered carefully and in detail by the project applicant as they will be assessed by the ethical committee, which consists of a group of multidisciplinary people, including both experts and laymen. The individual assessment of the ethical committee members should be subject to a thorough discussion within the ethical committee. This discussion can finally guide the decision on potential approval of the subject.

In order to make this analysis as transparent, rigorous and legitimate as possible, the form identifies (by color-coding) the criteria that will be assessed by the Ethical Committee when conducting their HBA. By clearly identifying this well-defined set of criteria that will be considered, both the project applicant and the ethics committee can be more focused and targeted in conducting the HBA and bias will be avoided. **It is important to note that researchers themselves are also responsible for carrying out an harm-benefit assessment of their work. They should always make a critical evaluation of the need for their animal studies first.**

The following color code was used:

- Primary benefits
- Likelihood of achieving the benefits
- Main harms
- Modulating factors for harm

The criteria mentioned in the correlation table (Annex I) are specified in further detail below in such a way that a similar interpretation by all parties involved is possible.

3.1. Primary benefits

To perform a HBA in a systematic way, it is necessary to define and describe the anticipated benefits.

In general, benefits of animal experimentation can be classified in five different domains **(COMMITTEE, 2003; KATHY LABER, 2016, VOL. 50(1S))**:

- **social benefits**: include benefits for human health, animal health, environmental health – e.g. improved health or welfare, plant production, food hygiene, safeguarding the environment;
- **socioeconomic benefits** – e.g. conservation of natural resources, cheaper healthcare for all;
- **scientific benefits** – e.g. resolution of controversies, increasing scientific knowledge;
- **educational benefits** – meeting educational objectives that cannot be satisfied by using non-animal methods;
- **safety and efficacy**.

To carefully identify the benefits, five key questions should be answered: ‘who?’, ‘what?’, ‘when?’ and ‘how?’.

Benefits may be dependent of the type of research that is conducted. The primary benefits of basic research may be limited to the acquiring of new knowledge or to the understanding of underlying foundations or phenomena and observable facts, without any particular future application or use. However, these gains in knowledge are considered as intrinsically valuable and knowledge gained through research may eventually support future advances that could bring benefits to humans, animals and environment **(DAVIES, 2017)**. However, these future advances may only be assessed over long periods of time. Therefore, expanding of knowledge may be an appropriate benefit in its own right, but should, where possible, be linked to dissemination of results and longer-term benefits **(DAVIES, 2017)**. For applied research it may be easier to identify direct impacts on human, animal or environmental health. However, in applied research the expectations are often not met in terms of benefits. Each research project is likely to generate only a small part of the expected benefits **(DAVIES, 2017)**. The

gap between fundamental and applied research may therefore be narrower than commonly taught. For regulatory testing, the benefits are in general limited to safety and efficacy benefits. There are however legal requirements that these are conducted. The benefits in these experiments are viewed in terms of the need to facilitate regulatory decisions for the protection of man and the environment, rather than the utility of the end-product **(OFFICE, 2014)**. For education and formation, the benefits can be linked to the domain of educational benefits.

In the assessment of benefits, it is also important to consider the timescale in which these benefits will manifest. It is suggested that first the immediate or short-term benefits should be considered. Second, the medium- and long-term benefits should be taken into account **(COMMISSION, 2013)**. Short-term benefits may be readily measurable, but medium-term benefits and long-term benefits are more difficult to assess.

3.2. Likelihood of achieving benefits

When assessing benefits, it is also important to take the likelihood of achieving those benefits in consideration. Several factors that influence this likelihood of achieving benefits can be determined and are mostly related to the scientific quality of the experiment that will be conducted.

Scientific quality impacts benefit in a way that it is a fundamental criterion to obtain reliable information and to generate benefit. Factors related to scientific quality that influence benefits include **(DAVIES, 2017; AURORA BRØNSTAD, 2016 JUN; 50(1 SUPPL); COMMISSION, 2013; GRIFFIN G, 2014 APR;33(1))**:

- **originality or novelty of the methods** – Is the study original? Will new methods be used? Is it certain that the same study has not been conducted before? Duplication of studies should be avoided;
- **statistical analysis** – What statistical analysis will be used? How will data be analyzed?;
- **clear experimental design** – How will the objectives be obtained with high quality/effective use of resources (animals, time, etc.)? Is there an appropriate choice of animal model and an appropriate number of animals that will be used? How is the optimal number of animals determined? How will control and experimental groups be used? The quality of the experimental design should be considered to make sure that the obtained data are scientifically acceptable;
- **clear objectives (SMART)** – How will the objectives be met? What will the objectives be? Are the objectives SMART (specific, measurable, achievable, realistic and timely), and how is this ensured?;
- **available resources and funding** – Are there sufficient resources and funding to conduct this study?;
- **experience of the research team** – What are the experiences of the research team? Does the research team have experience in this field of research? What are the previous results of similar studies conducted by the research team?;

Other factors influencing the likelihood of achieving benefits include **(COMMISSION, 2013; GRIFFIN G, 2014 APR;33(1))**:

- **liaison with other research groups and links to other areas of research**– What is the larger body of knowledge this study contributes to? Does a link exist between this study and studies of other research groups or studies in other areas of research? Consideration of how well this work adds to the continuum of knowledge gained from previous studies or studies in other areas of research;
- **record of success of previous experiments** – What is the record of success of previous experiments conducted by the research group? Have similar studies been conducted by the research group? What were the outcomes of previous experiments conducted by the research group?

3.3. Main harms

Harms can be defined as adverse welfare effects (including pain, suffering, distress or lasting harm) likely to be experienced by the animals used during the course of the experiment. These effects may be produced by acts of commission or omission and they may be immediate or delayed. Further, they may be a specific consequence of the procedures (project-related harm) or the result of the care and husbandry systems (contingent harm) **(Office, 2014)**. Harms can arise by acts that cause harm to the animals, but also from pleasures (things that will have a beneficial effect on the animal) denied to the animals. Further, harms can be experienced consciously by animals, in that they are aware of the harms, but there may also be harms that the animal is not aware of. However, these should also be taken into account when assessing harms.

To help identify animal suffering, the Farm Animal Welfare Council (FAWC) from the Brambell Report (1965) developed the Five Freedoms. These were originally defined for farm animals but were later adapted to research animals **(REID, 1994; Mellor DJ, Stafford KJ. Integrating practical, regulatory and ethical strategies for enhancing farm animal welfare., 2001 Nov;79(11))**. These domains are devised to provide a thorough, systematic and comprehensive means to assess negative welfare impacts (Mellor, 2016). The domains are based on the idea that an animal's welfare will be good when its nutritional, environmental, health, behavioral and mental needs are met **(REID, 1994)**. The Five Domains of potential animal welfare compromise are listed as follows **(Mellor DJ. Comprehensive assessment of harms caused by experimental, teaching and testing procedures on live animals., 2004 Jun;32 Suppl 1B 4)**:

- **nutrition** – water deprivation, food deprivation, malnutrition; – e.g. restrictions on water intake, food intake, food quality and food variety, voluntary overeating, force-feeding;
- **environment** – environmental challenge – e.g. thermal extremes, unsuitable substrate, close confinement, atmospheric pollutants, unpleasant or strong odors, inappropriate intensity of light, loud or unpleasant noise, environmental monotony, unpredictable events;
- **health** – disease, injury, functional impairment– e.g. acute or chronic disease, acute or chronic injury, husbandry mutilations, functional impairment due to limb amputation or lung, heart, vascular, kidney, neural or other problems, poisons, obesity or leanness, poor physical fitness such as muscle deconditioning;
- **behavior** – behavioral or interactive restriction – e.g. invariant environment (ambient, physical, biotic), inescapable sensory impositions, choices markedly restricted, constraint on environment-focused activity, constraint on animal-to-animal interactions, limits on threat avoidance, escape or defensive activity, limitations on sleep/rest;
- **mental state/experience** – anxiety, fear, pain, distress, thirst, hunger, boredom – e.g. thirst, hunger, malnutrition malaise, bloated, gastrointestinal pain, thermal discomfort (chilling, overheating), physical discomfort (joint pain, skin irritation, stiffness, muscle tension), respiratory discomfort (breathlessness), olfactory discomfort, auditory discomfort (impairment, pain), visual discomfort (glare or darkness eye strain), malaise from unnatural constancy, many types of pain, debility, weakness, sickness, malaise, nausea, dizziness, physical exhaustion, anger, frustration, boredom, helplessness, loneliness, isolation, depression, sexual frustration, anxiety, fearfulness, panic, anger, neophobia, exhaustion.

The first four domains represent physical elements of animal welfare. The fifth domain encompasses the mental element. Compromise in the first four domains will be accumulated in the fifth domain, which includes the component of suffering.

3.4. Modulating factors for harm

Besides the main harms, there are other factors that should be taken into account when assessing harms because they may aggravate and/or mitigate the harms imposed on the animals (Commission, 2013; Griffin G, 2014 Apr;33(1); Kathy Laber, 2016, Vol. 50(1S)):

- **methods used to control adverse effects** – description of the methods that are used to minimize harm according to the principle of the 3Rs (refinement, replacement, reduction – e.g. by using a different species or strain, obtaining animals from a different source, adapting or enriching animal housing and care, modifying the techniques involved, enhancing the monitoring of the animals and implementing humane endpoints, better use of anesthesia and analgesia and/or provision of other special care;
- **frequency of procedures** – the frequency of repetition of the procedures that impose harm on the animals;
- **duration of procedures and the duration in proportion to the lifespan of the animal** – the duration that the animals will be exposed to the procedures that induce harm and the duration of suffering in proportion to the total lifespan of the animal;
- **severity level (see** Fout! Verwijzingsbron niet gevonden.**)** – the severity level assigned to the experiment following the requirements of the European Directive 2010/63 (Commission, 2013);
- **animal species** – the species proposed for the project; potential relevant factors include: sentience, cognitive ability, phylogenetic scale, adaptation to laboratory conditions, rarity and societal concern;
- **number of animals** – the total number of animals that will be used in the project;

- **the way in which the experiment will be terminated** – description of the way in which the experiment will be terminated and explanations on how/if endpoints ensure that animals are not subjected to unnecessary suffering (i.e. humane endpoints);
- **whole life experience** – prior use of the animals (including number of uses and the time between uses and potential for recovery), fate of the animals (re-use or death), potential sensitization (increasing impact) or habituation (decreasing impact), and life events unrelated to the project that may affect how pain or distress is experienced (e.g. early maternal separation, painful event as neonate);
- **health status of the animals** – clinical and subclinical conditions that could cause harm to the animals;
- **housing** – enclosure size and characteristics, social-individual housing, environmental enrichment;
- **care, health care, monitoring regime** – quality and provision of food, water, sanitation and identification, the provided health care, and the regime of health monitoring;
- **genetic modulation** – genetic modifications that result in impact on the animal well-being;
- **staff competence** – competence of the animal care personnel regarding the care of the animals, and competence of the research team regarding the experimental procedures
- **origin of animals** – the origin or source from which the animals are acquired and the acclimatization procedure that will be applied for the animals;
- **transportation** – frequency and distance of transportation of the animals prior, during or after the experiment.

Each modulating factor for harm may mitigate or aggravate the harm inflicted on the animals due to the experiment. The effect may only be aggravating or mitigating, but also both effects are possible.

4. REFERENCES

Aurora Brønstad, C. E. (2016 Jun; 50(1 Suppl)). Current concepts of Harm-Benefit Analysis of Animal Experiments - Report from the AALAS-FELASA Working Group on Harm-Benefit Analysis - Part 1. *Laboratory Animals*, 1-20. doi:10.1177/0023677216642398.

Commission, E. (2013, 09 18-19). *ec.europa.eu*. Opgehaald van European Commission: https://ec.europa.eu/environment/chemicals/lab_animals/pdf/guidance/project_evaluation/en.pdf

Committee, T. A. (2003). *Review of cost-benefit assessment in the use of animals in research.* UK. Opgehaald van https://assets.publishing.service.gov.uk/government/uploads/system/uploads/attachment_data/file/119027/cost-benefit-assessment.pdf

Davies, P. G. (2017). *Review of harm-benefit analysis in the use of animals in research.* UK: Assets Publishing. Opgehaald van https://assets.publishing.service.gov.uk/government/uploads/system/uploads/attachment_data/file/675002/Review_of_harm_benefit_analysis_in_use_of_animals_18Jan18.pdf

Griffin G, C. J.-H. (2014 Apr;33(1)). Scientific uses of animals: harm-benefit analysis and complementary approaches to implementing the three Rs. Rev Sci Tech. 2014 Apr;33(1):265-72. doi: 10.20506/rst.33.1.2283. PMID: 25000799. *Rev Sci Tech.*, 265-72. doi:doi: 10.20506/rst.33.1.2283

Kathy Laber, C. E. (2016, Vol. 50(1S)). Recommendations for Addressing Harm–Benefit Analysis and Implementation in Ethical Evaluation – Report from the AALAS–FELASA Working Group on Harm–Benefit Analysis – Part 2. *Laboratory Animals*, 21–42.

Mellor DJ, Stafford KJ. Integrating practical, regulatory and ethical strategies for enhancing farm animal welfare. (2001 Nov;79(11)). *Aust Vet J.*, 762-8. doi:doi: 10.1111/j.1751-0813.2001.tb10895.x.

Mellor DJ. Comprehensive assessment of harms caused by experimental, teaching and testing procedures on live animals. (2004 Jun;32 Suppl 1B 4). *Altern Lab Anim.*, 53-7. doi:doi: 10.1177/026119290403201s73.

Office, H. (2014). *Guidance on the Operation of the Animals (Scientific Procedures) Act 1986.* UK: Assets Publishing.

REID, D. M. (1994). Concepts of animal well-being and predicting the impact of procedures on experimental animals. 3-18. Opgehaald van https://org.uib.no/dyreavd/harm-benefit/Concepts%20of%20animal%20well-being%20and%20predicting.pdf

Scientific, E. P. (2019, 06 26). *EUR-Lex*. Opgehaald van eur-lex.europa.eu: http://data.europa.eu/eli/dir/2010/63/2019-06-26

Staatsblad, B. (2021, 09 10). *www.ejustice.just.fgov.be*. Opgehaald van Justel: http://www.ejustice.just.fgov.be/eli/besluit/2013/05/29/2013024221/justel

ANNEX I : CORRELATION TABLE (FACTORS TAKEN INTO ACCOUNT FOR THE HBA AGAINST THE QUESTIONS IN THE PROJECT EVALUATION TPL)

Table 1 Primary benefits

	CORRESPONDS WITH	Pg.
SOCIAL BENEFITS	(iii) Scientific, social, socio-economical, educational, environmental, veterinary and/or medical relevance (including who will benefit from this research and when):	5
SOCIOECONOMIC BENEFITS	(iii) Scientific, social, socio-economical, educational, environmental, veterinary and/or medical relevance (including who will benefit from this research and when):	5
SCIENTIFIC BENEFITS	(iii) Scientific, social, socio-economical, educational, environmental, veterinary and/or medical relevance (including who will benefit from this research and when):	5
EDUCATIONAL BENEFITS	(iii) Scientific, social, socio-economical, educational, environmental, veterinary and/or medical relevance (including who will benefit from this research and when):	5
SAFETY AND EFFICACY	(iv) Safety and efficacy benefits:<br>Regulatory use and Routine production only	4

Table 2 Likelihood of achieving benefits

	CORRESPONDS WITH	Pg.
**MODULATING FACTORS RELATED TO SCIENTIFIC QUALITY**		
**ORIGINALITY OR NOVELTY OF THE METHODS**	(i) Background and state of the art:<br>(ii) Goals that are specific to the project:<br>(iv) Bibliographical references that contribute to the justification of the proposed research and the references of legal guidelines to support the necessity of the work described and / or benefits and relevant references for specific models that are proposed in your work program<br>Are you aware of any identical experiments that were performed in the past? If yes, please explain why these are not a mere duplication of experiments.	5<br>5<br>5<br>8
**STATISTICAL ANALYSIS**	(iii) Justification for the number of animals.	9
**CLEAR EXPERIMENTAL DESIGN**	(i) Describe in detail all actions / procedures performed (e.g. volume and frequency of sampling, etc.). To understand the chronology of the operations, an illustrative timeline is strongly recommended.	9
**CLEAR OBJECTIVES (SMART)**	General description, purpose and justification of the project.	5
**AVAILABLE RESOURCES AND FUNDING**	Funding	2
**EXPERIENCE OF THE RESEARCH TEAM**	Personnel	12
**OTHER MODULATING FACTORS**		
**LIASON WITH OTHER RESEARCH GROUPS AND LINKS WITH OTHER AREAS OF RESEARCH**	Identification of the partner establishment(s) (Internal or external)<br>Are special efforts being made to reduce the number of animals used (e.g. collaboration with other researchers, shared use of animals, allowing different laboratories to use the organs of the same animal)?	3<br>8
**RECORD OF SUCCESS OF PREVIOUS EXPERIMENTS**	Pilot study	2

Table 3 Main harms

	CORRESPONDS WITH	Pg.
NUTRITION	(iii) Are there other deviations from the standards (e.g., housing, specific diet, fasting…) described in Annex 4 of the Royal Decree of 29 May 2013. If yes, justify the scientific, animal-welfare or animal-health reasons and the duration of this deviation. Explain also the possible negative consequences for the animals and specify what measures are taken to limit those negative effects:<br>(iv) Explain the expected adverse effect of each procedure that is applied. State how you intend to control those effects (e.g. analgesics, anaesthesia, conditioning / training, enrichment, etc.) to minimize the severity. Detail the analgesia protocol, or any other mean used to mitigate these adverse effects (anaesthesia, anti-inflammatory drugs, antibiotics…). Provide the list of medication, as well as the dose, route of administration, duration and frequency. Specify which references were consulted to choose the most appropriate method of analgesia / anaesthesia (bibliographic reference or name and position of the person being consulted):	10<br>10
ENVIRONMENT	(iii) Are there other deviations from the standards (e.g., housing, specific diet, fasting…) described in Annex 4 of the Royal Decree of 29 May 2013. If yes, justify the scientific, animal-welfare or animal-health reasons and the duration of this deviation. Explain also the possible negative consequences for the animals and specify what measures are taken to limit those negative effects:<br>(iv) Explain the expected adverse effect of each procedure that is applied. State how you intend to control those effects (e.g. analgesics, anaesthesia, conditioning / training, enrichment, etc.) to minimize the severity. Detail the analgesia protocol, or any other mean used to mitigate these adverse effects (anaesthesia, anti-inflammatory drugs, antibiotics…). Provide the list of medication, as well as the dose, route of administration, duration and frequency. Specify which references were consulted to choose the most appropriate method of analgesia / anaesthesia (bibliographic reference or name and position of the person being consulted):	10<br>10
HEALTH	(iv) Explain the expected adverse effect of each procedure that is applied. State how you intend to control those effects (e.g. analgesics, anaesthesia, conditioning / training, enrichment, etc.) to minimize the severity. Detail the analgesia protocol, or any other mean used to mitigate these adverse effects (anaesthesia, anti-inflammatory drugs, antibiotics…). Provide the list of medication, as well as the dose, route of administration, duration and frequency. Specify which references were consulted to choose the most appropriate method of analgesia / anaesthesia (bibliographic reference or name and position of the person being consulted):	10

**BEHAVIOUR**	(iv) Explain the expected adverse effect of each procedure that is applied. State how you intend to control those effects (e.g. analgesics, anaesthesia, conditioning / training, enrichment, etc.) to minimize the severity. Detail the analgesia protocol, or any other mean used to mitigate these adverse effects (anaesthesia, anti-inflammatory drugs, antibiotics…). Provide the list of medication, as well as the dose, route of administration, duration and frequency. Specify which references were consulted to choose the most appropriate method of analgesia / anaesthesia (bibliographic reference or name and position of the person being consulted):	10
**MENTAL STATE/EXPERIENCE**	(ii) Are animals single housed from the start or during the course of the procedure?<br>(iv) Explain the expected adverse effect of each procedure that is applied. State how you intend to control those effects (e.g. analgesics, anaesthesia, conditioning / training, enrichment, etc.) to minimize the severity. Detail the analgesia protocol, or any other mean used to mitigate these adverse effects (anaesthesia, anti-inflammatory drugs, antibiotics…). Provide the list of medication, as well as the dose, route of administration, duration and frequency. Specify which references were consulted to choose the most appropriate method of analgesia / anaesthesia (bibliographic reference or name and position of the person being consulted):	10<br>10

Table 4 Modulating factors for harm

	CORRESPONDS WITH	Pg.
**METHODS USED TO CONTROL ADVERSE EFFECTS**	(ii) Are animals single housed from the start or during the course of the procedure? If yes, justify the scientific, animal-welfare or animal-health reasons and the duration of this deviation and specify what measures are taken to limit the discomfort (e.g. enrichment):<br>(iii) Are there other deviations from the standards (e.g., housing, specific diet, fasting…) described in Annex 4 of the Royal Decree of 29 May 2013. If yes, justify the scientific, animal-welfare or animal-health reasons and the duration of this deviation. Explain also the possible negative consequences for the animals and specify what measures are taken to limit those negative effects:<br>(iv) Explain the expected adverse effect of each procedure that is applied. State how you intend to control those effects (e.g. analgesics, anaesthesia, conditioning / training, enrichment, etc.) to minimize the severity. Detail the analgesia protocol, or any other mean used to mitigate these adverse effects (anaesthesia, anti-inflammatory drugs, antibiotics…). Provide the list of medication, as well as the dose, route of administration, duration and frequency. Specify which references were consulted to choose the most appropriate method of analgesia / anaesthesia (bibliographic reference or name and position of the person being consulted):<br>f) Humane endpoints	10<br>10<br>10<br>11
**FREQUENCY OF PROCEDURES**	Give a general overview of the different experiments. Adding a timeline or diagram can help to clarify the overview. The details of the procedures per experiment are set out in the next section.<br>(ii) Number of experimental groups and animals per group	9<br>9
**DURATION & DURATION IN PROPORTION TO LIFESPAN**	d) Re-use of animals	10
**SEVERITY LEVEL**	e) Severity classification	11
**ANIMAL SPECIES**	Species and number of animals	6
**NUMBER OF ANIMALS**	Species and number of animals	6
**THE WAY IN WHICH THE EXPERIMENT WILL BE TERMINATED**	Fate of the animals kept alive (if applicable)<br>Method(s) of humane killing	11<br>12

**WHOLE LIFE EXPERIENCE**	(ii) Number of experimental groups and animals per group<br>d) Re-use of animals	9<br>10
**HEALTH STATUS**	Species and number of animals	6
**HOUSING**	(iii) Are there other deviations from the standards (e.g., housing, specific diet, fasting…) described in Annex 4 of the Royal Decree of 29 May 2013. If yes, justify the scientific, animal-welfare or animal-health reasons and the duration of this deviation. Explain also the possible negative consequences for the animals and specify what measures are taken to limit those negative effects:	10
**CARE, HEALTH CARE, MONITORING REGIME**	(i) Indicate how the monitoring of animal welfare during the experiment will be guaranteed, in particular the frequency of the observations and the monitoring of the inconvenience.	10
**GENETIC MODULATION**	Species and number of animals	6
**STAFF COMPETENCE**	Personnel	12
**ORIGIN OF ANIMALS**	Origin of animals (copy and paste the table below if you use more than one supplier):	6
**TRANSPORTATION**	Transport (applicable between different sites and external partners)	4
