# Supplementary material for: Mission impossible accomplished? A European cross-national comparative study on the integration of the harm-benefit analysis into law and policy documents
Source: PLoS One. 2024 Feb 20;19(2):e0297375. doi: 10.1371/journal.pone.0297375 (PMC10878508; doi:10.1371/journal.pone.0297375)
Supplement: S4 File — (PDF) [file pone.0297375.s004.pdf]

GIRCOR

GRICE

Guide de l'évaluation éthique des projets impliquant l'utilisation d'animaux à des fins scientifiques

1ère édition - février 2021

GIRCOR

GRICE

Guide de l'évaluation éthique des projets impliquant l'utilisation d'animaux à des fins scientifiques

Dépôt légal – 1ère édition : février 2021
© GIRCOR, 2021
GIRCOR
Faculté de médecine Pitié Salpêtrière
91 boulevard de l'Hôpital
75634 PARIS Cedex 13

DOCUMENT ÉLABORÉ PAR LE GRICE
(GROUPE DE RÉFLEXION INTERPROFESSIONNEL
SUR LES COMITÉS D'ÉTHIQUE),

UN GROUPE DE TRAVAIL DU GIRCOR
(GROUPE INTERPROFESSIONNEL DE RÉFLEXION
ET DE COMMUNICATION SUR LA RECHERCHE)

SUIVANT LES RECOMMANDATIONS DU CNREEA
(COMITÉ NATIONAL DE RÉFLEXION ÉTHIQUE
SUR L'EXPÉRIMENTATION ANIMALE)

ET À LA DEMANDE DU MINISTÈRE CHARGÉ DE L'ENSEIGNEMENT SUPÉRIEUR ET DE LA RECHERCHE
(QUI ASSURE LE SECRETARIAT DU CNREEA)

Liberté • Égalité • Fraternité
RÉPUBLIQUE FRANÇAISE

MINISTÈRE
DE L'ENSEIGNEMENT SUPÉRIEUR,
DE LA RECHERCHE
ET DE L'INNOVATION

LE CNREEA

COMITÉ NATIONAL DE RÉFLEXION ÉTHIQUE SUR L'EXPÉRIMENTATION ANIMALE

“Au sein du dispositif réglementaire qui encadre l'utilisation des animaux à des fins scientifiques[1], le CNREEA est la commission consultative qui a pour mission d'émettre des avis sur les questions éthiques soulevées par l'expérimentation animale (articles R.214-134 et suivants du code rural et de la pêche maritime). Son secrétariat est assuré par les services de la Direction générale de la recherche et de l'innovation (DGRI) du ministère chargé de la recherche.

Dans ses missions, il est notamment chargé de formuler des recommandations aux comités d'éthique en expérimentation animale (CEEA) visant à améliorer leurs pratiques et de conduire l'élaboration et la mise à jour d'un guide de bonnes pratiques de fonctionnement des CEEA. En pratique, il a publié à ce jour deux recommandations concernant 1- la production d'anticorps par liquide d'ascite chez la souris et 2- les travaux pratiques sur organe isolé de grenouille en licence de biologie. Il a également conduit l'élaboration de deux guides : un sur les règles communes d'organisation et de fonctionnement des comités d'éthique et le présent guide.”

@ [1]www.enseignementsup-recherche.gouv.fr/cid70598/l-encadrement-reglementaire-de-l-utilisation-d-animaux-a-des-fins-scientifiques.html

LE GIRCOR

GROUPE INTERPROFESSIONNEL DE RÉFLEXION ET DE COMMUNICATION SUR LA RECHERCHE[2]

“Le GIRCOR est une association loi de 1901 qui a été créée dans le but d'expliquer au public les raisons et les conditions du recours à l'expérimentation animale en recherche biomédicale. Il rassemble les établissements de recherche publics et privés. Il prône une recherche scientifiquement valable et éthiquement acceptable.

Le GRICE (Groupe de réflexion interprofessionnel sur les comités d'éthique) est le groupe de réflexion du GIRCOR dédié aux activités des comités d'éthique en expérimentation animale (CEEA) qui a principalement pour mission de promouvoir les principes éthiques et le développement des comités d'éthique.

En 2012, le GIRCOR a été mandaté par le Directeur général de la recherche et l'innovation, pour établir deux documents de référence complémentaires relatifs à l'activité des CEEA :

- *Un document rassemblant des règles communes d'organisation et de fonctionnement des comités : recrutement et renouvellement des membres, conduite et rendu des débats, éléments constitutifs du règlement intérieur et du rapport d'activité ;*
- *Un document rassemblant les modalités de l'évaluation éthique des projets par les comités.*

Le 1er a été approuvé par le CNREEA lors de sa séance plénière du 19 octobre 2017 et diffusé par le CNREEA aux comités d'éthique et plus largement par le GIRCOR[3].

Le second fait l'objet du présent guide. Il a été établi sur la base du partage d'expérience entre des professionnels impliqués dans des comités d'éthique et regroupés au sein du GRICE, en relation avec les services de la Direction générale de la recherche et de l'innovation (DGRI). Il a été approuvé par le CNREEA à sa séance plénière du 17 septembre 2020.”

@ [2] www.recherche-animale.org

[3] https://www.recherche-animale.org/sites/default/files/gircor-grice_regles_communes_organisation_et_fonctionnement_des_comites_ethique_en_experimentation_animale_mars_2018.pdf

Membres du groupe de travail du GRICE

Fabielle ANGEL, Pauline ANTON-GAY, Fanny BELLEBEAU-BARBIER, Christophe CHARNAY, Hervé CASSARD, Anne-Dominique DEGRYSE, Delphine DENAIS-LALIEVE, Nicolas DUDOIGNON, Valérie DURAND, Isabelle GONCALVES, Christophe JOUBERT, Dominique GROUSSON, Catherine MAISONNEUVE, Sofiene MHEDHBI, Stéphan LANGONNET, Bernard PALATE, Isabelle PEYCLIT, Sabine RICHARD, Yves RICHARD, Violaine ROCHET, Frédéric SCHORSCH, Bruno VERSCHUERE, Samuel VIDAL (pilote).

Le GRICE tient à saluer les contributions essentielles de Samuel Vidal et Bruno Verschuere et leur adresse toute sa reconnaissance.

Le GRICE tient également à remercier les membres du groupe de travail du CNREEA qui ont apporté leur éclairage sur le texte proposé par le groupe de travail du GRICE :

Sandryne BRUYAS, Georges CHAPOUTHIER, Patrick GONIN, Nicolas GUY, Patricia LORTIC, Véronique MARY, Pierre MORMEDE, Valérie NIVET-ANTOINE, Emmanuel PICAVET, Sophie PICAVET, Raphaël LARRERE, Virginie VALLET.

Nicolas DUDOIGNON
Vice-Président du GIRCOR
Membre du CNREEA

Contact : contact@recherche-animale.org

GIRCOR

GRICE

Guide de l'évaluation éthique des projets impliquant l'utilisation d'animaux à des fins scientifiques

Plan

Plan

Avant-propos : Quelle éthique pour les animaux ?

1 – Introduction

2 – Définitions

2.1 – Liste des acronymes et mots clés

2.2 – Autorisation de projet

2.3 – Bien-être des animaux

2.4 – Chronogramme

2.5 – Comité d'éthique en expérimentation animale

2.6 – Degré de gravité des procédures expérimentales et types de contraintes

2.6.1 - Angoisse

2.6.2 - Détresse

2.6.3 - Dommages durables

2.6.4 - Douleur

2.6.5 - Inconfort

2.6.6 - Souffrance

2.7 – Phénotype dommageable

2.8 – Point limite

2.9 – Procédure expérimentale

2.10 – Projet

2.11 – Règle des 3R : Remplacement, Réduction et Raffinement

GIRCOR

GRICE

Guide de l'évaluation éthique des projets impliquant l'utilisation d'animaux à des fins scientifiques

Plan

3 – Principes généraux de l'évaluation éthique

3.1 – Garanties apportées par l'évaluation éthique

3.2 – Portée et valeur de l'avis du CEEA

3.3 – Relation avec l'évaluation scientifique

3.4 – Participer à la conformité règlementaire des projets

4 – Conduite de l'évaluation éthique d'un projet par le comité d'éthique

4.1 – Cadre réglementaire et méthodologie

4.2 – Documents nécessaires à l'évaluation

4.3 – Evaluation des objectifs et de la pertinence du projet

4.3.1 - Méthodes et principes d'évaluation de l'objectif et de la pertinence du projet

4.3.1.1 - Evaluation des avantages attendus

4.3.1.2 - Evaluation de l'intérêt et de la nécessité incontournable d'utiliser des animaux vivants dans le but décrit

4.3.1.3 - Possibilité d'atteindre les objectifs

4.3.2 - Diffusion et utilisation des résultats

4.4 – Evaluer la conformité du projet avec les exigences de remplacement, de réduction et de raffinement

4.4.1 - Remplacement

4.4.2 - Réduction

4.4.3 - Raffinement

4.5 – Appréciation de la classification des procédures expérimentales selon leur degré de gravité

4.6 – Appréciation des éléments techniques obligatoires

4.6.1 - Espèces animales concernées et origine des animaux

4.6.2 - Utilisation de primates

4.6.3 - Conditions d'hébergement et d'entretien des animaux

GIRCOR

GRICE

Guide de l'évaluation éthique des projets impliquant l'utilisation d'animaux à des fins scientifiques

Plan

4.6.4 - Conditions de mise à mort

4.6.5 - Licéité, choix et mise en œuvre des procédures expérimentales

4.6.5.1 - Domaine de licéité des procédures

4.6.5.2 - Choix des méthodes

4.6.5.3 - Point limite

4.6.5.4 - Procédure expérimentale impliquant une contrainte intense susceptible de se prolonger sans qu'il soit possible de la soulager

4.6.5.5 - Recours à l'anesthésie, l'analgésie ou à toute autre méthode visant à réduire les contraintes

4.6.5.6 - Utilisation continue, réutilisation des animaux et effets cumulatifs

4.7 – Analyse comparative des dommages et avantages du projet

4.8 – Nécessité d'une appréciation rétrospective

4.9 – Expression d'un avis

4.10 – Place de l'évaluation éthique dans la modification d'un projet autorisé

5 – Appréciation rétrospective des projets

5.1 – Méthodes et moyens mis en œuvre pour l'appréciation rétrospective

5.2 – Atteinte des objectifs scientifiques, réglementaires ou pédagogiques du projet

5.3 – Prise en compte des dommages constatés et de la gravité réelle des procédures expérimentales

5.4 – Contribution du projet au principe des 3R

Conclusion

Quelle éthique pour les animaux ?

Les animaux forment des lignées extrêmement variées et montrent une diversité considérable de besoins et d'états mentaux. Avant de donner, dans ce guide, quelques règles sur la manière de traiter ceux qui sont utilisés dans des expériences scientifiques ou médicales, sans doute est-il utile de rappeler, à leur propos, quelques principes généraux.

L'être humain se veut un être moral et, à ce titre, depuis l'Antiquité, les hommes ont cherché à protéger certains animaux, en considérant que, sur le plan de la sensibilité, ils ne se distinguaient pas vraiment des humains. Cette exigence a notamment fait l'objet de la célèbre requête du philosophe anglais Jeremy Bentham, qui affirmait que l'essentiel dans la protection des animaux était le respect de leur aptitude à souffrir. C'est bien cette question de la sensibilité douloureuse qui est au centre des préoccupations morales de l'éthique qui concerne l'expérimentation animale, mais aussi de l'ensemble des activités humaines à l'égard des animaux. Mais de quels animaux et de quelle sensibilité douloureuse s'agit-il ?

Dire qu'un animal est un « être sensible », c'est dire qu'il possède une sensibilité nerveuse. Les plantes sont sensibles… à la lumière et ce n'est évidemment pas de ça qu'il s'agit ici ! Mises à part de rares exceptions comme les éponges, l'écrasante majorité des animaux possèdent une sensibilité nerveuse, dite « nociception », leur permettant de repérer un agent extérieur qui risque de porter préjudice à leur corps (excès de pression, de chaleur, de froid…). Les individus concernés répondent à ces menaces par le retrait de la partie du corps menacée ou par la fuite quand elle est possible. Ces réponses sont inconscientes ou conscientes.

Chez certains groupes animaux au système nerveux très développé, qu'on appelle parfois « animaux sentients », la nociception devient consciente et on peut alors parler de douleur et de souffrance. Dans l'état actuel des connaissances, qui pourrait évoluer, c'est le cas chez les vertébrés et les mollusques céphalopodes, qui sont seuls concernés par la Directive Européenne 2010/63/EU relative à la protection des animaux utilisés à des fins scientifiques et donc par ce guide.

Il faut cependant bien voir que les impératifs moraux de ces deux textes recèlent un double conflit. Un conflit moral d'abord, qui oppose l'intérêt de l'espèce humaine (à améliorer ses connaissances et en particulier sa médecine) et l'intérêt des animaux (à ne pas souffrir). Un conflit épistémologique ensuite, puisque les animaux utilisés sont considérés comme biologiquement assez proches de l'homme pour que les résultats obtenus soient transposables aux hommes, mais assez loin tout de même pour que la question morale de leur utilisation ne se pose pas trop. Comme l'impératif moral de supprimer toute expérimentation animale est inatteignable de nos jours compte tenu des limites légales et éthiques des essais humains et de l'insuffisance des méthodes substitutives pour satisfaire les exigences d'efficacité et de sécurité de la société en matière de santé, d'alimentation ou d'environnement, les réponses de la Directive Européenne et de ce guide sont donc, moralement, des réponses de compromis, visant à définir un rapport raisonnable entre les dommages (causés aux animaux) et les avantages (pour l'homme).

Il importe de s'en souvenir et de souligner que l'expérimentation sur des animaux vivants n'est jamais un acte moralement gratuit. Le chercheur, tout en suivant les règles de ce guide, doit toujours avoir présent à l'esprit de limiter le recours aux animaux et d'améliorer, de toutes les manières possibles, le traitement des animaux qu'il utilise.

Georges CHAPOUTIER[4]
Biologiste et philosophe
Directeur de Recherche Emérite au CNRS

---

[4] *Suggestions de lecture :*
- Chapouthier G. et Tristani-Potteaux F., Le chercheur et la souris, CNRS Editions, Paris, 2013
- Le Neindre P., Dunier M., Larrère R., Prunet P. (dir.), La conscience des animaux, Paris, Quae, 2018

L'éthique du traitement des animaux

Que les réponses éthiques relèvent du compromis, voilà qui peut sembler insatisfaisant : l'éthique n'a-t-elle pas pour vocation de nous donner les moyens d'une réflexion systématique touchant la bonne manière de poser les problèmes et de les aborder, la conduite juste à tenir, ainsi que les principes corrects de l'organisation collective ? Le compromis ne renvoie-t-il pas au contraire à une manière conjoncturelle et simplement pragmatique d'allier des tendances contraires, voire des intérêts en présence, avec lesquels on a le sentiment de devoir composer ?

Cette opposition est sans doute trop simple. Les questions relatives au traitement des animaux doivent aujourd'hui être reconnues comme des questions qui divisent, qui donnent l'occasion de revendiquer la pertinence de points de vue marqués, ouvrant des chemins pratiques dans un sens ou dans un autre. Les antagonismes sont souvent très marqués. Ainsi, la thèse selon laquelle l'expérimentation animale reste « nécessaire » au regard de grands objectifs humanistes et de l'état des connaissances n'emporte pas une adhésion unanime, alors même qu'elle est habituellement présentée comme raisonnable. Les raisons de ce manque d'unanimité sont – et c'est ce qui importe le plus – compréhensibles, sinon elles-mêmes partageables sur une base très large. En effet, à mesure que s'affinent les connaissances sur les animaux (avec leurs représentations du monde ou leur cognition, leurs plaisirs et leurs souffrances, leurs émotions, leur vie sociale), et à mesure que les animaux domestiques prennent plus de place dans la vie des familles, la restriction coutumière du champ de la « considérabilité » morale aux seuls êtres humains apparaît sous un jour contestable, encore qu'il reste beaucoup à faire, dans de nombreux domaines, pour assurer aux êtres humains eux-mêmes un traitement digne dans la vie sociale.

En dépit de divisions qu'il faut prendre au sérieux, nous avons des institutions communes, qui doivent mériter sinon l'approbation de tous pour toutes leurs actions, du moins le respect d'efforts réfléchis pour tenir compte des attentes et des raisonnements éthiques qui les fondent. Pour des êtres dont le sort importe, les institutions ne peuvent donc se désintéresser de principes de bon traitement (certainement différencié en fonction des animaux), qui concernent largement la nociception, la sensation, les éléments relatifs au bien-être, sans exclure la pertinence, pour de nombreux animaux, de l'hypothèse d'une prise de relais de la sensation par des formes élaborées de conscience comparables à celle qui existent chez les hommes.

La recherche d'acceptabilité, et de progrès à cet égard, ne relève donc pas d'un goût pour le compromis qui détournerait de l'éthique proprement dite. Elle reflète le fait que des compromis s'imposent pour que les institutions répondent aux soucis éthiques qui s'expriment, sans imposer dogmatiquement un point de vue au détriment des autres et au préjudice de leur mission.

Les points de vue moraux sont inspirants mais souvent incomplets. Voudra-t-on privilégier une perspective comme celle de l'amélioration morale de l'homme à travers le bon traitement des animaux ? On se heurtera au manque d'intérêt fréquent pour ce qui n'est pas rapporté aux finalités des êtres humains. Peut-on chercher à promouvoir avant tout l'attention à la valeur « intrinsèque » de la vie et de l'intégrité corporelle des animaux ? Le problème est ici que tout le monde ne trouve pas intelligible le discours de l' « intrinsèque ». Par égard pour le pluralisme, il n'est pas arbitraire de privilégier des principes de dignité des institutions dans leur bon traitement des animaux ; ce peut être une cause de frustration mais aussi le gage d'un progrès continu.

Emmanuel PICAVET[5]

Professeur des universités (Ethique appliquée). Directeur du Centre de Philosophie Contemporaine de la Sorbonne (université Paris 1 Panthéon-Sorbonne)

---

[5] *Suggestions de lecture :*
- Picavet, E. 2020 "Valeur du désaccord et nature des compromis", in La Valeur du désaccord, dir. Loïc Nicolas, Jérôme Ravat et Albin Wagener, Paris, Les Editions de la Sorbonne (p. 73-83).

1 Introduction

En 2009, afin d'assister les comités d'éthique français dans leur démarche spontanée et volontaire, le Groupe de réflexion interprofessionnel sur les comités d'éthique (GRICE) a édité un Guide de l'Evaluation Ethique des Etudes sur Animaux dont l'objectif était de faciliter et d'homogénéiser la revue éthique des protocoles d'étude par les comités d'éthique, au sein des organismes et établissements de recherche, qu'ils soient publics ou privés. Depuis 2007, le GRICE anime également des formations pour les membres de comités d'éthique dans ce même but d'harmonisation des approches entre les différents comités, dont la mise en place n'était alors pas obligatoire.

Depuis 2013, la règlementation française, basée sur la Directive européenne 2010/63/UE, impose l'autorisation des projets qui comportent une ou plusieurs procédures expérimentales utilisant des animaux à des fins scientifiques, y compris réglementaires ou pédagogiques. Accordée par le ministère chargé de la recherche, l'autorisation de projet ne peut intervenir que si le projet a fait l'objet d'une évaluation éthique préalable. Cette dernière intègre des dispositions règlementaires précises et est prise en charge par les comités d'éthique en expérimentation animale (CEEA).

Les membres du CEEA, dénommés dans ce guide « évaluateurs », ont pour mission de construire un avis argumenté sur le projet soumis à leur évaluation avant son autorisation par le ministère. Alors seulement le projet pourra être réalisé.

L'objectif de ce guide est d'aider chacun des membres des CEEA à remplir la mission d'évaluation éthique préalable à l'autorisation et à leur réalisation, mais également d'aider les membres dans une autre mission d'évaluation éthique, réalisée une fois que les projets sont terminés : l'appréciation rétrospective des projets.

En dehors du cadre règlementaire et selon les termes de la Charte nationale, les CEEA sont invités à promouvoir les principes éthiques et, en ce sens, ils peuvent procéder à des évaluations sur d'autres sujets, non couverts par la règlementation, organiser des formations ou des opérations de communication sur le sujet de l'éthique en expérimentation animale. Les activités de promotion des principes éthiques identifiée dans la Charte nationale ne seront pas traitées spécifiquement dans ce guide. Toutefois, elles seront menées par les comités en cohérence avec ce guide et, plus généralement, avec la règlementation.

2.1 Liste des acronymes et mots clés

**AM**	Arrêté Ministériel : Arrêté du 1er Février 2013 relatif à l'évaluation éthique et à l'autorisation de projet impliquant l'utilisation d'animaux dans des procédures expérimentales[6] (les autres arrêtés seront mentionnés avec leur objet)
**AR**	Appréciation rétrospective
**APAFiS**	Autorisation de Projet utilisant des Animaux à des Fins Scientifiques
**CEEA**	Comité d'Ethique en Expérimentation Animale
**Charte nationale**	Charte nationale portant sur l'éthique de l'expérimentation animale, élaborée et mise à jour par le CNREEA en 2014[7]
**CNREEA**	Comité National de Réflexion Ethique sur l'Expérimentation Animale
**CRPM**	Code Rural et de la Pêche Maritime
**DAP**	Demande d'Autorisation de Projet
**DD(CS)PP**	Direction Départementale (de la Cohésion Sociale et) de la Protection des Populations
**Demandeur**	Etablissement utilisateur qui soumet une DAP via la plateforme APAFiS, représenté dans les échanges avec le CEEA par du personnel concepteur qui rédige la DAP
**EU**	Etablissement Utilisateur
**Evaluateur**	Membre de CEEA en charge de l'évaluation de projet
**GIRCOR**	Groupe Interprofessionnel de Réflexion et de Communication sur la Recherche[8]
**GRICE**	Groupe de Réflexion Interprofessionnel sur les Comités d'Ethique
**PE**	Procédure expérimentale
**SBEA**	Structure chargée du Bien-Etre des Animaux

[6] *https://www.legifrance.gouv.fr/affichTexte.do?cidTexte=JORFTEXT000027038013&categorieLien=id*
[7] *https://cache.media.enseignementsup-recherche.gouv.fr/file/Encadrement_des_pratiques_de_recherche/57/9/Charte_nationale_portant_sur_l_ethique_de_l_experimentation_animale_243579.pdf*
[8] *www.recherche-animale.org*

2.2 Autorisation de projet

L'autorisation de projet est une autorisation règlementaire définie par la directive européenne de 2010 et délivrée par le ministère chargé de la recherche après avis du CEEA. Elle doit être obtenue préalablement à la mise en œuvre de tout projet utilisant des animaux vivants à des fins scientifiques entrant dans le champ d'application de la section 6 du CRPM (articles R.214-87 et suivants).

Toute utilisation d'un animal dans une procédure expérimentale (PE) doit être rattachée à une autorisation de projet en cours de validité dans l'établissement utilisateur concerné. La notification d'autorisation du ministère peut être demandée à tout moment par les autorités de contrôle.

Les autorisations de projet sont délivrées pour une durée maximale de 5 ans à des demandeurs identifiés (établissements utilisateurs). Chaque autorisation est propre à un projet et un établissement utilisateur agréé par les services déconcentrés du ministère chargé de l'agriculture. Les modalités d'obtention des autorisations de projets sont précisées dans l'arrêté du 1er février 2013 relatif à l'évaluation éthique et à l'autorisation des projets impliquant l'utilisation d'animaux dans des PE.

2.3 Bien-être des animaux

L'ANSES, dans son rapport du 16 février 2018[9], définit le bien-être des animaux placés sous la responsabilité de l'homme de la façon suivante :

> « Le bien-être d'un animal est l'état mental et physique positif lié à la satisfaction de ses besoins physiologiques et comportementaux, ainsi que de ses attentes. Cet état varie en fonction de la perception de la situation par l'animal. »

[9] *Avis de l'Agence nationale de sécurité sanitaire de l'alimentation, de l'environnement et du travail relatif au « Bien-être animal : contexte, définition et évaluation. 16 février 2018 – Saisine n° « 2016-SA-0288 » (https://www.anses.fr/fr/system/files/SABA2016SA0288.pdf)*

L'Organisation mondiale de la santé animale (OIE) précise :

> Si la notion de bien-être animal se réfère à l'état de l'animal, le traitement qu'un animal reçoit est couvert par d'autres termes tels que soins, conditions d'élevage et bientraitance.

En 1979, le Farm Animal Welfare Council[10] a formalisé les grands principes du bien-être des animaux qui sont encore connus actuellement sous le terme de « Five freedoms » ou « Les cinq libertés » en français : l'absence de faim et de soif (liberté physiologique), l'absence d'inconfort (liberté environnementale), l'absence de douleur, de blessures et de maladies (liberté sanitaire), la liberté d'expression d'un comportement normal (liberté comportementale), l'absence de peur et de détresse (liberté mentale). Le respect de ces principes est à la base des référentiels d'évaluation du bien-être des animaux lorsqu'ils sont placés sous la responsabilité de l'homme.

Veiller au bien-être des animaux hébergés dans les établissements utilisateurs – toutes espèces confondues – nécessite une solide connaissance des besoins des espèces animales et la capacité à identifier leurs attentes et leurs états mentaux par l'observation de leur état général et de leur comportement. Tous les animaux d'une même espèce ne sont pas sensibles à leur environnement de la même façon et le bien-être a aussi une dimension individuelle.

Dans le contexte de l'expérimentation, il est le plus souvent difficile de parler de bien-être et c'est le concept de protection des animaux qui prévaut. Les principes en ont été définis dès 1959 par Russell et Burch sous la dénomination des 3R pour « Remplacer, Réduire et Raffiner » en français, dont le respect est à la base de la règlementation [cf. § 2.11].

L'étude du bien-être animal est un domaine dynamique où les connaissances évoluent vite et pour laquelle la formation continue est importante.

---

[10] *https://en.wikipedia.org/wiki/Five_freedoms*

2.4 Chronogramme

Un chronogramme est une représentation graphique des différentes étapes d'un projet ou d'une PE sur une échelle de temps.

La fourniture d'un chronogramme ou plan expérimental pour les PE et les projets n'est pas évoquée dans la règlementation et n'est pas exigée dans les formulaires de saisine. Cependant, elle s'avère très utile à la compréhension et donc à l'évaluation d'un projet ou d'une PE.

Dans le cadre de la présentation détaillée d'une ou plusieurs procédure(s) expérimentale(s), un chronogramme illustre de manière schématique l'enchaînement des différentes interventions ou manipulations réalisées sur les animaux, du début (réception ou naissance des animaux) à la fin de la PE (mise à mort, mise à disposition pour une autre procédure, placement, mise en liberté pour les animaux de la faune sauvage). Il donne une idée précise du déroulement du protocole et permet de visualiser l'ensemble des sources de contraintes appliquées aux animaux, leurs durées et les intervalles qui les séparent. Le chronogramme est très utile pour évaluer le degré de gravité d'une PE.

2.5 Comité d'éthique en expérimentation animale

Un Comité d'Ethique en Expérimentation Animale (ou CEEA) est l'autorité compétente identifiée par le ministère chargé de la recherche pour l'évaluation éthique des projets qui sont réalisés dans le ou les établissements utilisateurs qui lui sont rattachés. Il s'agit d'un groupe de travail indépendant et impartial dont la composition pluraliste et pluridisciplinaire assure la compétence nécessaire à l'évaluation éthique des projets et PE qui lui sont soumis. Ses qualités d'absence de conflit d'intérêts, d'indépendance, d'impartialité et de compétence, ainsi que le respect de la confidentialité des données, lui permettent d'être enregistré par le ministère chargé de la recherche en vue de son agrément. Les membres du CEEA s'engagent à respecter la Charte Nationale.

Les dispositions règlementaires relatives aux CEEA sont fournies dans les articles R214-117 et suivants du CRPM, ainsi que dans l'arrêté ministériel du 1er février 2013 relatif à l'évaluation éthique et à l'autorisation des projets impliquant l'utilisation d'animaux dans des PE.

Pour accompagner la règlementation, un guide de bonnes pratiques sur les règles d'organisation et de fonctionnement des CEEA en France a été publié

Retour au sommaire

par le GIRCOR en mars 2018[11]. Son élaboration a été conduite par le CNREEA et ce dernier l'a transmis aux comités d'éthique.

2.6 Degré de gravité des procédures expérimentales et types de contraintes

Également nommé degré de sévérité, le degré de gravité est une notion qui a été introduite dans la règlementation par la Commission Européenne en 2010 pour classer les PE en fonction des types de contraintes qu'elles imposent aux animaux et des dommages (douleur, angoisse, souffrance ou dommage durable) qui en découlent. Ces dommages sont définis ci-après (pour une analyse plus profonde, consulter les documents d'Expertise Scientifique Collective de l'INRA[12]).

Le degré de gravité d'une PE est déterminé en fonction de l'intensité et de la durée de la douleur, de la souffrance, de l'angoisse ou du dommage durable qu'un animal donné risque de subir au cours de la PE. Il existe 4 degrés de gravité : léger, modéré, sévère, sans réveil[13].

Le degré de gravité est évalué, pour chaque PE, en prenant en compte tous les effets potentiellement adverses sur les animaux, qu'ils soient psychologiques ou physiques.

L'évaluation du classement des PE est réalisée avant leur mise en œuvre au cours du processus d'autorisation de projet. Cette sévérité est également évaluée pour chaque animal après la réalisation des procédures. Dans le premier cas, on parle d'un degré de gravité évalué a priori et discuté avec le CEEA ; dans le second, il s'agit du degré de gravité réelle qui sera rapporté au ministère chargé de la recherche dans les enquêtes statistiques annuelles sur l'utilisation des animaux à des fins scientifiques.

---

[11] Règles communes d'organisation et de fonctionnement des comités d'éthique en expérimentation animale – GIRCOR- GRICE 1ère édition. Mars 2018

[12] Expertise Scientifique Collective INRA « Douleurs animales » 2009 (https://www.inrae.fr/sites/default/files/pdf/ce6134a6e6437d208ea4f10b2b9b3744.pdf) et Expertise Scientifique Collective « Conscience animale » 2017 (https://www.quae-open.com/produit/97/9782759228713/la-conscience-des-animaux)

[13] Consulter l'annexe de l'arrêté ministériel du 1er février 2013 relatif à l'évaluation éthique et à l'autorisation des projets impliquant l'utilisation d'animaux dans des procédures expérimentales, où des exemples d'actes expérimentaux dans les différentes catégories sont proposés.

2.6.1 Angoisse

Malaise psychique et physique. Grande inquiétude, anxiété profonde pouvant aller jusqu'à la panique née de la perception d'une menace imminente, mais imprécise.

Se traduit par un état d'alerte psychique et de mobilisation somatique se manifestant par des symptômes neurovégétatifs caractéristiques (spasmes, sudation, dyspnée, accélération du rythme cardiaque, vertiges, etc.)

Une douleur forte s'accompagne d'un retentissement touchant l'émotion (voir définition de souffrance), mais également l'affectivité, menant ainsi à l'angoisse. Cette dimension est plus ou moins importante, en fonction de l'état antérieur, de l'intensité, de la durée d'une douleur, allant d'un état d'angoisse ou d'anxiété à un véritable état dépressif.

2.6.2 Détresse

Angoisse causée par un sentiment d'abandon, d'impuissance, par une situation désespérée.

2.6.3 Dommages durables

Un dommage durable est une atteinte de l'intégrité corporelle, physique ou psychique, de manière durable dans le temps, quelle que soit l'intensité associée.

2.6.4 Douleur

La nociception est une modalité sensorielle mise en jeu par tout stimulus capable de produire une lésion tissulaire et « la douleur est une expérience sensorielle et émotionnelle aversive, représentée par la "conscience" que l'animal a de la rupture ou de la menace de rupture de l'intégrité de ses tissus »[14].

Pendant très longtemps, cliniciens et chercheurs ont considéré la douleur comme une sensation témoignant d'un traumatisme ou d'une agression tissulaire, ou apparaissant au cours du développement d'un processus pathologique. Cette ébauche de définition ne rendait pas compte du cortège émotionnel inhérent à toute douleur qui se prolonge dans la durée, ni des situations de chronicité qui

---

[14] *Assessment of acute pain in farm animals using behavioral and physiological measurements. V Molony, J E Kent. J Anim Sci. 1997 Jan;75(1):266-72*

font que, sans cause organique manifeste, une douleur peut être tout aussi invalidante qu'une douleur dont l'origine corporelle est identifiée. On observe par ailleurs une grande variabilité dans la perception de la douleur d'un individu à l'autre. Des recouvrements possibles existent indiscutablement entre la douleur, considérée comme une atteinte exclusivement physique ou fonctionnelle, et la souffrance, appréhendée exclusivement dans la dimension d'atteinte d'un « projet central de vie » et qui relèverait exclusivement du domaine émotionnel.

A noter, enfin, que la douleur (surtout persistante) altère les capacités cognitives et les capacités de réaction des animaux.

2.6.5 Inconfort

Caractère désagréable, incommode d'un état dans lequel se trouve un individu ou un groupe.

2.6.6 Souffrance

Fait de souffrir, état prolongé de douleur physique ou morale.

La souffrance, définie comme la perte de toute possibilité d'expression d'un projet comportemental primordial serait la conséquence d'une incapacité « à composer, à s'adapter, à faire avec » une situation imposée durablement ; ce type de situation peut également être décrit en termes de stress.

La souffrance est toujours associée à des états émotionnels hautement déplaisants : c'est un état émotionnel de détresse associé aux évènements qui menacent l'intégrité biologique ou psychologique de l'individu. Chez l'homme, le lien entre la douleur et la souffrance a été étudié : si l'individu connait une douleur forte et durable, cette douleur devient envahissante psychiquement et peut aboutir à une souffrance également intense, avec atteinte de l'intégrité psychique. A l'inverse, une souffrance n'est pas systématiquement associée à une douleur, mais peut avoir des origines psychiques : les amputés souffrent du membre qui a connu la douleur bien que ce membre ne soit plus là.

Chez les animaux sociaux, la séparation d'un animal de son milieu social est source de souffrance morale. Il est en de même pour les couples mère/petit lorsque le petit est séparé de sa mère.

Retour au sommaire

2.7 Phénotype dommageable

Un animal présentant un phénotype[15] dommageable est un animal susceptible de ressentir, du fait de son statut génétique, une douleur, une souffrance, une angoisse ou un dommage durable d'un niveau équivalent ou supérieur à celui causé par l'introduction d'une aiguille effectuée conformément aux bonnes pratiques vétérinaires. Cette définition place d'emblée un tel animal dans le champ d'application règlementaire de la PE. Dans la règlementation référencée dans ce guide, les animaux à phénotype dommageable sont des animaux génétiquement modifiés.

En réalité, la directive 2010/63/UE utilise dans sa version anglaise le libellé « animaux génétiquement altérés », qui a été traduit dans la version française de façon très ambigüe en « animaux génétiquement modifiés ». Les animaux concernés par la directive comprennent les animaux génétiquement modifiés par l'homme au moyen de toute technique de modification génétique introduisant des mutations constitutives ou induites (transgénèse additive ou soustractive, recombinaison homologue, Cre-loxP, CRISPR-Cas 9, mutagénèse aléatoire…) et les animaux présentant des mutations naturelles spontanées, qui peuvent être dommageables de manière permanente ou différée, selon la définition de la PE, et qui sont conservées pour leur intérêt scientifique.

Dans ce contexte scientifique jargonneux et comme la directive prend en considération la contrainte vécue par tous ces types d'animaux mutants quelle que soit la façon dont leur génome a été altéré, nous proposons l'utilisation dans ce guide du libellé général **« animaux génétiquement modifiés »**.

La création d'une nouvelle lignée d'animaux génétiquement modifiés, que ce soit par génie génétique ou par le croisement de deux lignées d'animaux génétiquement modifiés, doit être autorisée. Il en est de même pour, d'une part, l'élevage (le « maintien ») d'une lignée établie d'animaux à phénotype dommageable et, d'autre part, pour toute utilisation d'animaux génétiquement modifiés dans une PE, que leur phénotype soit dommageable ou non.

Une nouvelle souche ou lignée d'animaux génétiquement modifiés est considérée comme « établie » lorsque la transmission de la modification génétique est stable, soit après deux générations au moins, et lorsqu'une évaluation du bien-être a été réalisée incluant la détermination du caractère dommageable ou non du phénotype.

[15] *ensemble des caractères apparents d'un individu*

L'évaluation du phénotype d'une nouvelle lignée d'animaux génétiquement modifiés exige ainsi de réaliser un suivi documenté des animaux pendant au moins deux cycles de reproduction (F2) incluant des animaux des deux sexes et d'âges représentatifs (naissance, sevrage, adulte et à tout autre âge pertinent considérant les effets potentiellement attendus de la modification génétique).

Ainsi, pour la création d'une nouvelle lignée, le projet doit inclure une estimation du degré de gravité du phénotype attendu. Pour l'élevage ou l'utilisation d'une lignée établie, le degré de gravité est celui du phénotype observé lors de l'évaluation du bien-être de la lignée. Il peut être léger, modéré ou sévère.

Pour plus de détails sur la notion de phénotype dommageable, ainsi que sur les paramètres à observer pour évaluer le phénotype induit par une nouvelle modification génétique, il y a lieu de consulter le document de consensus de l'Union Européenne relatif aux animaux génétiquement modifiés[16].

2.8 Point limite

Le « point limite » est aussi appelé point limite adapté, point limite précoce ou point d'arrêt anticipé. On parle aussi de « critères d'arrêt » s'agissant des signes qui indiquent l'atteinte de ce point limite.

Le « point limite » peut être défini comme **l'indicateur** qui permet d'identifier le plus précocement possible la présence d'un(e) éventuel(-le) douleur, angoisse, détresse, inconfort ou souffrance chez un animal de laboratoire et de déclencher les actions appropriées.

Le principe directeur de cette démarche est que, dans les expériences faites sur les animaux, toute souffrance, détresse ou inconfort réel ou potentiel devraient être minimisés ou allégés en choisissant le point limite **le plus précoce, compatible avec les objectifs scientifiques (ou éducatifs ou réglementaires) de la procédure expérimentale**[17].

---

[16] *https://ec.europa.eu/environment/chemicals/lab_animals/pdf/corrigendum.pdf*
[17] *Le « point limite » en expérimentation animale. CNRS, septembre 2002. http://ethique.ipbs.fr/ptlimexpanim.pdf*

L’apparition du point limite doit entraîner la mise en application de mesures immédiates, visant à éviter, réduire ou supprimer ces dommages, sur la base d’exigences éthiques **tout en tenant compte des objectifs scientifiques** (ou éducatifs ou réglementaires) définis dans le projet[18].

La mise en pratique des **points limites** doit toujours être évaluée **au regard de l’objectif expérimental poursuivi** par la procédure dont l’atteinte est définie par un ou plusieurs **points limites expérimentaux**[19] (dans le cas d’une procédure à des fins scientifiques).

Les points limites expérimentaux sont définis pour les besoins des PE décrites dans les projets. Par exemple, pour les modèles de pathologie, il s’agit le plus souvent de signes ou de paramètres observables et mesurables (cliniques, morphologiques ou comportementaux) indiquant une modification plus ou moins avancée de l’état physiologique d’un animal. Ils sont calibrés pour permettre de répondre à l’objectif scientifique de la PE, mais doivent être pensés de manière à minimiser les dommages causés aux animaux. En comparaison à ces points limites expérimentaux, on cherchera à utiliser des critères plus précoces, soit observables et qui sont les prémisses de signes plus sévères, soit qui n’ont pas de manifestation extérieure mais annoncent une modification physiologique, voire une dégradation de l’état général de l’animal, débutante ou à venir (modifications de paramètres endocriniens et/ou hémato-biochimiques, changements dans l'expression de gènes, apparition d'anticorps…). Ainsi, dans la mesure où cela est compatible avec l’objectif expérimental de la procédure, on pourra établir une liste de critères anticipés (signes non visibles à l’examen clinique mais mesurables, manifestations transitoires ou discrètes) définissant l’atteinte du point limite adapté et permettant d’intervenir précocement sans nécessairement atteindre les points limites expérimentaux, ou d’interrompre une PE en cas de situation délétère non prévue pour l’animal.

Le choix d’un point limite adapté permet donc de borner le cadre d’une PE en mettant en balance la dégradation potentielle de l’état général d’un animal et l’intérêt de poursuivre son utilisation dans la procédure. En effet, au-delà

---

[18] Coenraad F.M. Hendriksen and David B. Morton (editors), Humane Endpoints in Animal Experiments for Biomedical Research – Proceedings of the International Conference, 22-25 November 1998, Zeist, The Netherlands, The Royal Society of Medicine Press Limited, United Kingdom, 1999. Dans https://www.humane-endpoints.info/fr/quappelle-t-on-points-limites

[19] On met ici en regard les points limites « éthiques », définis pour le bon traitement des animaux (Humane end-points), avec les points limites expérimentaux (Study end-points) définis pour les besoins des travaux scientifiques. La mise en œuvre des points limites anticipés vise à atteindre une juste balance lors de la mise en œuvre des procédures expérimentales.

de considérations réglementaires et éthiques, la dégradation de l'état général d'un animal perturbe les constantes biologiques dont l'analyse est indispensable aux fins de l'étude. Il n'est alors plus nécessaire – voire inutile, donc inacceptable – d'utiliser plus longtemps un tel animal et il y a donc lieu d'intervenir pour soulager son état de douleur ou de souffrance.

Selon les modèles animaux et l'objectif de chaque PE, le rédacteur de DAP pourra proposer une prise en charge graduelle des animaux adaptée à l'évolution de leur état général d'une part et aux besoins de son projet d'autre part, comme indiqué plus loin dans ce guide.

2.9 Procédure expérimentale

Le CRPM (Art. R214-89) définit la procédure expérimentale (PE) : il s'agit d'une utilisation, invasive ou non, d'un animal à des fins expérimentales ou à d'autres fins scientifiques ou à des fins éducatives, ou d'une intervention destinée ou de nature à aboutir à la naissance ou à l'éclosion d'un animal ou à la création et à la conservation d'une lignée d'animaux génétiquement modifiés, dès lors que cette utilisation ou cette intervention sont susceptibles de causer à cet animal une douleur, une souffrance, une angoisse ou des dommages durables équivalents ou supérieurs à ceux causés par l'introduction d'une aiguille effectuée conformément aux bonnes pratiques vétérinaires.

Il est également précisé que la mise à mort d'animaux à la seule fin d'utiliser leurs organes ou tissus, selon une méthode définie par arrêté conjoint du ministre chargé de l'agriculture et du ministre chargé de la recherche, n'est pas considérée comme une PE, à la condition expresse que l'animal soit mort au moment du prélèvement.

L'utilisation en question peut consister en un ou plusieurs actes réalisés sur des animaux pour atteindre l'objectif visé, l'ensemble de ces actes ou gestes constituant la procédure expérimentale en question.

Une procédure expérimentale fait partie d'un projet qui répondra à un objectif scientifique, éducatif ou réglementaire.

Il est demandé de présenter des projets en individualisant chaque procédure expérimentale dans la DAP, sans pour autant décliner autant de procédures expérimentales que d'actes expérimentaux.

Cela permet d'aborder de manière cohérente et homogène la classification du degré de gravité des procédures expérimentales, en cohérence avec les recommandations européennes[20] et de faciliter par ailleurs les décomptes statistiques.

Exemples de PE expérimentales :

- l'administration de substances et le suivi de leurs effets ;
- une intervention chirurgicale visant à poser un implant et l'évaluation de son efficacité ;
- la génération d'une lignée d'animaux génétiquement modifiés (qui peut impliquer plusieurs actes tels que superovulation, micro-injection, chirurgie pour la réimplantation des embryons modifiés) ou son phénotypage.

2.10 Projet

Un projet est un programme de travail répondant à un objectif scientifique défini, utilisant un ou plusieurs modèles animaux et impliquant une ou plusieurs procédures expérimentales.

Il peut s'agir de projets de recherche fondamentale, d'essais à visée règlementaire dans le cadre du développement ou du contrôle de produits de santé ou de projets à but pédagogique, qui reflètent la très grande variabilité des sujets de recherche ou de production.

Les programmes scientifiques peuvent inclure des modèles in silico ou in vitro, des approches biochimiques ou de biologie moléculaire, des approches épidémiologiques et des essais cliniques. Selon le CRPM, c'est seulement la partie de ces programmes qui concerne l'utilisation d'animaux vivants et sensibles dans des procédures expérimentales (selon la définition ci-dessus) qui est soumise à autorisation. Cette partie constitue un (ou éventuellement plusieurs) projet(s) qui fera(-ont) l'objet d'autant de demandes d'autorisation.

Quel que soit le nombre d'animaux, quelle que soit la durée, qu'il s'agisse d'une première étude exploratoire ou d'une mise au point, voire d'un complément non prévu à un projet autorisé, toute utilisation d'animaux dans une procédure expérimentale doit être couverte par une autorisation de projet.

[20] *https://ec.europa.eu/environment/chemicals/lab_animals/pdf/guidance/severity/fr.pdf*

2.11 Règle des 3R : Remplacement, Réduction et Raffinement

Le principe des 3R (« Replace, Reduce and Refine »), exposé en 1959 par Russel et Burch[21] est au cœur de la règlementation en vigueur en Europe depuis l'application de la Directive 2010/63/UE.

Son respect est une exigence règlementaire :

- Remplacement : Mise en œuvre des moyens reconnus qui évitent le recours aux animaux vivants pour atteindre l'objectif scientifique ;
- Réduction : Lorsque l'utilisation d'animaux vivants est nécessaire, limitation du nombre d'animaux utilisés tout en permettant l'obtention de résultats significatifs d'un point de vue statistique ;
- Raffinement : Réduction des contraintes imposées aux animaux (douleur, souffrance, angoisse ou dommages durables) par l'utilisation de moyens appropriés, par exemple : utilisation de médicaments antalgiques, analgésiques ou anesthésiques, adaptation de l'environnement des animaux (enrichissement), habituation aux conditions de la PE.

A l'instar des 3R, l'Expertise Collective INRA « Douleurs animales » a proposé une démarche pratique pour la gestion des douleurs animales, les « 3S », basée sur la **suppression** des sources de douleur chaque fois que cela est possible, en remettant en cause les pratiques, la **substitution** d'une pratique douloureuse par une autre qui l'est moins, le **soulagement** des douleurs inévitables par l'analgésie et le soin[22].

Le respect du principe des 3R est un élément clé de la licéité d'une PE (Art. R.214-105 du CRPM). Les 3R sont explicités par le rédacteur dans la DAP et ensuite évalués par le CEEA. Ils sont également reportés dans le résumé non technique du projet. Enfin, leur mise en œuvre est accompagnée par la SBEA pendant la réalisation du projet et vérifiée par les autorités lors des inspections.

---

[21] Russell, W.M.S. and Burch, R.L., (1959). The Principles of Humane Experimental Technique, Methuen, London. ISBN 0900767782

[21] https://www.cambridge.org/core/journals/animal/article/minimising-pain-in-farm-animals-the-3s-approach-suppress-substitute-soothe/66BACAC7C4B9A9B7AD0EAED232DDB258

« **Évaluation éthique »** est une expression consacrée par les textes français en référence à « l'évaluation très complète des projets, compte tenu de considérations éthiques dans l'utilisation des animaux » requise par la Directive 2010/63/UE dans laquelle elle « forme l'élément central de la procédure d'autorisation des projets ». Elle se décline dans l'article 4 de l'arrêté du 1er février 2013 relatif à l'évaluation éthique et à l'autorisation des projets impliquant l'utilisation d'animaux dans des PE qui vient préciser les termes du code rural et de la pêche maritime (CRPM, articles R.214-87 et suivants).

L'évaluation de projet vise à garantir la prise en compte des contraintes imposées aux animaux et à garantir l'application des principes de remplacement, de réduction et de raffinement. **Sa portée éthique**, et de fait subjective, s'inscrit dans une **analyse comparative des dommages et avantages du projet**, visant à apprécier si le dommage infligé aux animaux en termes de souffrance, de douleur et d'angoisse est justifié eu égard aux résultats escomptés, et pourra, en définitive, bénéficier aux êtres humains, aux animaux ou à l'environnement.

L'évaluation éthique a une valeur intrinsèque au regard des principes qui la guident et elle se concrétise in fine par un avis qui est le résultat des débats du comité d'éthique en expérimentation animale [cf. § 2.5].

Le CEEA est l'autorité compétente désignée dans les textes pour mener à bien l'évaluation des projets. Son avis est indispensable au ministère, autorité compétente pour la décision d'autorisation du projet.

Le CEEA s'engage à respecter les textes réglementaires et les principes de la Charte nationale, notamment en matière de composition, d'indépendance, d'impartialité et de respect de la confidentialité des informations, et établit son propre règlement intérieur.

Le délai de réponse du CEEA pour délivrer son avis au ministère est de 7 semaines à compter de la date de soumission du dossier de demande d'autorisation de projet (appelé dans ce document « DAP ») auprès du ministère chargé de la recherche, la transmission du dossier reçu par le ministère au CEEA étant instantanée suite au dépôt du dossier par le demandeur sur la plateforme APAFiS.

3.1 Garanties apportées par l'évaluation éthique

Les CEEA apportent quatre garanties :

- la garantie de respect de la **confidentialité** (des informations fournies dans les dossiers) ;
- la garantie de **l'indépendance**, un membre de CEEA formulant librement ses propos, sans influence hiérarchique ;
- la garantie de **l'impartialité**, les membres s'engageant à l'absence de conflit d'intérêts ;
- la garantie de la **compétence**, du fait de la composition des CEEA où plusieurs catégories de membres compétents sont représentées (concepteur de projet, applicateur de PE, soigneur des animaux, vétérinaire), mais aussi représentant de la société civile, dans un contexte de représentation pluridisciplinaire.

Le ministère vérifie que la compétence des comités est assurée en examinant leur composition au moment de l'enregistrement. Concernant la confidentialité, l'indépendance et l'impartialité, chaque membre de CEEA signe un engagement personnel que le comité transmet également au ministère.

Le CEEA veille quant à lui à ce que toutes les catégories de membres s'expriment au cours de l'évaluation. Le CEEA veillera également à ce qu'un éventuel expert extérieur, auquel il peut faire appel ponctuellement, applique les mêmes engagements moraux, garants de l'évaluation éthique.

L'évaluation éthique permet de **vérifier** que le projet satisfait aux critères généraux suivants :

- Le projet est justifié du point de vue scientifique, ou éducatif, ou requis par la loi ;
- Les objectifs du projet justifient l'utilisation d'animaux ;
- Le projet est conçu pour permettre le déroulement des procédures expérimentales dans les conditions les plus respectueuses de l'animal et de l'environnement, grâce à la mise en œuvre de méthodes actualisées sur la base des meilleures pratiques.

Dans ces conditions, **éthique et science se rejoignent** sur les trois notions suivantes :

- les objectifs sont clairement identifiés ;
- les moyens mis en œuvre sont alignés avec les objectifs et permettent l'obtention de résultats tangibles ;
- le recours aux animaux vivants est justifié et est réduit au minimum nécessaire pour l'obtention de ces résultats.

Comme détaillé plus loin dans ce guide, le CEEA élabore son avis sur la base d'une analyse comparative entre les avantages (bénéfices attendus) du projet et les dommages qu'il impliquera en relation avec les espèces animales utilisées et leur nombre, les contraintes auxquelles les animaux seront soumis (angoisse, douleur, souffrance) et leur degré de gravité.

Les membres du CEEA doivent donc trouver, dans les documents qui leur sont soumis par le demandeur d'autorisation de projet, toutes les informations dont ils ont besoin pour émettre un avis éclairé, par exemple, une bibliographie et des référentiels reconnus.

3.2 Portée et valeur de l'avis du CEEA

L'avis du CEEA prend sa valeur et présente une portée éthique grâce au processus d'évaluation qui permet d'élaborer cet avis.

Quel que soit le mode de fonctionnement du CEEA, les évaluateurs (représentés par le président, le vice-président ou adjoint ou des rapporteurs selon les cas), suite à une lecture attentive du projet, engagent une discussion avec le rédacteur de DAP. A l'occasion de ces échanges, ils demandent des précisions pour affiner leur compréhension du projet. Se préservant d'interpréter les éléments absents ou qui manqueraient de clarté, le CEEA demande toutes les informations complémentaires ou explications nécessaires. Au besoin, il consulte des experts du domaine en dehors de ses membres.

La participation du membre non spécialisé est tout aussi importante que celle des membres compétents en matière d'utilisation d'animaux à des fins scientifiques, car elle permet en tout premier lieu l'expression de la société civile, indispensable à l'analyse comparative des dommages et avantages du projet, et à la promotion

Retour au sommaire

des principes éthiques. Le membre non spécialisé pose souvent les questions fondamentales que pourrait se poser la société civile comme celles relatives à l'utilité du projet (« A quoi ça sert ? »). Son regard permet aussi de s'assurer que le résumé non technique du projet est rédigé de manière vulgarisée et donc compréhensible par tous.

A l'issue des échanges et délibérations mettant en jeu les cinq compétences requises dans sa composition, le CEEA émet son avis sur la version finale du projet issue des amendements éventuels. Cet avis est transmis au ministère via la plateforme APAFiS et le demandeur en est informé via cette même plateforme. A noter, le CEEA peut utiliser la plateforme pour échanger avec le rédacteur de DAP lors de l'évaluation éthique. Si ce n'est pas le cas, il devra conserver tous les échanges avec le rédacteur de DAP, en cas de besoin ultérieur.

L'avis du CEEA peut être :

- soit **favorable**, éventuellement avec proposition de reclassement du degré de gravité des PE, avec la mention d'une appréciation rétrospective et/ou l'avis du comité sur la réutilisation de tout ou partie des animaux du projet,
- soit **défavorable**. Dans ce cas, le comité doit documenter dans l'avis les raisons de sa décision.

3.3 Relation avec l'évaluation scientifique

L'évaluation éthique se déroule indépendamment de l'évaluation scientifique du projet et le CEEA ne se substitue pas à un comité d'évaluation scientifique, pédagogique ou une agence réglementaire.

Le rôle du CEEA est de s'assurer que la justification du projet a été considérée comme pertinente par une instance compétente au regard de sa finalité scientifique, réglementaire ou pédagogique. Par exemple dans le cas d'un projet à finalité scientifique, le comité peut s'appuyer sur la mention d'une évaluation scientifique par une instance d'évaluation de la recherche publique, par le conseil scientifique d'une entreprise privée ou sa direction. Il peut également prendre en compte une évaluation scientifique menée dans le cadre d'une demande de financement (agence de financement de la recherche publique, association reconnue d'utilité publique, fondation…).

3.4 Participer à la conformité règlementaire des projets

Le ministère chargé de la recherche intervient comme autorité compétente pour l'autorisation de projets sur avis du CEEA, autorité compétente pour assurer la mission d'évaluation éthique (Art. R. 214-119 du CRPM).

Par ailleurs, ce sont les services du ministère chargé de l'agriculture qui ont en charge le respect de la conformité réglementaire en matière de protection animale et ainsi la conformité de l'utilisation des animaux avec les projets autorisés. Ils sont notamment autorité compétente pour l'agrément des établissements.

Du fait de ses échanges avec le rédacteur de DAP, le CEEA participe en première ligne à la **vérification de la complétude du dossier.** Il contribue ainsi à la conformité administrative des demandes d'autorisation même si cette mission est attribuée dans les textes au ministère. Au cours de l'évaluation, le CEEA peut relever certains points qui indiqueraient un défaut de conformité réglementaire (par exemple, au sujet des objets d'utilisation, de l'origine des animaux, du bon usage des médicaments ou encore du résumé non technique). Ne pouvant considérer comme éthique une utilisation qui se situerait hors du cadre de la réglementation, le CEEA attirera l'attention du demandeur sur ces points de non-conformité, sans pour autant se substituer aux autorités compétentes en la matière.

En tout état de cause, c'est le ministère chargé de la recherche qui s'assure de la complétude et de la conformité de la dernière version du dossier suite à la réception de l'avis favorable et avant l'autorisation du projet.

4.1 Cadre réglementaire et méthodologie

Cette partie du Guide a pour objectif d'aider les membres d'un CEEA à construire un avis consolidé, fondé sur une analyse objective et systématique de chaque projet.

Ce chapitre répond aux dispositions du CRPM (article R214-119 et suivants) et de l'arrêté (article 4). L'évaluateur devra toutefois avoir connaissance de l'ensemble du corpus règlementaire : directive, code rural et les différents arrêtés d'application, car une disposition juridique prise de manière isolée peut mener à des erreurs d'interprétation.

Comme indiqué au § 3.1, l'objectif de l'évaluation éthique est d'analyser la balance entre avantages et dommages du projet en répondant à trois questions générales :

1. Le projet est-il justifié du point de vue scientifique, éducatif ou est-il requis par la loi ?
2. Les objectifs du projet justifient-ils l'utilisation d'animaux ?
3. Le projet est-il conçu pour permettre le déroulement des PE dans les conditions les plus respectueuses de l'animal et de l'environnement ?

Pour répondre à ces 3 questions, l'arrêté identifie de manière plus précise les champs d'analyse du CEEA :

a. Une évaluation des objectifs du projet, des avantages scientifiques attendus ou de sa valeur éducative ;

b. Une appréciation de la conformité du projet avec les exigences de remplacement, de réduction et de raffinement ;

c. Une appréciation de la classification des PE selon leur degré de gravité ;

d. Une analyse comparative des dommages et des avantages du projet ;

e. Une appréciation des éléments visés aux articles R. 214-90 à R. 214-95, R. 214-98, R. 214-105 à R. 214-109 et R. 214-113 du CRPM (il s'agit là d'éléments pour lesquels une justification est requise, permettant au CEEA de mener à bien l'évaluation éthique et au ministère d'accorder des dérogations le cas échéant). Ces points spécifiques sont abordés par la suite [cf. § 4.6] ;

f. La détermination de la nécessité de procéder à une appréciation rétrospective du projet et le moment auquel celle-ci doit intervenir (le CRPM la demandant à l'issue de la réalisation du projet).

Afin de mener à bien cette démarche, l'analyse de la DAP se fait en plusieurs étapes.

Etape 1 : Evaluation du projet dans son ensemble

- Prendre connaissance du titre du projet, de sa justification et de ses objectifs, ce qui permet d'envisager les bénéfices attendus ;
- Juger de la nécessité incontournable d'utiliser des animaux vivants pour atteindre les objectifs du projet et évaluer les éléments fournis en matière de remplacement ou d'obtention de résultats préalables ou complémentaires par d'autres voies (autre modèle moins sensible, méthode substitutive) ;
- Prendre connaissance du ou des modèles animaux utilisés, évaluer la prise en compte de la réduction et du raffinement dans la conception du projet ;
- Évaluer l'adéquation entre les moyens mis en œuvre et l'objectif du projet.

Etape 2 : Evaluation de chaque procédure expérimentale

- Examiner les techniques et les méthodes mises en jeu à l'aune des référentiels disponibles ; S'assurer que suffisamment de détails sont fournis pour une bonne appréciation du déroulement de chaque procédure et de son impact sur l'animal ;
- Analyser les contraintes (douleur, souffrance, angoisse ou dommages durables) auxquelles les animaux sont soumis et les moyens mis en œuvre pour les diminuer : médicaments antalgiques, anesthésiques, habituation ou mesures spécifiques d'enrichissement de l'hébergement. Examiner les points limites anticipés et les méthodes de mise à mort ;
- Appréhender le degré de gravité associé à chaque PE et envisager le reclassement (cf. annexe de l'arrêté pour des exemples illustratifs des classements de procédures) ;
- Analyser le besoin d'une appréciation rétrospective à l'issue du projet ;
- Donner un avis sur la réutilisation dans les cas où cela est nécessaire (cf. article R.214-113 du CRPM).

Etape 3 : Analyse comparative des dommages et avantages du projet

S'appuyant sur les éléments précédents, le CEEA émet un avis sur le projet qui lui est présenté. Il évalue les bénéfices attendus au regard des contraintes imposées aux animaux. Si les bénéfices l'emportent sur les contraintes, l'avis est favorable ; si les contraintes l'emportent sur les bénéfices, l'avis est défavorable.

4.2 Documents nécessaires à l'évaluation

> Notons que la **réflexion éthique** inclut **l'évaluation** éthique, mais également la phase de **rédaction** de la DAP, rendue obligatoire par les items du formulaire à remplir.

Pour rédiger sa DAP, le rédacteur de DAP utilise le formulaire fourni par le ministère chargé de la recherche en s'appuyant sur les bulles d'aide et une notice pour remplir les différents items du formulaire.

Le document de DAP disponible sur la plateforme APAFiS (en version .apafis), crypté par le demandeur lors de la soumission pour assurer la sécurité des informations, est décrypté par le CEEA grâce à l'application « comité d'éthique » du système APAFiS. Le fichier lisible (.pdf) peut être transmis aux évaluateurs et contiendra les informations à expertiser.

Outre la DAP, le CEEA a à sa disposition des documents de référence pour son évaluation, classés ici par ordre d'importance :

- les textes règlementaires : Art. R.214-87 et suivants du CRPM et les arrêtés d'application du 1er février 2013, ainsi que leurs annexes (annexe de l'arrêté pour la classification des procédures, annexe de l'arrêté sur l'agrément des établissements pour les méthodes de mise à mort) ;
- la Charte nationale portant sur l'éthique de l'expérimentation animale, ainsi que les recommandations du CNREEA sur des sujets spécifiques et les guides de bonnes pratiques dont le CNREEA a conduit l'élaboration ;
- les documents émanant du ministère chargé de la recherche : notices disponibles sur la plateforme APAFiS, mémorandums « avis » et « dossiers » transmis par le ministère aux CEEA, qui viennent expliciter la règlementation ;
- les informations émanant du ministère chargé de l'agriculture, concernant le bien-être animal, et plus particulièrement en matière de réglementation et de bonnes pratiques de l'usage des médicaments vétérinaires ;
- tout autre élément d'information transmis lors des colloques des présidents de CEEA, ainsi que les « documents de consensus » publiés par la Commission Européenne pour éclairer les Etats Membres sur l'application de la règlementation (par exemple pour l'évaluation du degré de gravité des PE).

Pourront également être pris en compte :

- les recommandations citées dans les références bibliographiques de ce document et tout référentiel technique dans les domaines de l'anesthésie vétérinaire, la gestion de la douleur, l'évaluation de la souffrance et des points limites anticipés, etc. ;
- les documents issus de travaux d'associations de professionnels françaises : AFSTAL[23] (articles de la revue STAL), GIRCOR[24] (guides) et OPAL[25] (Bref'Opal) ou européennes telles que FELASA[26] (recommandations issues de groupes de travail).

Les évaluateurs peuvent demander, pour leur bonne compréhension du projet, des documents complémentaires, par exemple :

- un chronogramme présentant schématiquement les étapes du projet et/ou des PE ;
- des publications scientifiques décrivant une expérience dans le modèle présenté ;
- un schéma, une figure qui permet d'illustrer un acte de chirurgie ou une geste complexe ;
- les modes opératoires cités dans la DAP, en relation avec les PE ;
- des détails sur l'organisation interne, lorsque les projets font appel à des compétences pluridisciplinaires ou des personnels sur plusieurs établissements.

L'avis du Comité est d'autant plus pertinent et transparent vis-à-vis de la société qu'il s'appuie sur des référentiels à destination de la communauté scientifique reconnus et partagés.

A noter : lorsqu'il réalise une évaluation éthique en dehors du champ réglementaire de l'autorisation de projet, le CEEA utilisera un autre formulaire que le formulaire réglementaire de demande d'autorisation de projet et l'évaluation se fera en dehors de la plateforme pour éviter tout problème lié à la sécurité des informations.

---

[23] *www.afstal.com*
[24] *www.recherche-animale.org*
[25] *http://opal-association.org/*
[26] *www.felasa.eu*

4.3 Evaluation des objectifs et de la pertinence du projet

4.3.1 Méthodes et principes d'évaluation de l'objectif et de la pertinence du projet

À l'exclusion de la pertinence scientifique de la question posée [cf. § 3.3], l'évaluation portera sur la pertinence du projet sur la base des avantages attendus, de l'intérêt et la nécessité d'utiliser des animaux, et de la démarche proposée pour atteindre les objectifs, sur la base de connaissances actualisées et de l'état de l'art, permettant d'éviter tout double emploi injustifié de PE et en tenant compte des contraintes auxquelles sont soumis les animaux.

4.3.1.1 Evaluation des avantages attendus

Dans sa démarche, le CEEA n'évalue pas la valeur intrinsèque de la question scientifique posée, de l'objectif pédagogique ou de l'exigence règlementaire. Cependant les membres du comité ne pourront évaluer les aspects éthiques du projet que si l'information fournie est complète, claire et étayée de références bibliographiques ou réglementaires. En outre, elle doit être compréhensible par tous.

L'évaluateur répertorie les avantages attendus en cherchant, dans les éléments présentés, des réponses satisfaisantes aux questions suivantes :

- Quels seront les bénéfices issus du projet ?
- Quels seront les bénéficiaires des résultats du projet (homme, animal, environnement)[27] ?
- A quelle échéance les résultats de ce projet seront-ils accessibles ou applicables (court terme, moyen terme, long terme) et à quelle échelle ?

L'évaluation du bénéfice attendu en recherche fondamentale est plus difficile, car souvent lointaine. Pour autant, l'avancée des connaissances est considérée comme un bénéfice en soi, même en l'absence d'application directe connue immédiate. Elle présente possiblement un intérêt à long terme.

A l'inverse, dans le cas des projets à visée réglementaire ou éducative, un bénéfice concret, voire immédiat, est attendu et doit donc être décrit le plus précisément possible, en s'appuyant, le cas échéant, sur le recul déjà obtenu lors des mises en œuvre précédentes des PE.

---

[27] Le cas échéant, les résultats peuvent être bénéfiques pour d'autres projets en améliorant une technique ou en faisant progresser la connaissance sur un modèle, sur la gestion du bien-être animal ou les 3R.

4.3.1.2 Evaluation de l'intérêt et de la nécessité incontournable d'utiliser des animaux vivants dans le but décrit

Ici se pose la question des méthodes de remplacement pour laquelle il faut s'assurer du caractère incontournable du recours aux animaux vivants (voir § 3.4.1). Il y a également lieu de garantir l'absence de double emploi injustifié de PE et donc d'animaux.

Le choix d'un modèle animal vivant et donc sensible doit avant tout répondre à la double question : « Pourquoi un modèle non animal ou animal non vivant ne convient-il pas ? » et, par conséquent, « En quoi un modèle animal permet de répondre à la question posée ? »

On s'attache à évaluer, parmi les éléments fournis, autant ceux qui montrent les limites des méthodes sans animaux que les apports des modèles animaux, ainsi que la complémentarité entre les modèles.

Par exemple :

- En enseignement et formation professionnelle : pour l'objectif pédagogique identifié, peut-on remplacer l'animal vivant par des vidéos ou des simulations informatiques ? Peut-on utiliser des cadavres d'animaux, des pièces anatomiques de boucherie ou encore d'autres matériaux inertes ?
- En recherche : pour l'objectif scientifique identifié, existe-t-il des méthodes équivalentes utilisant des espèces animales non concernées par la Directive 2010/63/EU (invertébrés hors céphalopodes) ou des stades de développement précoces dont la sensibilité n'est pas reconnue (larves précoces de poissons) ?

Il conviendra de se référer aux bases documentaires régulièrement mises à jour comme celles de l'ECVAM[28].

4.3.1.3 Possibilité d'atteindre les objectifs

Le Comité d'Ethique s'assurera de l'adéquation entre les objectifs visés et les méthodes envisagées.

[28] *https://ec.europa.eu/jrc/en/eurl/ecvam*

L'évaluation se fondera sur les éléments disponibles :

- Choix de la (ou des) espèce(s) animale(s) ;
- Choix du nombre d'animaux, du sexe et du stade de développement ;
- Approche statistique, constitution des lots, modalités de recueil et d'analyse des résultats ;
- Description du projet avec l'articulation des procédures et des PE ;
- Eventuellement, le synopsis et les chronogrammes annexés au projet.

Sur la base de l'ensemble de ces éléments, le CEEA peut mieux appréhender l'ampleur du projet et juger si sa mise en œuvre lui semble faisable ou s'il paraît trop ambitieux, en mettant en regard l'objectif fixé et les moyens mis en œuvre.

Concernant le choix de l'espèce animale, l'évaluateur doit pouvoir disposer d'un argumentaire s'appuyant sur la littérature scientifique disponible sur ce modèle et des éléments d'expérience apportés par le rédacteur de DAP. La justification par rapport à d'autres modèles animaux est obligatoire dans le cas des primates.

4.3.2 Diffusion et utilisation des résultats

En règle générale, la publication ou la diffusion des résultats expérimentaux, négatifs ou positifs, contribue à la pertinence des projets par la valorisation qui en est faite. C'est une valeur largement promue par la Commission Européenne. Les modalités de cette diffusion sont prises en compte dans l'évaluation du bénéfice attendu.

Parfois, un rédacteur de DAP indiquera au CEEA, en amont, qu'il ne compte pas publier, mais que les résultats de son étude contribueront à un autre projet ou au développement d'une nouvelle thématique scientifique.

Le dépôt de brevet est également un mode de valorisation des résultats.

Toutefois, dans l'industrie chimique ou pharmaceutique, la diffusion de résultats est limitée par les exigences de confidentialité.

Ces informations se retrouveront dans la section relative à la présentation et à la description du projet dans le formulaire de DAP.

4.4 Evaluer la conformité du projet avec les exigences de remplacement, de réduction et de raffinement

Estimer la prise en compte des principes de remplacement, raffinement et réduction permet d'évaluer la façon dont le rédacteur de DAP a conçu son projet en atténuant les dommages attendus, compte tenu de son objectif. Ces dommages sont à entendre au sens du nombre d'animaux et des contraintes auxquelles ils doivent être soumis.

3R et approches alternatives

Au sens où l'entend la Commission Européenne[29], le terme « alternatifs/ves » couvre tous les essais, tests et outils, toutes les méthodes, techniques, stratégies et approches qui contribuent à la mise en application concrète des 3R. C'est-à-dire :

- Obtenir les informations requises sans recourir aux animaux vivants (Remplacement) ;
- Réduire le nombre d'animaux tout en obtenant le même niveau d'information (Réduction) ;
- Optimiser l'utilisation des animaux vivants afin d'engendrer le moins de douleur, détresse ou angoisse ou afin d'améliorer le bien-être des animaux (Raffinement).

Les approches alternatives offrent des opportunités d'avancées en matière de 3R, mais contribuent tout autant au développement d'outils scientifiques plus précis et prédictifs.

L'expression « approches (ou méthodes) alternatives », lorsqu'elle est utilisée dans ce document, couvre l'ensemble des 3R. Lorsqu'il s'agit du seul « R » de remplacement, on utilise l'expression « substitution » ou « méthode substitutive ».

Rappelons que la stratégie générale de prise en compte de ces éléments doit être précisée dans la DAP, détaillée dans les PE et retranscrite de manière vulgarisée par le rédacteur de DAP dans le résumé non technique du projet.

---

[28] *https://ec.europa.eu/environment/chemicals/lab_animals/3r/alternative_en.htm*

4.4.1 Remplacement

Les renseignements concernant le remplacement sont présents dans deux parties de la saisine :

- Justification du recours à l'animal
- Description du Projet

Les programmes de recherche font appel à des approches mixtes qui combinent les modèles animaux et les modèles sans animaux. Ces informations se retrouvent de manière synthétique dans l'item « Description du projet ».

En effet, les informations présentées par le rédacteur de DAP doivent permettre au CEEA de comprendre la démarche globale et la façon dont l'animal s'insère dans le processus de recherche et de **s'assurer que toutes les options sans animaux ont été envisagées et écartées de manière argumentée avant de recourir à l'animal.**

Cette étape est primordiale, tant dans la réflexion du rédacteur de DAP que dans le travail des évaluateurs, car elle détermine le besoin incontournable de recourir à des animaux vivants et donc d'introduire une DAP dans laquelle sont décrites l'ensemble des autres éléments abordés dans ce guide.

On considère comme du **remplacement absolu** le fait de recourir à des méthodes artificielles (non biologiques telles que la simulation informatique, le recours à des bases de données), à des matériaux inertes, à des lignées cellulaires établies, à des animaux morts ou encore à des animaux vivants d'une espèce qui n'est pas concernée par les articles R. 214-87 et suivants du CRPM.

Le fait de recourir à des modèles biologiques in vitro réalisés à partir de cellules ou tissus prélevés sur des animaux d'espèces concernées par le CRPM (Art. R. 214-87 et suivants) constitue un **remplacement relatif** et peut contribuer aussi à l'objectif de réduction.

Enfin, le fait de recourir à une espèce concernée par le CRPM (Art. R. 214-87 et suivants), mais moins susceptible de ressentir les contraintes dans les conditions de la procédure, appelée « moins sensible » par esprit de vulgarisation, contribue à un **raffinement** de l'approche expérimentale.

4.4.2 Réduction

Les éléments concernant le nombre d'animaux seront étudiés avec attention. Le Comité s'attachera à vérifier que le nombre d'animaux prévu n'est ni trop élevé pour respecter le devoir de réduction, ni trop faible afin d'obtenir des données exploitables.

Pour s'assurer que le nombre d'animaux utilisé sera optimal, l'évaluateur vérifie que le plan d'expérience prévoit qu'il a été calculé en tenant compte des données qui vont être mesurées. Selon leur nature, et en particulier leur variabilité, et la puissance statistique recherchée, les tests statistiques qui sont envisagés permettent de déduire le nombre d'animaux nécessaire (c'est ce qu'on appelle le calcul de puissance statistique). Il est impératif que le rédacteur de DAP indique aussi une variable principale qui permettra de définir clairement le succès ou non du projet. Ceci permet des calculs rationnels du nombre et une analyse dommage-avantage claire et partagée.

Dans certains cas, l'effectif d'animaux est imposé par des contraintes réglementaires ou d'autres impératifs expérimentaux.

Dans tous les cas, le Comité cherchera des éléments de justification auprès du rédacteur de DAP et appuiera si possible son avis sur les conseils d'un statisticien.

4.4.3 Raffinement

Pour chaque contrainte identifiée dans un projet, une mesure compensatrice permettant de l'atténuer doit être envisagée et proposée par le rédacteur de DAP. Dans le cas où cela n'est pas possible, des justifications solides doivent être apportées.

La description détaillée des PE permet d'identifier les actions de raffinement qui contribuent à diminuer, voire supprimer, les contraintes. Ces renseignements sont donnés dans le chapitre traitant de l'anesthésie, de l'analgésie et de la réduction de la souffrance. En amont du détail indiqué pour chaque procédure, le rédacteur de DAP doit indiquer plus largement sa stratégie afin de réduire au minimum la douleur, la souffrance et l'angoisse infligées aux animaux.

L'évaluateur doit pouvoir estimer les contraintes prévues ou potentielles imposées à tous les animaux de leur arrivée et jusqu'à la fin de la procédure, identifier les étapes critiques et vérifier qu'il n'y a pas de lacune en matière de raffinement.

Le CEEA peut identifier des défauts dans la mise en œuvre du raffinement sans justification suffisante :

- l'isolement d'animaux sociaux sans mesure compensatrice appropriée, d'autant plus si c'est sur une longue période ;
- un protocole d'anesthésie ou d'euthanasie qui ne correspond pas à l'état de l'art au vu des interventions prévues ;
- une analgésie absente ou inadaptée tout au long de la procédure ;
- l'absence de mention de point limite anticipé ou un point limite inadapté à la procédure considérée, par exemple, non spécifique ou trop tardif, ou encore des mesures insuffisantes d'allègement des contraintes.

Dans ces conditions, d'une part, l'obligation de raffinement n'est pas remplie et, d'autre part, il est probable que l'analyse du CEEA aboutira à du dommage trop élevé au regard de l'avantage escompté. Il n'est donc pas possible de délivrer un avis favorable en l'état.

Le CEEA doit alors conseiller le rédacteur de DAP afin que :

- les méthodes soient raffinées conformément à l'état de l'art. C'est en effet le rôle du CEEA de conseiller les rédacteurs de DAP sur la base d'une veille bibliographique sur les méthodes d'anesthésie, d'analgésie, d'administration, de prélèvement ou sur d'autres techniques expérimentales ;
- une justification scientifique claire et solide soit apportée à chaque fois qu'une contrainte n'est pas compensable par une méthode de raffinement adaptée.

4.5 Appréciation de la classification des procédures expérimentales selon leur degré de gravité

Le rédacteur de DAP propose **un degré de gravité pour chacune des PE constituant le projet.** Il doit choisir entre quatre degrés de gravité : léger, modéré, sévère ou sans réveil.

Pour statuer entre les degrés croissants (léger, modéré ou sévère), on tient compte de la nature, de la fréquence, de l'intensité, de la durée des contraintes et des dommages durables imposés aux animaux sur la totalité de la durée de la PE. Des facteurs de modulation liés, entre autres, aux espèces animales et à leur stade de développement, au cumul et au raffinement des techniques entrent également en considération.

Le degré de gravité retenu pour une procédure donnée de manière prospective est le plus élevé que pourrait subir un animal en tenant compte de toutes les contraintes attendues une fois l'ensemble des méthodes de raffinement appropriées mises en œuvre.

Le quatrième degré dit « sans réveil » correspond strictement à une procédure qui se déroule **entièrement sous anesthésie générale** à l'issue de laquelle l'animal est euthanasié sans réveil. On exclut de cette catégorie une procédure au cours de laquelle l'animal aurait subi, par exemple, des injections ou prélèvements ou encore une mise à jeun ou un isolement avant le début de l'anesthésie.

Le CEEA estime si le classement proposé par le rédacteur de DAP est adapté. L'évaluation doit se baser sur les documents de référence publiés par la Commission Européenne[30] ou par les groupes de travail ESLAV/ECLAM/FELASA[31].

Le cas échéant, le CEEA doit également tenir compte de l'expérience acquise lors de PE antérieures démontrant l'expertise de l'équipe de recherche.

---

[30] *Cadre d'évaluation de la gravité des procédures (https://ec.europa.eu/environment/chemicals/lab_animals/pdf/guidance/severity/fr.pdf)*

[31] *Classification and reporting of severity experienced by animals used in scientific procedures: FELASA/ECLAM/ESLAV Working Group report. D Smith et al. Lab Anim. 2018 Feb;52(1_suppl):5-57. https://doi.org/10.1177/0023677217744587*

Si le comité peut faire adopter des mesures compensatrices complémentaires, l'estimation du degré de gravité peut être revue à la baisse.

Il y a lieu de différencier l'évaluation du degré de gravité a priori de l'évaluation du degré de gravité réel.

La première s'applique à la procédure indépendamment des aléas de sa mise en œuvre. On part du principe que le personnel est qualifié et les techniques maîtrisées et on envisage la « situation la plus grave » dans ces conditions.

La deuxième s'applique à chaque animal, une fois la procédure terminée et sur la base des effets constatés.

L'évaluation de la gravité est donc une **notion dynamique** impliquant une estimation a priori, une mesure pendant le déroulement de la procédure et un enregistrement final pour chaque animal inclus dans la procédure expérimentale.

Le degré de gravité peut être difficile à estimer pour un modèle animal qui n'a jamais été mis en œuvre et ne ressemble à aucun modèle référencé dans la littérature. Dans ce cas, le CEEA peut donner un avis favorable sur la base d'un classement en modéré et demander une appréciation rétrospective du projet, qui inclura un rapport sur la gravité constatée des procédures en s'appuyant sur un accompagnement par la SBEA de l'établissement. Le CEEA pourra également proposer, dans le cadre de la promotion des principes éthiques, non règlementaire mais prévue par la Charte nationale, une appréciation rétrospective d'étape qui sera menée d'un commun accord avec le rédacteur de DAP.

4.6 Appréciation des éléments techniques obligatoires

Conformément à l'alinéa e de l'article 4 de l'Arrêté ministériel relatif à l'évaluation et à l'autorisation de projet, l'évaluation éthique comporte pour partie l'appréciation de plusieurs éléments techniques obligatoires (visés aux articles R. 214-90 à R. 214-95, R. 214-98, R. 214-105 à R. 214-109 et R. 214-113 du CRPM).

Pour ces points particuliers, le CEEA s'assurera que les justifications appropriées sont apportées par le rédacteur de DAP en cas de demande de dérogation à la règle.

4.6.1 Espèces animales concernées et origine des animaux

Les animaux des espèces énumérées dans l'arrêté sur la fourniture des animaux[32] utilisés ou destinés à être utilisés dans des PE doivent être élevés à cette fin et proviennent d'éleveurs ou de fournisseurs agréés (article. R.214-90). Des dérogations peuvent être accordées uniquement pour raisons scientifiques dans le cadre de l'autorisation de projet et le CEEA a en charge l'évaluation de ces raisons scientifiques.

Pour le recours aux animaux errants ou vivants à l'état sauvage (article R. 214-91), le recours aux animaux non domestiques non tenus en captivité (article R. 214-92) ou encore le recours à des animaux d'espèces menacées autres que primates (article R. 214-93), le CEEA analysera les éléments scientifiques qui justifient que l'objectif de la PE ne peut être atteint en utilisant des animaux élevés spécifiquement en vue d'une utilisation dans des PE.

Une attention particulière sera également portée au recours aux primates (article R. 214-94), détaillé au point suivant.

L'origine des animaux est règlementée strictement et pour certaines de ces dérogations, un avis complémentaire de la Commission nationale pour la protection des animaux à des fins scientifiques (anciennement Commission nationale de l'expérimentation animale) sera nécessaire (article R.214-91 concernant les animaux d'espèces domestiques errants ou vivant à l'état sauvage ou article R.214-94 sur les primates), voire un accord de la Commission européenne devra être obtenu dans certains cas (article R.214-94 sur les primates).

---

[32] Arrêté du 1er février 2013 fixant les conditions de fourniture de certaines espèces animales utilisées à des fins scientifiques aux établissements utilisateurs agréés disponible à : https://www.enseignementsup-recherche.gouv.fr/cid70597/l-utilisation-des-animaux-a-des-fins-scientifiques.html. Cette liste exclut les animaux de rente pour lesquels il n'y a pas de contrainte concernant leur origine.

4.6.2 *Utilisation de primates*

Les primates, dénommés aussi primates non humains, présentent des similitudes étroites et parfois uniques avec l'humain. Leur utilisation en recherche doit respecter des exigences réglementaires particulières.

Conformément aux articles R.214-94 et R.214-105 du CRPM, l'utilisation des primates n'est autorisée que pour les PE qui ont pour objet :

- La recherche translationnelle ou appliquée menée pour la prévention, la prophylaxie, le diagnostic ou le traitement de maladies, de mauvais états de santé ou d'autres anomalies ou de leurs effets chez l'homme, les animaux ou les plantes,
- La mise au point, la production ou les essais de qualité, d'efficacité et d'innocuité des médicaments.

Dans ces deux cas, uniquement pour la prévention, la prophylaxie, le diagnostic ou le traitement d'affections humaines invalidantes ou potentiellement mortelles.

- La recherche fondamentale,
- La recherche en vue de la conservation des espèces.

Et quand il existe des éléments scientifiques démontrant que la finalité de la PE ne peut être atteinte en utilisant d'autres espèces que celles appartenant à l'ordre des primates.

Ces restrictions sont plus fortes lorsqu'il s'agit d'espèces de primates dites protégées (énumérées à l'annexe A du règlement (CE) no 338/97 du Conseil du 9 décembre 1996 relatif à la protection des espèces de faune et de flore sauvages par le contrôle de leur commerce), puisque la recherche fondamentale est exclue des objets d'utilisation de ces animaux.

Si pour des motifs scientifiquement justifiables, la finalité de la PE ne peut être atteinte qu'en utilisant des primates à des fins autres que la prévention, la prophylaxie, le diagnostic ou le traitement d'affections invalidantes ou susceptibles d'être mortelles, le responsable de projet dépose une demande écrite auprès du ministère chargé de la recherche. La Commission européenne serait consultée par ce dernier et pourrait s'opposer à cette utilisation.

Enfin, l'utilisation de primates anthropoïdes (singes appartenant aux genres Gorilla, Pan et Pongo) est interdite sauf conditions exceptionnelles. Il s'agirait alors d'une utilisation dans des PE aux fins de la préservation de l'espèce ou du fait de l'apparition imprévue, chez l'homme, d'une affection clinique invalidante ou susceptible d'être mortelle. Là encore, la Commission européenne serait consultée par le ministère chargé de la recherche et pourrait s'opposer à cette utilisation.

L'évaluateur s'assure que l'argumentaire pour le choix de l'espèce est étayé par un argumentaire scientifique spécifique qui doit démontrer la pertinence du primate par rapport aux autres espèces pour répondre à la question posée. De fait, l'utilisation de primates s'inscrit en principe à la suite de travaux sur d'autres espèces animales chez lesquelles, par exemple, l'innocuité des substances testées aura été évaluée.

Tout projet utilisant des primates fera l'objet d'une appréciation rétrospective (Cf. article R.214-120 du CRPM).

4.6.3 Conditions d'hébergement et d'entretien des animaux

Les conditions d'hébergement et d'entretien des animaux sont indiquées à l'article R. 214-95 du CRPM. Une demande d'autorisation de projet ne décrit pas les modalités d'hébergement et de soins aux animaux standard (qui sont appréciées au cours du processus d'agrément de l'EU). Elles ne sont évoquées que lorsqu'une adaptation de la règle est envisagée pour la PE (un isolement, une mise à jeun), pouvant engendrer un impact négatif sur le bien-être des animaux.

Dans ce cas, on en trouve la description dans le détail de la PE dans les items suivants :

- « Indiquez le cas échéant les raisons scientifiques justifiant une dérogation aux conditions d'hébergement des animaux »
- « Indiquez le cas échéant les méthodes pour réduire ou supprimer la douleur, la souffrance et l'angoisse (liste des médications anesthésiques, analgésiques, anti-inflammatoires… en précisant les doses, voies, durées et fréquences d'administration), y compris le raffinement des conditions d'hébergement, d'élevage et de soins »

L'évaluateur doit prendre connaissance des éventuelles modifications par rapport aux conditions de vie des animaux imposées par le CRPM et l'arrêté ministériel

fixant les conditions d'agrément, d'aménagement et de fonctionnement des établissements[33]. Il s'assure alors, au regard de l'état de l'art, que chaque situation qui n'est pas conforme avec ces textes est justifiée par un objectif scientifique ou au regard d'une bonne gestion du bien-être animal.

4.6.4 Conditions de mise à mort

Bien qu'il persiste dans le langage courant, le terme « euthanasie » a été remplacé dans les textes réglementaires de 2013 par « mise à mort » pour laquelle des conditions ont été édictées par la Commission Européenne dans la Directive 2010/63/UE et l'article de transposition en loi française, l'article R. 214-98 du CRPM.

Les méthodes autorisées, ainsi que les conditions particulières liées aux espèces et aux techniques, sont précisées dans l'annexe IV de l'arrêté du 1er février 2013 fixant les conditions d'agrément, d'aménagement et de fonctionnement des établissements. Ces méthodes visent à provoquer la mort le plus rapidement possible avec un minimum de douleur, de souffrance ou de détresse.

Le CEEA veille à l'adéquation entre les méthodes de mise à mort indiquées dans la demande d'autorisation, l'objectif scientifique des procédures et la prise en compte du bien-être des animaux. Il apporte une attention particulière aux situations dans lesquelles une méthode non reconnue est proposée.

Pour faciliter l'évaluation, les méthodes doivent être indiquées dans la DAP à l'item prévu. La mise à mort est également abordée à la fin de la partie « Description des procédures », où l'on indique quels animaux sont mis à mort.

Evaluation de la justification de la mise à mort

La justification de la mise à mort est donnée dans la partie « Devenir des animaux à la fin de cette procédure » dans la description de chaque PE.

Les situations les plus fréquentes dans lesquelles la mise à mort intervient au terme de la PE sont le prélèvement d'organes ou de tissus ou l'arrêt des effets délétères d'un test si aucun traitement n'est envisageable et que l'animal ne peut recouvrir un état général de santé et bien-être normal.

---

[33] *https://www.legifrance.gouv.fr/affichTexte.do?cidTexte=JORFTEXT000027037983&categorieLien=id*

En dehors de ces cas, le choix de la mise à mort doit être mis en balance avec les possibilités de réutilisation ou de placement des animaux. L'avis du vétérinaire ou d'une personne compétente est requis et renseigné dans la DAP.

Evaluation de la méthode de mise à mort

Les méthodes de mise à mort sont indiquées dans la DAP.

Le recours à une méthode qui n'est pas proposée dans l'annexe IV de l'arrêté d'application peut être envisagé par le rédacteur de DAP. L'évaluateur s'attachera alors à examiner la justification scientifique et la compatibilité de la méthode proposée avec le principe de Raffinement. Si la justification est admise par le CEEA (qui rend par la suite un avis favorable sur le projet) et autorisée par le ministère chargé de la recherche, ceci constituera de facto l'autorisation d'une méthode dérogatoire, dans le strict cadre du projet concerné.

Dans tous les cas, l'évaluateur vérifie aussi que la méthode est compatible avec l'objectif scientifique poursuivi.

Enfin, le CEEA doit s'interroger sur les modalités de la mise en œuvre de la méthode de mise à mort proposée.

4.6.5 Licéité, choix et mise en œuvre des procédures expérimentales

4.6.5.1 Domaine de licéité des procédures

Le CEEA doit s'assurer de la licéité d'une PE en analysant le ou les objets visés par la PE. Les objets autorisés sont indiqués dans le CRPM, article R.214-105.

Comme indiqué précédemment, il existe certaines restrictions pour les primates.

Seules sont licites les procédures expérimentales qui respectent les principes des 3R et qui ont un ou plusieurs des objets suivants :

a) La recherche fondamentale ;

b) Les recherches translationnelles ou appliquées menées pour :

- La prévention, la prophylaxie, le diagnostic ou le traitement de maladies, de mauvais états de santé ou d'autres anomalies ou de leurs effets chez l'homme, les animaux ou les plantes ;

- L'évaluation, la détection, le contrôle ou les modifications des conditions physiologiques chez l'homme, les animaux ou les plantes ;
- Le bien-être des animaux et l'amélioration des conditions de production des animaux élevés à des fins agronomiques ;

c) L'une des finalités visées au b lors de la mise au point, de la production ou des essais de qualité, d'efficacité et d'innocuité de médicaments à usage humain ou vétérinaire, de denrées alimentaires, d'aliments pour animaux et d'autres substances ou produits ;

d) La protection de l'environnement naturel dans l'intérêt de la santé ou du bien-être de l'homme ou de l'animal ;

e) La recherche en vue de la préservation des espèces ;

f) L'enseignement supérieur ou la formation professionnelle ou technique conduisant à des métiers qui comportent la réalisation de procédures expérimentales sur des animaux ou les soins et l'entretien de ces animaux ainsi que la formation professionnelle continue dans ce domaine ;

g) Les enquêtes médico-légales.

4.6.5.2 Choix des méthodes

Le choix des méthodes utilisées dans les PE doit permettre d'utiliser le moins d'animaux possible pour atteindre les objectifs du projet (article R.214-106 du CRPM).

Ce choix est de plus guidé par le souci de sélectionner les PE qui sont les plus susceptibles de fournir des résultats satisfaisants et satisferont le mieux aux exigences suivantes :

- utiliser les animaux des espèces les moins susceptibles de ressentir de la douleur, de la souffrance, de l'angoisse ou de subir des dommages durables dans les conditions de la PE et
- causer le moins possible de douleur, de souffrance, d'angoisse ou de dommages durables.

4.6.5.3 Point limite

Dans la mesure du possible, la mort doit être évitée en tant que point limite de la PE et remplacée par des critères d'arrêt précis adaptés et dont la mise en œuvre est aussi précoce que possible (article R. 214-107 du CRPM).

Aussi, prévoir, préalablement à la mise en œuvre des procédures, de limiter la survenue, l'intensité et la durée des contraintes qui pourraient être imposées aux animaux et générer douleur, souffrance ou angoisse est-il un devoir éthique pour tout concepteur de projet.

Les points limites adaptés doivent être les plus précoces possible et appropriés en fonction de chaque espèce, de chaque procédure et du but scientifique (ou éducatif ou réglementaire) recherché pour éviter toute souffrance inutile, scientifiquement non justifiée ou éthiquement non acceptable.

Un point limite doit être défini pour être appliqué dans différentes situations :

- les objectifs scientifiques ont été atteints et il n'existe pas de raison valable de poursuivre l'étude ;
- l'état d'un animal ne permet plus d'obtenir des informations scientifiques exploitables et pertinentes ;
- la souffrance est plus importante que prévue et le rapport entre les dommages apparus et les avantages attendus est défavorable ;
- des signes d'appel, non douloureux comme une perte de poids, et fiables, permettent d'anticiper l'apparition de signes plus graves.

Le CEEA vérifie que la détermination des points limites est passée par les étapes suivantes :

- prévision de l'apparition des signes de douleur, d'inconfort ou de détresse, qu'il s'agisse de signes visibles ou d'indicateurs non observables, et de leur évolution potentielle, quel que soit le protocole envisagé ;
- choix des indicateurs prédictifs les plus significatifs de la douleur ou de la contrainte induite : signes physiologiques, physiques ou comportementaux ;
- sélection du point limite adapté le plus précoce possible et compatible avec les objectifs de la procédure ;
- description de la conduite à tenir lorsqu'il est atteint.

Retour au sommaire

Ainsi, l'atteinte du point limite doit déclencher des actions, successives ou complémentaires, en accord avec l'état de l'animal et le contexte expérimental :

- intervention du responsable de la mise en œuvre général du projet et/ou du vétérinaire ;
- traitement antalgique de l'animal ou tout autre soin visant à améliorer son bien-être comme une lampe chauffante ou la facilitation de l'accès à l'eau de boisson ou à l'aliment ;
- modification de la PE (arrêt ou ajustement du traitement expérimental) ;
- suspension ou arrêt de la PE (pour l'animal concerné) ;
- mise à mort sans délai de l'animal dès l'instant où cette décision est prise.

Cette approche, qui doit être réalisée pour chaque PE, permet la bonne information du CEEA et sera utilement mise à disposition du personnel impliqué dans la mise en œuvre du projet.

Pour la partie qui s'intéresse au raffinement, l'évaluation éthique incite ainsi le rédacteur de DAP à proposer des signes annonciateurs de dégradation de l'état des animaux le plus précocement possible, qui permettent la mise en œuvre de soins adaptés. De fait, sans mettre en danger l'objectif scientifique de l'étude, ces mesures diminuent l'impact négatif des PE et permettent de diminuer le degré de gravité de la procédure.

Dans la mesure du possible (eu égard aux modèles animaux et à l'objectif de la PE), le rédacteur de DAP adoptera une **approche graduelle** dans l'application du point limite adapté, permettant dans un premier temps d'être alerté sur l'apparition de signes de dégradation de l'état général des animaux afin d'intervenir pour limiter les contraintes, puis en fonction de l'évolution de l'état général de chaque animal, objectivée par des critères prédéfinis, d'appliquer des mesures plus fortes de prise en charge, voire ultimement de décider de la mise à mort des animaux.

Prévoir la mise en place d'un suivi attentif qui est fondamental pour la mise en œuvre du point limite. Ce suivi porte sur l'observation de signes généraux ou spécifiques, cliniques ou comportementaux, en fonction du modèle. Il peut aussi se baser sur des paramètres non observables directement sur l'animal tels que des constantes biologiques ou des marqueurs moléculaires.

Retour au sommaire

Dans son évaluation, le CEEA s'assure que la fréquence d'observation est augmentée si l'état général des animaux s'aggrave ou si une douleur ou une souffrance apparait.

En sus des critères retenus, il est primordial d'avoir défini à l'avance les responsabilités et la chaine de communication et de prise de décision, notamment en cas de risque de conflit d'intérêts. Le CEEA peut demander des précisions sur les rôles respectifs des différents personnels impliqués dans la mise en œuvre du point limite anticipé :

- qui suit l'évolution des critères retenus ?
- que fait-on, qui décide et qui agit si le point limite adapté est atteint ?
- que fait-on s'il est atteint le week-end ou tout autre jour de fermeture ou d'activité réduite ?

4.6.5.4 Procédure expérimentale impliquant une contrainte intense susceptible de se prolonger sans qu'il soit possible de la soulager

Conformément à l'article R. 214-108 du CRPM, une PE ne peut pas être mise en œuvre si elle implique une douleur, une souffrance ou une angoisse intenses susceptibles de se prolonger sans qu'il soit possible de les soulager. Toute dérogation à cette interdiction ne peut être obtenue auprès du ministère chargé de la recherche que pour des raisons exceptionnelles dûment justifiées scientifiquement et doit être évaluée par le CEEA.

4.6.5.5 Recours à l'anesthésie, l'analgésie ou à toute autre méthode visant à réduire les contraintes

Sauf dans les cas où ce ne serait pas indiqué, les PE doivent être pratiquées sous anesthésie générale ou locale et en recourant à des analgésiques ou à toute autre méthode appropriée, afin que la douleur, la souffrance et l'angoisse soient limitées le plus possible (article R. 214-109 du CRPM).

Le CEEA évaluera les situations au cas par cas afin de s'assurer que les moyens adéquats sont mis en place ou que l'absence de méthode d'anesthésie ou d'analgésie est dûment justifiée et acceptable.

4.6.5.6 Utilisation continue, réutilisation des animaux et effets cumulatifs

La réglementation offre la possibilité d'utiliser à plusieurs reprises (on parle dans ce cas de réutilisation) des animaux à des fins scientifiques. Les conditions de cette réutilisation sont précisées dans l'article R214-113 du CRPM.

L'utilisation continue d'un animal au cours de plusieurs expériences n'est pas considérée comme une réutilisation, si l'objectif est de comparer les résultats de ces expériences chez ce même animal

Exemple : l'utilisation répétée d'un même animal pour une étude de pharmacocinétique d'un médicament dont on modifie l'excipient à chaque essai. Ce type d'étude compare la pharmacocinétique liée à différents excipients chez le même animal. Il s'agit d'une utilisation continue.

De la même manière, il n'y a pas lieu de découper la description des différentes phases d'un projet sous forme d'autant de PE dans la mesure où il s'agit d'un même projet et donc de l'utilisation continue d'un animal et non pas de réutilisation.

La réutilisation consiste à utiliser dans une nouvelle PE un animal qui a déjà été utilisé dans une PE. Ces procédures n'ont aucun lien entre elles, de sorte qu'un autre animal auquel aucune PE n'a été appliquée précédemment (animal dit « naïf ») pourrait aussi être utilisé.

La réutilisation permet la réduction du nombre d'animaux utilisés au travers des différentes procédures ou projets menés au sein des établissements. Cependant, elle peut nuire au raffinement en imposant à un animal donné les effets cumulatifs de PE successives, par exemple de sévérité importante et un temps de séjour au laboratoire plus long. **Des conditions strictes s'appliquent pour chaque réutilisation** et le sort de chaque animal concerné doit être pris en considération pour toute réutilisation.

L'évaluation par le CEEA d'une réutilisation d'animaux s'appuie à la fois sur les principes de réduction et de raffinement. Il est légitime de s'interroger, par exemple, sur le **nombre maximal** d'utilisations d'un même animal ou sur le **temps de séjour maximal** pour un animal dans un établissement. En l'absence de tout référentiel technique dans ce domaine, une réflexion éthique s'impose.

4.7 Analyse comparative des dommages et avantages du projet

Pour chaque projet, l'évaluateur recense les espèces et nombres d'animaux qui seront utilisés, ainsi que l'ensemble des contraintes qui induisent des dommages (douleurs, souffrances et dommages durables) aux animaux. Il relève également la liste des avantages attendus pour la recherche ou l'enseignement. L'évaluateur doit ensuite estimer la valeur de chaque élément d'information sur la base de laquelle le CEEA portera collectivement un jugement.

Comparer l'importance des dommages pour les animaux et des avantages pour la recherche ou l'enseignement engage des valeurs personnelles et professionnelles. Cette évaluation de la valeur intrinsèque de l'objectif scientifique ou pédagogique fait intervenir une composante sociétale dans l'évaluation de projet. Le législateur, en introduisant l'analyse dommages/avantages, souhaite qu'il soit tenu compte des attentes de la société civile sur les dommages acceptables.

La méthode suggérée en 2016 par le groupe de travail conjoint FELASA/AALAS « Harm/Benefit Analysis » est la suivante[34] :

1. recenser les bénéfices attendus : ils sont énoncés dans la description du projet ;
2. identifier les facteurs de modulation du bénéfice attendu :
    a) facteurs qui augmentent ce bénéfice : un besoin médical majeur, une large diffusion des conclusions, des résultats qui profiteront à d'autres thématiques scientifiques ou aux 3R ;
    b) facteurs qui réduisent ce bénéfice : des moyens insuffisants, une conception du projet de qualité insuffisante, un ou des modèles animaux mal décrits ou mal maîtrisés ;
3. recenser les contraintes : nombre d'animaux, espèce animale, caractère invasif ou délétère des pratiques générant douleur, souffrance, stress pris en compte dans la classification des PE selon leur degré de gravité ;

---

[34] *Laboratory Animals, Vol 50, Issue 1 Suppl 2016 Recommendations for Addressing Harm-Benefit Analysis and Implementation in Ethical Evaluation – Report from the AALAS-FELASA Working Group on Harm-Benefit Analysis – Part 2 Kathy Laber, Christian E Newcomer, Thierry Decelle, Jeffrey I Everitt, Javier Guillen, Aurora Brønstad*

4. identifier les facteurs de modulation des contraintes :

   a) facteurs qui augmentent les contraintes : un défaut dans la prévention ou le traitement de la douleur, un nombre d'animaux important, la sensibilité élevée du modèle (espèce, stade physiologique, stade de développement, génotype) ;

   b) facteurs qui réduisent les contraintes : le recours à des méthodes peu invasives, des méthodes d'analgésie ou de surveillance des animaux particulièrement adaptées, des points limites adaptés.

Chaque CEEA peut construire une grille de lecture des bénéfices et des contraintes qu'il utilisera systématiquement pour aider les évaluateurs à prendre position plus facilement.

Dans le cas où tous les autres éléments de la revue éthique d'un projet (licéité, 3R, points limites) sont évalués favorablement, une analyse dommages/avantages défavorable peut-elle justifier un avis défavorable ? Cette question donne tout son sens à la dénomination de comité d'éthique. En effet, un tel avis équivaut à donner un avis défavorable à un projet pour motif de valeur morale insuffisante.

Avant d'émettre un tel avis, le CEEA doit s'assurer qu'il a toutes les informations nécessaires à la compréhension du projet et il peut demander l'avis de représentants de la société civile et d'experts externes qu'il sollicitera. Si le projet comprend des PE de gravité au plus « modérée », il peut aussi accompagner son avis favorable d'une demande d'appréciation rétrospective qui lui permettra de réaliser une analyse comparative des dommages et des avantages non plus a priori, mais réelle. Elle pourra être utilisée pour les évaluations futures.

4.8 Nécessité d'une appréciation rétrospective

Les modalités de l'appréciation rétrospective (AR) sont présentées dans le chapitre 5 ci-après.

Conformément à l'article R214-120 du CRPM, l'AR est obligatoire pour les projets qui comportent au moins une procédure de classe de gravité sévère et pour les projets utilisant des primates.

Les projets qui ne comportent que des procédures de classe de gravité légère ou sans réveil en sont exemptés.

Pour les autres projets, le CEEA décide si une appréciation rétrospective doit être réalisée.

Les différentes situations sont présentées dans le tableau ci-dessous.

Tableau 1 : Règles relatives à l'obligation d'appréciation rétrospective

Article R214-120 du Code Rural et la Pêche Maritime	
**PROJET INCLUANT**	**APPRÉCIATION RÉTROSPECTIVE**
Au moins une procédure de classe de gravité "sévère"	Obligatoire
Au moins une procédure utilisant des Primates	
Au moins une procédure de classe de gravité "modérée"	Sur décision du Comité d'Ethique
Uniquement des procédures de classe de gravité "légère" ou "sans réveil"	Exemption

4.9 Expression d'un avis

L'avis du CEEA est l'étape ultime qui synthétise l'ensemble des réflexions décrites jusqu'ici menées par les évaluateurs, sur la base des discussions et échanges avec le rédacteur de DAP qui aura, le cas échéant, amendé son projet en conséquence. Cette synthèse se retrouve dans l'analyse comparative des dommages et avantages du projet que mène le CEEA.

Dans certains cas, le CEEA pourra considérer que certains critères ne lui permettent pas de donner un avis favorable. Ce peut être un défaut dans la justification du recours à l'animal, de la pertinence du modèle animal ou du nombre d'animaux, une carence manifeste dans la prévention ou la maitrise de la souffrance ou encore des bénéfices attendus trop peu tangibles.

Dans d'autres cas, le rédacteur de DAP sera en mesure de montrer que toutes les mesures possibles ont été envisagées mais qu'elles présentent des limites, par exemple lorsque le recours à une méthode plus raffinée n'est pas compatible avec l'objectif scientifique. Dans ce cas, le CEEA pourra donner un avis favorable tout en adressant en parallèle une recommandation au rédacteur dans le but qu'elle soit appliquée dès que l'état de l'art le permettra.

En aucun cas, le CEEA ne peut donner un avis favorable s'il émet des réserves par rapport au projet dans sa forme la plus aboutie après échanges avec le rédacteur de DAP.

Tout mode de communication de l'avis peut être utilisé entre le CEEA et le rédacteur de DAP. L'avis officiel quant à lui est transmis au ministère chargé de la recherche via un formulaire sur lequel le CEEA indique sa décision ainsi que certaines précisions :

- avis défavorable : il faut en donner les raisons ;
- avis favorable : aucune justification n'est demandée. Il faut cependant indiquer les éventuelles demandes de modification de degré de gravité d'une PE ou la demande d'AR.

Il faut aussi donner l'accord explicite du CEEA en cas de réutilisation d'animaux où l'avis du CEEA est requis (cas dérogatoire des PE sévères).

4.10 Place de l'évaluation éthique dans la modification d'un projet autorisé

L'avis du CEEA ne vaut que pour le projet soumis dans son ultime version avant autorisation. Il revient au demandeur et au CEEA de s'assurer de l'adéquation de l'avis avec la durée du projet.

Une fois le projet autorisé, le personnel de l'EU mentionné comme tel dans la DAP endosse la responsabilité de « Responsable de mise en œuvre du projet » ; il s'assure ainsi de la mise en œuvre du projet conformément à l'autorisation de projet délivrée par le ministère.

Si des évolutions doivent être apportées au projet (tel qu'il a été autorisé), il doit discuter avec la SBEA de l'impact potentiel sur le bien-être des animaux en vue d'introduire une demande de modification de l'autorisation de projet auprès du ministère lorsque ces changements ont une incidence négative sur le bien-être des animaux.

La demande de modification de l'autorisation fera l'objet d'une évaluation éthique qui s'attachera à mettre en balance l'impact de ces changements (dommages sur les animaux) au regard des avantages attendus afin de rendre un avis sur la version modifiée du projet.

Pour cette évaluation d'un projet modifié, le CEEA dispose d'un délai maximal de 3 semaines pour rendre son avis.

5 Appréciation rétrospective des projets

L'appréciation rétrospective (AR) d'un projet s'inscrit dans un processus d'amélioration continue. Elle permet de savoir a posteriori si le projet mis en œuvre a atteint ses objectifs scientifiques, éducatifs ou réglementaires (justifiant ainsi de la pertinence de l'approche) et si les dommages réellement subis par les animaux correspondent à l'évaluation qui en avait été faite a priori.

L'AR permet d'améliorer les connaissances sur les modèles animaux et de mieux apprécier leur contribution à l'atteinte des objectifs de chaque projet. Elle permet au CEEA de mettre en perspective, à l'issue de la mise en œuvre du projet, l'analyse comparative des dommages et avantages qu'il avait menée lors de l'évaluation éthique.

Elle est réalisée par le CEEA sur la base des éléments transmis par le demandeur une fois le projet achevé et grâce auxquels le CEEA rédige une synthèse qu'il pourra utiliser pour les évaluations futures.

La réglementation demande que l'AR soit menée à l'issue du projet. Le CEEA précise dans sa demande à quel moment elle sera réalisée et quand il souhaite en recevoir les éléments. Ce moment peut dépendre du délai d'obtention de certaines données comme par exemple des résultats d'analyses histologiques, obtenues bien après la fin de mise en œuvre de la dernière PE.

Dans certains cas, en dehors du cadre strictement réglementaire, le CEEA peut souhaiter recevoir des informations pendant la réalisation du projet pour faire des points d'avancement intermédiaires, c'est ce qu'on appelle les évaluations d'étape, qui concourent à la promotion des principes éthiques (non détaillée dans ce guide).

5.1 Méthodes et moyens mis en œuvre pour l'appréciation rétrospective

Pour être correctement menée (conformément à l'article 7 de l'AM), l'AR se base sur des informations fournies par le demandeur.

Le (ou les) responsables du projet pourra(ont) utilement collaborer avec la SBEA et tout autre acteur de l'EU qui pourra l'aider à rassembler des éléments qui concerneront la mise en œuvre des 3R ou la classe de gravité des procédures, ainsi que les évènements imprévus.

Compte tenu de la durée du projet et du temps nécessaire au recueil des informations, plusieurs années peuvent s'écouler entre la demande d'AR mentionnée par le ministère dans la notification d'autorisation de projet et la réalisation de l'AR. Il est fortement conseillé au responsable du projet de s'assurer pendant toute cette période que l'ensemble des informations nécessaires à l'AR sont collectées et sauvegardées.

Le CEEA attend du responsable du projet un compte-rendu sur trois points : si les objectifs du projet ont été réalisés ; des informations sur les dommages infligés aux animaux, ainsi que le nombre et les espèces des animaux utilisés et la gravité réelle des procédures expérimentales ; les éléments qui peuvent contribuer à renforcer l'application des exigences de remplacement, de réduction et de raffinement.

Les évaluateurs demandent que ces informations soient présentées de façon claire et accessible, sur la base de données factuelles issues des PE réalisées. L'évaluation peut nécessiter une expertise scientifique dont le Comité ne dispose pas et l'appel à un expert externe peut être envisagé.

Une fois les informations obtenues et l'AR réalisée, le CEEA informe le responsable du projet des conclusions de son analyse. L'AR peut comprendre des recommandations à prendre en compte dans des projets futurs.

On comprendra aisément que les appréciations rétrospectives sont des bilans pour progresser en termes de 3R. Des modèles de questionnaires relatifs à l'appréciation rétrospective sont proposés par les CEEA.

5.2 Atteinte des objectifs scientifiques, réglementaires ou pédagogiques du projet

L'AR permet de savoir si le projet a répondu à la question posée, justifiant ainsi l'autorisation qui a été obtenue. Si ce n'est pas le cas, le CEEA s'assure qu'il dispose des éléments lui permettant d'analyser les raisons de cet échec et d'en tirer les conséquences pour les futurs projets. On se questionnera alors sur l'adéquation entre les modèles animaux, les moyens mis en œuvre et les objectifs prédéfinis.

5.3 Prise en compte des dommages constatés et de la gravité réelle des procédures expérimentales

Le degré de gravité réelle est déterminé pour chaque animal, à la fin de chaque utilisation, sur la base de toutes les observations faites au cours de la mise en œuvre des PE.

Tous les éléments utiles à l'AR doivent être transmis au CEEA :

- le nombre d'animaux utilisés par espèce ;
- un récapitulatif des degrés de gravité réelle subis par les animaux en lien avec les PE, incluant l'effet cumulatif éventuel lié à la réutilisation d'animaux ;
- une description des contraintes et des dommages inattendus ou supplémentaires subis par les animaux qu'ils soient liés au projet ou non. Ceci permet d'apprécier des difficultés techniques qui n'auraient pas été prévues dans la DAP ;
- une description du devenir des animaux : mise à mort prévue en fin de PE, mise à mort suite à l'atteinte de points limites anticipés, animal trouvé mort, animal gardé en vie (réutilisation, placement, cession).

Le responsable du projet s'assure que ces informations sont collectées et enregistrées pendant le déroulement du projet. Des grilles d'évaluation des contraintes, du stress et/ou de la douleur sont très utiles pour cette évaluation. Pour des exemples précis, on peut se référer aux publications de la Commission européenne[35].

---

[35] *Evaluation de projet et appréciation rétrospective (https://ec.europa.eu/environment/chemicals/lab_animals/pdf/guidance/project_evaluation/fr.pdf) et Cadre d'évaluation de la gravité des procédures (https://ec.europa.eu/environment/chemicals/lab_animals/pdf/guidance/severity/fr.pdf)*

5.4 Contribution du projet au principe des 3R

Parmi les informations qu'il transmet au CEEA en vue de l'AR, le demandeur explique la façon dont le principe des 3R a été appliqué lors de la mise en œuvre du projet.

Remplacement : depuis le début du projet, y a-t-il eu des avancées techniques ou technologiques permettant de se passer de l'utilisation d'animaux en les remplaçant par des modèles non animaux ?

Réduction : le nombre d'animaux utilisé a-t-il été identique, inférieur ou supérieur au nombre d'animaux prévu et pour quelle raison ?

Raffinement : lors de la demande d'autorisation du projet en question, des mesures de raffinement ont été proposées. Il s'agit de savoir si elles ont été appliquées et efficaces, voire si elles ont pu être améliorées en cours de mise en œuvre du projet sur la base de questions telles que les suivantes :

- a-t-il été nécessaire d'appliquer les points limites anticipés prévus ? Etaient-ils pertinents ou trop tardifs ? A-t-il été possible de les améliorer ?
- le protocole de prévention ou traitement de la douleur a-t-il été efficace ou a-t-il été nécessaire de le revoir ?
- des soins ont-ils été nécessaires suite à l'apparition de troubles cliniques inattendus ?
- le cas échéant, comment ont été gérées les contraintes relatives à l'hébergement et à l'entretien des animaux ? En particulier, comment a été compensée la mise en hébergement individuel d'animaux sociaux ? Cela a-t-il été efficace ? Des signes de stress ont-ils été notés ? Faut-il à l'avenir, renforcer le protocole d'interaction entre congénères ?

L'AR permet ainsi de poser un regard plus objectif sur les modèles et la méthodologie utilisés et de promouvoir le raffinement.

Conclusion

Mise en place de manière volontaire par de nombreux établissements français dès les années 1990, l'évaluation des projets est désormais ancrée dans la réglementation suite à la transposition de la Directive européenne 2010/63/UE.

Fortement réglementée et basée sur des référentiels scientifiques et techniques nationaux et internationaux reconnus, elle n'en conserve pas moins une portée éthique forte au regard des principes qui la soutiennent, de la composition et de l'organisation des comités d'éthique qui la réalisent et de l'importance qu'elle revêt pour la société civile.

Au-delà de l'avis porté sur chaque projet en vue de son autorisation, elle contribue à une sensibilisation continue des personnes responsables de la mise en œuvre des projets et à une amélioration continue des pratiques expérimentales, notamment au travers de l'exercice d'appréciation rétrospective des projets. En ce sens, l'action des comités d'éthique est complémentaire de celle des structures chargées du bien-être des animaux qui accompagnent la réalisation des projets sur le terrain et délivrent des conseils en matière de bien-être animal et de mise en œuvre de la règle des 3R.

Ce nouveau référentiel aspire à donner sa pleine dimension à l'évaluation éthique des projets utilisant des animaux à des fins scientifiques. Au-delà de la prise en compte de critères techniques, la mise en jeu d'une analyse comparative entre les dommages et les avantages élève l'exercice d'évaluation au plus proche des préoccupations de la société civile qui est attachée à une utilisation responsable des animaux.

Souhaitons qu'il en soit fait un bon usage par la communauté scientifique afin de lui donner cette juste portée.

GRICE

Guide de l'évaluation éthique des projets impliquant l'utilisation d'animaux à des fins scientifiques

En 2009, le GRICE, Groupe de réflexion interprofessionnel sur les comités d'éthique appliqués à l'expérimentation animale, publiait le Guide de l'évaluation éthique des études sur animaux alors document de référence approuvé par le CNREEA, Comité national de réflexion éthique en expérimentation animale, pour les chercheurs qui souhaitent avoir recours à l'expérimentation animale et pour les comités d'éthique en expérimentation animale (CEEA).

Suite à la transposition en droit français de la Directive 2010/63/UE, les comités d'éthique sont devenus des autorités compétentes en charge de l'évaluation éthique des projets. A ce titre, le Directeur Général de la Recherche et de l'Innovation du ministère chargé de la recherche a sollicité le GRICE en 2012 pour rédiger des documents de référence sur le fonctionnement des comités d'éthique.

Ce guide répond à cette demande pour ce qui concerne les modalités de l'évaluation éthique des projets par les comités. Il s'appuie sur les textes réglementaires et sur la Charte nationale portant sur l'éthique de l'expérimentation animale et propose un ensemble de principes afin d'aider chacun des membres des CEEA à remplir la mission d'évaluation éthique préalable à l'autorisation des projets et celle d'appréciation rétrospective des projets.

Il engage ainsi à une harmonisation du fonctionnement des comités français pour assurer un traitement homogène des projets, tout comme il permet aux concepteurs de projet d'avoir une bonne compréhension des principes et critères sur la base desquels leurs projets sont évalués.

Éditions MIMOSA - 04 67 99 49 49
