# Supplementary material for: Mission impossible accomplished? A European cross-national comparative study on the integration of the harm-benefit analysis into law and policy documents
Source: PLoS One. 2024 Feb 20;19(2):e0297375. doi: 10.1371/journal.pone.0297375 (PMC10878508; doi:10.1371/journal.pone.0297375)
Supplement: S5 File — (PDF) [file pone.0297375.s005.pdf]

Ethisch toetsingskader voor proefdiergebruik
Praktische handreiking voor Dierexperimentencommissies

Inleiding

Een kerntaak van de Centrale Commissie Dierproeven (CCD) is het beoordelen of projectvergunningsaanvragen voor dierproeven voldoen aan wet- en regelgeving. Een onderdeel van de beoordeling is het uitvoeren van een ethische toets, waarbij het belang van het onderzoek wordt afgewogen tegen het ongerief dat het proefdier bij dit onderzoek ondergaat. Dit is de zogenoemde schade-batenanalyse.

De CCD vraagt voor de beoordeling van projectvergunningsaanvragen advies aan een dierexperimentencommissie (DEC). Het advies van de DEC is zwaarwegend voor de CCD. Het is daarom van belang dat een DEC alle in de Wet op dierproeven (Wod) genoemde criteria betrekt bij de totstandkoming van haar advies, en dat de ethische afweging van de DEC voldoende navolgbaar en onderbouwd is. Voorliggend ethisch toetsingskader biedt handvatten voor het maken en verwoorden van de schade-batenanalyse.

Een moeilijkheid bij het maken van de ethische afweging is dat de factoren die ten opzichte van elkaar gewogen moeten worden niet direct vergelijkbaar zijn. Het ongerief van de proefdieren moet namelijk gewogen worden tegen de baten voor de mens, andere dieren (de doeldieren) en/of het milieu. Ook kunnen bij het maken van een ethische afweging persoonlijke waarden een rol spelen. Bij individuele leden van een DEC kan dit tot een verschillende uitkomst van de ethische afweging leiden. Om de discussie te stroomlijnen en te borgen dat alle voor de beoordeling essentiële criteria betrokken worden, is het nodig om eerst een goed beeld van het morele probleem en de context te krijgen. Pas als alle voor de ethische afweging relevante feiten inzichtelijk zijn gemaakt, kan de ethische afweging gemaakt worden. Dit ethisch toetsingskader voor proefdiergebruik onderscheidt daarvoor de volgende stappen:

I) Probleem definiëren
II) Probleem analyseren
III) Probleem wegen
IV) Advies

Bovengenoemde stappen worden in dit document in meer detail beschreven.

Voordat gestart kan worden met het maken van de ethische afweging is het van belang te beoordelen of de aanvraag vergunningplichtig is. Uit de Wod volgt wanneer een projectvergunning vereist is (artikel 1, lid 1 onder a en artikel 1b Wod). Voor meer informatie daarover zie de handreiking *Wat is een dierproef* op de website van de CCD.

Ook is het van belang te beoordelen of het wettelijk gezien wel toegestaan is om dierproeven uit te voeren voor de beschreven doelstelling en met de aangevraagde dieren. Het is in Nederland bijvoorbeeld niet toegestaan om proeven uit te voeren waarbij gebruik wordt gemaakt van chimpansees, bonobo’s, orang-oetans en gorilla’s (Artikel 10e, lid 1 Wod). Ook is het niet toegestaan LD50/LC50-methoden toe te passen (Artikel 10, lid 3

Wod) en dierproeven te verrichten voor het ontwikkelen van nieuwe, dan wel het testen van bestaande cosmetica (Artikel 10d Wod).

Tot slot is het in Nederland niet toegestaan dierproeven uit te voeren als de doelstelling ook zonder dierproef kan worden bereikt, of met een dierproef waarbij minder dieren kunnen worden gebruikt of minder ongerief wordt berokkend (Artikel 10, eerste lid, onder a Wod). Als dit van toepassing is in een aanvraag, kunnen de desbetreffende onderdelen in een aanvraag niet worden vergund. Als er nog andere proeven in de aanvraag worden beschreven die wettelijk wel zijn toegestaan, kan daarvoor nog wel een ethische afweging worden gemaakt.

Als het wettelijk gezien is toegestaan dierproeven uit te voeren voor de in de aanvraag beschreven doelstelling en met de beschreven dieren, en daarvoor een vergunning vereist is, kan de beoordeling van de aanvraag worden gestart.

Stap I) Probleem definiëren

A) Wat is de centrale morele vraag voor de DEC met betrekking tot het project?

Het is voor de DEC belangrijk om voorafgaand aan de discussie over een aanvraag vast te stellen welke morele vraag beantwoord moet worden en deze expliciet te verwoorden. Het helder formuleren van de centrale morele vraag is ook van belang voor een goede onderbouwing van het DEC-advies ten overstaan van anderen, in dit geval de CCD en/of de aanvrager.

Een centrale morele vraag moet met ja of nee te beantwoorden zijn en het probleem dient zo goed mogelijk te worden verwoord. De vraag moet bovendien in zijn formulering neutraal zijn.

In zijn algemeenheid ziet de centrale morele vraag er als volgt uit:
Rechtvaardigt het directe doel van het project (indien van toepassing aangevuld met het uiteindelijke doel) in combinatie met de haalbaarheid van het project, het ongerief dat dieren wordt aangedaan?

Deze centrale morele vraag wordt in het Format DEC advies weergegeven onder D1.

Stap II) Probleem analyseren

Het is na het vaststellen van de centrale morele vraag van belang om inzicht te krijgen in alle feiten die relevant zijn voor de ethische afweging. Het gaat hierbij om de onderwerpen die aan de orde komen in deel C van het Format DEC advies. Deze onderwerpen worden hieronder besproken. Bij elk van de punten is de corresponderende vraag in het Format DEC advies weergegeven.

<u>C1 Is de aanvraag toetsbaar?</u>

Om een ethische afweging te kunnen maken, is het van belang dat een aanvraag toetsbaar is. Met andere woorden, is alle voor de ethische afweging benodigde informatie op heldere wijze beschreven in de aanvraag? Dit betekent dat duidelijk moet zijn wat de doelstellingen van het project zijn en op welke wijze de aanvrager beoogt de doelstelling te behalen (de strategie). Dit is van belang voor de beoordeling van de haalbaarheid van de doelstelling. Om te kunnen beoordelen of dieren niet onnodig gebruikt worden in proeven, moet inzichtelijk zijn gemaakt op basis van welke criteria de aanvrager gedurende het project besluiten zal nemen over de voortgang van het project (keuzemomenten).

Ook moet inzichtelijk zijn gemaakt welke handelingen de individuele dieren ondergaan, welk ongerief en andere welzijnsaantastingen de dieren ondergaan. Tot slot moet inzichtelijk zijn gemaakt op welke wijze de aanvrager het principe van de 3V's (vervanging, vermindering en verfijning) toepast.

Grote onderzoeksprogramma's zijn over het algemeen op een zodanig laag abstractieniveau geschreven te zijn dat het niet mogelijk is om een ethische afweging te kunnen maken. Een te brede doelstelling en een te groot aantal subdoelstellingen moeten worden vermeden. Ook de strategie en de keuzemomenten dienen navolgbaar te worden beschreven. Zie voor meer informatie over de toetsbaarheid van een aanvraag de handreiking *Invulling definitie project* op de website van de CCD. De DEC wordt gevraagd om weer te geven of een aanvraag toetsbaar is. Als de aanvraag niet te beoordelen is, kan ook geen ethische afweging worden gemaakt. Zie D3 voor meer informatie (stap III).

<u>C2 Is andere wet- en regelgeving van toepassing?</u>

De aanvrager wordt gevraagd in het projectvoorstel aan te geven of andere wetgeving van toepassing is en, indien nodig, ontheffing is aangevraagd/verkregen. Dit is van belang, omdat sommige wetgeving voorrang heeft op de Wod.

Indien in andere wetgeving is opgenomen dat dieren en/of handelingen aan dieren uitgezonderd kunnen zijn van de desbetreffende wetgeving, dient de CCD te beoordelen of aan de daarvoor gestelde voorwaarden in die wet wordt voldaan.

Indien andere wetgeving die buiten de bevoegdheid van de CCD valt voorrang heeft op de Wod, kan het project mogelijk niet/niet volledig worden uitgevoerd. Hiermee komt de haalbaarheid van het project in het geding en worden de dieren mogelijk onnodig gebruikt. Het is van belang om te voorkomen dat dergelijke dierproeven uitgevoerd worden. In sommige gevallen kan de Minister ontheffing verstrekken, waardoor de desbetreffende handelingen in de dierproeven alsnog kunnen worden uitgevoerd. De aanvrager is zelf verantwoordelijk voor het aanvragen van een dergelijke ontheffing bij de verantwoordelijke instanties.

Als de DEC vermoedt dat andere wetgeving van toepassing is dan benoemd door de aanvrager, dan wordt zij gevraagd dit te signaleren in het advies.

<u>C3 Voor welke doeleinden worden de dieren gebruikt?</u>

In de Wod worden de volgende doeleinden beschreven waarvoor proefdieren gebruikt kunnen worden: fundamenteel onderzoek, translationeel of toegepast onderzoek, wettelijk vereist onderzoek of routinematige productie, onderzoek ter bescherming van het milieu in het belang van de gezondheid of het welzijn van mens of dier, onderzoek gericht op het behoud van de diersoort, hoger onderwijs of opleiding en forensisch onderzoek. De DEC wordt gevraagd te beoordelen of de juiste doeleinden zijn aangegeven in het project.

<u>C4 Is het doel van het onderzoek gerechtvaardigd binnen de context van het onderzoeksveld?</u>

In de projectaanvraag wordt een omschrijving van zowel het directe doel als het uiteindelijke doel gegeven. Het directe doel dient te kunnen worden behaald gedurende de looptijd van het project. Het uiteindelijke doel hoeft niet te worden behaald binnen de looptijd van het project. Zowel het directe als het uiteindelijke doel zijn voor de ethische afweging van belang.

Bij fundamenteel onderzoek is er over het algemeen geen directe relatie tussen het directe doel (bijvoorbeeld het leveren van inzichten in bepaalde mechanismen) en het uiteindelijke

doel (bestrijding van ziekten bij de mens). Het is dan van tevoren niet in te schatten of het uiteindelijke doel daadwerkelijk behaald zal worden of wat het uiteindelijke doel is (soms is de uiteindelijke toepassing niet vooraf vast te stellen). De relatie tussen het directe doel en het uiteindelijke doel zal in de aanvraag met terughoudendheid verwoord moeten zijn. Bij de ethische weging kan worden afgewogen of het verkrijgen van de in de aanvraag benoemde wetenschappelijke kennis voldoende rechtvaardiging is voor proefdiergebruik.

Voor translationeel onderzoek ligt dit anders. Voor dergelijke projecten zal duidelijk moeten zijn dat er een directe en reële relatie is tussen het directe doel en het uiteindelijke doel. Dit betekent dus dat het waarschijnlijk is dat het uiteindelijke doel binnen een specifiek tijdvak behaald zal worden. Het kan voorkomen dat de DEC tot de conclusie komt dat het directe doel weliswaar haalbaar is, maar dat de bijdrage aan het uiteindelijke doel beperkt zal zijn of zeer onzeker is. Dat gegeven is van belang bij de uiteindelijke afweging.

Voor de ethische afweging is het niet alleen van belang te beoordelen of de doelstelling haalbaar is, maar ook of het directe doel gerechtvaardigd is binnen de context van het onderzoeksveld. Om dit te kunnen beoordelen, is het van belang dat de wetenschappelijke kennis waarop het project gebaseerd is, compleet en eenduidig is. Ook moet duidelijk zijn wat het project bijdraagt aan het onderzoeksveld. Zie de handreiking *Synthesis of Evidence in proefdieronderzoek* van het NCad voor meer informatie over de wijze waarop een dergelijk vooronderzoek kan worden uitgevoerd. Zonder tot een diepgaand wetenschappelijk oordeel te komen, moet de DEC ervan overtuigd zijn dat het projectvoorstel aansluit bij recente inzichten en geen belangrijke hiaten heeft die op een beperkte bruikbaarheid van de resultaten kunnen wijzen. De DEC wordt gevraagd om dit oordeel op te nemen in haar advies.

<u>C5 Wie zijn de belanghebbenden in het project?</u>

Om tot een moreel oordeel te komen is het belangrijk om inzichtelijk te maken wie/welke groepen belang hebben bij de uitvoering of de resultaten van het project. Mogelijke belanghebbenden zijn: proefdieren, patiënten, doeldieren, vergunninghouders, onderzoekers, en andere voor het project relevante belangengroepen of entiteiten, zoals het milieu en de samenleving als geheel. Het is niet alleen van belang inzichtelijk te maken wie de belanghebbenden van het project zijn, maar ook welke waarden (ideaalbeelden of belangrijke principes die nagestreefd worden) op het spel staan voor elk van de belanghebbenden. Hierbij kan gedacht worden aan bevordering of aantasting van waarden op het gebied van welzijn, autonomie en rechtvaardigheid. In tabel 1 staan voor bovengenoemde belanghebbenden voorbeelden weergegeven van waarden die in het geding kunnen zijn of bevorderd kunnen worden.

Tabel 1:
Enkele voorbeelden van kernwaarden die op het spel kunnen staan voor verschillende belanghebbenden.

Morele waarden<br>Belanghebbenden	Welzijn	Autonomie	Rechtvaardigheid
Proefdieren	Gezondheid<br>Pijn<br>Stress	Natuurlijk gedrag	Alternatieven<br>Intrinsieke waarde<br>Integriteit
Doeldieren	Gezondheid<br>Pijn<br>Stress	Natuurlijk gedrag	Proportionaliteit<br>Intrinsieke waarde
Doelgroep(en) project	Kwaliteit,	Keuzevrijheid	Beschikbaarheid van

	Veiligheid<br>Gezondheid<br>Pijn		bijvoorbeeld het product<br>Proportionaliteit
Vergunninghouder, Onderzoekers	Commerciële, wetenschappelijke ontwikkelingen	Vrijheid van handelen	Wetgeving (bestaande)
Andere voor het project relevante belangen-groepen of entiteiten, zoals milieu en de samenleving als geheel	Conservatie	Biodiversiteit<br>Natuurlijkheid	Duurzaamheid<br>Voorzorg

Proefdieren:
Voorbeelden van waarden op het gebied van welzijn en autonomie die voor proefdieren in het geding kunnen zijn: goede gezondheid, vrij zijn van pijn en stress en de mogelijkheid om soort-specifiek gedrag te vertonen. Voor proefdieren kunnen ook waarden op het gebied van rechtvaardigheid in het geding zijn. Hierbij kan gedacht worden aan waarden zoals respect voor de intrinsieke waarde of integriteit van het dier. Daarnaast volgt uit de Wod dat dierproeven niet mogen worden uitgevoerd als er een alternatief is waarvoor geen of minder dieren nodig zijn (nee, tenzij…). Deze artikelen in de wet komen voort uit het huidige uitgangsprincipe dat dieren alleen gebruikt worden voor proeven als het echt niet anders kan.

Doeldieren:
Voor doeldieren kunnen dezelfde waarden op het gebied van welzijn en autonomie in het geding zijn als voor proefdieren. Denk hierbij bijvoorbeeld aan proeven gericht op het verder vergroten van de productiecapaciteit van dieren (zie voor meer informatie Uitvoeringsbeleid CCD *Aanvragen t.b.v. veehouderij*). Voor doeldieren kunnen deze waarden echter ook bevorderd worden, bijvoorbeeld als de resultaten van een project leiden tot een betere gezondheid van de doeldieren. Voor doeldieren kunnen ook waarden op het gebied van rechtvaardigheid in het geding zijn. Hierbij kan gedacht worden aan waarden als de intrinsieke waarde of integriteit van het dier en proportionaliteit van de handelingen aan het doeldier (verhouding tussen doel en ingezette middel).

Doelgroepen:
Voor doelgroepen zoals patiënten kunnen verschillende waarden op het gebied van welzijn bevorderd worden. Hierbij kan gedacht worden aan goede gezondheid, vrij zijn van pijn en stress en goede kwaliteit van leven. Daarnaast kunnen ook waarden op het gebied van autonomie en rechtvaardigheid bevorderd worden, zoals keuzevrijheid (bijvoorbeeld meerdere behandelingsmogelijkheden), de beschikbaarheid van het product voor alle patiënten en de proportionaliteit van beschikbare behandelmethodes. Bij de maatschappij kan worden gedacht aan waarden op het gebied van welzijn en rechtvaardigheid, zoals zo min mogelijk zieke mensen en beschikbaarheid van gezond voedsel voor iedereen.

Onderzoekers en vergunninghouders:
Ook voor onderzoekers en vergunninghouders geldt dat er verschillende waarden bevorderd kunnen worden door de resultaten van het project of het verkrijgen van de vergunning. Als het gaat om waarden op het gebied van welzijn of autonomie kan voor vergunninghouders en onderzoekers gedacht worden aan commerciële belangen, carrière, wetenschappelijke ontwikkeling en vrijheid van handelen. Bij rechtvaardigheid kan gedacht worden aan de bestaande wetgeving waaruit volgt dat het vereist is om dierproeven te doen om een product op de markt te brengen. Hierbij kan ook gedacht worden aan het in stand houden van een goede concurrentiepositie ten opzichte van andere (EU)landen.

Milieu:
Ook voor het milieu geldt dat er verschillende waarden bevorderd of aangetast kunnen worden. In de huidige samenleving wordt conservatie van dier- of plantensoorten, bevordering van biodiversiteit en bevordering van natuurlijkheid van gebieden steeds vaker als ideaalbeeld gezien. Dit betreft waarden op het gebied van welzijn en autonomie. Dergelijke waarden kunnen door proefdieronderzoek zowel bevorderd als aangetast worden. Bij rechtvaardigheid kan gedacht worden aan wet- en regelgeving waarin vereist wordt dat landen stappen nemen om klimaatveranderingen te verminderen.

De DEC wordt gevraagd om inzichtelijk te maken welke waarden worden bevorderd en in het geding zijn.

Zowel de kans dat de bovenstaande waarden bevorderd of aangetast worden als de mate waarin dat gebeurt, zijn relevant voor de ethisch afweging. Dit komt aan de orde bij de waardering van de waarden in stap III (probleem wegen).

De waarden die op het spel staan zullen niet door iedereen in de DEC als even belangrijk gewogen worden. Dit kan aan de orde komen wanneer de waarden later in stap III (Probleem wegen) gewaardeerd en gewogen worden. Als al direct duidelijk is dat alle DEC-leden een waarde slechts in beperkte mate moreel relevant vinden, is het goed om deze toch te benoemen in het DEC-advies, zodat inzichtelijk gemaakt kan worden dat de DEC zich bewust is dat de desbetreffende waarden voor een of meerdere belanghebbenden op het spel staan. Bij stap III (probleem wegen) kan dan worden aangegeven en onderbouwd dat de DEC deze moreel maar beperkt relevant vindt.

De verschillende waarden die op het spel staan kunnen strijdig zijn. Bevordering van biodiversiteit kan bijvoorbeeld samengaan met een aantasting van de vrijheid van handelen van de mens. De waardering en weging van deze waarden komt in stap III aan bod. In stap II is het van belang om de verschillende waarden inzichtelijk te maken.

<u>C6 Welk effect heeft het project op het milieu?</u>

Uit de Wod volgt dat dierproeven zo humaan en milieuvriendelijk als mogelijk uitgevoerd moeten worden (Artikel 10a2 lid 1 onder c Wod). Daarnaast dienen de voordelen van een project voor mens, dier en milieu te worden betrokken bij de schade-batenanalyse (Artikel 10a2 lid 2 onder d Wod). De DEC wordt daarom gevraagd te beoordelen of de in het project beschreven milieueffecten voldoende zijn weergegeven, en dit dient in stap III ook te worden meegewogen.

<u>C7 Zijn kennis en kunde van de onderzoeksgroep en andere betrokkenen bij de dierproeven voldoende gewaarborgd?</u>

Het is van belang te beoordelen of de kennis en kunde van de onderzoeksgroep en andere personen die betrokken zijn bij de dierproeven voldoende zijn gewaarborgd. Wanneer er niet voldoende kennis en kunde bij de onderzoeksgroep aanwezig is, kan dit de haalbaarheid van het project in het geding brengen. Er dient echter ook voldoende kennis en kunde aanwezig te zijn om te kunnen voldoen aan de 3V-beginselen en om te kunnen voorkomen dat mens, dier en het milieu onnodige negatieve effecten ondervinden als gevolg van de dierproeven. Het is moreel niet aanvaardbaar om dierproeven te doen als niet aan deze zorgvuldigheidseisen wordt voldaan. De DEC wordt gevraagd om dit te beoordelen.

<u>C8 Hoe realistisch is de geschetste haalbaarheid van het project?</u>

In het Europose werkdocument *Project evaluatie en Beoordeling achteraf* worden handvatten gegeven voor de beoordeling van projectvergunningen. In dit document wordt aangegeven dat zowel de mogelijke uitkomst als de haalbaarheid van de doelstellingen van het project moeten worden geanalyseerd en gewogen. Ook in de wet staat dat bij de schade-batenanalyse het te verwachten resultaat moet worden meegenomen (Artikel 10a2 lid 2 onder d Wod).

Het gebruik van proefdieren voor een bepaald project kan niet gerechtvaardigd worden als op voorhand helder is dat de doelstellingen niet op de beschreven wijze of met de betrokken personen behaald kunnen worden. Het is daarom van belang om te beoordelen of alle doelstellingen realistisch zijn verwoord en of met de voorgestelde strategie, dierexperimenten en betrokken personen die doelstellingen haalbaar zijn binnen de looptijd van het project.

<u>C9 Worden dieren gebruikt die in principe niet in dierproeven mogen worden gebruikt?</u>

Uit de Wod volgt dat een aantal specifieke groepen dieren in principe niet mogen worden gebruikt in dierproeven. Van dit uitgangspunt kan alleen worden afgeweken indien wetenschappelijk is onderbouwd waarom het doel van de proef alleen kan worden bereikt als gebruik gemaakt wordt van de desbetreffende dieren. Daarnaast gelden er voor elk van deze categorieën dieren nog specifieke beperkende voorwaarden. Indien deze dieren toch zullen worden gebruikt is het van belang te beoordelen of het voor het behalen van de doelstelling noodzakelijk is om deze dieren te gebruiken en of aan alle in de wet genoemde beperkende voorwaarden is voldaan.

Het gaat hierbij om de volgende categorieën dieren:

Bedreigde diersoort(en):
In de Wod staat dat bedreigde diersoorten (Artikel 10e, lid 4 Wod) in principe niet gebruikt mogen worden voor dierproeven. Hier kan alleen van worden afgeweken indien wetenschappelijk is onderbouwd waarom het doel van de proef alleen kan worden bereikt als gebruik gemaakt wordt van deze dieren. Bovendien mogen bedreigde diersoorten niet voor alle doeleinden gebruikt worden. Bedreigde dieren mogen alleen worden gebruikt voor translationeel onderzoek gericht op vermijding, voorkoming, diagnose of behandeling van ziekten, gezondheidsstoornissen of andere afwijkingen, dan wel de gevolgen daarvan, bij mensen, dieren of planten. Ook wettelijk vereist onderzoek en onderzoek gericht op het behoud van de soort zijn toegestaan.

Niet-humane primaten:
Zoals eerder aangegeven mogen chimpansees, bonobo's, orang-oetans en gorilla's nooit worden gebruikt voor dierproeven (Artikel 10e, lid 1 Wod). Ook voor de overige niet-humane primaten is het uitgangspunt dat deze niet gebruikt worden in dierproeven (Artikel 10e Wod). Daarop kan wel een uitzondering worden gemaakt. Dit kan echter alleen als wetenschappelijk is aangetoond dat het doel van de proef alleen kan worden bereikt als gebruik gemaakt wordt van niet-humane primaten. Bovendien mogen niet-humane primaten niet voor alle doeleinden gebruikt worden. Als een project translationeel of wettelijk vereist onderzoek betreft dan dient dit te zijn gericht op vermijding, voorkoming, diagnose of behandeling van gezondheid ondermijnende of mogelijk levensbedreigende klinische aandoeningen bij de mens. Is dit niet het geval, dan zijn de dierproeven alleen toegestaan als de Minister daarvoor ontheffing verleent. In die gevallen wordt de DEC gevraagd in haar advies op deze bepaling in de wet te wijzen. Voor niet-humane primaten geldt verder dat deze alleen in dierproeven mogen worden gebruikt wanneer zij nakomelingen zijn van niet-humane primaten die in gevangenschap zijn gefokt of afkomstig zijn uit zichzelf in stand houdende fokkolonies.

Dieren in/uit het wild:
Uit de Wod volgt dat dieren in/uit het wild in principe niet gebruikt mogen worden voor dierproeven (Artikel 10f Wod). Hier kan van worden afgeweken indien wetenschappelijk is onderbouwd waarom het doel van de proef alleen kan worden bereikt als gebruik gemaakt wordt van dieren in/uit het wild.

Voor dieren in/uit het wild is bovendien vastgelegd dat het vangen van de dieren plaats moet vinden door een deskundig persoon met methoden die bij dieren geen pijn, lijden, angst of blijvende schade veroorzaken die te vermijden zijn. Dieren die na de vangst gewond blijken te zijn of in slechte gezondheid verkeren, moeten worden onderzocht door een dierenarts of een andere ter zake deskundige persoon. Er dienen bovendien maatregelen te worden getroffen om de dieren zo min mogelijk te laten lijden. De DEC wordt gevraagd te beoordelen of dergelijke maatregelen voldoende beschreven staan in de aanvraag. Voor meer informatie over proeven met dieren in/uit het wild zie handreiking *Dierproeven met wilde dieren in hun biotoop*.

Zwerfdieren en verwilderde dieren:
Zwerfdieren en verwilderde dieren mogen in principe niet gebruikt worden voor dierproeven (Artikel 10h Wod). Hier kan alleen van worden afgeweken als er essentiële behoefte bestaat aan dierproeven op het terrein van de gezondheid of het welzijn van deze dieren of op het terrein van ernstige bedreigingen voor het milieu of de gezondheid van mens en dier. Bovendien moet door wetenschappelijke onderbouwing zijn aangetoond dat het doel van de proef alleen kan worden bereikt als gebruik gemaakt wordt van zwerfdieren en/of verwilderde dieren. De DEC wordt gevraagd om te beoordelen of voor de in de aanvraag beschreven doelstelling mag worden afgeweken van de wet en of is aangetoond dat voor het behalen van de doelstelling echt zwerfdieren en/of verwilderde dieren nodig zijn.

Niet gefokt voor dierproeven:
Ook dieren die behoren tot één van de soorten genoemd in bijlage I van richtlijn 2010/63/EU, maar niet speciaal voor het gebruik in dierproeven zijn gefokt (Artikel 11 Wod), mogen in principe niet worden gebruikt voor dierproeven. Het gaat hierbij om muizen, ratten, cavia's, konijnen, hamsters, gerbils, niet-humane primaten, katten, honden, zebravissen en kikkers. Van dit uitgangspunt kan alleen worden afgeweken indien wetenschappelijk is onderbouwd waarom het doel van de proef niet kan worden bereikt met dieren die wel gefokt zijn voor dierproeven. De DEC wordt gevraagd om te beoordelen of het voor het behalen van de doelstelling noodzakelijk is om dieren te gebruiken die niet gefokt zijn voor dierproeven.

<u>C9 Worden dieren gebruikt die al eerder in een dierproef zijn gebruikt?</u>

Het gebruiken van dieren die al eerder in een proef zijn gebruikt, is alleen onder specifieke voorwaarden toegestaan (Artikel 1e, lid 2 Wod). Hergebruik is in principe alleen toegestaan als het ongerief in zowel de voorgaande proef als de voorliggende proef maximaal matig is. Ook moet het eerder gebruikte dier volledig zijn hersteld van de voorgaande proeven. De DEC wordt gevraagd om te beoordelen of hieraan wordt voldaan. Alleen in uitzonderlijke situaties kan het worden toegestaan een dier opnieuw te gebruiken dat maximaal één keer is gebruikt in een dierproef die ernstig ongerief heeft veroorzaakt. De DEC beoordeelt bij dit onderdeel of het noodzakelijk is om dieren te hergebruiken die al eerder ernstig ongerief hebben onderdaan en betrekt dit bij de ethische afweging.

<u>C9 Worden handelingen uitgevoerd die in principe niet zijn toegestaan?</u>

Locatie: buiten instelling vergunninghouder:
Het is niet toegestaan proeven uit te voeren buiten de instelling van een vergunninghouder als deze locatie niet is aangemeld bij en beoordeeld door de NVWA (Artikel 10g Wod). Dit

valt buiten de bevoegdheid van de CCD. De DEC wordt gevraagd om dit op te merken in haar advies.

Geen toepassing verdoving/pijnbestrijding:
Het is in principe niet toegestaan om handelingen aan dieren uit te voeren zonder pijnbestrijding toe te passen als dit wel geïndiceerd is (Artikel 13 Wod). Dit om onnodig ongerief bij de dieren te voorkomen. Van dit uitgangspunt mag alleen worden afgeweken als door het toedienen van de pijnbestrijding het doel van de proef niet kan worden behaald of het toedienen van de pijnbestrijding meer ongerief veroorzaakt dan de handeling zelf. Als de aanvrager afwijkt van dit uitgangspunt, beoordeelt de DEC onder C9 of dit is toegestaan.

Dodingsmethode niet volgens bijlage IV van richtlijn 2010/63/EU:
Voor het doden van dieren geldt dat een methode gebruikt moet worden die staat beschreven in bijlage IV van de Europese richtlijn 2010/63/EU (Artikel (13c, lid 3 Wod). Als de aanvrager hiervan afwijkt, beoordeelt de DEC onder C19 of dit is toegestaan. Zie C19 voor meer informatie.

C10 Worden dieren conform de Europese richtlijn gehuisvest en verzorgd?

Het uitgangspunt in de wet is dat dieren gehuisvest en verzorgd worden op een wijze die minimaal voldoet aan de eisen in bijlage III van richtlijn 2010/63/EU. Afwijken hiervan mag alleen indien dit in voldoende mate wetenschappelijk is onderbouwd of om redenen van dierenwelzijn en diergezondheid (Artikel 7 Dierproevenbesluit 2014). Het is dus niet toegestaan af te wijken van de richtlijn om praktische en/of financiële redenen. Indien dit de opgegeven reden is, kan de aanvraag niet worden vergund. Als er sprake is van afwijkende huisvesting om wetenschappelijke redenen, beoordeelt de DEC of de doelstelling inderdaad niet kan worden behaald als de dieren conform de richtlijn worden gehuisvest. Bij afwijkende huisvesting om redenen van dierenwelzijn beoordeelt de DEC of inderdaad beter is voor het welzijn als het dier afwijkend gehuisvest wordt.

C11 Wat is de mate van ongerief?

Voor de ethische toetsing is het van belang dat de DEC scherp in beeld heeft waar de welzijnsaantasting bij het proefdier uit bestaat. Het ongerief wordt geclassificeerd als terminaal, licht, matig of ernstig ongerief. De ongeriefsclassificatie wordt bepaald aan de hand van de mate van pijn, lijden, angst en blijvende schade die een individueel dier tijdens de procedure naar verwachting zal ondervinden. Om dit te kunnen beoordelen moet voor elk van de dieren duidelijk zijn welke handelingen ze ondergaan, inclusief de duur van de handelingen, het maximale aantal herhalingen en de tijd tussen de handelingen. Het gaat hierbij niet alleen om de directe gevolgen van de handelingen die de dieren ondergaan, maar bijvoorbeeld ook om ongerief veroorzaakt door het type huisvesting, transport en aantasting van het fenotype. Ook is het van belang dat inzichtelijk is op welke wijze de aanvrager het ongerief en andere negatieve welzijnseffecten zal beperken. Verschillende handelingen tasten in verschillende mate het welzijn van proefdieren aan. Hierdoor kan het uiteindelijke ongerief voor de dieren door cumulatie hoger worden. Training kan er daarentegen voor zorgen dat het ongerief juist lager wordt. De DEC wordt gevraagd om te beoordelen of het cumulatieve ongerief correct is geclassificeerd en of alle factoren die bijdragen aan het ongerief zijn meegewogen door de aanvrager.

C12 Op welke wijze wordt de integriteit van de dieren aangetast?

Handelingen aan dieren leiden ertoe dat de heelheid van het dier (integriteit) wordt aangetast en/of dieren minder goed in staat zijn natuurlijk gedrag te vertonen. Dit hoeft niet direct gepaard te gaan met ongerief of slechtere gezondheid. Het is daarom voor de ethische afweging niet alleen van belang om naar de mate van ongerief te kijken, maar ook naar de aantasting van de integriteit.

De integriteit van een dier kan op verschillende manieren worden aangetast; fysiek, gedragsmatig en mentaal.

Fysiek:
Bij fysieke aantasting van de integriteit kan worden gedacht aan handelingen die het lichaam of de werking van het lichaam veranderen, zoals het couperen van staarten bij varkens, het verwijderen van een stukje snavel bij kippen, het veranderen van fysieke eigenschappen van dieren door te fokken op meer productie of nakomelingen of het genetisch veranderen van dieren.

Gedragsmatig:
De integriteit van een dier kan ook worden aangetast door geen ruimte te geven aan het natuurlijke gedrag van het dier. Hierbij kan gedacht worden aan varkens die niet kunnen wroeten, jonge dieren die niet bij de moeder kunnen drinken, het niet aanwezig zijn van verstopplekken voor prooidieren zoals muizen en individuele huisvesting bij groepsdieren als ratten.

Mentaal:
De integriteit van dieren kan ook mentaal worden aangetast. Hierbij kan gedacht worden aan het beperken van voor de dierproef ongewenst gedrag, zoals stressgevoeligheid, agressie en angst, door toepassing van specifieke fokstrategieën of kalmerende medicijnen.

De DEC wordt gevraagd om aan te geven op welke wijze de integriteit van de dieren wordt aangetast tijdens de dierproeven.

<u>C13 Zijn de humane eindpunten goed gedefinieerd?</u>

In de Wod staat dat de dood als eindpunt van een dierproef, waarbij dieren dood gevonden worden in de kooi, zoveel mogelijk dient te worden vermeden en vervangen door in een vroeg stadium vaststelbare, humane eindpunten (Artikel 13b lid 1). Door de toepassing van humane eindpunten kan onnodig lijden bij de dieren worden voorkomen. Als een humaan eindpunt wordt bereikt moet een dier uit de proef genomen worden. Het is niet altijd nodig het dier te doden. In sommige situaties kan het volstaan de pijn bij het dier op andere wijze weg te nemen of het dier weg te halen uit de stressvolle omgeving.

Humane eindpunten zijn primair in het belang van het dier. Een goede definitie van humane eindpunten is echter ook van belang voor de haalbaarheid van de doelstellingen; een dier in een te slechte toestand kan immers onbetrouwbare resultaten geven. Voor de ethische afweging is het daarom van belang te beoordelen of de humane eindpunten goed zijn gedefinieerd om onnodig ongerief bij de dieren te helpen voorkomen. Bij de beoordeling van humane eindpunten dient ook aan het wetenschappelijke eindpunt te worden gedacht. Het is immers niet gerechtvaardigd om dieren langer ongerief te laten ondergaan dan noodzakelijk voor het behalen van de doelstelling.

<u>C14-C16 Worden de 3 V’s voldoende geborgd?</u>

Het uitgangspunt van de Wod is dat dieren een intrinsieke waarde hebben. De intrinsieke waarde moet worden gerespecteerd. Daaruit volgt het “nee, tenzij”-beleid met betrekking tot het gebruik van proefdieren. Dit uitgangspunt houdt in dat dierproeven niet mogen worden uitgevoerd als de doelstelling ook kan worden bereikt anders dan door middel van een dierproef, of door middel van een dierproef waarbij minder dieren kunnen worden gebruikt of minder ongerief wordt berokkend (Artikel 10, eerste lid, onder a Wod).

Indien de doelstelling kan worden behaald zonder het gebruik van proefdieren mag de CCD geen vergunning verstrekken voor de desbetreffende dierproeven, ongeacht de uitkomst

van een eventuele schade-batenanalyse. Om dit te kunnen beoordelen, dient de aanvrager aangegeven te hebben welke proefdiervrije alternatieven zijn overwogen, en te onderbouwen waarom deze niet geschikt zijn voor het behalen van de doelstelling. De DEC wordt gevraagd te beoordelen of de doelstelling ook kan worden behaald door proefdiervrije alternatieven, ook in bredere context. Hierbij speelt uiteraard ook het dilemma rond de vraag wat zwaarder weegt: meer dieren met minder ongerief of minder dieren met meer ongerief?

De DEC gaat na of de aanvrager zich heeft ingespannen om het optimale aantal dieren vast te stellen met behoud van kwaliteit van onderzoek. Ofwel: kan het met minder dieren? Denk hierbij aan actuele statistische poweranalyses, combinaties van handelingen in één dier meewegend het cumulatieve ongerief voor dat dier) of het vergelijken van de linker- met de rechterzijde van een dier (waar dit wetenschappelijk verantwoord is) in plaats van het gebruiken van controlegroepen.

De DEC verzekert zich er daarnaast van dat de aanvrager al het mogelijke heeft gedaan en gedurende het project zal doen om mogelijk ongerief voor de proefdieren te identificeren, te beperken en waar mogelijk te voorkomen.

Bij wettelijk voorgeschreven onderzoek wordt vaak verwezen naar voorgeschreven richtlijnen. Ook bij het volgen van deze richtlijnen is er vaak echter wel degelijk ruimte voor het maken van keuzes. Het is daarom van belang te controleren of bij wettelijk voorgeschreven onderzoek alternatieve methoden gebruikt mogen worden zonder dieren, met minder dieren of met minder ongerief, en hierover een oordeel op te nemen in het DEC-advies.

C17 Wordt onnodige duplicatie voorkomen?

Uit de Europese richtlijn 2010/63/EU volgt dat onnodige duplicatie van voorgeschreven proeven dient te worden voorkomen. Bij de advisering over aanvragen voor wettelijk vereist onderzoek wordt de DEC daarom gevraagd te beoordelen of dit voldoende is geborgd.

C18 Worden zowel mannelijke als vrouwelijke dieren gebruikt?

Een ander aspect dat bij de beoordeling van vergunningen wordt meegewogen is het geslacht van de dieren die worden gebruikt. Indien in het project slechts gebruik wordt gemaakt van één geslacht, wordt de DEC gevraagd te beoordelen of de aanvrager het probleem van vermindering van proefdieren in voorraad gedood en translatie naar de mens voldoende heeft verkend en betrokken bij de proefopzet.

Bij projecten gericht op translatie naar de mens is het van belang te beoordelen of het (gezien het stadium waarin het project zich bevindt) noodzakelijk is de proeven met één geslacht uit te voeren of dat het noodzakelijk of wenselijk is de proeven met zowel mannelijke als vrouwelijke dieren uit te voeren. Indien de aanvrager kiest voor het gebruik van één geslacht dient de aanvrager dit goed te onderbouwen. Indien een aanvrager aangeeft beide geslachten te gebruiken, maar hiervoor dientengevolge meer dieren nodig heeft, is het in het kader van vermindering van belang te beoordelen of dit voor het behalen van de doelstelling echt nodig is, ook voor proeven gericht op toepassing in de mens. De DEC wordt gevraagd om de keuze van de aanvrager, om de dierproeven met één of twee geslachten uit te voeren, te beoordelen.

C19 Worden dieren gedood door middel van een passende methode die zo min mogelijk ongerief veroorzaakt?

In het kader van verfijning is het van belang dat voor het doden van dieren een voor de diersoort passende methode wordt gebruikt die zo min mogelijk ongerief en stress voor de

dieren oplevert (Artikel 13c, lid 2 Wod). Welke methodes gebruikt mogen worden volgt uit bijlage IV van de Europese richtlijn 2010/63/EU. Hier kan alleen van worden afgeweken als door middel van een wetenschappelijke onderbouwing is aangetoond dat de doelstelling niet kan worden behaald als een in de richtlijn genoemde methode wordt gebruikt. In bijlage IV van de richtlijn worden ook dodingsmethodes genoemd die alleen mogen worden toegepast als aan specifieke voorwaarden wordt voldaan. Denk hierbij bijvoorbeeld aan het doden van muizen door middel van decapitatie. Dit is alleen toegestaan als alle andere in de richtlijn genoemde methodes om wetenschappelijke redenen niet kunnen worden gebruikt. Van de DEC wordt gevraagd om te beoordelen of dieren gedood worden door middel van een passende methode uit de Europese richtlijn. Indien een methode wordt gebruikt waarvoor specifieke voorwaarden gelden, beoordeelt de DEC of aan deze voorwaarden wordt voldaan.

Als een aanvrager een methode wil gebruiken die niet wordt genoemd in de Europese richtlijn, maar die wel even humaan wordt geacht dan kan de NVWA hiervoor een ontheffing verstrekken. Dit valt buiten de bevoegdheid van de CCD. In die gevallen wordt de DEC gevraagd dit aan te geven in haar advies.

<u>C20 Is herplaatsing of hergebruik overwogen?</u>

De Wod biedt de mogelijkheid om proefdieren te herplaatsen na afloop van een dierproef (Artikel 13d). Hiervoor gelden de volgende voorwaarden: 1) De gezondheidstoestand van het dier moet het toelaten, 2) er mag geen gevaar zijn voor de volksgezondheid, diergezondheid of het milieu en 3) er moeten passende mogelijkheden worden genomen om het welzijn van de dieren te waarborgen. Daarnaast staat de Wod, onder voorwaarden, hergebruik van dieren toe (zie C9). Indien dieren gedurende het project om niet-wetenschappelijke redenen worden gedood, wordt de DEC gevraagd te beoordelen of herplaatsing of hergebruik van deze dieren mogelijk zou zijn.

<u>C21 Niet Technische Samenvatting</u>

De DEC wordt gevraagd te beoordelen of de NTS een evenwichtige weergave is van het project, en begrijpelijk is geformuleerd.

Stap III) Probleem wegen

Als inzicht is verkregen in de voor de beoordeling relevante feiten en waarden, kan gestart worden met stap III (probleem wegen). In het kader van transparantie en uniformiteit van de ethische afweging van dierproefgebruik wordt de DEC gevraagd een aantal aspecten van de afweging expliciet te benoemen in het DEC-advies:

<u>D1 De centrale morele vraag</u>

De centrale morele vraag is onder stap I (Probleem definiëren) vastgesteld en kan worden weergegeven onder D1.

<u>D2 Weging van de belangrijkste waarden die op het spel staan en onderbouwing van deze weging</u>

Van de DEC wordt verwacht dat zij voor de verschillende belanghebbenden de morele waarden waaraan tegemoetgekomen wordt, of die in het geding zijn, ten opzichte van elkaar weegt. Om dit proces te vergemakkelijken kan de DEC de belangrijkste belanghebbenden en de belangrijkste waarden die op het spel staan waarderen. De DEC kan dit bijvoorbeeld verwoorden in termen van een gering, een zeer beperkt, een beperkt , een reëel, of een groot voordeel of nadeel.

Het is van belang inzichtelijk te maken hoe de waardering van de waarden tot stand is gekomen (waarom wordt een waarde als een beperkt nadeel of juist als groot voordeel gewaardeerd?) en op welke wijze de haalbaarheid van het directe en uiteindelijke doel hierbij is betrokken. Bij de waardering van de waarden kan ook de waarschijnlijkheid dat (en de mate waarin) een specifieke waarde bevorderd of aangetast wordt, worden meegenomen. Als er bijvoorbeeld sprake is van een onzeker toekomstig resultaat en het dus zeer onwaarschijnlijk is dat een specifieke waarde op termijn bevorderd wordt, kent de CCD aan een dergelijk belang slechts een zeer beperkte waarde toe.

Bij de waardering en de weging van de belangen kan niet alleen rekening gehouden worden met de waarden die op het spel staan, maar ook met het feit dat specifieke categorieën dieren (zoals niet-humane primaten en dieren uit het wild) een aparte categorie vormen in de wet, vanwege onder meer een verhoogde welzijnsimpact van handelingen aan deze dieren.

De DEC wordt gevraagd aan te geven waarom zij van mening is dat bevordering van waarden voor de ene belanghebbende zwaarder weegt dan de aantasting van waarden voor de andere belanghebbende. Als hierover discussie is in de DEC, dient dit inzichtelijk te worden gemaakt.

DEC-leden kunnen vanuit verschillende perspectieven hun ethische overwegingen inbrengen en een afweging maken. Hieronder worden drie verschillende perspectieven toegelicht:

1) Voor sommige personen staat bij het maken van een ethische afweging de gevolgen van een handeling centraal. Het gaat dan niet om de handeling zelf of om de intenties van de handelende persoon, maar vooral om de gevolgen die de handeling heeft, zoals het nut, risico en toekomstige welzijnsaantasting. Zijn die gevolgen goed, dan is de handeling ook goed. Dit wordt ook wel gevolgenethiek (consequentialistische ethiek) genoemd.

2) Voor anderen is het van belang dat een handeling in lijn is met een geldende regel, zoals een waarde en de daaruit volgende norm of wet. Of een handeling positieve gevolgen heeft wordt dan minder meegewogen. Als een handeling niet aan een specifieke waarde voldoet, kan die vanuit dat perspectief moreel problematisch zijn. Dit wordt ook wel waardenethiek (deontologie, rechtsethiek, plichtsethiek) genoemd. Waarden zoals autonomie van de mens en intrinsieke waarde en integriteit van het dier worden hier vaak genoemd.

3) Een derde perspectief is de deugdethiek. Voor personen die vanuit dit perspectief denken, staan het karakter van een persoon en het ontwikkelen van goede karaktereigenschappen centraal, deugden als moed, geduld, wijsheid en eerlijkheid. Dit betekent dat de vraag of een handeling goed is niet bepaald wordt door het gevolg van de handeling, maar door de intenties en het karakter van de persoon die handelt. Dit kan aan de orde komen waar het gaat om het vertrouwen dat de aanvrager, ook op details, en waar relevant in overleg met de IvD, integere keuzes zal maken. Een tweede aspect van de deugdethiek is dat voor iedereen de basisvoorwaarden bereikbaar moeten zijn om te kunnen gedijen naar zijn of haar aard. Op proefdieren toegepast gaat dit vaak om het kunnen vertonen van soorteigen gedrag.

Als meerdere perspectieven naar voren komen tijdens een discussie over een aanvraag, is het goed om dit inzichtelijk te maken in het DEC-advies.

Het is voor de CCD niet mogelijk om op voorhand aan te geven hoe verschillende waarden gewogen worden, omdat de waardering van de verschillende belangen afhangt van meerdere factoren, zoals de achtergrond van het project, de mate van het effect, de waarden die voor elk van de belanghebbenden bevorderd worden of in geding zijn, de haalbaarheid van de doelstellingen, ontwikkelingen in de samenleving en voortschrijdend inzicht. De CCD heeft voor aanvragen t.b.v. de veehouderij een

uitvoeringsbeleid opgesteld waarin wordt aangegeven hoe de CCD specifieke doelstellingen waardeert (zie *Uitvoeringbeleid CCD Aanvragen t.b.v. veehouderij* op website CCD).

D3 Beantwoorden centrale morele vraag

Voor het beantwoorden van de centrale morele vraag maakt de DEC gebruik van bovenstaande afweging van waarden. Als uit deze afweging blijkt dat voor de DEC de waarden die bevorderd worden zwaarder wegen dan de waarden die voor de proefdieren in het geding zijn, betekent dit niet dat de centrale morele vraag ook automatisch met 'ja' moet worden beantwoord. Er zijn namelijk meer factoren die van belang zijn, zoals de punten die benoemd zijn onder stap II (Probleem analyseren). Het gaat dan onder meer om relevante wet- en regelgeving anders dan de Wod, de vraag of voldaan is aan wet- en regelgeving rond het gebruik van bijzondere categorieën dieren en/of handelingen en de toepassing van de 3 V's. Dit geldt ook voor ongerief en aantasting van integriteit, voor zover deze feiten niet al zijn meegenomen als waarde (zie stap II (Analyse van probleem)). Het is, in het kader van transparantie, van belang dat de DEC benoemt welke elementen zijn meegewogen bij de beantwoording van de centrale morele vraag en aangeeft hoe deze zijn meegewogen.

Het maken van een ethische afweging is niet altijd mogelijk. Dit kan bijvoorbeeld het geval zijn als er informatie ontbreekt waardoor het ongerief dat de dieren ondergaan, de haalbaarheid van de doelstelling en/of het belang van het project niet beoordeeld kunnen worden. De DEC kan de CCD dan eenmalig adviseren de aanvrager in de gelegenheid te stellen de aanvraag te herschrijven. Zie document *Adviestraject DEC – CCD* voor de handelswijze bij een aanvraag die niet te beoordelen is.

Stap IV) Advies

E1 Advies aan CCD

Als de centrale morele vraag is beantwoord, kan advies worden uitgebracht aan de CCD. Het is hierbij van belang dat het advies op logische wijze volgt uit de eerdere vragen in het Format DEC advies. Bij een negatief advies of het advies om de aanvraag deels of onder voorwaarden te vergunnen, zullen discussie- en knelpunten immers al bij de inventarisatie van de relevante feiten en de ethische afweging aan de orde zijn gekomen.

E2 Consensus of meerderheidsstandpunt

Het uitgebrachte advies kan op basis van consensus tot stand zijn gekomen, dan wel gebaseerd zijn op een meerderheidsstandpunt. Indien het advies gebaseerd is op een meerderheidsstandpunt is het, in het kader van transparantie, belangrijk om naast de weergave van argumenten voor het meerderheidsstandpunt ook de argumenten voor het minderheidsstandpunt weer te geven in het advies. De DEC wordt gevraagd het minderheidsstandpunt te specificeren op niveau van verschillende belanghebbenden van het project en de waarden die op het spel staan voor elk van de belanghebbenden. Voor de navolgbaarheid is het goed om ook aan te geven waarom de meerderheid van de DEC zich niet kan vinden in het minderheidsstandpunt.

E3 Dilemma's

Het is denkbaar dat tijdens het beoordelen van een aanvraag en het opstellen van het advies knelpunten en dilemma's naar voren komen, zowel binnen als buiten de context van het project, die de verantwoordelijkheid van de DEC overstijgen. Hierbij kan bijvoorbeeld gedacht worden aan doelen die gesteld worden in het licht van leefstijl-gerelateerde aandoeningen van de mens of doelen die bijdragen aan ontwikkelingen in de veehouderij.

Dilemma's kunnen zich ook voordoen wanneer de opbrengsten van wettelijk vereist onderzoek niet lijken op te wegen tegen de negatieve gevolgen voor de proefdieren (proportionaliteitsbeginsel). Hierbij kan gedacht worden aan de wettelijke eis bij registratie van stoffen om eerder uitgevoerd onderzoek te herhalen (zoals effectiviteitstesten van elke batch generieke geneesmiddelen ten opzichte van het referentiemiddel, of herhaling van onderzoek vanwege (kleine) verschillen tussen internationale richtlijnen). Tot slot kunnen dilemma's zich ook voordoen wanneer er alternatieven beschikbaar zijn, waardoor volgens de Wod de dierproeven niet uitgevoerd zouden mogen worden, maar de beschikbare alternatieven op basis van andere wettelijke bepalingen nog niet gezien worden als een passend alternatief (subsidiariteit).

De DEC wordt gevraagd in haar advies de discussie rondom dilemma's die de individuele aanvragen overstijgen, en raken aan meer fundamenteel ethische vraagstukken, te benoemen. Dit kan voor de CCD aanleiding zijn om de minister te vragen om te komen tot een breed gedragen maatschappelijk standpunt, of om het NCad of een andere instantie te vragen om, los van individuele vergunningsaanvragen, ten algemene een advies te geven hoe om te gaan met dergelijke dilemma's. Een dergelijk traject heeft bijvoorbeeld geleid tot het uitvoeringsbeleid voor aanvragen t.b.v. veehouderij.

Het is niet de bedoeling dat discussiepunten die van belang zijn voor de ethische afweging van de voorliggende aanvraag hier worden benoemd (zoals proefopzet, haalbaarheid, geschiktheid diermodel, transleerbaarheid model, inschatting ongerief, haalbaarheid van de doelstelling zonder gebruik van dieren). Dergelijke discussiepunten horen te worden benoemd bij de desbetreffende C-vraag en bij D3 (beantwoording centrale morele vraag). Alleen als het een vaker terugkerend of een aanvraag overschrijdend dilemma is, zou dit hier aangegeven moeten worden.
