# Supplementary material for: Mission impossible accomplished? A European cross-national comparative study on the integration of the harm-benefit analysis into law and policy documents
Source: PLoS One. 2024 Feb 20;19(2):e0297375. doi: 10.1371/journal.pone.0297375 (PMC10878508; doi:10.1371/journal.pone.0297375)

Vol. 12, N° 3, 2017

swiss academies
of arts and sciences

swiss academies
communications

www.swiss-academies.ch

Weighing of interests for proposed animal experiments

Guidance for applicants

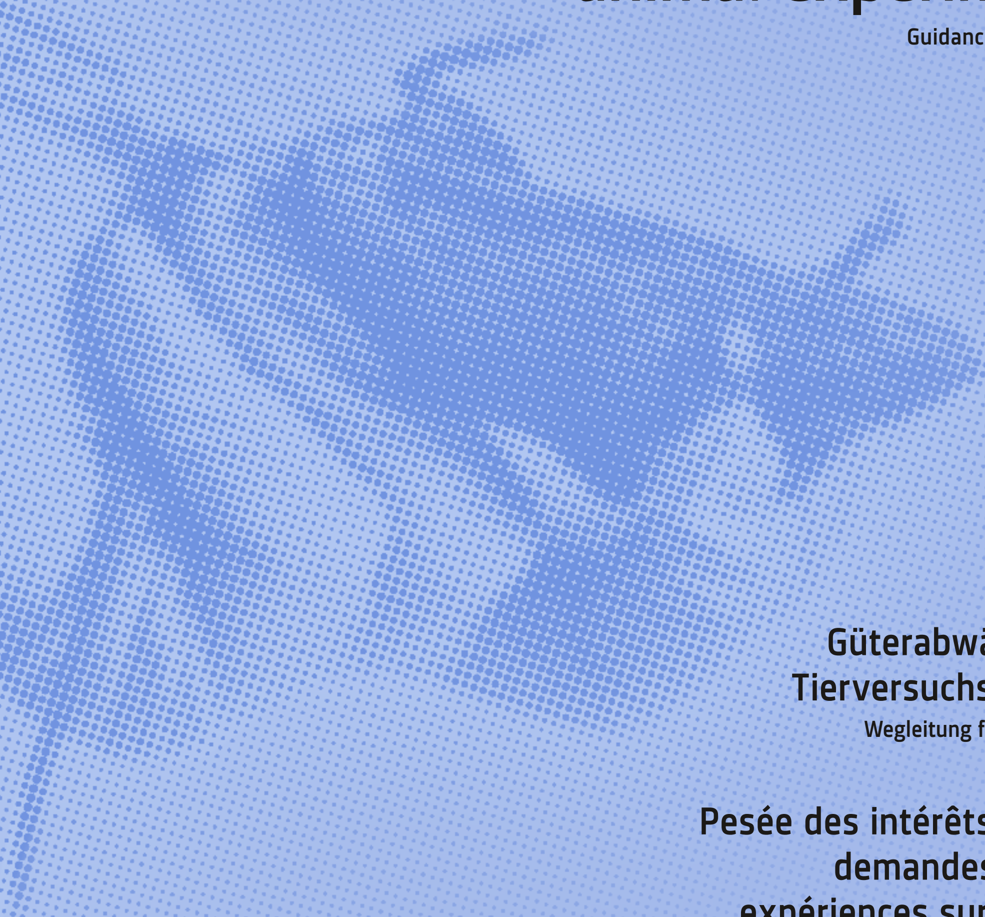

Güterabwägung bei Tierversuchsanträgen

Wegleitung für Antragsteller

Pesée des intérêts dans les demandes pour les expériences sur animaux

Guide destiné aux requérants

Issued by

Ethics Committee for Animal Experimentation (ECAE)
of the Swiss Academies of Arts and Sciences

Kommission für Tierversuchsethik (KTVE)
der Akademien der Wissenschaften Schweiz

Commission pour l'éthique dans les expérimentations
animales (CEEA) des Académies suisses des sciences

Haus der Akademien,
Laupenstrasse 7, CH-3001 Bern
www.swiss-academies.ch,
www.akademien-schweiz.ch,
www.academies-suisses.ch

Design
Howald Fosco Biberstein, Basel

Translation
The german text is the authentic version.
English: Jeff Acheson, Bottmingen
Français: Dominique Nickel, Bern

Cover photo
Stefan Schmidlin, Basel
The cover photo shows an intraperitoneal (IP) injection in the mouse,
a procedure frequently performed in animal experiments.

Printing
Kreis Druck, Basel

1st edition 2017 (2500)
Printed copies of this publication are available
free of charge – also in bulk – from: order@samw.ch

Copyright: ©2017 Swiss Academies of Arts and Sciences

This is an open-access publication distributed under
the terms of the Creative Commons attribution license
(http://creativecommons.org/licenses/by/4.0/),
which permits unrestricted use, distribution, and
reproduction in any medium, provided the original
author and source are credited.

Recommended form of citation:
Swiss Academies of Arts and Sciences (2017)
Weighing of interests for proposed animal experiments.
Guidance for applicants. Swiss Academies Communications 12 (3).

ISSN (print): 2297-8275 (English), 2297-1793 (Deutsch), 2297-1815 (français)
ISSN (online): 2297-184X (English), 2297-1807 (Deutsch), 2297-1823 (français)
DOI: http://doi.org/10.5281/zenodo.844378

e **Weighing of interests for proposed animal experiments** **3**
Guidance for applicants

d **Güterabwägung bei Tierversuchsanträgen** **17**
Wegleitung für Antragsteller

f **Pesée des intérêts dans les demandes pour les expériences sur animaux** **31**
Guide destiné aux requérants

e

d

f

Weighing of interests for proposed animal experiments

Guidance for applicants

e

Information on the preparation of this guidance

The Ethics Committee for Animal Experimentation (ECAE) is jointly operated by the Swiss Academy of Medical Sciences (SAMS) and the Swiss Academy of Sciences (SCNAT) and serves as an expert and advisory body in the area of animal experimentation, reviewing legislation, preparing opinions and issuing recommendations. The present guidance was developed to make it easier for applicants to carry out the weighing of interests for proposed animal experiments. The project – which was supported by animal welfare officers in higher education and industry, as well as the cantonal animal experimentation committees – was preceded by a careful evaluation process.

The following are members of the ECAE: Professor Rolf Zeller, Basel (Chair); Dr Beatrice Roth, Zurich (Vice Chair); lic. theol., Dipl.-Biol. Sibylle Ackermann, Bern (ex officio); Dr Christoph Ammann, Zurich; PD Dr Alessandra Bergadano, Basel; Dr Katharina Friedli, Ettenhausen; PD Dr Birgit Ledermann, Basel; Professor Christian Lüscher, Geneva; Professor Gregor Rainer, Fribourg; Dr Hans Sigg, Zurich; Professor Susanne E. Ulbrich, Zurich; Professor Brigitte von Rechenberg, Zurich; Professor Hanno Würbel, Bern.

The ECAE met on several occasions to consider the issues in depth. After detailed discussions and hearings with external specialists, the guidance was drafted by a group of authors (S. Ackermann, Ch. Ammann, A. Bergadano, B. Roth, H. Würbel) and submitted several times to the ECAE for review. Expert consultation procedures were held in German- and French-speaking Switzerland in the early summer and autumn of 2016 respectively. The comments received were taken into account in the final version.

1. Authorisation of animal experiments in Switzerland 6

2. Key element of authorisation: weighing of interests 6

3. Relationship between «dignity» and weighing of interests 7

4. The weighing of interests for proposed animal experiments – a multi-step procedure 8

4.1 Assessment of instrumental essentiality 8

4.2 Assessment of goal-related essentiality 9

4.2.1 Legitimate interests of society 10

4.2.2 Harms caused to animals 10

5. Step-by-step guide to the weighing of interests 13

Step 1: Description of research goal and gain in knowledge 13

Step 2: Description of the appropriateness and necessity of the experiment 13

Step 3: Enumeration of interests 14

Step 4: Weighting of the interests enumerated 15

Step 5: Weighing of interests 16

6. Sources and links 16

e

If, as a researcher, you submit an application to conduct an animal experiment in Switzerland, you are required under the Federal Act on Animal Protection (TSchG; SR 455) to consider ethical as well as scientific aspects. The ethical evaluation takes the form of a «weighing of interests». This guidance explains the principle of the weighing of interests for proposed animal experiments and should help you to carry out the procedure correctly.

1. Authorisation of animal experiments in Switzerland

Animal experiments in Switzerland are subject to stringent regulations. A justification is required whenever sentient animals are to be used for experimental purposes. As a researcher, you are responsible for providing such a justification in the form of a weighing of interests. Your application will be examined by the competent authority – generally, the Cantonal Veterinary Office, supported by a cantonal animal experimentation committee. In the course of the authorisation procedure, queries may be raised which necessitate revision of the application. In addition, an authorisation may be issued subject to certain conditions.

Experiments classified as «degree of severity 0» are not usually considered by the animal experimentation committee, but are reviewed directly by the competent authority. Research involving animals always requires a clear justification and – like the production or purchase and keeping of experimental animals – official authorisation.

2. Key element of authorisation: weighing of interests

A weighing of interests is carried out in legal or ethical conflict situations. These arise when it is not possible to give equal consideration to the mutually opposed interests at stake. In the case of animal experiments, certain *legitimate interests of society* (often expressed in terms of the *«expected gain in knowledge»*, e.g. new insights into fundamental biological processes) are opposed to the *protection of animals from harm* (e.g. pain, distress, degradation).

Which of the two sides is to be accorded greater weight has to be determined on a case-by-case basis by means of a weighing of interests. This establishes whether, in view of the expected gain in knowledge, it is appropriate to subject the animals in question to the harms envisaged.

The weighing of interests submitted as part of your application will be reviewed for plausibility, rationality and completeness by the animal experimentation committee and by the regulatory authority.

3. Relationship between «dignity» and weighing of interests

In Switzerland, the «dignity of living beings» has been enshrined in the Federal Constitution since 1992, and the «dignity of animals» in the TSchG since 2008. Here, it is important to note that while dignity includes animals' welfare (e.g. freedom from pain), it is not reducible to it. If the dignity of animals is to be respected, their independence and individual characteristics need to be taken seriously and any interventions need to be critically assessed and justified within the specified legal framework.

According to Art. 3, let. a of the TSchG, «The dignity of animals is not duly respected if they are subjected to harms that cannot be justified by preponderant interests.» The weighing of interests is the means used to determine whether or not a proposed experiment is compatible with the dignity of the animals concerned. If legitimate interests of society are found to be preponderant, then the animals' dignity is considered to be respected notwithstanding the harms caused to them. The dignity of animals is not duly respected if the societal interests adduced do not outweigh the harms caused.

e

«Dignity» as defined in the Animal Protection Act
In this Act, dignity means the inherent value of the animal, which is to be respected by anyone who handles it. The dignity of animals is not duly respected if they are subjected to harms that cannot be justified by preponderant interests – in particular, pain, suffering or injury, distress or degradation, profound alteration of their appearance or capacities, or excessive instrumentalisation. (Art. 3, let. a)

4. The weighing of interests for proposed animal experiments – a multi-step procedure

Under Art. 17 of the TSchG, «Experiments which may cause animals pain, suffering, injury or distress, substantially impair their general condition or otherwise violate their dignity are to be restricted to the essential minimum.» The aim of the authorisation procedure is to ensure that animal experiments are restricted to the «essential minimum».

Your responsibility as a researcher is to explain why your proposed experiment is to be considered «essential». Here, two types of «essentiality» are to be distinguished: the experiment must be essential both *instrumentally* and in terms of the goal pursued

It is always advisable to discuss these questions with your institution's animal welfare officer at an early stage.

Instrumental essentiality
Is the proposed experiment appropriate and necessary to achieve the research goal?

Goal-related essentiality
Is the goal of the experiment sufficiently important to justify the harms caused to the animals?

4.1 Assessment of instrumental essentiality

To obtain authorisation, you must be able to show that the proposed animal experiment is a suitable instrument for achieving the research goal. The *assessment* of instrumental essentiality involves two elements – the *appropriateness* and the *necessity* of the proposed experiment.

The *appropriateness* of the experiment depends on the extent to which the expected results are meaningful (i.e. robust, reproducible and generalisable) and enable the research question to be answered. The crucial factors are the selection of the most suitable animal model, sound operationalisation (i.e. measurability of the variables of interest), a suitable protocol and compliance with good scientific practice – e.g. with regard to randomisation, sample size calculation, statistical analysis and evaluation. Here, guidance can be obtained, for example, from the ARRIVE guidelines.

The *necessity* of the experiment depends on whether the requirements of the 3R principle (replacement, reduction, refinement) have been fully complied with. This is the case if the research goal cannot be achieved using alternative (non-animal) methods, or with phylogenetically lower species, fewer animals and/or less harmful interventions.

For the demonstration of instrumental essentiality, it is advisable to seek additional expertise, e.g. – depending on the type of experiment – in anaesthesia, pain relief or biometric planning.

4.2 Assessment of goal-related essentiality

The assessment of goal-related essentiality reflects the possibility that a proposed experiment – even if it is excellent according to scientific criteria – may be disproportionate and hence inadmissible. An experiment is considered essential in goal-related terms if the expected gain in knowledge (and thus ultimately the associated societal interest) appears to be so important that it outweighs the expected harms to the animals, and the experiment is therefore justified.

The «legitimate interests of society» thus need to be weighed in the scales against the «harms caused to animals». You should first identify the items to be placed on either side of the scales for your proposed experiment (cf. Sections 4.2.1 and 4.2.2) and then weight the various items as objectively and impartially as possible; the weighing of interests will yield either a favourable or an unfavourable outcome for your experiment (cf. Section 5).

Obviously, an application is only to be submitted if your weighing of interests indicates that the legitimate interests of society preponderate. The animal experimentation committee and the regulatory authority may accept your assessment of goal-related essentiality, or come to a different conclusion. The latter may be the case if there are doubts as to the correctness or relevance of the initial hypothesis or the research goal, or if the harms caused to the animals are deemed to be too severe – either relative to the goal pursued or in absolute terms (in the sense of an upper limit to acceptable suffering).

4.2.1 Legitimate interests of society

Experiments are only admissible if they serve legitimate interests of society. Under Art. 137 of the Animal Protection Ordinance (TSchV; SR 455.1), the following research goals can be regarded as legitimate interests:

- preservation or protection of the life and health of human beings or animals;
- new knowledge concerning fundamental biological processes;
- protection of the natural environment.

When writing your application, you should describe as realistically as possible the gain in knowledge which your experiment is expected to yield, avoiding speculative prospects. The crucial factor is the extent to which the proposed experiment can contribute to the attainment of one of the above mentioned research goals.

In assessing the importance of the experiment, it is helpful to apply the criteria defined by the major research funding institutions (e.g. SNSF, DFG) for the scientific peer review process, in particular with regard to:

- the expected gain in knowledge;
- the scientific importance for the research field and possibly other disciplines;
- the originality of the experiment;
- special significance for other (e.g. science or social policy) reasons.

4.2.2 Harms caused to animals

According to Art. 3 of the TSchG, harms are caused to animals «if they are subjected to … in particular, pain, suffering or injury, distress or degradation, profound alteration of their appearance or capacities, or excessive instrumentalisation.» The term «harms» thus covers not only interventions *experienced* as burdensome by animals (i.e. *pathocentric* harms) but also interventions where this need not be the case. Animal experiments generally involve harms in both of these categories.

For *pathocentric* harms, experiments are assigned to severity categories (degree of severity 0–3) depending on the occurrence and intensity of the individual criteria (pain, suffering, injury, distress). Here, a number of harms from the same or different categories (e.g. reduced food supply, repeated blood sampling, animal infection) are not added up to yield a higher degree of severity; the severity category reflects the degree of the most severe harm. Nonetheless, *cumulative harms* are to be taken into account in the weighing of interests: for example, if an experiment involving two category 2 harms is to be justified, it must yield a more important gain in knowledge than an experiment involving only one category 2 harm.

Pathocentric harms
relate to experiential criteria, adversely affecting animals' welfare. These include:
- pain,
- suffering,
- injury,
- distress.

Non-pathocentric harms
cannot necessarily be experienced as burdensome by animals, but respect for their dignity is nonetheless called into question. These include:
- excessive instrumentalisation,
- degradation,
- profound alteration of appearance or capacities.

The determination and description of *non-pathocentric* harms (excessive instrumentalisation, degradation, profound alteration of appearance or capacities) are clarified by the examples given in the Box on page 12.

It should be noted that while the term «excessive instrumentalisation» suggests prejudgement of the outcome of the weighing of interests, there are cases where it is concluded that excessive (in the sense of «substantial») instrumentalisation is in fact justified by important societal interests. In addition, excessive instrumentalisation and degradation may be two aspects of the same phenomenon. They occur when an animal's status as an independent being is not given due consideration, or an animal is used for alien purposes in an inconsiderate manner with no adequate justification. This is the case, for example, in situations where an animal suffers a total loss of control or undergoes a high degree of mechanisation.

Examples of non-pathocentric harms

Profound alteration of appearance or capacities

Nude mice are widely used as model organisms. Because of a genetic mutation, they lack a thymus and body hair. Appropriate measures can be taken to compensate for the animals' weak immune system and lack of hair (aseptic environment, higher ambient temperature, etc.), so that no pathocentric harms arise. However, the breeding of mice with this mutation does represent a profound alteration of the animals' appearance (hairlessness) and capacities (immune response). This is relevant for the assessment: an experiment involving nude mice which could be conducted with «normal» mice without sacrificing any gain in knowledge will be judged to be inadmissible in the weighing of interests – despite the absence of pathocentric harms – because there is no justification for the non pathocentric harms.

Excessive instrumentalisation and degradation

Parabiosis experiments are carried out, for example, in ageing and stem cell research. In these experiments, two animals (e.g. mice) are stitched together side by side and develop a shared circulatory system. For the animals, parabiosis involves pathocentric harms, but non-pathocentric harms are also taken into account in the assessment: the surgical joining of two animals terminates their status as independent beings and leads to a substantial loss of control, thus fulfilling the criteria for degradation and excessive instrumentalisation.

5. Step-by-step guide to the weighing of interests

Your application to conduct an animal experiment should be submitted online via e-TV. To obtain access, you have to complete «Form-A: Application for licence to perform animal experiments»; in the final section (63), you are requested to carry out the weighing of interests.

Step 1

Description of research goal and gain in knowledge

Using the information you provided in sections 44.1 and 44.2, summarise the aim of the project and the expected gain in knowledge.

Suggested wording

The animal experiment described here has a goal considered legitimate under Art. 137 of the TSchV … (indicate the general research goal)
It is designed to fill the following gap in scientific knowledge … (describe the knowledge sought in itself)
It is expected to yield the following concrete gains in knowledge … (indicate the expected benefits of the knowledge sought)

Step 2

Description of the appropriateness and necessity of the experiment (demonstration of instrumental essentiality)

In the second step, you have to show that the proposed experiment is both appropriate and necessary to achieve the research goal:

Step 2.1 Appropriateness of the experiment

Using the information you provided in sections 51.1–51.3 and 54.3–55, explain the scientific validity of your experiment by showing that
- your approach is an appropriate way of achieving the specified research goal;
- the method chosen can be expected to deliver meaningful and reproducible results.

e

Suggested wording

The proposed experiment outlined here is appropriate for achieving the specified research goal because … (emphasise the quality of the approach adopted: validity of animal model, suitable control groups, specific expertise and experience of project leaders, etc.)
It can be expected to deliver meaningful and reproducible results because … (indicate measures taken to avoid bias: randomisation, blinding etc.)

Step 2.2 Necessity of the experiment

Explain why you consider your experiment to be necessary in the planned form, and why it cannot be conducted without using animals, or with fewer animals or less harmful interventions.

Suggested wording

The 3R criteria (replacement, reduction, refinement) are
met: replacement is not possible, as an examination of alternative methods indicated that …
(if appropriate, give details of a systematic literature review of alternative methods)
A reduction in the number of animals is not appropriate because … (give details of biometric planning)
Refinement has been pursued by … (specific information on all refinements); a further reduction of harms is not possible because … (specific justification for all remaining harms)

Step 3

Enumeration of interests

Compile a list of the relevant items on both sides of the scales for the weighing of interests:

«Legitimate interests of society»: Indicate the expected gain in knowledge and its importance for the research field and show that your research goal reflects at least one of the legitimate interests specified in the legislation.

Suggested wording

The expected gain in knowledge lies in the achievement of … It is important for the research field because … This is in accordance with the societal interest(s) legally recognised as
legitimate …

«Harms caused to animals»: Using the information you provided in sections 56.1–56.4, list the harms caused to the animals, and indicate whether and to what extent cumulative harms occur. Also mention the measures used to control pain (sections 53.1–53.2).

Suggested wording

The animals used in the experiment can be expected to experience the following harmful effects … (list of all pathocentric and non-pathocentric harms)

Items listed under
«Legitimate interests of society»

All the legitimate interests on the part of society applicable in this particular case (from the three categories: preservation or protection of the life and health of human beings or animals, new knowledge concerning fundamental biological processes, protection of the natural environment).

Items listed under
«Harms caused to animals»

All the harms arising in this particular case, both pathocentric (pain, suffering, injury, distress) and non-pathocentric (excessive instrumentalisation, degradation, profound alteration of appearance or capacities).

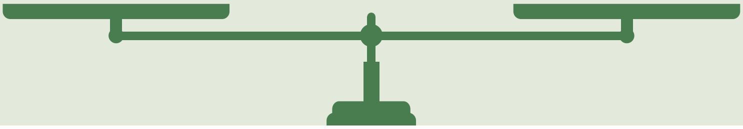

Step 4

Weighting of the interests enumerated

In this step, you explain clearly what weight is to be accorded to each item listed on either side of the scales, using, for example, the categories low/moderate/high/very high, or the classification (* to ****) proposed in the FSVO Guide to the weighing of interests.

Suggested wording

The harms caused to the animals in the experiment described here are to be rated as follows … (e.g. pain: high)
The weighting of the societal interests is based on … (e.g. knowledge concerning protection of the natural environment in the area of … is of e.g. high relevance for …).

Step 5

Weighing of interests (demonstration of goal-related essentiality)

Finally, explain clearly why your weighing of interests yields an outcome favourable for the proposed experiment.

Suggested wording

The weighting of the relevant items means that, compared to the expected gain in knowledge, the harms are ...

By carrying out the weighing of interests correctly, you have fulfilled your legal responsibility: you are submitting an application to conduct an animal experiment in which legitimate societal interests outweigh the harms caused to the animals – i.e. the experiment is proportionate and the dignity of animals is respected.

6. Sources and links

3R Research Foundation Switzerland: www.forschung3r.ch

Animal Protection Act (SR 455): www.admin.ch/opc/de/classified-compilation/20022103/index.html

Animal Protection Ordinance (SR 455.1): www.admin.ch/opc/de/classified-compilation/20080796/index.html

ARRIVE guidelines: www.nc3rs.org.uk/arrive-guidelines

Form-A: www.blv.admin.ch/blv/en/home/tiere/tierversuche/forschende.html

Guide to the weighing of interests/Explanatory notes on the weighing of interests, issued by the Federal Food Safety and Veterinary Office (FSVO): www.blv.admin.ch/blv/en/home/tiere/tierversuche/schweregrad-gueterabwaegung.html

Information sheet (in German) on the weighing of interests, issued by the Joint Animal Experimentation Committee of the Cantons of Basel-Stadt, Basel-Landschaft and Aargau: www.ethz.ch/en/research/ethics-and-animal-welfare/animal-welfare/legal-requirements/ethical-benefit-evaluation.html

The dignity of animals and the evaluation of interests in the Swiss Animal Protection Act. A position paper issued by the Ethics Committee for Animal Experimentation of the Swiss Academies of Arts and Sciences (2010): www.akademien-schweiz.ch/en/index/Portrait/Kommissionen-AG/Kommission-fuer-Tierversuchsethik.html

Güterabwägung bei Tierversuchsanträgen

Wegleitung für Antragsteller

d

Hinweise zur Ausarbeitung dieser Wegleitung

Die Kommission für Tierversuchsethik (KTVE), eine gemeinsame Kommission der Schweizerischen Akademie der Medizinischen Wissenschaften (SAMW) und der Akademie der Naturwissenschaften Schweiz (SCNAT), ist als Expertise- und Beratungsorgan im Bereich «Tierversuche» angelegt und prüft gesetzliche Erlasse, verfasst Stellungnahmen und Empfehlungen. Die vorliegende Wegleitung wurde erarbeitet, um den Antragstellern von Tierversuchen die Durchführung der Güterabwägung zu erleichtern. Dem Entscheid ging ein sorgfältiger Evaluationsprozess voraus. Das Vorhaben wurde von den Tierschutzbeauftragten der Universitäten und der Industrie sowie den kantonalen Tierversuchskommissionen begrüsst.

Folgende Persönlichkeiten gehören der Kommission für Tierversuchsethik (KTVE) an: Prof. Rolf Zeller, Basel (Präsident); Dr. Beatrice Roth, Zürich (Vizepräsidentin); lic. theol., Dipl.-Biol. Sibylle Ackermann, Bern (ex officio); Dr. Christoph Ammann, Zürich; PD Dr. Alessandra Bergadano, Basel; Dr. Katharina Friedli, Ettenhausen; PD Dr. Birgit Ledermann, Basel; Prof. Christian Lüscher, Genf; Prof. Gregor Rainer, Fribourg; Dr. Hans Sigg, Zürich; Prof. Susanne E. Ulbrich, Zürich; Prof. Brigitte von Rechenberg, Zürich; Prof. Hanno Würbel, Bern.

Die KTVE hat sich in mehreren Sitzungen vertieft mit der Thematik auseinandergesetzt. Nach eingehenden Diskussionen und der Anhörung externer Fachpersonen hat eine Autorengruppe (S. Ackermann, Ch. Ammann, A. Bergadano, B. Roth, H. Würbel) die Wegleitung verfasst und der KTVE wiederholt zur Diskussion unterbreitet. Im Frühsommer respektive im Herbst 2016 wurde eine Expertenvernehmlassung durchgeführt in der Deutschschweiz und der Romandie. Die eingegangenen Stellungnahmen sind in der Endfassung berücksichtigt.

1. Bewilligung von Tierversuchen in der Schweiz 20

2. Kernstück der Bewilligung: die Güterabwägung 20

3. Zum Verhältnis von «Würde» und Güterabwägung 21

4. Die Güterabwägung bei Tierversuchsanträgen – ein mehrstufiges Verfahren 22

4.1 Prüfung der instrumentellen Unerlässlichkeit 22

4.2 Prüfung der finalen Unerlässlichkeit 23

4.2.1 Schutzwürdige Interessen der Gesellschaft 24

4.2.2 Belastungen der Tiere 24

5. Durchführen der Güterabwägung Schritt für Schritt 27

Schritt 1: Darstellung Versuchsziel und Erkenntnisgewinn 27

Schritt 2: Darstellung Eignung und Erforderlichkeit Ihres Versuchs 27

Schritt 3: Auflistung der Güter 28

Schritt 4: Gewichtung der aufgelisteten Güter 29

Schritt 5: Abwägung der Güter 30

6. Quellen und Links 30

d

Wenn Sie als Forscherin oder Forscher in der Schweiz einen Antrag für einen Tierversuch stellen, müssen Sie gemäss Schweizerischem Tierschutzgesetz (TSchG) neben wissenschaftlichen auch «ethische» Erwägungen vornehmen und nachvollziehbar darlegen. Letzteres geschieht durch eine «Güterabwägung». Diese Wegleitung erläutert das Prinzip der Güterabwägung bei Tierversuchsanträgen und soll Sie dabei unterstützen, diese korrekt durchzuführen.

1. Bewilligung von Tierversuchen in der Schweiz

Tierversuche sind in der Schweiz streng geregelt. Jede Verwendung empfindungsfähiger Tiere zu Versuchszwecken bedarf grundsätzlich einer Rechtfertigung. Als Forscherin oder Forscher ist es Ihre Aufgabe, diese in Form einer Güterabwägung zu liefern. Ihr Antrag wird von der zuständigen Behörde geprüft. Dies ist in der Regel das kantonale Veterinäramt, welches von einer kantonalen Tierversuchskommission unterstützt wird. Im Verlauf des Bewilligungsverfahrens können Rückfragen gestellt werden, die eine Überarbeitung des Antrags erfordern. Ferner kann eine Bewilligung Ihres Antrags an Auflagen gebunden sein.

Versuche mit Schweregrad Null (SG0) kommen in der Regel nicht vor die Tierversuchskommission, sondern werden direkt von der zuständigen Behörde geprüft. Forschung mit Tieren bedarf immer einer nachvollziehbaren Rechtfertigung und – wie auch die Produktion bzw. Anschaffung und Haltung von Versuchstieren – einer behördlichen Bewilligung.

2. Kernstück der Bewilligung: die Güterabwägung

Eine Güterabwägung wird in einer rechtlichen oder ethischen Konfliktsituation durchgeführt. Eine solche liegt vor, wenn auf beiden Konfliktseiten schutzwürdige Güter auf dem Spiel stehen, die nicht gleichermassen berücksichtigt werden können. Im Fall von Tierversuchen sind es *bestimmte schutzwürdige Interessen der Gesellschaft* (oft *«angestrebter Erkenntnisgewinn»* genannt, z.B. neue Kenntnisse über grundlegende Lebensvorgänge), die dem *Schutz der Tiere vor Belastungen* (z.B. Schmerzen, Angst, Erniedrigung) entgegenstehen.

Welche der beiden Seiten stärker zu gewichten ist, muss in jedem einzelnen Fall durch eine Güterabwägung eruiert werden. Diese klärt, ob es angesichts des angestrebten Erkenntnisgewinns angemessen ist, die vorgesehenen Tiere den geplanten Belastungen auszusetzen.

Die von Ihnen vorgelegte Güterabwägung wird von der Tierversuchskommission und von der Bewilligungsbehörde auf ihre Plausibilität, Rationalität und Vollständigkeit geprüft.

3. Zum Verhältnis von «Würde» und Güterabwägung

In der Schweiz wurden 1992 die «Würde der Kreatur» in der Bundesverfassung und 2008 die «Würde des Tieres» im TSchG verankert. Dabei ist wichtig zu beachten, dass die Würde das Wohlergehen der Tiere (z.B. Schmerzfreiheit) umfasst, jedoch nicht auf dieses reduziert werden kann. Die Achtung der Würde bedingt, das Tier in seiner Eigenständigkeit und Eigenart ernst zu nehmen und alle Handlungen an Tieren kritisch zu hinterfragen und im gesetzlich vorgegebenen Rahmen zu rechtfertigen.

Das TSchG hält in Art. 3, Bst. a fest: «Die Würde des Tieres wird missachtet, wenn eine Belastung des Tieres nicht durch überwiegende Interessen gerechtfertigt werden kann.» Die Güterabwägung ist das Mittel um festzustellen, ob ein Versuchsvorhaben mit der Würde der betroffenen Tiere vereinbar ist oder nicht. Ergibt die Güterabwägung, dass schutzwürdige Interessen der Gesellschaft überwiegen, gilt die Würde der Tiere trotz der den Tieren entstehenden Belastungen als geachtet. Eine Missachtung der Würde des Tieres liegt vor, wenn die ins Feld geführten Interessen der Gesellschaft die Belastungen der Tiere nicht überwiegen.

Der «Würde-Begriff» im Tierschutzgesetz

«In diesem Gesetzt bedeute[t] Würde: Eigenwert des Tieres, der im Umgang mit ihm geachtet werden muss. Die Würde des Tieres wird missachtet, wenn eine Belastung des Tieres nicht durch überwiegende Interessen gerechtfertigt werden kann. Eine Belastung liegt vor, wenn dem Tier insbesondere Schmerzen, Leiden oder Schäden zugefügt werden, es in Angst versetzt oder erniedrigt wird, wenn tief greifend in sein Erscheinungsbild oder seine Fähigkeiten eingegriffen oder es übermässig instrumentalisiert wird.» (Art 3, Bst. a)

d

4. Die Güterabwägung bei Tierversuchsanträgen – ein mehrstufiges Verfahren

Das TSchG schreibt in Art. 17 vor: «Tierversuche, die dem Tier Schmerzen, Leiden oder Schäden zufügen, es in Angst versetzen, sein Allgemeinbefinden erheblich beeinträchtigen oder seine Würde in anderer Weise verletzen können, sind auf das unerlässliche Mass zu beschränken.» Das Ziel des Bewilligungsverfahrens liegt darin, Tierversuche auf das «unerlässliche Mass» zu beschränken.

Ihre Aufgabe als Forscherin bzw. Forscher ist es darzulegen, warum Sie Ihren Tierversuch als «unerlässlich» betrachten. Dabei sind zwei Arten von «Unerlässlichkeit» zu unterscheiden: Ihr Versuch muss sowohl *instrumentell* wie auch *final* unerlässlich sein.

Eine frühzeitige Besprechung mit dem bzw. der Tierschutzbeauftragten Ihrer Institution empfiehlt sich in jedem Fall.

Instrumentelle Unerlässlichkeit
Ist das Versuchsvorhaben geeignet und erforderlich, um das angestrebte Versuchsziel zu erreichen?

Finale Unerlässlichkeit
Ist das Versuchsziel (ge)wichtig genug, um die Belastungen der Tiere zu rechtfertigen?

4.1 Prüfung der instrumentellen Unerlässlichkeit

Für das Erlangen einer Bewilligung müssen Sie darlegen können, dass der geplante Tierversuch das geeignete «Instrument» ist, um Ihr Versuchsziel zu erreichen. Bei der Prüfung der instrumentellen Unerlässlichkeit geht es konkret um zwei Aspekte, die *Eignung* und die *Erforderlichkeit* Ihres Versuchsvorhabens.

Die *Eignung* Ihres Versuchs hängt davon ab, wie aussagekräftig (d.h. wie belastbar, reproduzierbar und generalisierbar) die zu erwartenden Ergebnisse sind und wie umfassend sich Ihre Fragestellung damit beantworten lässt. Ausschlaggebend sind die Wahl des am besten geeigneten Tiermodells, eine gute Operationalisierung (d.h. wie die Fragestellung messbar gemacht wird), ein geeigneter Versuchsplan, sowie die Kriterien der guten wissenschaftlichen Praxis u.a. bezüglich Randomisierung, Stichprobenberechnung, statistischer Auswertung und Evaluationsplan. Orientierung bieten z.B. die Inhalte der ARRIVE-Guidelines.

Die *Erforderlichkeit* Ihres Versuchs hängt davon ab, ob Sie die Anforderungen des 3R-Prinzips (Replace, Reduce, Refine) umfassend berücksichtigt haben. Dies ist der Fall, wenn das Versuchsziel nicht mit tierversuchsfreien Methoden, an evolutiv niedriger stehenden Tierarten, mit weniger Tieren und/oder mit weniger belastenden Methoden zu erreichen ist.

Für die Darlegung der instrumentellen Unerlässlichkeit empfehlen wir Ihnen das Einholen zusätzlicher Expertise, je nach Art Ihres Versuchs etwa in Anästhesie, Analgesie oder biometrischer Planung.

4.2 Prüfung der finalen Unerlässlichkeit

Die Prüfung der finalen Unerlässlichkeit reflektiert die Möglichkeit, dass Ihr Versuchsvorhaben – selbst wenn es nach wissenschaftlichen Kriterien exzellent ist – unverhältnismässig und daher unzulässig sein könnte. Ihr Versuch gilt als final unerlässlich, wenn der zu erwartende Erkenntnisgewinn (und damit letztlich das damit verbundene gesellschaftliche Interesse) so gewichtig erscheint, dass er die zu erwartenden Belastungen der Tiere überwiegt und der Versuch dadurch gerechtfertigt ist.

Hierbei werden also die Inhalte der beiden Waagschalen «schutzwürdige Interessen der Gesellschaft» und «Belastungen der Tiere» gegeneinander abgewogen. Dazu benennen Sie, welche Güter in Ihrem Versuch in den beiden Waagschalen liegen (vgl. Kap 4.2.1 und 4.2.2). Anschliessend gewichten Sie die einzelnen Güter möglichst objektiv und unvoreingenommen, was zum Ausgang der Güterabwägung zu Gunsten oder Ungunsten Ihres Versuchs führt (vgl. Kap. 5).

Natürlich reichen Sie als Antragsteller bzw. Antragstellerin Ihr Gesuch nur ein, wenn Ihre Güterabwägung zu Gunsten der schutzwürdigen gesellschaftlichen Interessen ausfällt. Die Tierversuchskommission und die Bewilligungsbehörde können Ihrer Einschätzung der finalen Unerlässlichkeit folgen oder zu einem anderen Schluss kommen. Letzteres ist der Fall, wenn Zweifel an der Richtigkeit oder Relevanz der Ausgangshypothese oder des Versuchsziels bestehen, oder wenn die den Tieren zugefügten Belastungen als zu gravierend eingeschätzt werden, entweder relativ zum angestrebten Versuchsziel oder absolut (im Sinne einer Obergrenze des zumutbaren Leidens).

4.2.1 Schutzwürdige Interessen der Gesellschaft

Ihr Versuch ist nur zulässig, wenn er schutzwürdigen gesellschaftlichen Interessen dient. Laut Tierschutzverordnung (TSchV Art. 137) können folgende Versuchsziele als schutzwürdige Interessen zur Geltung gebracht werden:

- Erhaltung oder Schutz des Lebens und der Gesundheit von Mensch und Tier;
- neue Kenntnisse über grundlegende Lebensvorgänge;
- Schutz der natürlichen Umwelt.

Beim Verfassen Ihres Antrags sollten Sie möglichst realistisch den tatsächlich zu erwartenden Erkenntnisgewinn Ihres Versuchs beschreiben und von spekulativen Ausblicken absehen. Entscheidend ist, inwiefern der geplante Versuch einen Beitrag zur Erreichung eines der oben genannten Forschungsziele leisten kann.

Hilfreich für die Einschätzung der Bedeutung des Versuchs ist der Rückgriff auf Kriterien des wissenschaftlichen Begutachtungsprozesses (Peer Review), gemäss den massgebenden Forschungsförderungsinstitutionen (z. B. SNF, DFG) insbesondere auf:

- den erwarteten Erkenntnisgewinn;
- die wissenschaftliche Bedeutung für das Forschungsgebiet und evtl. weitere Disziplinen;
- die Originalität des Versuchs;
- eine besondere Bedeutung aus anderen Gründen, z. B. wissenschafts- oder gesellschaftspolitische.

4.2.2 Belastungen der Tiere

Belastungen der Tiere liegen gemäss Art. 3 des TSchG vor, «wenn dem Tier insbesondere Schmerzen, Leiden oder Schäden zugefügt werden, es in Angst versetzt oder erniedrigt wird, wenn tief greifend in sein Erscheinungsbild oder seine Fähigkeiten eingegriffen oder es übermässig instrumentalisiert» wird. Unter den Begriff Belastung fallen also sowohl Eingriffe, die vom Tier als belastend *erlebt* werden (= *pathozentrische* Belastungen), als auch solche, bei denen dies nicht der Fall zu sein braucht. In einem Tierversuch kommen meist Belastungen aus beiden Kategorien vor.

Für die *pathozentrischen* Belastungen wird gemäss Auftreten und Ausprägungen der einzelnen Kriterien (Schmerzen, Leiden, Schäden, Angst) eine Schweregradeinteilung (SG0 – SG3) des Versuchs vorgenommen. Dabei werden mehrere Belastungen aus derselben oder unterschiedlichen Schweregrad-Kategorien (z. B. Futterreduktion, wiederholte Blutentnahme, Infektion des Tiers) nicht zu einem höheren Schweregrad aufaddiert; der Schweregrad eines Versuchs entspricht dem Grad der schwerwiegendsten Belastung. Trotzdem sind bei der Güterabwägung *kumulative Belastungen* zu beachten: Damit z. B. ein Versuch gerechtfertigt werden kann, der zwei Belastungen der Kategorie SG2 erfordert, muss er einen gewichtigeren Erkenntnisgewinn erbringen als ein Versuch, der nur eine SG2-Belastung beinhaltet.

Pathozentrische Belastungen
beziehen sich auf erlebensrelevante Kriterien, die das Wohlergehen der Tiere beeinträchtigen. Dazu gehören:
- Schmerzen,
- Leiden,
- Schäden,
- Angst.

Nicht-pathozentrische Belastungen
sind für das Tier nicht unbedingt als Belastung erfahrbar, stellen aber dennoch die Achtung der Würde in Frage. Dazu gehören:
- übermässige Instrumentalisierung,
- Erniedrigung,
- tief greifende Eingriffe in Erscheinungsbild oder Fähigkeiten.

Zur Verdeutlichung der Erfassung und Beschreibung der *nicht-pathozentrischen* Belastungen (übermässige Instrumentalisierung, Erniedrigung, tief greifende Eingriffe in Erscheinungsbild oder Fähigkeiten) finden Sie anbei Beispiele (vgl. Kasten auf Seite 26).

Zu beachten ist, dass der Begriff «übermässige Instrumentalisierung» eine Vorwegnahme des Ausgangs der Güterabwägung suggeriert; es gibt jedoch Fälle, in denen die Güterabwägung ergibt, dass eine übermässige (im Sinne von «erhebliche») Instrumentalisierung durch gewichtige gesellschaftliche Interessen gerechtfertigt ist. Übermässige Instrumentalisierung und Erniedrigung können zudem zwei Aspekte desselben Phänomens darstellen. Sie liegen vor, wenn dem Status des Tieres als eigenständiges Lebewesen nicht Rechnung getragen wird oder es in rücksichtsloser, nicht ausreichend begründbarer Weise fremden Zwecken unterworfen wird. Dies gilt etwa in Situationen eines totalen Kontrollverlusts für das Tier oder wenn Tiere hochgradig mechanisiert werden.

d

Beispiele für nicht-pathozentrische Belastungen

Tief greifende Eingriffe in Erscheinungsbild und Fähigkeiten

Nacktmäuse (nude mice) werden verbreitet als Modellorganismen eingesetzt. Diesen fehlen aufgrund einer Mutation der Thymus und das Haarkleid. Das schwache Immunsystem und das fehlende Fell können durch geeignete Massnahmen (keimfreie Umgebung, höhere Raumtemperaturen etc.) kompensiert werden, so dass den Tieren keine pathozentrischen Belastungen entstehen. Die Zucht solchermassen mutierter Mäuse bedeutet allerdings einen *tief greifenden Eingriff ins Erscheinungsbild* (Haarlosigkeit) und die *Fähigkeiten* (Immunantwort) der Mäuse. Dies ist für die Beurteilung relevant: Ein Versuch mit Nacktmäusen, der ohne Verlust an Erkenntnisgewinn mit «normalen» Mäusen durchgeführt werden kann, wird in der Güterabwägung trotz Abwesenheit pathozentrischer Belastungen als unzulässig beurteilt, weil die nicht-pathozentrischen Belastungen keine Rechtfertigung erhalten.

Übermässige Instrumentalisierung und Erniedrigung

Parabiose-Experimente werden u.a. in der Alters- und Stammzellforschung eingesetzt. Dabei werden zwei Tiere (z.B. Mäuse) entlang der Flanke zusammengenäht und der Blutkreislauf der Tiere verbunden. Parabiose ist mit pathozentrischen Belastungen für die Tiere verbunden, bei der Abwägung fallen aber auch nicht-pathozentrische Belastungen ins Gewicht: Das Zusammennähen zweier Tiere beendet den Status der Tiere als eigenständige Lebewesen und führt in hohem Masse zu Kontrollverlust; wodurch die Kriterien der Erniedrigung und übermässigen Instrumentalisierung erfüllt sind.

5. Durchführen der Güterabwägung Schritt für Schritt

Ihren Tierversuchsantrag reichen Sie mit Hilfe der Webapplikation e-Tierversuche ein (um einen Zugang zu erhalten, müssen Sie das Formular «Form-A: Gesuch für Tierversuche» ausfüllen). Unter Ziffer 63 werden Sie am Schluss um die Durchführung der Güterabwägung gebeten.

Schritt 1

Darstellung Versuchsziel und Erkenntnisgewinn

Stellen Sie unter Rückgriff auf Ihre Formulierungen in den Paragraphen 44.1 und 44.2 summarisch das Versuchsziel und den angestrebten Erkenntnisgewinn dar.

Formulierungsvorschlag

Der hier beschriebene Tierversuch dient dem gemäss TSchV Art. 137 zulässigen Versuchsziel … (= Nennung des allgemeinen Versuchsziels)
Er wurde geplant, um folgende Forschungslücke zu schliessen … (= Beschreibung der angestrebten Erkenntnis an sich)
Folgende konkrete Erkenntnisgewinne werden dadurch angestrebt … (= Nennung des erwarteten Nutzens der angestrebten Erkenntnis)

Schritt 2

Darstellung Eignung und Erforderlichkeit Ihres Versuchs (Nachweis der instrumentellen Unerlässlichkeit)

Im zweiten Schritt stellen Sie dar, dass Ihr Versuchsvorhaben geeignet und erforderlich ist, um das angestrebte Versuchsziel zu erreichen. Dazu sind zwei Aspekte nötig:

Schritt 2.1 Eignung Ihres Versuchs

Stellen Sie unter Rückgriff auf §51.1 – §51.3 und §54.3 – §55 die wissenschaftliche Aussagekraft Ihres Versuchs dar, indem Sie zeigen,
– dass Ihr Versuchsansatz geeignet ist zur Erreichung des angestrebten Versuchsziels;
– dass Ihre Methodenwahl aussagekräftige und reproduzierbare Ergebnisse verspricht.

d

Formulierungsvorschlag

Das skizzierte Versuchsvorhaben ist geeignet, um das angestrebte Versuchsziel zu erreichen, weil … (= Qualität des Versuchsansatzes betonen: Validität Tiermodell, geeignete Kontrollgruppen, spezifische Expertise und Erfahrung der Versuchsleitung etc.)
Es verspricht aussagekräftige und reproduzierbare Ergebnisse, weil … (= Nennen der Massnahmen zur Vermeidung von Bias: Randomisierung, Verblindung etc.)

Schritt 2.2 Erforderlichkeit Ihres Versuchs

Stellen Sie dar, warum Sie Ihren Versuch in der geplanten Form für erforderlich halten und er nicht ohne Tiere, mit weniger Tieren oder mit geringeren Belastungen durchführbar ist.

Formulierungsvorschlag

Die Kriterien der 3R (Replace, Reduce, Refine) sind erfüllt:
Replacement ist nicht möglich, weil die Prüfung von Alternativmöglichkeiten ergab, dass … (ggf. Angaben zu systematischer Literatursuche von Alternativmethoden)
Eine Reduktion der Tierzahl ist nicht sinnvoll, weil … (= Angaben zur biometrischen Planung)
Dem Refinement wurde Rechnung getragen durch … (= konkrete Angaben zu allen Refinements); eine weitere Verminderung der Belastungen ist nicht möglich, weil … (= konkrete Begründung für alle verbleibenden Belastungen)

Schritt 3

Auflistung der Güter

Tragen Sie die relevanten Punkte für beide Waagschalen der Güterabwägung zusammen:

Waagschale «schutzwürdige gesellschaftliche Interessen»: Listen Sie den erwarteten Erkenntnisgewinn und seine Bedeutung für das Forschungsgebiet auf und zeigen Sie, dass Ihr Versuchsziel zumindest eines der gesetzlich festgelegten schutzwürdigen Interessen verfolgt.

Formulierungsvorschlag

Der angestrebte Erkenntnisgewinn liegt im Erreichen von … Er ist für das Forschungsgebiet bedeutend, weil … Dies steht im Einklang mit dem/den gesetzlich schutzwürdigen gesellschaftlichen Interesse(n) …

Waagschale «Belastungen der Tiere»: Zur Auflistung der Belastungen der Tiere nehmen Sie Rückgriff auf §56.1 – §56.4 und geben an, ob kumulative Belastungen auftreten und wie diese ins Gewicht fallen. Nennen Sie hier auch die belastungsmindernden Massnahmen (§53.1 – §53.2).

Formulierungsvorschlag

Es ist damit zu rechnen, dass die im Versuch verwendeten Tiere folgende Belastungen erfahren … (= Auflistung aller pathozentrischen und nicht-pathozentrischen Belastungen)

Güter in der Waagschale «schutzwürdige Interessen der Gesellschaft»
Alle im konkreten Fall geltenden schutzwürdigen Interessen von Seiten der Gesellschaft (aus den drei Kategorien: Erhaltung oder Schutz des Lebens und der Gesundheit von Mensch und Tier, neue Kenntnisse über grundlegende Lebensvorgänge, Schutz der natürlichen Umwelt).

Güter in der Waagschale «Belastung der Tiere»
Alle im konkreten Fall auftretenden Belastungen, sowohl pathozentrischer Art (Schmerzen, Leiden, Schäden, Angst) wie nicht-pathozentrischer Art (übermässige Instrumentalisierung, Erniedrigung, tief greifender Eingriff in Erscheinungsbild oder Fähigkeiten).

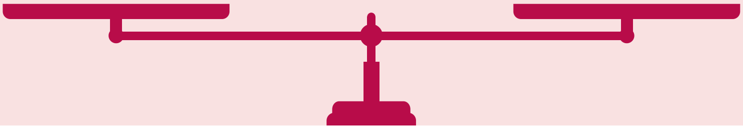

Schritt 4

Gewichtung der aufgelisteten Güter

In diesem Schritt erläutern Sie nachvollziehbar für jeden aufgelisteten Punkt in den Waagschalen, wie sehr er ins Gewicht fällt, zum Beispiel unter Verwendung der Kategorien gering/mittel/hoch/sehr hoch oder der Klassifizierung * bis ****, wie in der Anleitung Güterabwägung des BLV vorgeschlagen.

Formulierungsvorschlag

Die im hier beschriebenen Versuch für die Tiere auftretenden Belastungen sind folgendermassen einzustufen … (z. B. Schmerzen hoch)
Die Gewichtung der gesellschaftlichen Interessen beruht auf … (z. B. Erkenntnisse zum Schutz der natürlichen Umwelt im Bereich … von z. B. hoher Relevanz für …)

Schritt 5

Abwägung der Güter (Nachweis der finalen Unerlässlichkeit)

Legen Sie zum Schluss nachvollziehbar dar, warum Ihre Güterabwägung zugunsten der gesellschaftlichen Interessen ausfällt.

Formulierungsvorschlag

Die Gewichtung der relevanten Güter führt dazu, dass die Belastungen gegenüber dem angestrebten Erkenntnisgewinn …

Mit der korrekten Durchführung der Güterabwägung haben Sie Ihre gesetzlich geforderte Verantwortung wahrgenommen: Sie reichen ein Tierversuchsvorhaben ein, bei dem schutzwürdige Interessen der Gesellschaft die Belastungen der Tiere überwiegen, der Versuch also verhältnismässig ist und die Würde des Tiers geachtet wird.

6. Quellen und Links

Anleitung Güterabwägung / Erläuterungen Güterabwägung, herausgegeben vom Bundesamt für Lebensmittelsicherheit und Veterinärwesen (BLV): www.blv.admin.ch/blv/de/home/tiere/tierversuche/schweregrad-gueterabwaegung.html

ARRIVE Guidelines: www.nc3rs.org.uk/arrive-guidelines

Ethikkommission für Tierversuche der Akademien der Wissenschaften Schweiz (2010), Würde des Tieres und Güterabwägung im Schweizerischen Tierschutzgesetz.
Ein Positionspapier: www.akademien-schweiz.ch/index/Portrait/Kommissionen-AG/Kommission-fuer-Tierversuchsethik.html

Formular A: www.blv.admin.ch/blv/de/home/tiere/tierversuche/forschende.html

Gemeinsame Tierversuchskommission der Kantone Baselstadt, Baselland und Aargau, Merkblatt Güterabwägung für Tierversuche: www.ethz.ch/content/dam/ethz/main/research/ethik-tierschutz/documents/Merkblatt_Gueterabwaegung_de.pdf

Stiftung Forschung 3R: www.forschung3r.ch

Tierschutzgesetz: www.admin.ch/opc/de/classified-compilation/20022103/index.html

Tierschutzverordnung: www.admin.ch/opc/de/classified-compilation/20080796/index.html

Pesée des intérêts dans les demandes pour les expériences sur animaux

Guide destiné aux requérants

f

Indications concernant l'élaboration de ce guide

La Commission pour l'éthique dans les expérimentations animales (CEEA), une commission de l'Académie suisse des sciences médicales (ASSM) et de l'Académie suisse des sciences naturelles (SCNAT), est un organe d'expertise et de conseil dans le domaine des «expérimentations animales»; elle examine des actes législatifs et rédige des prises de position et des recommandations. Le présent guide a été élaboré pour faciliter aux requérants la réalisation de la pesée des intérêts. La décision a été précédée d'un processus d'évaluation minutieux. Le projet a été salué par les responsables de la protection des animaux des universités et de l'industrie ainsi que par les commissions cantonales pour les expériences sur les animaux.

Les personnes suivantes font partie de la Commission pour l'éthique dans les expérimentations animales (CEEA): Prof. Rolf Zeller, Bâle (Président); Dr Beatrice Roth, Zurich (Vice-Présidente); lic. théol., Dipl.-Biol. Sibylle Ackermann, Berne (ex officio); Dr Christoph Ammann, Zurich; PD Dr Alessandra Bergadano, Bâle; Dr Katharina Friedli, Ettenhausen; PD Dr Birgit Ledermann, Bâle; Prof. Christian Lüscher, Genève; Prof. Gregor Rainer, Fribourg; Dr Hans Sigg, Zurich; Prof. Susanne E. Ulbrich, Zurich; Prof. Brigitte von Rechenberg, Zurich; Prof. Hanno Würbel, Berne.

La CEEA s'est rencontrée à plusieurs reprises pour réfléchir à cette thématique. Après des discussions approfondies et l'audition d'experts externes, le groupe d'auteurs (S. Ackermann, Ch. Ammann, A. Bergadano, B. Roth, H. Würbel) a rédigé le texte et l'a soumis au débat dans la CEEA. Des procédures de consultation ont eu lieu en Suisse alémanique au printemps 2016 et en Suisse romande en automne 2016. La version finale tient compte des prises de position parvenues au secrétariat général.

1. Autorisation de procéder à des expérimentations animales en Suisse 34

2. La pesée des intérêts est l'élément clé de l'autorisation 34

3. Relation entre «dignité» et pesée des intérêts 35

4. Pesée des intérêts dans les demandes pour des expériences sur animaux – une procédure en plusieurs étapes 36

4.1 Vérification de l'indispensabilité instrumentale 36

4.2 Vérification de l'indispensabilité finale 37

4.2.1 Intérêts publics dignes de protection 38

4.2.2 Contraintes pour les animaux 38

5. Réalisation d'une pesée des intérêts pas à pas 41

Etape 1: Présentation de l'objectif de l'expérimentation et gain de connaissances 41

Etape 2: Présentation de l'adéquation et de la nécessité de votre essai 41

Etape 3: Liste des intérêts 42

Etape 4: Pondération des intérêts énumérés 43

Etape 5: Pesée des intérêts 44

6. Sources et liens 44

f

Conformément à la loi fédérale sur la protection des animaux (LPA), les chercheuses et les chercheurs qui déposent une requête pour une expérience sur les animaux doivent prendre en compte, en plus des éléments scientifiques, également les aspects «éthiques» et les présenter de manière compréhensible. Pour ce faire, il est nécessaire de procéder à une «pesée des intérêts». Le principe de la «pesée des intérêts» dans les requêtes pour des expériences sur les animaux est explicité dans ce guide avec l'objectif de vous soutenir dans sa réalisation.

1. Autorisation de procéder à des expérimentations animales en Suisse

En Suisse, les expérimentations animales sont soumises à des règles très strictes. Toute utilisation d'animaux sensibles à des fins d'expérimentations doit être justifiée. En tant que chercheuse/chercheur, il est de votre devoir de fournir cette justification sous la forme d'une pesée des intérêts. Votre requête est évaluée par l'organe compétent. Il s'agit généralement de l'office véterinaire cantonal qui est soutenu par une commission cantonale pour les expérimentations animales. L'examen de la requête peut soulever des questions qui exigent une reformulation de la requête. Par ailleurs, votre requête peut être soumise à des conditions.

Les projets dont le degré de gravité est de zéro (DG0) ne sont, en principe, pas soumis à la Commission pour les expérimentations animales, mais sont directement examinés par l'autorité compétente. Néanmoins, toute activité de recherche impliquant des animaux requiert toujours une justification fondée et – tout comme la production, l'acquisition et la détention d'animaux de laboratoire – une autorisation délivrée par l'autorité compétente.

2. La pesée des intérêts est l'élément clé de l'autorisation

D'une façon générale, une pesée des intérêts est réalisée quand une situation soulève des problèmes juridiques ou éthiques, lorsque pour les deux parties des intérêts dignes de protection s'opposent et sont difficiles à concilier. Dans le cas des expérimentations animales, les intérêts publics dignes de protection (souvent appelés «bénéfice escompté en termes de connaissances», par exemple nouvelles connaissances concernant des processus vitaux essentiels)

s'opposent à la protection des animaux contre les contraintes (par exemple douleurs, anxiété, avilissement).

La pesée des intérêts évalue au cas par cas la partie à laquelle il convient d'accorder le plus de poids. Elle clarifie si, au regard du bénéfice de connaissances escompté, les contraintes imposées aux animaux impliqués dans l'expérimentation sont justifiées. La pesée des intérêts que vous soumettez est évaluée quant à sa plausibilité, sa rationalité et son intégralité par la Commission pour l'éthique dans les expérimentations animales et par l'organe chargé d'établir les autorisations.

3. Relation entre «dignité» et pesée des intérêts

En Suisse, la «dignité de la créature» est ancrée dans la Constitution fédérale depuis 1992 et la «dignité de l'animal» dans la LPA depuis 2008. Il importe à cet égard de noter que la dignité comprend le bien-être des animaux (par exemple l'absence de douleur), mais ne peut être réduite à cet aspect. Le respect de la dignité suppose que l'identité et la spécificité de l'animal soient prises en compte, que toute intervention sur un animal fasse l'objet d'une réflexion critique et soit justifiée dans les limites du cadre légal.

La LPA précise dans l'art. 3, let. a: «Il y a atteinte à la dignité de l'animal lorsque la contrainte qui lui est imposée ne peut être justifiée par des intérêts prépondérants.» La pesée des intérêts permet de déterminer si un projet de recherche respecte la dignité de l'animal concerné ou non. Lorsque la pesée des intérêts conclut que les intérêts publics dignes de protection sont prépondérants, on considère que la dignité des animaux est respectée malgré les contraintes qui leur sont imposées. Il y a atteinte à la dignité de l'animal lorsque les intérêts de la société ne prédominent pas sur les contraintes imposées à l'animal.

La notion de «dignité» dans la loi sur la protection des animaux

«Au sens de la présente loi, on entend par dignité: la valeur propre de l'animal, qui doit être respectée par les personnes qui s'en occupent; il y a atteinte à la dignité de l'animal, lorsque la contrainte qui lui est imposée ne peut être justifiée par des intérêts prépondérants; il y a contrainte notamment lorsque des douleurs, des maux ou des dommages sont causés à l'animal, lorsqu'il est mis dans un état d'anxiété ou avili, lorsqu'on lui fait subir des interventions modifiant profondément son phénotype ou ses capacités, ou encore lorsqu'il est instrumentalisé de manière excessive.» (art 3, let. a)

4. Pesée des intérêts dans les demandes pour des expériences sur animaux – une procédure en plusieurs étapes

La LPA précise dans l'art. 17: «Les expériences qui peuvent causer aux animaux des douleurs, des maux ou des dommages, les mettre dans un état d'anxiété, perturber notablement leur état général ou porter atteinte à leur dignité d'une autre manière doivent être limitées à l'indispensable.» L'objectif de la procédure d'autorisation est de limiter les expériences sur les animaux «à l'indispensable».

Indispensabilité instrumentale
Le projet de recherche est-il adapté et indispensable, pour parvenir à l'objectif visé?

Indispensabilité finale
L'objectif de l'essai est-il suffisamment important pour justifier les contraintes imposées aux animaux?

Votre devoir en tant que chercheur est d'exposer en quoi vous considérez votre expérience sur animaux comme indispensable. Ce faisant, on distingue deux types d'«indispensabilité»: votre essai doit être indispensable tant du point de vue instrumental que final. Le recours à la ou au responsable de la protection des animaux de votre institution est recommandé dans tous les cas.

4.1 Vérification de l'indispensabilité instrumentale

Pour obtenir une autorisation, vous devez être capable de démontrer que l'expérimentation prévue est l'«instrument» approprié pour atteindre l'objectif visé. Concrètement la vérification de l'indispensabilité instrumentale concerne deux aspects: l'adéquation et la nécessité de votre projet d'étude.

L'adéquation de votre projet dépend d'une part de la force de conviction des résultats escomptés (c'est-à-dire de leur solidité et des possibilités de les reproduire et de les généraliser) et, d'autre part, de la capacité du projet à répondre aux questions soulevées. Parmi les éléments décisifs on peut citer le choix d'un bon modèle animal, une bonne opérationnalité (c'est-à-dire le fait de pouvoir mesurer la question de recherche), un plan d'expérience approprié ainsi que les critères de la bonne pratique scientifique concernant entre autres la randomisation, les calculs de la taille des échantillons, les modèles statistiques et le plan d'évaluation. Les contenus des guidelines ARRIVE, par exemple, peuvent servir de fil conducteur.

Pour remplir le critère de la nécessité, les exigences du principe des 3 R (Replace, Reduce, Refine) doivent être satisfaites, c'est-à-dire que l'objectif de l'expérience ne peut pas être atteint avec des méthodes n'impliquant pas d'animaux, avec des espèces animales moins évoluées, avec un nombre réduit d'animaux et/ou avec des méthodes moins contraignantes.

Pour démontrer l'indispensabilité instrumentale, il peut être utile de rechercher une expertise supplémentaire, par exemple en anesthésie, analgésie ou planification biométrique, en fonction de la nature de l'expérience projetée.

4.2 Vérification de l'indispensabilité finale

La vérification de l'indispensabilité finale envisage la possibilité que votre projet d'expérience – même s'il est excellent selon les critères scientifiques – pourrait être disproportionné et donc irrecevable. Votre projet est considéré comme finalement indispensable lorsque le gain de connaissances escompté attendu (et donc finalement l'intérêt de la société) semble tel, qu'il l'emporte sur les contraintes imposées aux animaux et justifie donc l'expérimentation.

Ainsi, les «intérêts publics dignes de protection» et les «contraintes imposées aux animaux» sont mis en balance. Pour ce faire, vous désignez quels intérêts vous placez dans chacun des deux plateaux de la balance (cf. chapitres 4.2.1 et 4.2.2). Vous évaluez ensuite, aussi objectivement que possible et en toute impartialité, d'une part les éléments favorables et, d'autre part, les éléments défavorables à votre expérience (cf. chapitre 5).

Il va de soi qu'en tant qu'investigateur vous ne soumettez votre projet que si la pesée des intérêts joue en faveur des intérêts publics dignes de protection. La commission cantonale pour les expériences sur les animaux et l'autorité chargée de délivrer l'autorisation peuvent approuver votre estimation de l'indispensabilité finale ou arriver à une conclusion différente. Ce dernier cas peut survenir en présence de doutes quant à l'exactitude ou la pertinence des hypothèses de base ou de l'objectif de l'essai ou lorsque les contraintes infligées aux animaux sont considérées comme trop lourdes, soit par rapport à l'objectif visé, soit dans l'absolu (à la limite supérieure de la souffrance acceptable).

4.2.1 Intérêts publics dignes de protection

Votre essai n'est recevable que lorsqu'il sert des intérêts publics dignes de protection. Selon l'ordonnance sur la protection des animaux (OPA art. 137), les objectifs suivants peuvent être invoqués comme intérêts dignes de protection:

- la sauvegarde et la protection de la vie ou de la santé humaines ou animales;
- l'acquisition de nouvelles connaissances sur des phénomènes vitaux essentiels;
- la protection de l'environnement naturel.

Lors de la rédaction de votre requête, il importe de décrire de manière aussi réaliste que possible le gain de connaissances effectivement attendu de votre expérimentation en renonçant à toute perspective de nature spéculative. La question de savoir dans quelle mesure l'expérience prévue peut permettre d'avancer pour atteindre l'un des objectifs cités ci-dessus, est décisive.

Pour l'évaluation de l'importance de l'essai, il peut être utile de recourir aux critères des processus d'évaluation scientifique (peer review) conformément aux principales institutions de recherche (par exemple FNS, DFG), notamment sur

- le gain attendu en termes de nouvelles connaissances;
- l'importance scientifique pour le domaine de recherche et, éventuellement, d'autres disciplines;
- l'originalité de l'essai;
- une signification particulière pour d'autres raisons qu'elles soient politiques, scientifiques ou sociétales.

4.2.2 Contraintes pour les animaux

Selon l'art. 3 LPA, «il y a contrainte notamment lorsque des douleurs, des maux ou des dommages sont causés à l'animal, lorsqu'il est mis dans un état d'anxiété ou avili, lorsqu'on lui fait subir des interventions modifiant profondément son phénotype ou ses capacités, ou encore lorsqu'il est instrumentalisé de manière excessive». Les «contraintes» englobent des interventions vécues comme pénibles par l'animal (= contraintes pathocentriques), mais également d'autres interventions que l'animal ne perçoit pas forcément comme pénibles. Le plus souvent, les expérimentations animales présentent des contraintes des deux catégories.

Pour les contraintes pathocentriques, une répartition selon le degré de gravité (DG0 – DG3) est établie en fonction de différents critères (douleurs, maux, dommages, anxiété) et de leur sévérité. Ce faisant, l'addition de contraintes de la même catégorie ou de différentes catégories (par ex. réduction de l'alimentation, prélèvements sanguins répétés, infection de l'animal) n'implique pas forcément le classement dans une catégorie de degré de gravité plus élevé; le degré de gravité d'un essai correspond au niveau de la contrainte la plus lourde. Il importe malgré tout de tenir compte dans la pesée des intérêts des contraintes cumulées: par exemple, pour justifier un essai qui comporte deux contraintes de la catégorie DG2, il faut que celui-ci apporte un gain de connaissances plus élevé qu'un essai qui ne comporte qu'une seule contrainte de DG2.

Les contraintes pathocentriques concernent les interventions vécues comme pénibles et affectant le bien-être des animaux. Elles comprennent:
- les douleurs,
- les maux,
- les dommages,
- l'anxiété.

Les contraintes non pathocentriques ne sont pas forcément vécues comme contraintes par l'animal, mais remettent néanmoins en cause le respect de sa dignité. Elles comprennent:
- l'instrumentalisation excessive,
- l'avilissement,
- les interventions modifiant profondément son phénotype ou ses capacités.

Les exemples présentés dans le cadre ci-dessous (p. 40) illustrent la caractérisation et la description des contraintes non pathocentriques (instrumentalisation excessive, avilissement, interventions modifiant profondément le phénotype ou les capacités).

Notez que si le terme d'«instrumentalisation excessive» permet par lui-même d'anticiper le résultat de la pesée des intérêts, il existe néanmoins des cas, où la pesée des intérêts conclut qu'une instrumentalisation excessive (dans le sens de «sévère») est justifiée par des intérêts publics prépondérants. Par ailleurs, l'instrumentalisation excessive et l'avilissement peuvent constituer deux aspects du même phénomène qui se manifestent lorsque le statut de l'animal en tant que créature vivante autonome n'est pas respecté ou qu'il est traité sans égards et sans justification suffisante à d'autres fins. Il s'agit notamment de situations de perte totale de contrôle ou lorsque les animaux sont mécanisés à outrance.

f

Exemples de contraintes non pathocentriques

Interventions modifiant profondément le phénotype et les capacités

Des souris athymiques (nude mice) sont largement utilisées comme organismes modèles. Suite à une mutation génétique, elles sont dépourvues de thymus et de poils. Le système immunitaire déficient et l'absence de poils peuvent être compensés par des mesures appropriées (environnement stérile, température ambiante plus élevée, etc.) de telle façon que les animaux ne subissent aucune contrainte pathocentrique. Toutefois, l'élevage de souris ainsi mutées correspond à une intervention modifiant profondément le phénotype (absence de poils) et les capacités (réponse immunitaire) des souris. L'élément suivant est décisif pour l'évaluation: une expérimentation avec des souris athymiques, pouvant être réalisé avec des souris «normales» sans perte de connaissances, sera jugé irrecevable dans la pesée des intérêts malgré l'absence de contraintes pathocentriques, car les contraintes non pathocentriques ne peuvent être justifiées.

Instrumentalisation excessive et avilissement

Les expériences de parabiose sont utilisées, entre autres, dans la recherche sur le vieillissement et sur les cellules souches. Pour ce faire, deux animaux (par exemple des souris) sont assemblés l'un à l'autre par une suture le long du flanc et leurs systèmes circulatoires sont reliés. La parabiose constitue une contrainte pathocentrique pour les animaux, mais les contraintes non pathocentriques entrent également dans la pesée. Le fait de relier deux animaux par une suture met fin à leur statut de créature vivante autonome et entraîne dans une large mesure une perte de contrôle; ainsi les critères de l'avilissement et de l'instrumentalisation excessive sont remplis.

5. Réalisation d'une pesée des intérêts pas à pas

Vous soumettez votre requête pour une expérience sur animaux à l'aide de l'application web e-Tierversuche (pour obtenir un accès, veuillez remplir le «Formulaire A: Demande pour expérience sur animaux»). Conformément au §63, vous êtes prié(e) de procéder à une pesée des intérêts.

Etape 1

Présentation de l'objectif de l'expérimentation et gain de connaissances

Présentez sommairement l'objectif de votre expérimentation et le gain de connaissance escompté en vous basant sur vos formulations dans les paragraphes 44.1 et 44.2.

Proposition de formulation

Conformément à l'art. 137 OPAn, l'expérimentation animale décrite ici vise l'objectif … (= mention de l'objectif général de l'expérimentation)
Il a été planifié dans le but de combler la lacune suivante dans la recherche … (= description du gain de connaissances escompté)
Les gains de connaissances concrètes suivantes sont ainsi visés … (= mention de l'utilité attendue des connaissances escomptées)

Etape 2

Présentation de l'adéquation et de la nécessité de votre essai (démonstration de l'indispensabilité instrumentale)

La deuxième étape consiste à démontrer que votre projet de recherche est adéquat et nécessaire pour parvenir à l'objectif visé. Ce faisant, deux aspects doivent être considérés:

Etape 2.1 Adéquation de votre expérimentation

Présentez la pertinence scientifique de votre projet en vous basant sur les §51.1 – §51.3 et §54.3 – §55, en démontrant,

– que votre approche expérimentale est adaptée à l'objectif visé par votre essai;
– que le choix de la méthode promet des résultats parlants et reproductibles.

f

Proposition de formulation

Le projet de recherche, tel qu'il est esquissé, est adéquat pour atteindre les objectifs de l'expérience, car … (= souligner la qualité de l'approche choisie: validité du modèle animal, groupe de contrôle adéquat, etc.); il promet des résultats parlants et reproductibles, car … (= mention des mesures destinées à réduire les biais: randomisation, étude en aveugle, etc.)

Etape 2.2 Nécessité de votre expérimentation

Expliquez en quoi votre expérimentation est nécessaire dans la forme prévue et pourquoi elle ne peut être réalisée sans animal, avec moins d'animaux ou avec des contraintes moins lourdes.

Proposition de formulation

Les critères 3R (Replace, Reduce, Refine) sont remplis:
Le remplacement n'est pas possible: la vérification des possibles alternatives a montré que … (le cas échéant, indications concernant la recherche systématique de méthodes alternatives dans la littérature)
La réduction du nombre d'animaux n'est pas opportune, car … (= indications concernant la planification biométrique)
L'exigence du raffinement a été prise en compte par … (= indications concrètes concernant tous les raffinements); une diminution des contraintes n'est pas possible, car … (= justification concrète pour toutes les contraintes restantes)

Etape 3

Liste des intérêts

Récapitulez les principaux éléments des deux plateaux de la balance dans la pesée des intérêts:

Plateau de la balance «Intérêts publics dignes de protection»: Énumérez les gains de connaissances attendus et leur signification pour le domaine de recherche en question; montrez que l'objectif de votre expérimentation vise au moins un des intérêts dignes de protection fixés par la loi.

Proposition de formulation

Le gain de connaissances attendu réside dans …
Il est significatif pour ce domaine de recherche, parce que … Conformément à la loi, il est en accord avec le ou les intérêts publics dignes de protection suivants …

Plateau de la balance «Contraintes imposées aux animaux»: Pour établir la liste des contraintes imposées aux animaux, basez-vous sur les §56.1 – §56.4 et indiquez s'il y a des contraintes cumulatives et quelle est leur importance. Indiquez également ici les mesures susceptibles de diminuer les contraintes (§53.1 – §53.2).

Proposition de formulation

On peut prévoir que les animaux impliqués dans l'expérience seront soumis aux contraintes suivantes … (= liste de toutes les contraintes pathocentriques et non pathocentriques)

Intérêts dans le plateau «Intérêts publics»
Tous les intérêts dignes de protection pour la société dans le cas concret (des trois catégories: maintien ou protection de la vie et de la santé de l'être humain et de l'animal, nouvelles connaissances concernant des processus vitaux fondamentaux, protection de l'environnement naturel).

Intérêts dans le plateau «Contraintes imposées aux animaux»
Toutes les contraintes survenant dans le cas concret, qu'elles soient pathocentriques (douleurs, maux, dommages, anxiété) ou non pathocentriques (instrumentalisation excessive, avilissement, interventions modifiant profondément le phénotype ou les capacités de l'animal).

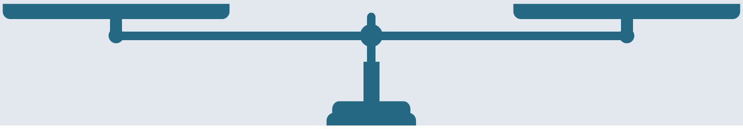

Etape 4

Pondération des intérêts énumérés

Dans cette étape, expliquez clairement pour chaque élément des plateaux de la balance, quelle est son importance, en s'appuyant par exemple sur les catégories moindre/moyenne/élevée/très élevée ou sur la classification * – **** telle qu'elle est proposée dans la directive sur la pesée des intérêts de l'OSAV.

Proposition de formulation

Les contraintes imposées aux animaux, telles qu'elles sont décrites dans la présente expérience, peuvent être classées comme suit … (par ex. douleurs extrêmes)
La pesée des intérêts de la société est basée sur … (par ex. connaissances concernant la protection de l'environnement naturel dans le domaine … sont d'importance pour …)

Etape 5

Pesée des intérêts (démonstration de l'indispensabilité finale)

Pour conclure, vous devez démontrer pourquoi votre pesée des intérêts est en faveur des intérêts publics.

Proposition de formulation

La pesée des principaux intérêts montre que les contraintes par rapport au gain de connaissances attendu …

En réalisant une pesée des intérêts correcte, vous assumez votre responsabilité, tel que l'exige la loi: vous soumettez un projet d'expériences sur des animaux, dans lequel les intérêts publics dignes de protection priment sur les contraintes imposées aux animaux, c'est-à-dire que l'essai est proportionnel et la dignité de l'animal est respectée.

6. Sources et liens

Commission d'éthique des cantons de Bâle-Ville, Bâle-Campagne et Argovie, notice pesée des intérêts pour les expérimentations animales: www.ethz.ch/content/dam/ethz/main/research/ethik-tierschutz/documents/Merkblatt_Gueterab-waegung_de.pdf

Commission d'éthique pour l'expérimentation animale des Académies suisses des sciences (2010), La dignité de l'animal et la pesée des intérêts dans la loi fédérale sur la protection des animaux. Une feuille de route: www.akademien-schweiz.ch/fr/index/Portrait/Kommissionen-AG/Kommission-fuer-Tierversuchsethik.html

Dignité de l'animal / Pesée des intérêts: explications, Édité par l'Office fédéral de la sécurité alimentaire et des affaires vétérinaires (OSAV): www.blv.admin.ch/blv/fr/home/tiere/tierversuche/schweregrad-gueterabwaegung.html

Directives ARRIVE: www.nc3rs.org.uk/arrive-guidelines

Fondation Recherches 3R: www.forschung3r.ch

Formulaire A: www.blv.admin.ch/blv/fr/home/tiere/tierversuche/forschende.html

Loi sur la protection des animaux: www.admin.ch/opc/fr/classified-compilation/20022103/index.html

Ordonnance sur la protection des animaux: www.admin.ch/opc/fr/classified-compilation/20080796/index.html

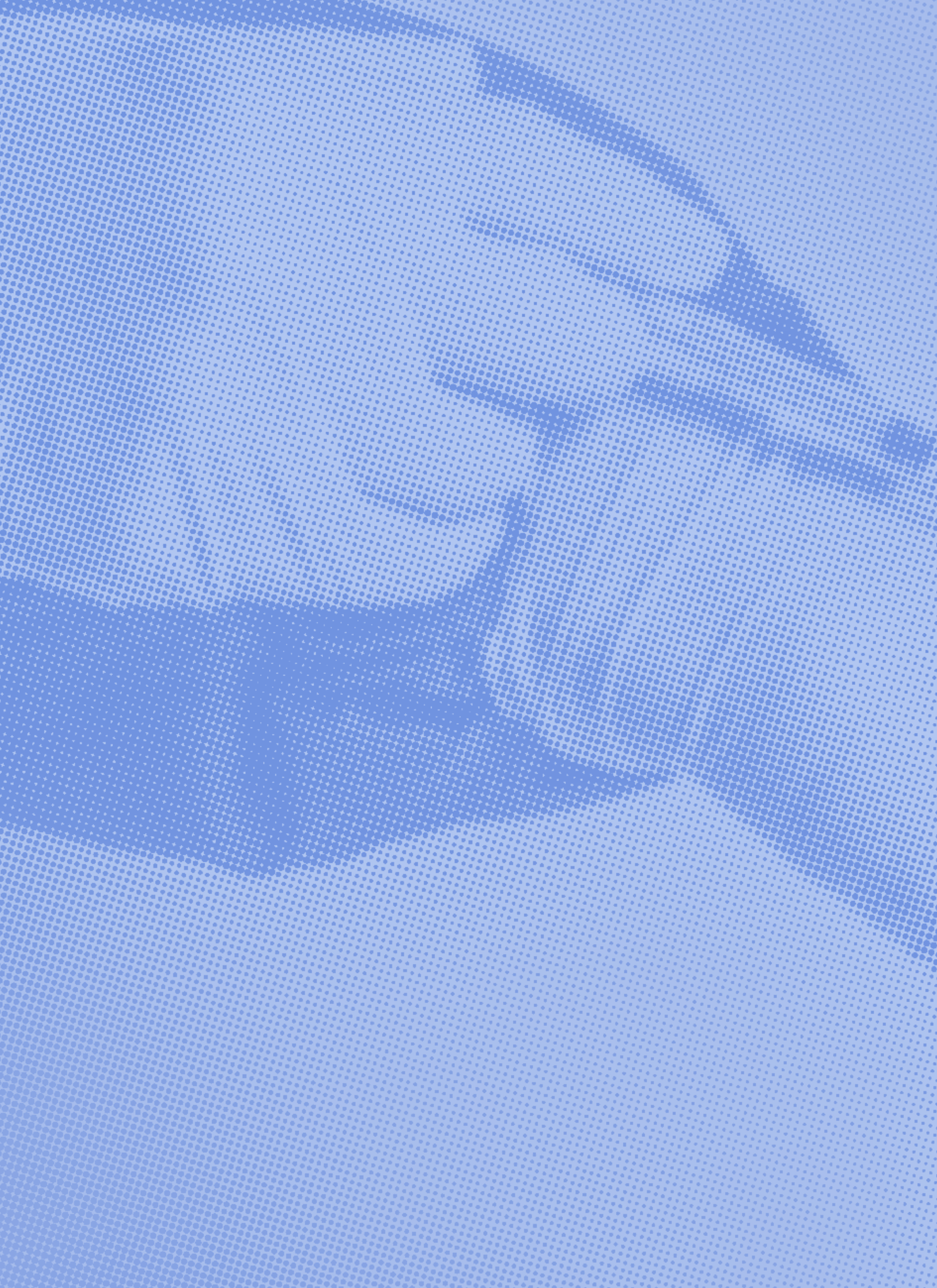

Supplement: S6 File — (PDF) [file pone.0297375.s006.pdf]
