# Supplementary material for: Mission impossible accomplished? A European cross-national comparative study on the integration of the harm-benefit analysis into law and policy documents
Source: PLoS One. 2024 Feb 20;19(2):e0297375. doi: 10.1371/journal.pone.0297375 (PMC10878508; doi:10.1371/journal.pone.0297375)
Supplement: S7 File — (PDF) [file pone.0297375.s007.pdf]

Home Office

The Harm–Benefit Analysis Process

New Project Licence Applications

Advice Note: 05/2015

Animals in Science Regulation Unit

December 2015

Contents

Table of Contents

Executive Summary 3

Glossary of terms 4

The Harm–Benefit Analysis Process New Project Licence Applications 7

- **Introduction** 7
- **Aim** 8
- **Scope** 8
- **The 3Rs** 8
- **Legislative Framework** 9
- **Existing Guidelines** 10
- **Overview of the Application Process** 10
- **Detailed Consideration of the Application Process** 11
- **The Harm–Benefit Analysis** 14
- **Robustness and Consistency of Decision-Making** 21
- **Other ASPA-Related Harm–Benefit Analyses Conducted by the Home Office** 21

Annex A: Current ASRU-I PPL assessment form 23

Annex B: References 34

Annex C: ASRU-I's Internal Referral Policy Document 35

Keywords:

Harm–Benefit Analysis, Animals (Scientific Procedures) Act 1986, Project Licence, Severity, 3Rs, Refinement, Replacement, Reduction

Disclaimer: 'The views expressed in this report are those of the authors, not necessarily those of the Home Office (nor do they represent Government policy).'

Executive Summary

This paper describes the current process used by the Home Office to conduct a harm–benefit analysis during evaluation of a project licence application. The harm–benefit analysis is required under the Animals (Scientific Procedures) Act 1986. It is the process of assessing the likely harms that the animals will experience and the likely benefits to be delivered and then determining whether the likely harms to animals are justified by the benefits likely to accrue. Harm–benefit analysis is undertaken, on behalf of the Secretary of State, by the Animals in Science Regulation Unit Inspectorate, who are veterinary or medically qualified advisers with particular expertise in assessing these research proposals. Other sources of advice in this regard include the Animals in Science Committee and/or independent assessors. The outcome of the harm–benefit analysis forms the basis of the recommendation to the Secretary of State to either grant a project licence or refuse an application.

Glossary of terms

3Rs The principles of Replacement, Reduction and Refinement

AALAS American Association for Laboratory Animal Science

ACHM Animals containing human material – as categorised in the AMS report on that subject

Actual severity The actual intensity of pain, suffering, distress or lasting harm experienced by an animal in a procedure or series of procedures. It should be the highest level experienced at any point during the course of the procedure and should take into account any cumulative effects

Admixed embryos Embryos combining both human and animal material

AMS Academy of Medical Sciences

Animals Procedures Committee Predecessor to the Animals in Science Committee

ASC The Animals in Science Committee – the independent, non-departmental public body set up under ASPA sections 19 and 20

ASPA The (Scientific Procedures) Act 1986 as amended by the Animals (Scientific Procedures) Act 1986 Amendment Regulations 2012 incorporating changes brought in by the European Directive (2010/63/EU) on the protection of animals used for scientific purposes – also referred to as the Act

ASRU The Animals in Science Regulation Unit, the unit of the Home Office responsible for implementing ASPA and comprising inspectors, licensing officers and those responsible for policy

ASRU-I The Animals in Science Regulation Unit Inspectorate. ASRU-I is part of ASRU and comprises inspectors who are veterinary or medically qualified advisers

AWERB Animal Welfare and Ethical Review Body

Benefits See “Likely benefits” below

Conspecifics Animals of the same species

Cumulative effect The effect which occurs where, in a series of procedures, a second or subsequent procedure has a compound effect, which may be positive or negative, in terms of causing pain, suffering, distress or lasting harm

Directive This refers to the European Directive on the protection of animals used for scientific purposes (2010/63/EU)

Establishment A place holding a licence which has been granted under section 2C of ASPA

Establishment licence A licence granted under section 2C of the Act, also known as a ‘section 2C licence’

EU European Union

EU Directive European Directive on the protection of animals used for scientific purposes (2010/63/EU)

EWG Expert Working Group

FAWC Farm Animal Welfare Council

FELASA Federation of European Laboratory Animal Science Associations

GA animals Genetically Altered animals (GAAs); this includes all genetically modified animals (transgenic, knock-out and other forms of genetic alteration) and mutations, whether naturally occurring or induced

Habituation A decrease in response to a stimulus after repeated presentations of that stimulus

Harm–benefit analysis An analysis in which the likely adverse effects in a procedure within a project are weighed against the potential benefits of the project for people, animals or the environment

Harms See “Likely harms” below

HO Home Office

Humane end-point Clear, predictable and irreversible criteria that allow early termination of a procedure before an animal experiences harm that is not authorised or scientifically justified

Inspector An inspector in ASRU appointed under ASPA section18

in vivo In the living organism

Likely benefits The benefits for people, animals or the environment which are considered achievable if the project objectives are successfully met

Likely harms The pain, suffering, distress or lasting harm likely to be experienced by animals during the course of the procedures within a project after applying all appropriate refinement techniques

NACWO Named Animal Care and Welfare Officer

NC3Rs National Centre for the Replacement, Refinement and Reduction of Animals in Research

NHP Non-human primate

NIO Named Information Officer

NVS Named Veterinary Surgeon

PSDLH Pain, suffering, distress or lasting harm. This includes anything that affects the animal’s physical, mental or psychological discomfort, whether occurring immediately (such as at the time of an injection) or in the longer term (such as the consequences of applying a carcinogen)

PEL An establishment (or section 2C) licence under ASPA

PIL A personal licence under ASPA

potentiation The increase in strength of nerve impulses along pathways which have been used previously

PPL A project licence under ASPA

Protected animals All living vertebrates, other than a human, including certain immature forms, and any living cephalopod

Protocol A procedure or series of procedures carried out for a particular purpose as part of an authorised project

Regulated procedure Any procedure applied to a protected animal for a qualifying purpose which may have the effect of causing the animal a level of pain, suffering, distress or lasting harm equivalent to, or higher than, that caused by the introduction of a needle in accordance with good veterinary practice

Retrospective assessment The formal assessment required in the Directive (**Article 39**) of specific types of projects, either during or at the end, to determine, amongst other things, whether the objectives have been achieved and whether lessons can be learnt to further the implementation of the 3Rs

sensitisation An amplified response to a stimulus resulting from repeated exposure to it

Severity The intensity of the pain, suffering, distress or lasting harm experienced by an animal during a procedure

Severity classification The process of assigning a severity category to a protocol. It may be mild, moderate, severe or non-recovery. It is based upon the greatest degree of pain, suffering, distress or lasting harm likely to be experienced by any animal within that protocol after applying all appropriate refinement techniques

SoS Secretary of State

The Harm–Benefit Analysis Process

New Project Licence Applications

Introduction

1. The Animals (Scientific Procedures) Act 1986 (ASPA) regulates procedures that are carried out on “protected animals” (any living vertebrate other than man and any living cephalopod) for scientific or educational purposes that may cause pain, suffering, distress or lasting harm (PSDLH).

2. ASPA has a three-part licensing system. Those carrying out regulated procedures must hold a “personal licence” (PIL). The regulated procedures to be carried out must be authorised by a “project licence” (PPL), which specifies the programme of work within which the procedures are being performed. The place at which regulated procedures are conducted must normally be specified in an “establishment licence” (PEL).

3. The PPL is granted by the Secretary of State (SoS) and specifies a programme of work that authorises the application, as part of that programme, of specified regulated procedures to animals of specified descriptions at a specified place or specified places. The programme of work, lasting up to five years, must have specific scientific aims and objectives. The PPL will include one or more protocols listing procedures to be applied to the subject animals, the expected adverse effects and the humane end-points and the severity mitigation strategies that must be applied to minimise PSDLH.

4. In carrying out the evaluation of a PPL application, to determine whether or not a PPL should therefore be granted, a harm–benefit analysis (HBA) must be undertaken. This is the process of assessing the likely harms that the animals will experience and the likely benefits to be delivered, and then determining whether the likely harms to animals are justified by the benefits likely to accrue.

5. HBA is undertaken, on behalf of the SoS, by the Animals in Science Regulation Unit Inspectorate (ASRU-I), who are veterinary or medically qualified advisers with particular expertise in assessing these research proposals.

6. The Animals in Science Regulation Unit (ASRU) is committed to describing how it currently carries out the HBA process. This is timely because:

    a. ASPA was amended on 1 January 2013 and new Guidance to ASPA was published in March 2014. This is a good opportunity to reflect on current process and determine whether enhancements could be made

    b. Currently, there are few published texts on how HBA is undertaken. A clear explanation of the HBA process, in the public domain, is in line with the Government’s policy on openness and transparency and will assist public understanding of how animal research is regulated in the United Kingdom (UK)

c. Other European Union (EU) States are developing methods for HBA following implementation of the European Directive on the protection of animals used for scientific procedures (2010/63/EU). A working group has been set up for the Federation of European Laboratory Animal Science Associations (FELASA) and American Association for Laboratory Animal Science (AALAS) to provide guidelines for undertaking HBA.

Aim

7. The aim of this paper is to describe the current (2015) process used by the Home Office (HO) to conduct an HBA during evaluation of a PPL application.

Scope

8. This paper is intended to be an outward-facing document that informs duty holders about the HBA conducted by the HO, under ASPA. The paper also provides openness and transparency on the ASRU decision-making process for wider stakeholders. In accordance with the paper's aim, it is limited to describing the current process for new PPL applications. However, HBA is an ongoing process. Reducing harms and optimising benefits should be considered throughout the conduct of animal research. This review will therefore also identify other instances where HBA is used during the regulation of animal research. The Animals in Science Committee (ASC) is undertaking further consideration of HBA and this paper will provide them with a starting place for their work.

9. In publishing a clear description of how we undertake the HBA process, we aim to explain to the public, and other stakeholders, how this process enables ASRU to make reasoned, balanced decisions, with regard to advising whether or not a PPL should be granted. This increased transparency will also illustrate how Inspectors take a consistent approach to evaluating harms and benefits in their advice on PPL authorities to the SoS. This document builds on the points covered in Appendix I of the Guidance on the Operation of ASPA and will place these in a practical context.

The 3Rs

10. Russell and Burch discuss the concept of Replacement, Reduction and Refinement (the 3Rs), as a key tool to minimise potential harms, in their seminal work, "The Principles of Humane Experimental Technique" (Universities Federation for Animal Welfare; 1959). The principle of the 3Rs is firmly embedded in the conduct of *in vivo* research in the UK and is explicitly detailed in EU and UK legislation. The currently accepted definitions are as follows:

    a. <u>Replacement</u>. The use of a scientifically satisfactory method or testing strategy not entailing the use of protected animals

    b. <u>Reduction.</u> The minimisation of the number of protected animals used in a programme of work without compromising the objectives of the programme

    c. <u>Refinement</u>. The elimination or minimisation of any possible PSDLH in the breeding, accommodation and care of protected animals and the methods used in regulated procedures.

Legislative Framework

11. The requirement to undertake an HBA is set out in the EU Directive 2010/63/EU, which was transposed into the existing UK legislation by amendment of ASPA.

    a. Section 5(1) of ASPA defines a PPL as "a licence granted by the SoS which specifies a programme of work and authorises the application, as part of that programme, of specified regulated procedures to animals of specified descriptions at a specified place or specified places"

    b. Section 5B(2) of ASPA precludes the granting of a PPL unless the SoS has carried out a favourable evaluation of the programme of work. The evaluation is favourable only if it verifies that:
       i) carrying out the programme of work is justified from a scientific or educational point of view or is required by law
       ii) the purposes of the programme of work justify the use of protected animals
       iii) the programme of work is designed so as to enable the regulated procedures applied as part of it to be applied in the most humane and environmentally sensitive manner possible

    c. Section 5B(3)(d) of ASPA requires that an HBA of the programme of work (within a PPL application) is undertaken to assess whether the harm that would be caused to protected animals in terms of suffering, pain and distress is justified by the expected outcome, taking into account ethical considerations and the expected benefit to human beings, animals or the environment

    d. Section 5C(3) of ASPA defines the permissible purposes under the Act. The SoS cannot grant a PPL unless they have verified that the programme of work is to be carried out for at least one of these purposes, namely:
       i) basic research
       ii) translational or applied research with one of the following aims:
          a) the avoidance, prevention, diagnosis or treatment of disease, ill-health or other abnormality, or their effects, in man, animals or plants;
          b) the assessment, detection, regulation or modification of physiological conditions in man, animals or plants; or
          c) the improvement of the welfare of animals or of the production conditions for animals reared for agricultural purposes.
       iii) the development, manufacture, or testing of the quality, effectiveness and safety of drugs, foodstuffs and feedstuffs or any other substances or products, with one of the aims mentioned in paragraph ii)
       iv) the protection of the natural environment in the interests of the health or welfare of man or animals
       v) research aimed at preserving the species of animal subjected to regulated procedures as part of the programme of work
       vi) higher education or training for the acquisition, maintenance or improvement of vocational skills
       vii) forensic inquiries.

Existing Guidelines

12. Guidelines regarding the HBA have already been published, including:

a. Working document on Project Evaluation and Retrospective Assessment, EU Commission's Expert Working Group for Project Evaluation and Retrospective Assessment (2013). The European Commission established an Expert Working Group (EWG) for Project Evaluation and Retrospective assessment of projects to facilitate the implementation of Directive 2010/63/EU. The UK HO contributed to the EWG

b. Guidance on the harm–benefit analysis of project licence applications (Appendix I of Guidance on the Operation of ASPA) (2014). This provides a summary of the factors taken into account during HBA

c. Animals Procedures Committee: Review of Cost–Benefit Assessment in the Use of Animals in Research (2003). This was a major report pre-dating the amendment to ASPA on 1 January 2013, which addressed the adequacy of the current cost–benefit assessment (as HBA was then termed).

Overview of the Application Process

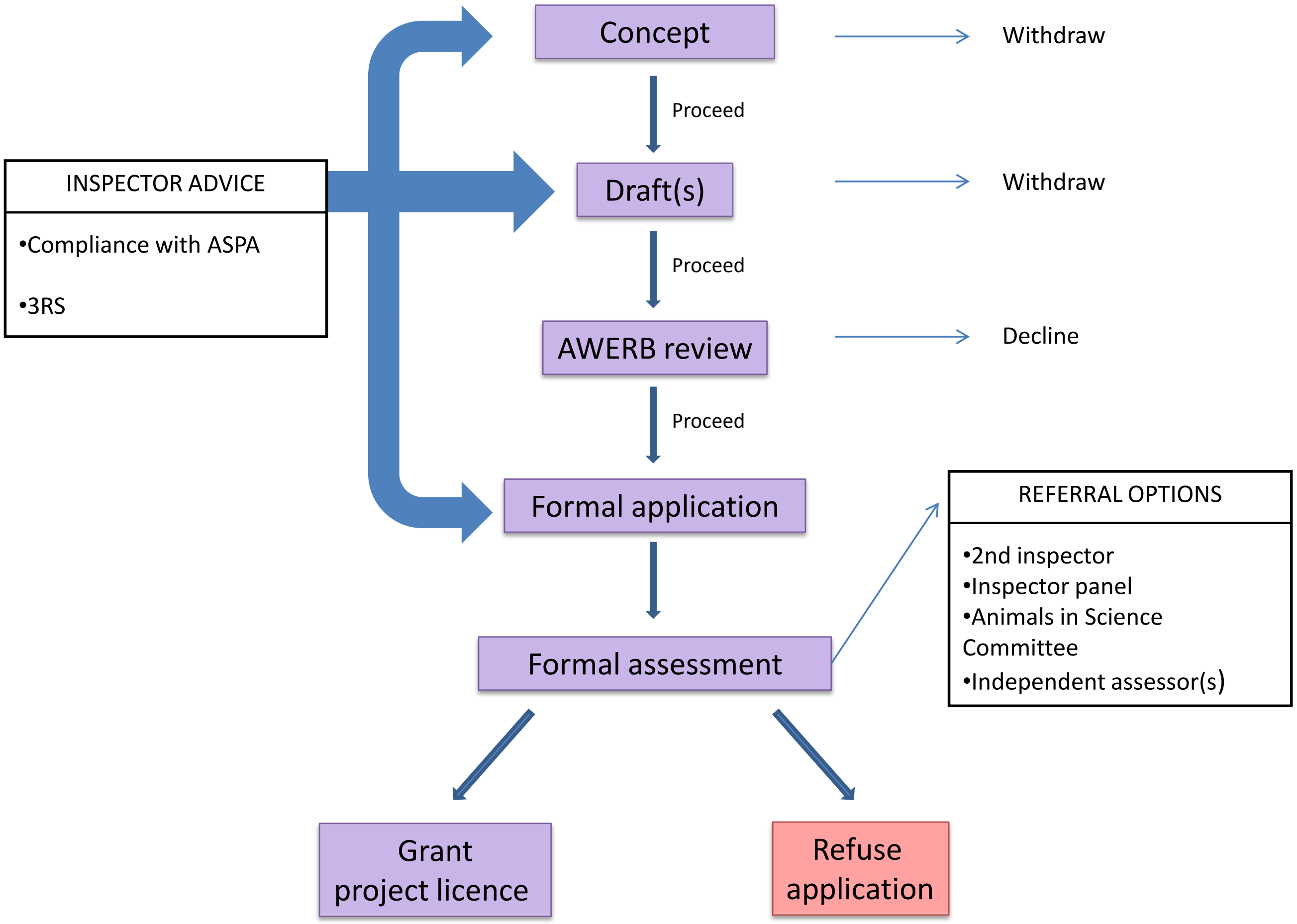

Sources of advice to the applicant

- Members of the Animal Welfare and Ethical Review Body (AWERB) at their establishment, especially the Named Animal Care and Welfare Officer (NACWO) and the Named Veterinary Surgeon (NVS)
- The assigned Inspector (if a draft was submitted to the Inspectorate)
- With the assistance of the Named Information Officer (NIO) at their establishment, specialist websites and databases about the 3Rs, such as the National Centre for the 3Rs website
- Scientist colleagues who have specific experience with the relevant animal models and/or scientific discipline (e.g. experimental design)

Detailed Consideration of the Application Process

13. The PPL application process can be split into a number of discrete phases:

   a. Concept. The applicant formulates a programme of work that fulfils one or more of the permissible purposes. They have the opportunity to seek informal advice on an early concept for a project from their assigned Inspector prior to submitting a draft or formal application. This might be before funding is secured or the programme of work is clearly defined. This allows concepts to be discarded completely if they:
      i) are of poor quality
      ii) are incompatible with the requirements of ASPA
      iii) have a balance of harm and benefit that would clearly fail an HBA.

   b. Draft. Applicants often submit a draft application before, or in parallel with, consideration of the project by their establishment's AWERB. Inspectors identify proposals that would obviously fail the HBA and advise that they are not proceeded with at this early stage. As with the concept, if the draft proposal has the potential to satisfy the requirements of ASPA and to pass the HBA, the Inspector gives feedback in order to minimise harms, to clarify the specific benefits to be delivered, to fully implement the 3Rs and to clarify the likelihood of delivery of the benefits. Often, additional information is requested at this stage to ensure that the Inspector has all the necessary relevant facts to undertake the HBA

   c. AWERB Review. Before an application can be formally submitted to ASRU it should have been reviewed by the establishment's AWERB, as one of the additional tasks listed in the HO Guidance. The AWERB advises the PEL holder whether to support a project application, primarily considering such proposals from a local perspective and bringing local knowledge and expertise to bear

   d. Formal Application. Following AWERB review and sign off by the PEL holder, the application is formally submitted to the HO. Some applicants will have addressed all of the Inspector's concerns before submission of the formal application so further information may not be needed at this stage. However, Inspectors may still need to negotiate and agree application of the 3Rs to ensure that the harms to be authorised in the PPL are both minimised and justified by the likely benefits

   e. Formal Assessment. The Inspector identifies omissions or required changes that must be addressed before the final HBA can be undertaken. The deficits relating to harms and

benefits usually fall into the following categories (based on Appendix I; Guidance on the Operation of ASPA):

- failing to adequately explain benefits:
  - often lacking the wider context of the research programme (and potential benefits of the specific project to the overall research programme)
  - benefits not sufficiently described or translational potential not explained– especially in the area of basic research; unsubstantiated/unrealistic claims of potential benefits
  - benefits not linked to the objectives set out in the application
  - not indicating the timescale when benefits may be expected (when feasible).
- failing to sufficiently address likelihood of success i.e. likelihood of attaining the objectives set for the project:
  - no information on the group's (or establishment's) track record (for example, previous experience; relevant publications; resource availability, including animal facilities and funding) to help assess likelihood of success
  - justification for the work is not well structured, lack of key indicators of success such as milestones to signal progression through the programme of work, insufficient focus and relevance
  - insufficient details to allow evaluation of the likelihood of achieving success
  - insufficient details on animal models (and where applicable, the use of Genetically Altered [GA] animals) and why they are the best and/or most practicable model for the purpose
  - insufficient information on how the regulated procedures contribute to the objectives of the project.
- failing to sufficiently address the application of the 3Rs:
  - omission of, or incomplete, information necessary to consider whether or not all 3Rs have been addressed – for example, missing information on how harms are reduced to a minimum consistent with scientific objectives and no justification given for circumstances where recognised good practices are not employed e.g. use of analgesia; social housing.
  - reassurance that alternatives to in vivo use could not meet the scientific objectives
- failing to adequately estimate harms:
  - procedures on animals not sufficiently detailed to estimate harms to individual animals
  - no information on nature and level of harms
  - insufficient information on welfare assessment or humane end-points
  - no or insufficient justification for re-use of animals.

Once the application does not contain any critical errors and has the essential information necessary for an HBA to be conducted, it is judged to be "complete and correct", the assessment and HBA is completed on the PPL assessment form (Annex A) and a recommendation for referral, granting or refusal is made.

f. Referral. Where applications raise issues requiring more detailed consideration (e.g. high severity work or other matters of particular public concern), additional advice may be sought either by the Inspector or by the SoS. Applications may be referred within the Inspectorate, to officials or Ministers, to independent assessors, or to the ASC.

i) *Internal referral within the Inspectorate.* An application may be referred to a second Inspector or panel of Inspectors if specific expertise is required on an aspect of the

application, or if the application falls into certain categories. This process is covered by an internal policy document (see Annex C). Such referrals can be mandatory or recommended. They either focus on a particular aspect of the application (e.g. a protocol that has been classified as severe) or relate to the whole application

ii) *Independent assessor.* Advice from an independent assessor may be sought by the SoS when the issues raised require specific, expert knowledge not available within ASRU-I, or when there is debate within the scientific or welfare communities, or between the Inspectorate and the applicant about:
   - the scientific validity of the proposed hypothesis(es) to be tested
   - the scientific validity of the proposed methodology
   - the scope for further application of the 3Rs
   - the likely benefits arising from the programme of work
   - the likely harms to animals
   - the choice of species (e.g. is the use of a specially protected species essential?).

   Independent assessors are typically active researchers with relevant expertise. They can be drawn from the international research community, or may be UK-based. When the SoS intends to consult an independent assessor the applicant is notified, in accordance with section 9(2) of ASPA, and the SoS will take account of the applicant's views regarding the choice of independent assessor. The applicant is usually told the identity of the independent assessor and the questions they have been asked to address. The applicant may also be given the opportunity to read the independent assessor's report. The report may also be made available to the ASC. The assessor's advice will be taken into account in the SoS's decision, but is not binding

iii) *ASC.* There are a number of criteria that require PPL applications to be referred to the ASC:
   - the use of wild-caught non-human primates (NHPs)
   - the use of cats, dogs, equidae or NHPs in severe protocols
   - use of endangered species
   - projects with major animal welfare or ethical implications
   - projects involving the use of admixed embryos falling into Category 3 of the Academy of Medical Sciences (AMS) report on Animals Containing Human Material (ACHM) and Category 2 where the predominance of the admixed embryo is unclear or uncertain
   - projects which may invoke any of the 'safeguard clauses' in the Directive with respect to the purpose of NHP use, proposals for the use of a great ape or proposals to cause long-lasting pain, suffering or distress that cannot be ameliorated
   - projects of any kind raising novel or contentious issues, or giving rise to societal concerns.

   The applicant is always invited to attend the ASC meeting at which the project is considered, and is given the opportunity to make a presentation about the application. They are also questioned by the Committee. The assigned Inspector also attends in order to provide any technical advice that the Committee might require. Other Inspectors may also attend. The Committee may recommend to the SoS that a PPL be granted (with or without further changes or additional conditions) or that the application be refused. Where appropriate, the assigned Inspector discusses the points raised by the Committee with the applicant and, if necessary, a final version is produced.

g. <u>Recommendation</u>. Having completed the assessment the Inspector then recommends that the application is either granted or rejected. As a consequence of the iterative process described above, it is unusual for an application to be rejected outright at the

formal application stage. It is more common for it to be returned to the applicant for either minor or major changes before recommendation. Most unsuitable applications are identified at the concept or draft stage and are withdrawn completely or revised by the applicant and re-submitted. The Inspector's recommendation includes the duration of the project, whether any additional conditions should be applied to the licence and the details of any requirement for retrospective assessment of the project

h. Grant or Refusal. The ASRU Licensing Team then acts on behalf of the SoS and considers the advice provided in the Inspector's recommendation in order to grant or refuse the licence. Where appropriate recommendations may be triaged up to senior officials or, occasionally, to the Minister for a final decision

i. Timescales. Unless the application involves a complex or multi-disciplinary programme, the assessment will be completed within 40 working days of receiving a complete and correct application. The PPL will either be granted or the applicant is notified of the intention to refuse the application. For applications describing a complex or multi-disciplinary programme, the period may be extended by up to 15 working days and the applicant will be notified accordingly

j. Disagreements. There may be occasions when the applicant and assigned Inspector are unable, despite discussion, to agree on one or more aspects of an application. In such cases there is an informal resolution process that is likely to involve other members of the Inspectorate, with particular knowledge or experience of the issue, and/or the Principal Inspector with responsibility for assessment of project licence applications. This process can be either requested by the applicant or sought by the assigned Inspector.

   However, should this not prove to be successful, there is the formal option to make representations under section 12 of ASPA, which is described at Appendix E of the Guidance on the Operation of ASPA (2014).

The Harm–Benefit Analysis

14. ***Harms.*** Each protocol in the project has a severity classification applied, which is proposed by the applicant but confirmed and agreed by the Inspectorate. These classifications are defined in ASPA, (non-recovery, mild, moderate or severe). However, this broad classification, which reflects the upper severity limit (or worst-case scenario for any individual animal) within each protocol, does not provide sufficient information for the harms to be fully assessed – for example, information regarding the nature, incidence or duration of the harms or the percentage of animals affected also has to be taken into account. In addition, the detailed information provided in each protocol is used to determine the different levels of severity likely to be experienced within that protocol. The following aspects need to be considered:

   a. Contingent Harms. These are the inherent and inescapable harms arising from the experimental or scientific use of an animal. Examples of contingent harms include being housed in a cage (as opposed to being able to range freely in the wild), inability to express a wide range of the natural behaviours, handling or transport stress, and olfactory exposure to a large number of conspecifics (in the case of laboratory rodents housed in conventional caging).

      The Brambell Report (1965) recommended that animals should have the freedom to stand up, lie down, turn around, groom themselves and stretch their limbs. The Farm Animal Welfare Council (FAWC) developed these into the "Five Freedoms", which are a

framework for the analysis of animal welfare. These principles are still used to help improve the welfare of animals kept by humans for many different purposes. They have been highly influential in animal welfare legislation and farm animal welfare accreditation schemes, and are a useful framework when considering contingent harms for laboratory animals:

i) *Freedom from hunger or thirst* by ready access to fresh water and a diet to maintain full health and vigour
ii) *Freedom from discomfort* by providing an appropriate environment including shelter and a comfortable resting area
iii) *Freedom from pain, injury or disease* by prevention or rapid diagnosis and treatment
iv) *Freedom to express (most) normal behaviour* by providing sufficient space, proper facilities and company of the animal's own kind
v) *Freedom from fear and distress* by ensuring conditions and treatment which avoid mental suffering.

b. Project-Related Harms. These are specific to the regulated procedures undertaken. These can range in complexity from single housing of social species to major surgical interventions. As with contingent harms, the “Five Freedoms” can also be of relevance to project-related harms. The key issues for project-related harms include:
- the types of procedures
- the frequency of procedures
- the duration of procedures
- the proportion of animals likely to reach each level of severity within a protocol
- the nature, severity and likelihood of each adverse effect and the proportion of animals predicted to be affected
- the origin, species, strain and age/stage of development of animals being used
- the number of animals
- the fate of each animal (e.g. humane killing, re-use, rehoming)
- are animals killed? If so, by what method and how many?
- are animals to be re-used?

c. Cumulative Effects. These are the net impacts of all the events (procedurally and husbandry-based) and effects that affect adversely, positively, and by way of amelioration, the welfare of an animal over its lifetime. They include likely habituation, potentiation and/or sensitisation and any temporal element in which recovery between events and memory of them and/or their consequences is likely to be affected

d. Mitigation/Amelioration of Harms. Applicants are required to justify their choice of species, models and methods, and are asked to explain why these are the most refined (i.e. minimise PSDLH). They are also asked how they will minimise suffering whilst achieving their objectives, and to provide additional justification for any severe severity models

Refinement strategies must be specifically and practically defined in the adverse effects section of each protocol. The following information must be provided:

i) *Mitigation strategies.* These are actions taken to reduce the frequency and severity of adverse effects, so that a procedure can be undertaken in the most refined way, whilst allowing the scientific objective to be achieved e.g. the peri-operative use of analgesia in animals undergoing surgery
ii) *Humane end-points.* These are clear, predictable and irreversible criteria that allow early termination of a procedure before an animal experiences harm that is not authorised or scientifically justified. In other words, they serve as absolute upper limits (or caps) to the nature and level of suffering that an animal will experience. An

example of a robust and useful humane end-point would be an upper limit to a blood value for a biochemical marker of renal disease. Humane end-points are not necessarily quantitative, but can be based on practical, professional judgement, for example:
"If post-operative complications occur, animals will be killed unless, in the opinion of the Named Veterinary Surgeon, such complications can be remedied promptly and successfully using no more than minor interventions such that the severity classification of the procedure is not increased."

Humane end-points should be used to intervene before death occurs. The use of death as an end-point must be avoided except in very exceptional circumstances. This is reinforced by PIL Standard Condition 7 and PPL Standard Condition 9. It would usually only be authorised where it is a requirement in regulatory testing required by law.

e. Additional Controls of Harm. In addition to the inclusion of mitigation strategies and humane end-points in protocols, as a means of minimising harms (see 14.d. above), further controls are provided by Standard Conditions that are applied to PILs and PPLs:
   i) *Unnecessary PSDLH.* PIL Standard Condition 5 states that "Where the licence holder is applying a regulated procedure to an animal the holder must ensure that any unnecessary PSDLH that is being caused to the animal is stopped". PPL Standard Condition 8 states that "The licence holder shall ensure that where a regulated procedure is being applied to an animal as part of the programme of work specified in this licence, any unnecessary PSDLH that is being caused to the animal shall be stopped". These two Standard Conditions set a general upper limit to harm under ASPA
   ii) *Long-lasting, severe pain, suffering or distress that cannot be ameliorated.* PIL Standard Condition 8 requires a licence holder to ensure the immediate killing of an animal that is being or has been subjected to a regulated procedure and "is in severe pain, suffering or distress which is likely to be long-lasting and cannot be ameliorated". PPL Standard Condition 7 prohibits the application of a regulated procedure to an animal as part of the programme of work specified in a PPL "if the procedure may cause the animal severe pain, suffering or distress that is likely to be long-lasting and cannot be ameliorated". These two Standard Conditions set an absolute upper limit to harm under ASPA.

f. Further Limits to Harms. There are other specific limits elsewhere in the legislation:
   i) The use of Great Apes (chimpanzees, pygmy chimpanzees, gorillas and orang-utans) and the use of stray animals of a domestic species are now prohibited under ASPA
   ii) Specific justification is required for the use of models categorised as severe and robust explanation must be provided as to why there is no other more refined method that would achieve the scientific objectives
   iii) Procedures can be applied to specially protected species (dogs, cats, NHPs and equidae) only in particular circumstances. The SoS will only grant a PPL for dogs, cats or equidae where the purpose of the programme of work specified in the licence can be achieved only by their use; or where it is not practicable to obtain other suitable animals. The use of NHPs will only be authorised for procedures carried out for specific permissible purposes and if scientific justification is provided showing that the purpose of the programme of work cannot be achieved by the use of animals that are not NHPs.

15. ***Benefits.*** The benefits considered during the HBA are the specific, expected beneficial outcomes of the objectives of the project, and not the non-specific benefits of the area of

research in general. For example, a specific benefit would be understanding how the blood supply to a tumour develops, with the aim of informing the development of a drug to stop tumour growth, not "cancer research". Sufficient information about benefits must be included in the application to inform the Inspector of the measurable outcomes likely to result from the project. However, there may be further indirect benefits that are not immediately obvious, i.e. they will occur downstream of the direct benefits. The field-related and the cross-field benefits that inform related and non-related areas of science can also be difficult to identify and capture. On the other hand, the indirect and long term benefits are sometimes overstated by the applicant, and the Inspector may need to seek more information to assess the likelihood that the different aspects of benefits will be delivered. Since the HBA is done before the work is started, there is always some uncertainty about benefit delivery. This makes the evaluation of benefit difficult. It is, by necessity, a value-laden judgement of the benefits and the likelihood of their delivery. Benefits can be subdivided as follows:

a. <u>Direct or Project-Related Benefits</u>. The benefits ascribed directly to a project should be realistic, achievable, and measurable, have a defined time-frame and be directly linked to the programme of work. They are informed by the background to the project, scientific aims and objectives, permissible purpose under ASPA and the programme of work. The stated likely benefits should be put into context with the current state of the research area, any knowledge gaps and their importance. They need to be specifically defined and mapped against clear scientific outputs of the project e.g. to define the role of gene x in the development of blood supply to a tumour.

b. <u>Indirect Benefits</u>. Indirect benefits are often omitted by the applicant, are difficult to predict, but can be important to consider particularly in the evaluation of fundamental/basic science projects as opposed to applied science projects. The benefits that arise from a project may have a wider impact than immediately obvious. These indirect benefits include:
   i) *Field-related benefits.* These are benefits that have a wider application within the scientific field of interest, beyond the narrower scope of the project e.g. a cellular signalling pathway that is being investigated for one aspect of organ development may also be biologically active elsewhere in the embryo
   ii) *Cross-field benefits.* These are benefits that have application to fields of research in addition to the one being investigated in any particular PPL. Leaps of scientific application and resulting benefits can be difficult to define or predict. They are not uncommon due to the overlapping and translational nature of biomedical research e.g. studies of the components of venom of the Gila monster, Heloderma suspectum, have resulted in the identification of a therapeutic agent for the treatment of type 2 diabetes and has now been clinically trialled in neurology
   iii) *3Rs benefits.* The benefits arising from application of the 3Rs, whilst not the principal output of the project, will add value to the HBA e.g. the development of a more refined disease model in one PPL could be of significant value to the wider scientific user community. Also, PPLs that authorise work providing a centralised service for animal model production e.g. breeding of GA mice as a service, will frequently lead to Reduction and Refinement because the staff conducting the work develop particular expertise in that area.

16. The assessment of the possible benefits of a PPL can be facilitated by answering the following questions:

a. <u>What will be the benefits of the work?</u> These are the direct and indirect benefits that are discussed above:
   - what data may be acquired?

- what drugs may be developed?
- what scientific questions will be answered?
- what knowledge gaps might be filled?
- what is the project's output?

b. Who and how many will benefit from the work?
- other researchers?
- human or veterinary patients?
- a relatively small set of patients, e.g. people with a rare genetic disease, or potentially millions, e.g. a vaccine candidate for malaria?
- the environment?

c. How will the benefits accrue?
- improved scientific knowledge/understanding?
- new or more efficacious therapies?
- cheaper therapies?
- impact on patients' quality of life?

d. When will the benefits be achieved? This can range from within the lifespan of the PPL (e.g. toxicological safety testing) to decades in the future (e.g. basic research into malarial immunology that may eventually contribute to the development of an efficacious vaccine).

e. What benefits are not allowed? There are prohibitions to certain categories of benefits
- developing or testing offensive weapons
- developing or testing alcohol or tobacco products
- testing cosmetics.

17. ***Weighting Harms and Benefits.*** Having considered the aspects of the harms and the benefits in detail, an overall judgement is made regarding the severity of the harms, and the value and likelihood of delivery of the benefits, i.e. both harms and benefits are *weighted*. In order to determine whether or not a project should be granted, the harms are then weighed against the benefits. The *weighing* of harms against benefits can be considered to be the process of determining if the overall harm that will occur is justified by the benefits that are likely to be delivered. Since there are no agreed quantitative units for either harm or benefit, this is not a quantitative analysis.

a. Weighting Harms. The Inspector makes an overall judgement of the final "weight" (i.e. extent, degree, severity) of the harms, taking into account the types of information listed in paragraph 14. This includes objective information e.g. species used, numbers of animals used in each protocol, lists of procedures to be applied, and subjective judgement of factors that are not known and must be predicted e.g. severity classification of each protocol as defined by the descriptors in ASPA and Guidance on the Operation of ASPA, likely overall life experience of the animal and unexpected adverse effects that may happen at unpredictable frequency. This is distilled on the assessment form and may include such information as the typical harms to be experienced, worst-case scenarios, the severity limits in place and the specified humane end-points. A numerical scoring system is not used although the terms "non-recovery", "mild", "moderate" and "severe", as defined in ASPA, may be used to express the differing levels of harm within each protocol and overall within the project.

b. Weighting Benefits.

i) *The value of benefits.* Weighting (determining the value) of particular benefits is

usually more difficult than weighting of harms. In scientific research, there is always uncertainty about which benefits will be delivered and when, so when a project is evaluated, the likelihood of delivery of benefit also has to be taken into account.

Although the perceived value of benefit can be subjective, Inspectors are expected to make consistent judgements on PPL applications. It has to be recognised that views differ between the various stakeholder groups that have an interest in the HBA process. For example, developing drugs for better treatment of breast cancer may be considered more valuable by some people than the study of addiction to recreational drugs, although both would provide benefit to many individuals within society. Factors such as the seriousness of a human disease, number of patients affected, and hence overall impact on society, are all taken into account when determining the importance of a benefit. In acting on behalf of the regulator, the role of the Inspector is to make a balanced, rational decision based on the information provided in the PPL application and other relevant sources.

Some groups argue that the use of live animals in education is less important than testing the safety of medicines under a regulatory regime, or that human health should come before animal health. It is therefore difficult to place benefits from animal research in a simple hierarchical order. The importance of work is subjective and changes with time and place, and depends on culture, environment and the emergence of new knowledge and societal attitudes.

Although this weighting of benefits is difficult, Inspectors make judgements about the importance of benefits in each project and how likely they are to be delivered. PPLs that are extremely likely to benefit human health directly are generally considered of high value, particularly if they are targeted at a common disease with high mortality and morbidity. Basic science projects that lead to better understanding of normal organs or processes are sometimes vital precursors to the study of disease processes and therapies. These would be considered to be of high value if they address a knowledge gap and they are likely to inform development of diagnostic or therapeutic options. They would be considered of lower value if the work is not contributing significantly to new knowledge.

Many types of product, including industrial chemicals, pesticides, medicines and foodstuffs, are subject to legal controls which include a requirement for safety assessment. Regulators require sufficient information to assess the risk of the product to humans, animals or the environment. In some cases, the information must demonstrate that the product is efficacious, particularly in the case of medicines. In many cases, regulatory authorities require a number of different types of data before a product can be registered, and some of these data can currently only be derived from tests on animals. These substances and products can be marketed only after appropriate safety and efficacy data have been submitted. The benefits arising from PPLs authorising such work relate to the safety of humans, animals or the environment. Practically, the benefits will arise from the subsequent use of a medicine which may be safer or more efficacious than current therapies or that may fulfil a hitherto unmet clinical need; the use of a more environmentally sensitive pesticide; or the economic benefits arising from the use of a new industrial chemical.

ii) *The likelihood that benefits will be realised.* The following factors are taken into account when determining the likelihood that benefits are going to be achieved for a particular programme of work:

a) The appropriateness of animal models and the extrapolation of results to the

medical or veterinary condition, if relevant. The Inspector uses their knowledge of animal models from a particular field, literature review, continuous professional development and observations from inspection of other similar work to judge the appropriateness of a model

b) Clear and persuasive arguments by the applicant. If there is uncertainty here, clarification is sought either within the application or by face-to-face discussions with the applicant
c) Trust and confidence in the culture of care at an establishment where work will be conducted. This information is gathered during inspection of establishments and may include observations of the applicant and their group undertaking procedures, meetings with named people and other staff working under ASPA and understanding of the local processes in place to prevent non-compliance
d) Are the objectives realistic?
e) Is the work timely? Information on the timeliness of a project should be presented in the application. If further information is required, the Inspector requests it from the applicant, and/or reviews the relevant literature
f) Is the work scientifically sound? This is judged from the information provided by the applicant to support their scientific hypotheses, knowledge of whether the programme of work has been peer-reviewed during the funding process, literature review to substantiate the claims made in the application, discussions with colleagues, scientists or other relevant experts. Further advice may be sought via referral to other Inspectors, independent assessors or the ASC
g) Is it deliverable in the timeframe outlined?
h) Is it adequately resourced? (financial, appropriate facilities, equipment and personnel: both scientific and care staff with appropriate training and expertise)
i) What is the experience/track record of the applicant or the research group in the field and in the specific area of planned work? Information on these aspects is requested in the application and are substantiated by the Inspectorate, if necessary, by literature review
j) Is there a clearly defined plan of work, including information about choice of methods/experimental design/species/animal model?
k) What is the publication plan (where appropriate)?

The Inspector summarises their overall judgement of the value and likelihood of delivery of the benefits. The significance of the likely benefits and the likelihood of their delivery are described on the assessment form.

Example

The applicant is a world-leading expert with strength and depth in their research team, and has ten years of experience with the proposed animal models. Her work has previously been inspected, and is considered to be done competently with due attention to the 3Rs, in a background of a good culture of care at the establishment. She is developing a new therapeutic target for a common terminal condition in children. She has a proven track record of taking potential therapeutics through to clinical trials. Benefit is therefore considered **important** and **likely** to be delivered.

18. The Decision-Making Process.

a. Weighing (Balancing) Harms and Benefits. Finally, a decision is made as to whether the harm that might be caused to protected animals in terms of PSDLH is justified by the expected benefits. This is a value-laden judgement that is based upon the factors described and discussed in this paper. The final recommendation is made to the SoS (or his/her officials). In most cases the advice comes solely from the Inspectorate. All relevant information provided by the applicant is taken into account, as well as information from other sources available to the Inspector, such as literature review, discussion with expert colleagues and knowledge from the Inspector's own expertise and experience. However, in more complex cases that have been the subject of referral, advice may also come from the ASC and/or independent assessor(s). A final decision to grant or refuse is then made by the SoS on the balance of the advice received from these various sources

b. Formal Recording of the HBA. The HBA is recorded using the current PPL Assessment Form (see Annex A). The Inspector reports their weighting of harms and benefits, and the weighing of the benefits against the harms. The Inspector sets out the basis for their decision to either recommend the granting of the project licence or the refusal of the application. It is this completed section that fulfils the SoS's obligations under section 5 of ASPA.

Robustness and Consistency of Decision-Making

19. In order to make consistent, well-judged decisions when undertaking HBA, Inspectors need a common understanding of how the aspects of harm and benefit are evaluated. Proper reasoning processes need to be undertaken in order to reach reasonable conclusions. There needs to be awareness of the concerns and views of the public and other key stakeholders. A number of mechanisms are used to ensure that proper reasoning processes are undertaken, that reasonable conclusions are drawn, and that significant inconsistency is avoided:
   - internal referral of specified cases to one or more Inspectors who have relevant expertise and/or experience
   - the use of Inspector review panels, where complex cases are discussed amongst several different Inspectors with different areas of expertise
   - regular case discussion groups amongst the Inspectorate
   - referral of cases to the ASC
   - the use of independent assessors
   - engagement with external stakeholders who share disparate views.

Other ASPA-Related Harm–Benefit Analyses Conducted by the Home Office

20. Although outside the scope of this paper, it should be noted that the Inspectorate conducts HBA in respect of PPLs in four other situations:

a. PPL Amendments. Applications to amend a PPL are subjected to an HBA following the same principles as that conducted for a new application. A further assessment is made as to whether or not the amendment is compatible with the objectives and the scope of the existing programme of work. Clearly an incompatible amendment cannot be recommended, regardless of how positive the HBA may be

b. Retrospective Assessments of PPLs. The requirement for a retrospective assessment and HBA for programmes that involve NHPs or where procedures are “severe” is set by section 5B(7) of ASPA. Furthermore, in accordance with UK policy, those projects using cats, dogs and equidae and authorising the use of endangered animals, together with all PPLs for education and training will normally be assessed retrospectively. Such assessments may be done either during the lifetime of the project at a certain time-point, e.g. annually, or at the end of a project. Section 5F(2) of ASPA specifies the points to be considered in a retrospective assessment:
   i) *Whether the programme of work has been carried out*
   ii) *Whether the objectives of the programme of work have been achieved*
   iii) *The amount of harm caused to animals*
   iv) *Any lesson(s) that can be learnt from the programme of work which may contribute to the further implementation of the principles of 3Rs.*

c. Ongoing HBA of PPLs during Inspection. One of the key activities undertaken during Inspection is to check work against the authorities and conditions of the PPL under which it is being conducted. This contributes to the ongoing HBA of a particular project. It is a means of ensuring the accuracy of the harms described in the application in relation to the subject animals’ actual experiences and contributes to the Inspector’s understanding of the model and its effects on the animals. Progress towards the PPL’s objectives is also inspected (e.g. discussion of publications). This may contribute to the HBA for other applications using similar animal models. Retrospective assessment and reporting of actual severity also make a significant contribution to achieving this

d. PPL Standard Condition 18 Notifications. PPL Standard Condition 18 requires PPL holders to notify the HO if the controls described in the PPL or the severity limits appear to have been, or are likely to be, breached. A further HBA will need to be undertaken if the harms need to be more severe than originally predicted in order to achieve the objectives.

Annex A: Current ASRU-I PPL assessment form

OFFICIAL-SENSITIVE
(when completed)

ANNEX A TO HBA PAPER

Published: 09 Oct 2015
For Review: Dec 2015

ASSESSMENT OF PROJECT LICENCE APPLICATION

For each question answer yes/no/not applicable as appropriate and insert text if necessary or expected.

PPL Number	
Title	
Applicant	

Is the title suitable?
Yes ☐ No ☐

Continuation of existing project?
Yes ☐ No ☐

Is the application low risk (see below)?

**If the application falls into all categories below, it is considered to be a low risk licence. Low risk licences only require the harm-benefits section of the assessment form to be filled in. They should not normally require a second iteration from the applicant.**	**Tick box where applicable**
1. Low risk establishment by Risk Based Inspection criteria	☐Yes
2. Species do not include special, endangered, wild caught or feral species	☐Yes
3. Simple plan of work e.g. fewer than seven protocols, clear workflow, no complex Continued Use	☐Yes
4. Work does not include Re-Use, re-homing, setting free at the end of or during procedures, or use of Neuromuscular Blocking Agents.	☐Yes
5.Non-recovery and mild to moderate severity protocols only	☐Yes
6. Does not involve CNS, pain or cardiac disease models	☐Yes
7. All disease models are standard and in common use	☐Yes
8. Work falls outside of all mandatory referral categories	☐Yes
9. If it is a renewal, group has no history of infringements in the past 3 years and work has been inspected at least once	☐Yes
10. Does not require Retrospective Assessment	☐Yes

OFFICIAL-SENSITIVE
(when completed)
Contains personal information, subject to confidentiality requirements under the Data Protection Act. Not for general release beyond ASRU without prior permission.
All government information may be subject to an FOIA request and subsequent assessment.

PROJECT SUMMARY CONSIDERATION

Is the project summary acceptable?
Yes ☐ No ☐

Is retrospective assessment of this project mandatory? (Non Human Primate, dog, cat and Equidae work, work involving endangered animals, protocol(s) classified as severe, Education and Training licences?)
Yes ☐ No ☐

When should review take place?

Person (PPLH and team)

Is the PPLH suitable, taking into account the points listed in section 5C (2)?
Yes ☐ No ☐

Is the team suitable?
Yes ☐ No ☐

Place

Primary
Are the facilities, infrastructure and staffing suitable for the proposed work?
Yes ☐ No ☐

Contains personal information, subject to confidentiality requirements under the Data Protection Act. Not for general release beyond ASRU without prior permission.
All government information may be subject to an FOIA request and subsequent assessment.

Additional establishment(s)

Are the facilities, infrastructure and staffing suitable for the proposed work?

Yes ☐ No ☐ N/A ☐

POLE (Place other than a Licensed Establishment)

Is an adequate scientific reason given for any work taking place at a POLE?

Yes ☐ No ☐ N/A ☐

Is the location of any work at a POLE adequately described?

Yes ☐ No ☐ N/A ☐

Programme of Work

Is the need for the work adequately explained and justified?

Yes ☐ No ☐

Contains personal information, subject to confidentiality requirements under the Data Protection Act. Not for general release beyond ASRU without prior permission.

All government information may be subject to an FOIA request and subsequent assessment.

Do any parts of the proposed work fall outside the permissible purposes as specified in A(SP)A 1986?
Yes ☐ No ☐
Indicate which permitted purposes are covered by the proposed work

☐ (a) basic research;

(b) translational or applied research with one of the following aims—

☐ (I) the avoidance, prevention, diagnosis or treatment of disease, ill-health or other abnormality, or their effects, in man, animals or plants;
☐ (II) the assessment, detection, regulation or modification of physiological conditions in man, animals or plants; or
☐ (III) the improvement of the welfare of animals or of the production conditions for animals reared for agricultural purposes;

☐ (c) the development, manufacture or testing of the quality, effectiveness and safety of drugs, foodstuffs and feed-stuffs or any other substances or products, with one of the aims mentioned in paragraph (b);

☐ (d) the protection of the natural environment in the interests of the health or welfare of man or animals;

☐ (e) research aimed at preserving the species of animal subjected to regulated procedures as part of the programme of work;

☐ (f) higher education or training for the acquisition, maintenance or improvement of vocational skills;

☐ (g) forensic inquiries.

Is the programme of work adequately specified, described and justified?
Yes ☐ No ☐

Contains personal information, subject to confidentiality requirements under the Data Protection Act. Not for general release beyond ASRU without prior permission.
All government information may be subject to an FOIA request and subsequent assessment.

Could the purpose of any part of the programme specified in the application be achieved satisfactorily by any other scientifically satisfactory alternative method not entailing the use of protected animals?
Yes ☐ No ☐

Is the use of species of special interest (cats, dogs, primates, equidae, endangered species, animals taken from the wild and feral animals) justified?
Yes ☐ No ☐ N/A ☐

If animals are to be captured from the wild, is the method used one that does not cause avoidable pain suffering distress or lasting harm?
Yes ☐ No ☐ N/A ☐

Are there appropriate arrangements for examining and minimising the suffering of an animal taken from the wild and found to be injured or in poor health, before regulated procedures regulated procedures are applied?
Yes ☐ No ☐ N/A ☐

If not, is the justification satisfactory for not treating such animals?
Yes ☐ No ☐ N/A ☐

Contains personal information, subject to confidentiality requirements under the Data Protection Act. Not for general release beyond ASRU without prior permission.
All government information may be subject to an FOIA request and subsequent assessment.

Does the proposed re-use of animals that have undergone previous procedures comply with s14 of ASPA 1986 and is it justified?
Yes ☐ No ☐ N/A ☐

Does any proposed use of neuromuscular blocking agents comply with s17 of ASPA 1986 and HO Guidelines?
Yes ☐ No ☐ N/A ☐

Does the re-homing or setting free of animals at the end of their use in regulated procedures meet the requirements of S17A of ASPA 1986?
Yes ☐ No ☐ N/A ☐

Do any additional measures need to be applied?
Yes ☐ No ☐ N/A ☐

Regulated procedures (protocols)

The applicant will have proposed severity classifications for each protocol. You need to confirm the severity classification of each protocol as non-recovery, mild, moderate or severe using the criteria in Annex VIII of Directive 2010/63/EU – specify the number of protocols under each classification.

Non-recovery	Mild	Moderate	Severe

Are the proposed regulated procedures clearly described?
Yes ☐ No ☐

Contains personal information, subject to confidentiality requirements under the Data Protection Act. Not for general release beyond ASRU without prior permission.
All government information may be subject to an FOIA request and subsequent assessment.

Does the proposed programme of work:

- use the minimum number of protected animals without compromising the objectives of the programme? ;

Yes ☐ No ☐

- involve animals with the lowest capacity to experience pain, suffering, distress or lasting harm;

Yes ☐ No ☐

- cause the least pain, suffering, distress or lasting harm; and

Yes ☐ No ☐

- appear most likely to produce satisfactory results?

Yes ☐ No ☐

Are the controls for adverse effects adequate and severity classifications appropriate?

Yes ☐ No ☐

If non-Schedule 1 methods of killing are used, are they scientifically justified to the effect that the objectives could not be achieved by the use of Schedule 1 methods instead?

Yes ☐ No ☐

Contains personal information, subject to confidentiality requirements under the Data Protection Act. Not for general release beyond ASRU without prior permission.
All government information may be subject to an FOIA request and subsequent assessment.

Fate of animals (if not to be killed according to A(SP)A Schedule 1 or under non-recovery anaesthesia)

☐ Continued use
☐ Re-use
☐ Kept alive at the establishment under the supervision of the NVS
☐ Set free or re-homed
☐ Other (please specify below)

Is the fate of animals not killed at the end of procedures legal and justified?

Yes ☐ No ☐ N/A ☐

Harm Benefit Assessment

What benefits are expected from the project?

Summarise the nature of the harms to the animals, and their expected incidence, severity and duration (assuming the specified end points and controls are applied).

Contains personal information, subject to confidentiality requirements under the Data Protection Act. Not for general release beyond ASRU without prior permission.
All government information may be subject to an FOIA request and subsequent assessment.

Summary of harm-benefit analysis

PART B: REFERRALS

Internal review sought?
Yes ☐ No ☐

If yes, please use internal referral form (copy form to assessment PI)

Additional ASRU inspector comments

Name	Date

Referral to Animals in Science Committee

Reason for referral:

- ☐ proposed use of wild-caught, non-human primates;
- ☐ proposed use of cats, dogs, equidae or non-human primates in protocols of severe severity;
- ☐ proposed use of endangered animals
- ☐ it is a project with major animal welfare or ethical implications, involving:
  - ☐ xeno-transplantation of whole organs;
  - ☐ chronic pain models in severe severity protocols;
  - ☐ study of the central nervous system in protocols of severe severity;
- ☐ it raises novel or contentious issues, or may give rise to serious societal concerns
- ☐ proposed use of admixed embryos falling into category 3 of the Academy of Medical Sciences report on Animals Containing Human Material and category 2 where the predominance of an admixed embryo is unclear or uncertain;

Contains personal information, subject to confidentiality requirements under the Data Protection Act. Not for general release beyond ASRU without prior permission.
All government information may be subject to an FOIA request and subsequent assessment.

Referral to external assessor

Inspector: Date:

PART C: RECOMMENDATION

Recommendation

- ☐ Grant licence
- ☐ Apply additional conditions (see below)
- ☐ Retrospective assessment in X years/ No retrospective assessment

Licensing to BF 3 months before RA date

- ☐ Refuse application

Duration of licence

Additional Information including Additional Conditions

Contains personal information, subject to confidentiality requirements under the Data Protection Act. Not for general release beyond ASRU without prior permission.
All government information may be subject to an FOIA request and subsequent assessment.

Basis of recommendation to grant licence (where applicable)

I have taken into account the whole application and my additional knowledge. In my professional opinion a licence granted on the terms advised above and incorporating Parts A to E of this application as the Schedule will satisfy the relevant criteria of the Animals (Scientific Procedures) Act 1986.

**Inspector:**	
**Signature:**	
**Date:**	

Spell Check

Annex B: References

1. Russell W.M.S and. Burch R.L. (1959) "The Principles of Humane Experimental Technique" Universities Federation for Animal Welfare (UFAW).

2. EU Directive 2010/63/EU of the European Parliament and of the Council of 22nd September 2010 on the protection of animals used for scientific purposes

3. Project Evaluation and Retrospective Assessment, EU Commission's Expert Working Group for Project Evaluation and Retrospective Assessment (2013).

4. Guidance on the harm-benefit analysis of project licence applications (Appendix I) in Guidance on the Operation of the Animals (Scientific Procedures) Act (2014) pp125-130

5. Animals Procedures Committee: Review of Cost-Benefit Assessment in the Use of Animals in Research June 2003.

6. Report of the Technical Committee to Enquire into the Welfare of Animals kept under Intensive Livestock Husbandry Systems, the Brambell Report, December 1965.

7. The Farm Animal Welfare Council (FAWC), "Five Freedoms" Press Notice 5th December 1979

8. The Animals (Scientific Procedures) Act 1986 Chapter 14 Amendment Regulations 2012

Annex C: ASRU-I's Internal Referral Policy Document

Published: 20 Oct 2015
To be reviewed: 02 Oct 2016

For Information

ASRU Inspectorate Referral Process for Licence Applications and Amendments

Part 1 Internal Referrals

Note: All referrals below are mandatory and must be made prior to final recommendation to grant.

**Project Licences**		
**Referral Category**	**Notes**	**Refer to**
**History of non-compliance**	**Licence application from a person whose previous licence(s) was revoked as a result of breaches of the Act**	**Principal Inspector**
**Modular training exemption**	Request for training **e**xemption	Principal Inspector
**Tobacco**	Project application or amendment request involving the use of tobacco or tobacco products in regulated procedures	Chief Inspector
**Weapons**	Project application or amendment request for the development or testing of weapons	Chief Inspector
**Ascites**	Project application or amendment request for the production of monoclonal antibodies by the ascites method	Principal Inspector
**Education and Training**	Project application for education and training (including microsurgical training)	Responsible Inspector
**Cephalopods**	Project application or amendment request involving regulated procedures on cephalopods	Responsible Inspector
**Traps**	Project application or amendment request involving the testing of animal traps	Principal Inspector
**Pesticides**	Project application or amendment request involving the testing of pesticides	Responsible Inspector
**Severe Severity**	Project application or amendment request with protocol(s) of severe severity classification only where advice required on refinement of harm-benefit analysis	Any Inspector

Referral Category	Notes	Refer to
**Discharge of genetically-altered animals from ASPA**	Request for the discharge from the controls of ASPA of genetically altered animals to remain within the UK	Responsible Inspector
**Genetically-altered farm animals**	Project application or amendment request for the use of genetically altered farm animals	Responsible Inspector
**Genetically-modified food**	Project application or amendment request involving safety testing of genetically-modified food	Chief Inspector
**Sourcing non-purpose bred non-human primates**	Request for the acquisition for use in regulated procedures, or breeding or supply, of non-human primates that have not been purpose bred for procedures (not including wild caught)	Responsible Inspector
**Non-standard acute oral test**	Project application or amendment request involving the determination of acute oral toxicity using protocols other than OECD Test Guideline 420, 423, 425	Inspector Regulatory Toxicology Group
**Skin corrosivity or phototoxicity tests**	Project application or amendment request involving the use of protected animals for the determination of skin corrosivity or phototoxicity	Inspector Regulatory Toxicology Group
**Non Local Lymph Node Assay skin sensitivity test**	Project application or amendment to request the use of guinea pig assay for the determination skin sensitisation potential	Inspector Regulatory Toxicology Group
**Non-standard eye irritancy test**	Project application or amendment to request the testing for eye irritancy (Draize eye test) by a protocol not conforming to the Home Office guidelines	Inspector Regulatory Toxicology Group
**Import or export of surgically-prepared animals**	Request for the import or export of surgically-prepared animals	Responsible Inspector
**Strychnine**	Project application or amendment request involving the use of strychnine in conscious animals	Responsible Inspector
**Wild Birds**	Project application or amendment request involving the use of wild birds	Responsible Inspector
**Establishment Licences**		
**Referral Category**	**Notes**	**Refer to**
**Breeders/suppliers**	Application for the licensing of commercial breeders and suppliers	Responsible Inspector
**Non-Schedule 1 methods of killing**	Application for the use of humane killing methods that are not listed on Schedule 1 of ASPA	Responsible Inspector
**AWERB**	Application for new Establishment Licence or changes to the AWERB of existing Establishment Licences	Responsible Inspector

ISBN:

ISSN:

© Crown copyright 2015

This publication is licensed under the terms of the Open Government Licence v3.0 except where otherwise stated. To view this licence, visit nationalarchives.gov.uk/doc/open-government-licence/version/3 or write to the Information Policy Team, The National Archives, Kew, London TW9 4DU, or email: psi@nationalarchives.gsi.gov.uk.

Where we have identified any third party copyright information you will need to obtain permission from the copyright holders concerned.
